# Medication for Acromegaly Reduces Expression of MUC16, MACC1 and GRHL2 in Pituitary Neuroendocrine Tumour Tissue

**DOI:** 10.3389/fonc.2020.593760

**Published:** 2021-02-15

**Authors:** Rihards Saksis, Ivars Silamikelis, Pola Laksa, Kaspars Megnis, Raitis Peculis, Ilona Mandrika, Olesja Rogoza, Ramona Petrovska, Inga Balcere, Ilze Konrade, Liva Steina, Janis Stukens, Austra Breiksa, Jurijs Nazarovs, Jelizaveta Sokolovska, Valdis Pirags, Janis Klovins, Vita Rovite

**Affiliations:** ^1^ Latvian Biomedical Research and Study Centre, Riga, Latvia; ^2^ Riga East Clinical University Hospital, Riga, Latvia; ^3^ Riga Stradins University, Riga, Latvia; ^4^ Pauls Stradins Clinical University Hospital, Riga, Latvia; ^5^ University of Latvia Faculty of Medicine, Riga, Latvia

**Keywords:** somatostatin/dopamine (SSA/DA) therapy, acromegaly, transcriptome, next generation sequencing (NGS), somatotropinoma

## Abstract

Acromegaly is a disease mainly caused by pituitary neuroendocrine tumor (PitNET) overproducing growth hormone. First-line medication for this condition is the use of somatostatin analogs (SSAs), that decrease tumor mass and induce antiproliferative effects on PitNET cells. Dopamine agonists (DAs) can also be used if SSA treatment is not effective. This study aimed to determine differences in transcriptome signatures induced by SSA/DA therapy in PitNET tissue. We selected tumor tissue from twelve patients with somatotropinomas, with half of the patients receiving SSA/DA treatment before surgery and the other half treatment naive. Transcriptome sequencing was then carried out to identify differentially expressed genes (DEGs) and their protein–protein interactions, using pathway analyses. We found 34 upregulated and six downregulated DEGs in patients with SSA/DA treatment. Three tumor development promoting factors *MUC16, MACC1*, and *GRHL2*, were significantly downregulated in therapy administered PitNET tissue; this finding was supported by functional studies in GH3 cells. Protein–protein interactions and pathway analyses revealed extracellular matrix involvement in the antiproliferative effects of this type of the drug treatment, with pronounced alterations in collagen regulation. Here, we have demonstrated that somatotropinomas can be distinguished based on their transcriptional profiles following SSA/DA therapy, and SSA/DA treatment does indeed cause changes in gene expression. Treatment with SSA/DA significantly downregulated several factors involved in tumorigenesis, including *MUC16, MACC1*, and *GRHL2.* Genes that were upregulated, however, did not have a direct influence on antiproliferative function in the PitNET cells. These findings suggested that SSA/DA treatment acted in a tumor suppressive manner and furthermore, collagen related interactions and pathways were enriched, implicating extracellular matrix involvement in this anti-tumor effect of drug treatment.

## Introduction

Pituitary neuroendocrine tumors (PitNETs) are benign intracranial endocrine tumors with potentially high prevalence in the population. Clinically significant PitNET affects approximately 0.1% of the general population, but clinically insignificant or undiagnosed PitNET can be found in approximately 16.7% (1, 2).

Somatotropinoma, which develops from anterior pituitary somatotroph cells, are characterized by increased synthesis and secretion of growth hormone (GH). They constitute 10–15% of all clinically significant somatotropinomas and usually cause acromegaly in adults or gigantism in children with additional comorbidities (3). Acromegaly is a rare and chronic endocrine disorder and is characterized by abnormal growth of the extremities, cardiovascular diseases, and metabolic disorders, such as diabetes mellitus that is caused by increased levels of insulin growth factor 1 (IGF-1), activated by high GH levels (4).

The goal of therapeutic strategies for this condition is to normalize GH and IGF-1 levels, remove tumor mass and/or stabilize tumor growth while maintaining normal pituitary function (5). Somatotropinoma resection is recommended as the primary therapy, but often medical treatment is chosen as the first-line therapy to reduce tumor mass (4, 5). After PitNET resection, often the drug treatment is then used to better control the disease (5). Somatostatin receptors (SSTRs) are the main targets for this therapy, and 90% of somatotropinomas express predominantly SSTR2 and SSTR5 subtypes. If somatostatin analogs (SSAs) fail to control IGF-1 levels, dopamine agonists (DAs) that target dopamine receptor 2 (D2R) are used as complementary management options (6). Although SSA and DA have demonstrated an inhibitory effect on the secretion of both hormones and cell proliferation, approximately one-third of acromegalic patients are resistant to cabergoline and octreotide treatment (6).

The molecular mechanisms involved in the downstream signaling and pharmacological resistance after SSA/DA therapy are still poorly understood (6). Their principle inhibitory effect on hormone secretion is mediated through the inhibition of calcium channels and activation of potassium channels (through SSTR1, SSTR2, SSTR5) and also by inhibition of the adenylyl cyclase-cAMP signaling pathway (through SSTR3, SSTR4) (7, 8). However, stimulation of other second messenger signaling molecules, such as protein tyrosine phosphatases, plays an important role in controlling growth caused by somatostatin/somatostatin receptor ligands that prevent cell proliferation by inhibition of the phosphoinositide 3-kinase (PI3K)/AKT signaling pathway, mainly through SSTR2 or *via* the inhibition/activation of MAPK signal pathways by SSTR1, SSTR2, and SSTR5 (8).

Studies have found that long-term SSA therapy is associated with lower beta-arrestin expression levels (9, 10), by potentially working as natural desensitization factors for the SSTR, and expression profiles of PitNETs can change due to SSA treatment. It has been demonstrated that several factors that act as tumor suppressors and influence gene expression in PitNET development, such as aryl hydrocarbon receptor-interacting protein (AIP) and tumor suppressor gene PLAG1 like zinc finger 1 (ZAC1), modulate the antiproliferative effects of SSA (11–13). SSAs can induce an increase in the expression of AIP and ZAC1 (14, 15), suggesting that SSA treatment affects factors related to PitNET development and clinical parameters, that could affect not only molecular signaling but also the transcriptional profiles of PitNET cells.

In this study, we compared differences in the total transcriptome of PitNET patients with and without SSA/DA treatment before surgery. Our objective was to determine whether the transcriptome signature could distinguish those acromegaly patients that had been on therapy with those that had not, and also to identify candidate genes whose expression is affected by SSA/DA treatment.

## Materials and Methods

### Sample and Data Collection

All somatotropinoma tissue samples were obtained from patients who underwent planned resections at Pauls Stradins Clinical University Hospital, Latvia. All patients were recruited from the Genome Database of the Latvian population (LGDB), a government-funded national biobank (16), and all biobank activities and research in this article comply with the Declaration of Helsinki. Broad informed consent for LGDB and project-specific consent for research involving the pituitary tumors were obtained from all patients. Both the biobank and PitNET research was approved by the Central Medical Ethics Committee of Latvia (protocol No. 22.03.07/A7 and 01.29.1/28, respectively). Sample collection and the research process both complied to the Declaration of Helsinki. After resection, PitNET tissue samples were stored in RNAlater^®^ Solution (Thermo Fisher Scientific, USA) and stored for up to 24 h at +4°C and then at −20°C for up to 2 months, until DNA/RNA extraction.

### Total RNA Extraction From Pituitary Neuroendocrine Tumor Tissue

Twenty to 30 mg of PitNET tissues was homogenized using a FastPrep-24™ homogenizer in RLT Plus buffer in Lysing Matrix D 1.5 ml tubes (MP Biomedicals, USA), and total RNA was extracted with AllPrep DNA/RNA Mini Kit (Qiagen, Germany) and stored at −80°C. Total RNA quality control was carried out using Agilent 2100 bioanalyzer and an Agilent RNA 6000 Pico Kit (Agilent Technologies, USA), and concentration was measured using Qubit 2.0 fluorometer and Qubit™ RNA HS Assay Kit (Thermo Scientific, USA).

### Transcriptome Library Preparation and Sequencing

Transcriptome libraries were prepared with the Low Input RiboMinus™ Eukaryote System v2 and Ion Total RNA-Seq Kit v2 (Thermo Fisher Scientific, USA) following the manufacturer’s instructions. The average concentration of total RNA was 88.15 ng/µl (range: 35.2–138 ng/µl). Input amount for the transcriptome library preparation was 500, ng and the average library concentration was 23.55 ng/µl (8.91–31.8 ng/µl) with an average fragment length of 214 bp (189–236 bp). The preparation of transcriptome libraries was carried out in relation to 1:1 based on patient samples who had or had no medical therapy before PitNET resection, to reduce any batch effects. Libraries were sequenced at the Latvian Biomedical Research and Study centre, Genome Centre Core facility using the Ion Proton™ System for Next-Generation Sequencing (Ion OneTouch and Ion PI™ Hi-Q™ OT2 Reagents 200 Kit for emulsion PCR and Ion PI™ Hi-Q™ Sequencing 200 Solutions kit and Ion PI™ Chip V3 chips for sequencing, all from Thermo Fisher Scientific, USA). Libraries were sequenced three to four times to acquire at least 20 M reads per sample, and at least 10 M would represent uniquely mapped reads, so that the rRNA content would not reach 50% of the sample. For sample PA05, 20 M of total reads was not achieved, and uniquely mapped reads were close to 7 M. Nevertheless, the relevant sample was included in the data analysis based on a 2014 study by Liu et al., analyzing the effects of read number and biological repetitions on differentially expressed genes (DEGs). They concluded that by increasing the amount of biological repetitions independent of the number of reads from the sample library, they were able to increase the number of DEGs and logFC resulting in increased accuracy of expression levels and greater efficiency in the resulting analysis (17).

### Data Analysis

Transcriptome data has been deposited in an online repository (GEO Submission GSE160195). Differentially expressed genes were obtained using the DESeq2 (*v1.26.0*) package (18) from the Bioconductor (*v3.10*) project (19) and R (*v3.6.1*) software (www.r-project.org). Read counts were first filtered based on read count frequency in all samples. Genes with at least ten reads in at least three samples were included in the analysis. To detect sample outliers, read count data were transformed with variant stabilizing transformations (VSTs), considering the design of the experiment and visualized with sample distance heat mapping, PCA and MDS methods. Read count density visualization was used to check for problematic samples. Counts were replaced with trimmed mean values for genes which were marked as outliers based on their dispersion Cook’s distance values, which were calculated as the.99 percentile of the F (p, m − p) distribution for each gene. To account for batch effects, surrogate variables (SVAs) were calculated using the sva (*v3.34.0*) package (Bioconductor project) and added to the design matrix. DESeq function call was then used on raw data but filtered by read count frequency counts to detect DEGs. “Independent Hypothesis Weighing” (IHW) was specified as the independent filtering method, which is not the default in DESeq2, to gain statistical power for testing, using the IHW (*v1.14.0*) package (20), which uses the base mean value of counts for each gene, in this case, as a weight for the adjusted p-value calculation. Next, we performed log fold shrinkage with the apeglm (*v1.8.0*) package (21), which uses the heavy tailed Cauchy prior distribution, to account for extra variability that comes from genes with low counts and high dispersion values. All parameters were left at their default values, except for “coef” which lets us denote which design coefficient we want to perform shrinkage on. Genes with FDR <0.05 and transformed absolute logFC value >1.5 were deemed as significant and selected. To visualize differential expression results, a heatmap was constructed with the pheatmap (*v1.0.12*) package using the differences from means across all samples of the VST normalized counts for each gene. Heatmap data was clustered both by genes and samples using the kmeans algorithm. A volcano plot was also plotted with the Enhanced Volcano (*v1.4.0*) package using the same log fold shrinkage values obtained from apeglm. Kendall’s Tau correlation analysis between read count matrix and Knosp classification index and between read count matrix and Ki-67 proliferation index was performed to test whether these factors affect the expression levels of detected DEG’s and whether they should be included in the model (22). To further assess potential relationships between the significant differentially expressed genes and their link to a common signaling pathway or functional protein–protein interaction, enrichment analysis was carried out between statistically significant DEGs, using the STRING (*v11.0*) database (23), with the significance threshold set at 0.4. Experiments, databases, co-expression, neighborhood, gene fusion and co-occurrences were all set as sources for active interactions. The enrichment was performed by calculating the probabilities for each of the selected evidence sources, a prior was set to account for random interactions between two proteins and then the probabilities are added. A homology correction was added to the co-occurrence score. Finally, we annotated the DEGs using the Gene Ontology (GO) (*release: 2020-01-01*) (24) and GO enrichment analysis tools (25) and functional annotation tool from the DAVID Bioinformatics Resources 6.8 database (26), as well as gene set enrichment analysis (GSEA) with fgsea (v1.14.0) (minGSsize = 10, maxGSsize = 1,000) and clusterProfiler (v3.16.1) packages, from the Bioconductor project. All scripts used in the study are in **Supplementary Material 1** and deposited in GitHub portal (https://github.com/rsak-384/PitNET-after-therapy).

### Selection of the Validation Cohort

We searched online data repositories and the literature for PitNET transcriptome data, with SSA and/or SSA/DA treatment status before surgery. From the studies closely matching our own, we found transcriptomes of ten somatotropinomas with and without SSA/DA therapy, from the “Pangenomic Classification of Pituitary Neuroendocrine Tumors” study (27) which were carefully chosen using the “ArrayExpress” archive of functional genomics data. Both therapy and nontherapy group samples were screened using the mentioned metadata, to ensure that the samples were as similar as possible between both groups, in terms of clinical data, in order to maximize the likelihood of detecting a true signal.

### Cell Line Culture and Stimulation

The rat pituitary GH3 cell line was obtained from ATTC. GH3 cells were maintained in F-12K medium supplemented with 2.5% fetal bovine serum, 15% horse serum, penicillin (100 U/ml) and streptomycin (100 µg/ml).Cells were cultured at 37°C in a humidified incubator containing 5% CO_2_. For the gene and protein expression experiments, cells were grown on a 6-well plate at a density of 1.5 × 10^6^ cells/well and treated with 0.1 µM and 1 µM octreotide for 4, 8, and 24 h.

### RNA Extraction and Quantitative Real-Time Polymerase Chain Reaction

Total RNA was isolated from GH3 cells usingTRIzol reagent, according to the manufacturer’s protocol. First-strand cDNA was synthesized from 1.6 µg RNA with RevertAidH Minus kit (ThermoFisherScientific). qRT-PCR was performed using Absolute Blue SYBR green MasterMix reagent (ThermoFisherScientific) with a ViiA7 real-time PCR detection system (AppliedBiosystems). The primer pairs that were employed for MUC16 were forward 5′-GCCTAGGAAGAACCAAAACTCA-3′ and reverse 5′-TCCAATGTGTAGTTCCCCAGT-3′; for GRHL2 were forward 5′-CCTCTGCCTGAGTCAAGACC-3′ and reverse 5′-TAGGAGCTGTGGCTGGCTAT-3′; for MACC1 were forward 5′- CCTGGATGCCTTAGGTGGTA-3′ and reverse 5′-CCCACCCAGGACTCTGATTA-3′; for GUSB were forward 5′-GACTGATCCTTCCATGTATCCCA-3′ and reverse 5′-CCCGCATAGTTGAAGAAGTCG-3′. mRNA levels were quantified and normalized to levels of reference gene GUSB using the 2^−ΔΔCt^ method and presented as relative expression compared with values of untreated cells.

### Western Blot

Total protein samples from control and octreotide treated GH3 cells were extracted by RIPA buffer (50 mM Tris-HCl; pH 8.0, 150 mM NaCl, 5 mM EDTA, 1% Igepal CA-630, 0.5% sodium deoxycholate, 0.1% SDS, and supplemented with Halt™ protease inhibitor cocktail; Thermo Fisher Scientific). Protein concentration was quantified with BCA Protein Assay reagent (Thermo Fisher Scientific), according to the manufacturer’s instructions. Lysates (45 µg) were electrophoresed through a 10% SDS-PAGE gel and transferred to Hybond-C-extra nitrocellulose membranes (Amersham Biosciences) for 45 min in a semi-dry transfer system. Membranes were blocked with 5% non-fat milk in TBS-T buffer (20 mM Tris, pH 7.6, 137 mM NaCl, and 0.05% Tween 20) for 1 h at room temperature. Membranes were incubated with 1 µg/ml MACC1(PA5-20758, Thermo Fisher Scientific) and 1 µg/ml *β*-actin (ab8224, Abcam) primary antibodies for 1 h at room temperature and incubated with horseradish peroxidase-conjugated anti-mouse IgG (sc-2005, Santa Cruz Biotechnology) and anti-rabbit (sc-2004, Santa Cruz Biotechnology) secondary antibodies for 1 h at room temperature. Membranes were then washed in TBS-T buffer three times and developed with Pierce ECL western blotting substrate (Thermo Fisher Scientific). UVP software VisionWorks LS (Thermo Fisher Scientific) was used to capture signals.

## Results

### Characterization of the Study Group

For all the patients within this study (**Table 1**), it was their first time diagnosis of somatotropinoma, displaying clinical symptoms of acromegaly (ICD-10, E22.0). One patient (PA11) had tumor with complementary prolactin expression (**Supplementary Material Figure 1**). All patients had primary PitNET having a tumor over 10 mm, at least in one dimension. Patient’s mean age at the time of diagnosis of PitNET was 43 years (age distribution varied from 22 to 65 years). Ten were females, and two were males. Three patients had SSA/DA therapy and three SSA treatment before PitNET resection, and six patients did not have SSA/DA treatment. We are fully aware that the use of DAs in our study group was more frequent than usual. This could be explained by the fact that not all patients agreed to surgical therapy immediately, and therefore, the preoperative treatment period in most cases in our cohort was longer than 3–6 months. In turn, if IGF-1 remains modestly elevated during SSA administration, the proposed algorithm recommends adding cabergoline to the treatment plan.

**Table 1:** Clinical characteristics of PitNET patients.

Patient code	Age at diagnosis	Gender	Diagnosis according to clinical features	Tumor size/Knosp grade	KI-67/Cytokeratin granulation pattern	Medical therapy before sampling/time
PA01	41	F	Somatotropinoma	Macro/2	<1%/no data	SSA, DA/2 years
PA02	25	F	Somatotropinoma	Macro/1	<1%/sparsely	SSA/1 year
PA03	31	M	Somatotropinoma	Macro/4	5%/no data	SSA/3 month
PA04	31	F	Somatotropinoma	Macro/1	5%/sparsely	SSA, DA/2 years
PA05	40	M	Somatotropinoma	Macro/0	<1%/no data	SSA, DA/2 years
PA06	22	F	Somatotropinoma	Macro/4	1%/densely	SSA/3 month
PA07	60	F	Somatotropinoma	Macro/1	3–4%/sparsely	No
PA08	53	F	Somatotropinoma	Macro/1	<1%/no data	No
PA09	65	F	Somatotropinoma	Macro/4	12%/densely	No
PA10	53	F	Somatotropinoma	Macro/2	1–2%/no data	No
PA11	31	F	Somatotropinoma co-secreting prolactin	Macro/3	9%/densely	No
PA12	62	F	Somatotropinoma	Macro/1	3%/no data	No

### Differentially Expressed Genes

**Figure 1:**
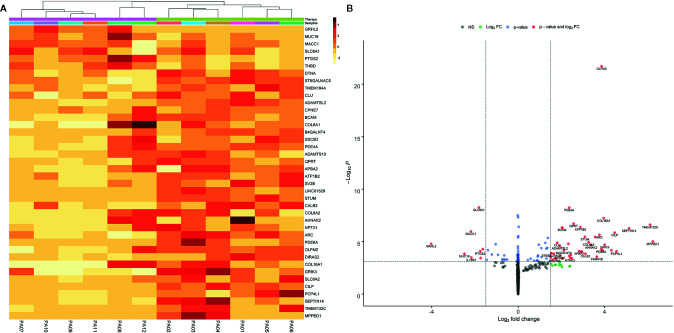
Heatmap **(A)** of the statistically significant differentially expressed genes. Heatmap intensities were obtained by filtering apeglm transformed log fold change values with the following thresholds: abs. lfc >1.5 and padj <0.05. The color scale represents the approximate difference in each individual sample from the average, vst normalized read counts for that gene. Data is clustered both by genes and samples using the kmeans algorithm included in the pheatmap package. Volcano plot **(B)** of differential expression results with apeglm transformed log fold change values. Dashed vertical lines represent absolute log fold change threshold of 1.5 and horizontal dashed line represents p values threshold of 1.31e-4 or FDR ~0.05. Red dots represent significantly DEGs (FDR < 0.05 and absolute L2FC > 1.5).

A total of 18,266 genes remained after sequencing read count filtering, based on the presence of gene expression in at least three samples. Raw counts were replaced for 691 genes as they were flagged as outliers. Three statistically significant surrogate variables were detected with the SVA package and were included in the analysis model. Differential expression analysis between the non-therapy group and the therapy group with DESeq2 and log fold shrinkage with the apeglm algorithm detected 40 DEGs after applying thresholds of logFC >1.5 and P-adj <0.05. In total 34 (85%) DEGs were upregulated with a median logFC of 3.02 (IQR = 1.73), and six (15%) genes were downregulated with a median logFC of −2 (IQR = 0.64). Even though two distinct sample outliers were apparent in MDS, PCA and sample distance heatmap plots (**Supplementary Material Figures 2**, **3** and **4**), they were not removed because both samples passed sample quality evaluation using FastQC. Two distinct sample groups were observed in the heatmap (**Figure 1A**) that were derived from clustering. These visual results are concordant with the two patient groups, with and without therapy, that were used in the study design.

Volcano plot using apeglm transformed log-fold change values presents the distribution of differentially expressed genes based on their log fold changes while being controlled for genes with low counts or abnormally high dispersion values. These manipulations confirmed that most of the DEGs had increased expression when comparing samples receiving therapy to those with no therapy (**Figure 1B**).

**Figure 2:**
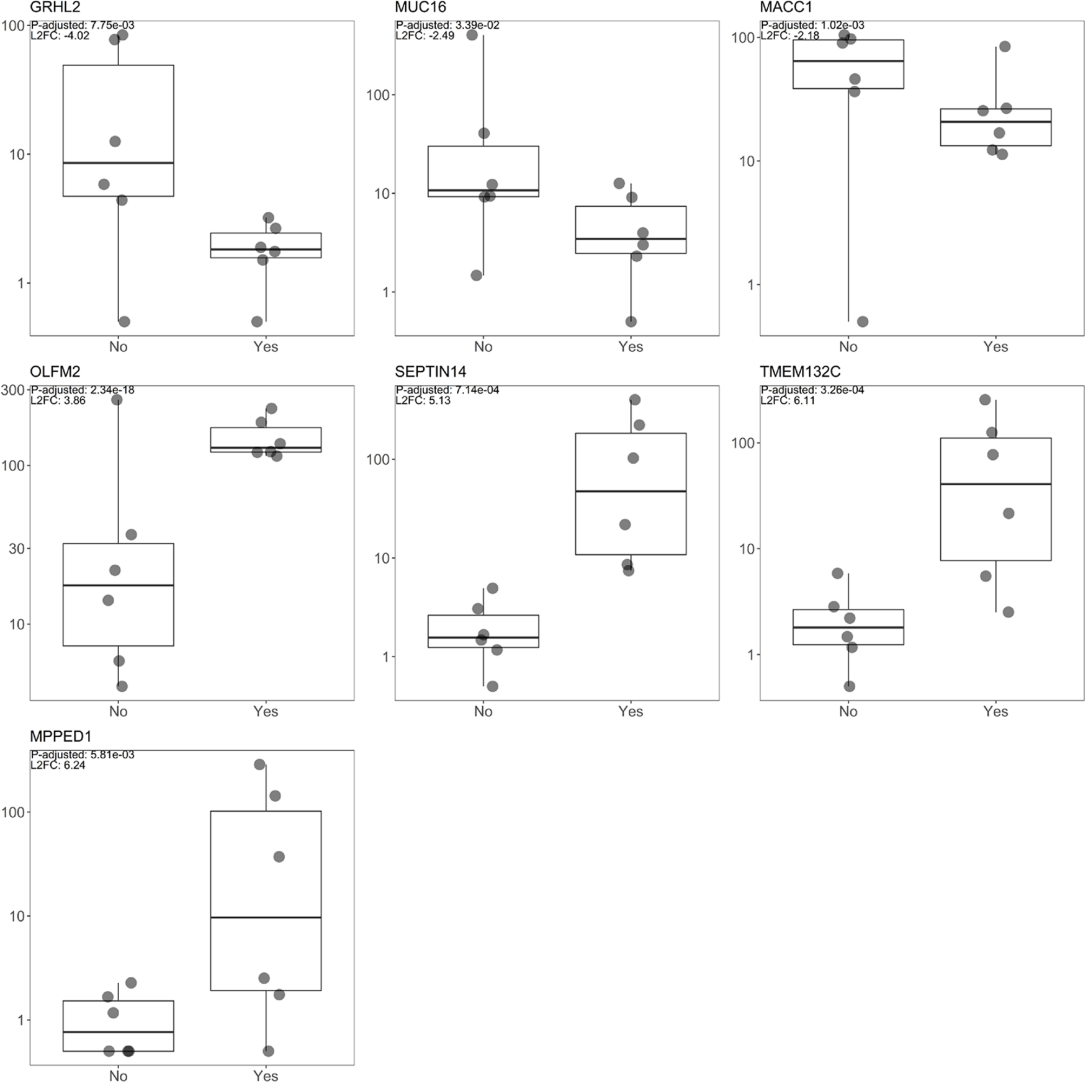
Box plot diagrams displaying raw read count distribution at the log10 scale for both the SSA/DA treated and untreated groups for the seven DEGs (*OLFM2*, *MACC1, GRHL2, MUC16, TMEM132C, SEPTIN14, MPPED1*), with the top L_2_FC positive and negative changes and for DEGs with the lowest FDR value (*OLFM2*).

The three most upregulated genes in the treated group were metallophosphoesterase domain containing 1 (*MPPED1)*, transmembrane protein 132C (*TMEM132C)*, and septin 14 (*SEPTIN14*); in contrast, the three most downregulated genes in the SSA/DA and SSA therapy groups were grainyhead like transcription factor 2 (*GRHL2)*, mucin 16 (*MUC16*), and MET transcriptional regulator (*MACC1—Metastasis-Associated in Colon Cancer*) (**Table 2**, **Figure 2**). Furthermore, *OLFM2* displayed the most consistent statistically significant differential upregulation in all the SSA/DA and SSA therapy group samples as indicated by low variance, high logFC difference, and P-value. logFC, P values, and box plots for all the significant DEGs are presented in **Supplementary Material Tables 1** and **2**.

**Table 2:** Top three differentially expressed genes based on their logFC values.

Gene symbol	Gene name	logFC	lfcSE	P-adjusted	Expression
*MPPED1*	Metallophosphoesterase Domain Containing 1	6.236	1.898	0.0058	Upregulated
*TMEM132C*	Transmembrane Protein 132C	6.109	1.325	0.0003	Upregulated
*SEPTIN14*	Septin 14	5.134	1.242	0.0007	Upregulated
*MACC1*	MET Transcriptional Regulator MACC1	−2.181	0.512	0.0001	Downregulated
*MUC16*	Mucin 16, Cell Surface Associated	−2.485	0.839	0.0339	Downregulated
*GRHL2*	Grainyhead Like Transcription Factor 2	−4.017	1.126	0.0077	Downregulated

By performing Kendall’s Tau correlation analysis between the read count matrix and Knosp classification index and Ki-67 proliferation index, no coherent correlation trend could be observed across all DEG’s for either of the potential factors; therefore there is no reason for their inclusion in the differential expression model **Supplementary Material Table 3**).

### Protein–Protein Interactions and Pathway Analysis

**Figure 3:**
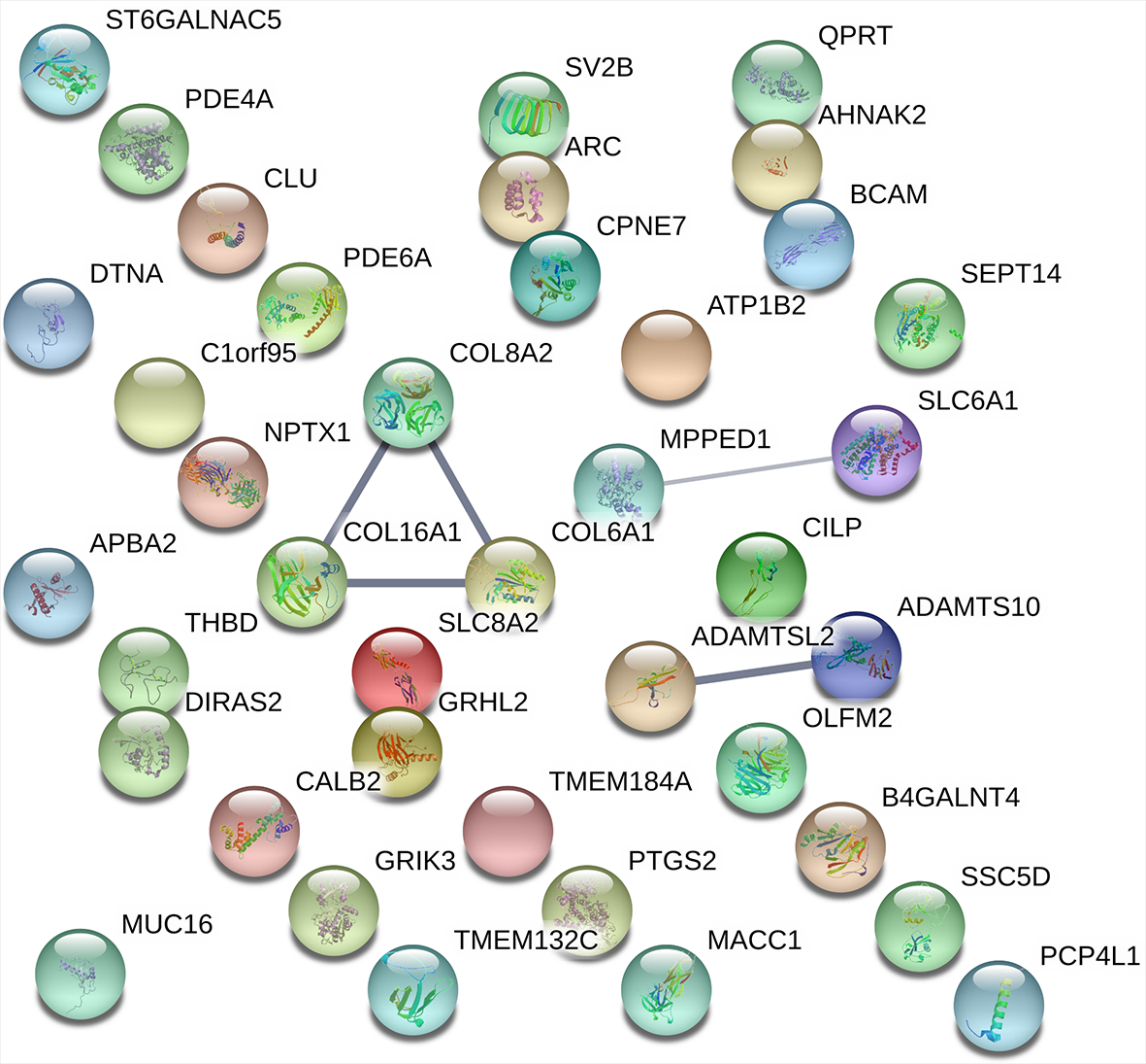
Visual representation of protein–protein interaction enrichment analysis between the statistically significant 39 out of 40 DEGs using STRING (*v11.0*) database. The line thickness between the connected nodes indicates the strength of the supporting data. Experiments, databases, co-expression, neighborhood, gene fusion, and co-occurrence.

By visualizing protein–protein interactions between all of the significant DEGs using STRING database, we were able to identify five potential interactions between the proteins of the following genes: *COL8A2*, *COL16A1*, *SLC8A2*, *COL6A1*, *SLC6A1*, *ADAMTSL2*, and *ADAMTS10*. According to the database protein interaction analysis results, the number of expected interactions (edges) in the network was one, but the achieved number was five, with a PPI enrichment p-value of 0.00022, meaning that our network holds more reciprocal interactions than would be expected if the same amount of random proteins where to be drawn from the genome (**Figure 3**).

The protein for the gene *LINC01529* could not be identified for *Homo Sapiens*; therefore, it was not included in the resulting network hence the final network consisted of protein interactions for 39 DEGs. Specific sources and their contribution for each pair of interactions are presented in **Supplementary Material Table 4**.

To determine which molecular mechanisms could be affected by the DEGs, GO enrichment analysis was performed along with functional annotation using DAVID. Several genes from the submitted list of DEGs (FDR < 0.05) were found to be significantly enriched in multiple pathways categorized as a part of the “Cellular component” annotation data set. The majority of these results are associated with either the plasma membrane, extracellular matrix (ECM), or synapses (**Table 3**).

**Table 3:** GO enrichment “cellular component” data set top results enriched by SSA/DA therapy in PitNET patients, ranked by statistical significance.

Gene Ontology ID	Description	Number of involved genes	Enrichment fold	Genes	P-value	FDR
GO:0062023	Collagen-containing extracellular matrix	8/424	10.1	CILP, CLU, COL16A1, ADAMTS10, BCAM, COL8A2, COL6A1, SSC5D	1.10^−6^	0.001
GO:0031012	Extracellular matrix	9/566	8.5	ADAMTSL2, CILP, CLU, COL16A1, ADAMTS10, BCAM, COL8A2, COL6A1, SSC5D	8.64^−7^	0.002
GO:0045202	Synapse	12/1309	4.9	ARC, SV2B, NPTX1, SLC6A1, DTNA, ATP1B2, CLU, SLC8A2, GRIK3, APBA2, OLFM2, CALB2	3.07^−6^	0.002
GO:0043005	Neuron projection	12/1386	4.6	ARC, SV2B, PTGS2, NPTX1, SLC6A1, DTNA, CLU, SLC8A2, GRIK3, APBA2, PDE6A, CALB2	5.52^−6^	0.003
GO:0098590	Plasma membrane region	11/1228	4.8	ARC, PDE4A, PTGS2, SLC6A1, ATP1B2, SLC8A2, THBD, GRIK3, PDE6A, OLFM2, CALB2	1.10^−5^	0.004
GO:0071944	Cell periphery	24/5954	2.2	ARC, SV2B, PDE4A, PTGS2, NPTX1, MUC16, SLC6A1, DTNA, ATP1B2, CLU, CPNE7, TMEM184A, SLC8A2, THBD, BCAM, GRIK3, SEPT14, AHNAK2, COL6A1, DIRAS2, APBA2, PDE6A, OLFM2, CALB2	2.06^−5^	0.007
GO:0120025	Plasma membrane bounded cell projection	14/2256	3.3	ARC, SV2B, PDE4A, PTGS2, NPTX1, SLC6A1, DTNA, ATP1B2, CLU, SLC8A2, GRIK3, APBA2, PDE6A, CALB2	3.32^−5^	0.010
GO:0042995	Cell projection	14/2354	3.2	ARC, SV2B, PDE4A, PTGS2, NPTX1, SLC6A1, DTNA, ATP1B2, CLU, SLC8A2, GRIK3, APBA2, PDE6A, CALB2	5.33^−5^	0.013
GO:0030054	Cell junction	13/2073	3.4	ARC, SV2B, NPTX1, SLC6A1, DTNA, ATP1B2, CLU, SLC8A2, GRHL2, GRIK3, APBA2, OLFM2, CALB2	6.34^−5^	0.014
GO:0042383	Sarcolemma	4/138	15.5	DTNA, COL6A1, SLC8A2, AHNAK2	1.40^−4^	0.028
GO:0005886	Plasma membrane	22/5837	2.0	ARC, SV2B, PDE4A, PTGS2, NPTX1, MUC16, SLC6A1, DTNA, ATP1B2, CPNE7, TMEM184A, SLC8A2, THBD, BCAM, GRIK3, AHNAK2, COL6A1, DIRAS2, APBA2, PDE6A, OLFM2, CALB2	2.27^−4^	0.041
GO:0005788	Endoplasmic reticulum lumen	5/313	8.5	PTGS2, CLU, COL16A1, COL8A2, COL6A1	2.98^−4^	0.046
GO:0098793	Presynapse	6/504	6.4	SV2B, SLC6A1, SLC8A2, GRIK3, APBA2, CALB2	3.38^−4^	0.049
GO:0016020	Membrane	30/9948	1.6	ARC, TMEM132C, SV2B, PDE4A, PTGS2, NPTX1, B4GALNT4, MUC16, ADAMTSL2, SLC6A1, DTNA, ATP1B2, CLU, CPNE7, TMEM184A, SLC8A2, THBD, BCAM, GRHL2, GRIK3, AHNAK2, COL6A1, DIRAS2, STUM, SSC5D, APBA2, PDE6A, OLFM2, ST6GALNAC5, CALB2	2.96^−4^	0.050

By performing KEGG pathway analysis as a part of DAVID, we obtained one significantly (FDR < 0.05) enriched pathway for protein digestion and absorption. However, by performing Reactome pathway analysis in the same run, we obtained multiple significant hits, which were associated with collagen processes, glycosylation, and cell surface interactions (**Table 4**).

**Table 4:** Pathway enrichment analysis results (FDR < 0.05).

Database/algorithm	Pathway ID	Description	Number of involved genes	Genes	FDR or P value
KEGG/DAVID	hsa04974	Protein digestion and absorption	3/90	*ATP1B2, COL6A1, SLC8A2*	FDR=0.0451
Reactome/DAVID	R-HSA-8948216	Collagen chain trimerization	3/44	*COL16A1, COL6A1, COL8A2*	FDR=0.0134
Reactome/DAVID	R-HSA-3906995	Diseases associated with O-glycosylation of proteins	3/64	*COL16A1, COL6A1, COL8A2*	FDR=0.0193
Reactome/DAVID	R-HSA-216083	Integrin cell surface interactions	3/83	*COL16A1, COL6A1, COL8A2*	FDR=0.0193
Reactome/DAVID	R-HSA-1442490	Collagen degradation	3/65	*ADAMTS10, ADAMTSL2, MUC16*	FDR=0.0193
Reactome/DAVID	R-HSA-5173105	O-linked glycosylation	3/108	*ADAMTS10, ADAMTSL2, MUC16*	FDR=0.0241
Reactome/DAVID	R-HSA-196807	Nicotinate metabolism	2/31	*PTGS2, QPRT*	FDR=0.0296
Reactome/DAVID	R-HSA-5173214	O-glycosylation of TSR domain-containing proteins	2/37	*ADAMTS10, ADAMTSL2*	FDR=0.0356
Reactome/DAVID	R-HSA-5083635	Defective B3GALTL causes Peters-plus syndrome (PpS)	2/38	*ADAMTS10, ADAMTSL2*	FDR=0.0356
Reactome/DAVID	R-HSA-5578775	Ion homeostasis	2/53	*ATP1B2, SLC8A2*	FDR=0.0499
GSEA/KEGG	hsa04974	Protein digestion and absorption	6/56	*SLC8A2, COL16A1, COL8A2, ATP1B2, COL6A2, ELN*	P= 5.12 E-5
GSEA	GO:0097447	GO dendritic tree	17/466	*SLC8A2, GRIK3, ARC, CALB2, APBA2, NOS1, JPH4, DPYSL5, ADGRB1, CLU, DBN1, CYP46A1, P2RX6, MAPT, KNDC1, MAPK8IP3, MARK4*	P =0.018
GSEA	GO:0005201	GO extracellular matrix structural constituent	8/104	*CILP, COL16A1, COL8A2, COL6A1, MFAP4, ELN, CHADL, LAMB2*	P=0.005
GSEA	GO:0060713	GO-labyrinthine-layer-morphogenesis	1/13	*GRHL2*	P=0.018
GSEA	GSEA: M7327	Linn pas 4 targetsdn	5/50	*ARC, NPTX1, SV2B, CPNE7, FAM131A*	P=0.02
GSEA	GSEA:M5884	Naba core matrisome	11/175	*CILP, COL16A1, COL8A2, NTNG1, COL6A1, MFAP4, ELN, VWA5B2, SSPOP, CHADL, LAMB2*	P=5.1 E-5

When applying GSEA analysis six signaling pathways were detected as significantly enriched (P-adjusted < 0.05) in the discovery cohort with the Molecular Signature Database (v7.2) (MSigDB) hallmark gene sets, and one signaling pathway was detected when using the KEGG gene set from the cluster Profiler package. Protein digestion and absorption were the only signaling pathway to overlap when using results from DAVID and those of GSEA. We also assessed the involvement in signaling pathways related to cell proliferation, growth, and apoptosis of individual DEGs identified in our study (full pathway list in **Supplementary Material Table 5**) and identified that QPRT, SEPTIN14, CLU, PTGS2 are involved in following pathways ALCALA_APOPTOSIS, GO_CELL_CYCLE, HALLMARK_APOPTOSIS, GO_CELL_CYCLE and GO_RESPONSE_TO_TUMOR_NECROSIS_FACTOR (**Supplementary Material Table 5**).

### Validation by Independent Data Set

To test for biological reproducibility of the experiment, the aforementioned 10 samples were run through the exact same workflow to determine differential gene expression detection as before, resulting in 88 significant DEGs (FDR < 0.05 and logFC> ± 1.5), 70 (79.55%) of which were upregulated, with a median logFC of 2.71 (IQR = 1.67) and 18 (20.45%) downregulated, with a median logFC of −2.1 (IQR = 0.59). From these 85 DEGs, amyloid beta precursor protein binding Family A member 2 (APBA2) was the only one differentially expressed in both discovery and validation cohorts, with logFC of 2.83 in the discovery cohort and 3.31 in the validation cohort.

Gene set enrichment analysis was performed on the validation cohort to test whether there may be any overlapping signaling pathways between both sets of samples. No overlapping signaling pathways, however, were detected although 478 signaling pathways were found in the publication repository data when using the MSigDB hallmark set and five when using the KEGG pathways analysis (27).

Discovery set DEGs were also cross validated with leading edge genes from the significant validation cohort pathways. Three genes were confirmed to be involved in multiple pathways, which were THBD, BCAM, and APBA2 (**Supplementary Material Table 6**).

### Functional Characterization in GH3 Cells

**Figure 4:**
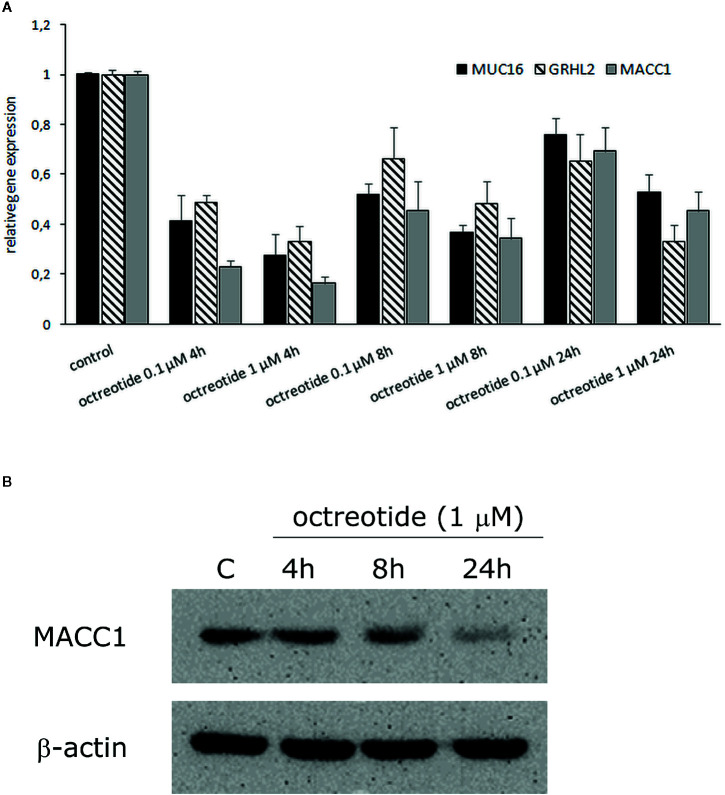
Effect of octreotide on *MUC16*, *GRHL2*, and *MACC1* gene expression in GH3 cells. **(A)** Cells were treated with 0.1 µM and 1 µM octreotide for 4, 8, and 24 h. Gene expression was analyzed by real-time PCR and expressed relative to value from untreated control cells, which was defined as 1. Data were normalized to GUSB and values were calculated using a comparative (2-ΔCt) method. Results are mean ± SD from two independent experiments. **(B)** Western blot analysis of *MACC1* protein expression in octreotide treated GH3 cells. *β*-actin was used as a loading control. The experiments were performed twice with similar results.

To functionally characterize potential effects of SSA on *MUC16*, *GRHL2*, and *MACC1*, we treated GH3 cells with octreotide and observed changes in expression by real-time PCR for all three candidates and Western blot for *MACC1*. The results demonstrate that octreotide inhibits RNA expression of all three factors with most inhibition after 4 h of treatment. The MACC1 protein expression was also inhibited after octreotide stimulation and the most decrease in *MACC1* level was observed after 24 h (**Figure 4**).

## Discussion

The first-line medical management of somatotropinomas is the use of SSAs, causing reduced tumor cell proliferation and hormone secretion. If the control of IGF-1 is not achieved, DAs are added to the treatment regimen. The targets of this therapy are SSTRs and D2Rs that alter intracellular signaling to promote an antiproliferative effect, and there is indirect evidence that this may lead to altered gene transcription. However, the effects of SSA/DA medication on the overall cellular transcriptional profile and consequent functional changes have not been elucidated. This is one of the first reports that have evaluated transcriptome profiles from PitNET tumor tissue in two groups of patients with and without medical therapy, which was applied before tumor resection. As a result, we were able to distinguish tumor preoperative treatment status based on transcriptome profiles. We found 40 DEGs potentially affected by SSA/DA treatment, with significant downregulation seen in several tumorigenesis promoting factors. We extended these findings by applying pathway analysis, which revealed an alteration in the expression of collagen related genes.

Many studies have analyzed the influence of SSA on cell signaling components, and these involve major effects on SSTR1, SSTR2, and SSTR5 binding and alteration of the PI3K/AKT and MAPK pathways (8). It has also been demonstrated the SSA can reduce the Ki-67 proliferation index in tumor tissue from acromegaly patients (28), but the underlying causes were not clear. The only study to date that has assessed the impact of SSA as a pre-operative treatment on transcriptome profiles from PitNETs has been carried out on three somatotropinomas and was compared to untreated PitNETs (29). The authors detected decreased Ki-67 expression in the pre-treated group and increased *MUC1* and *CD40* expression (29). In our data, we did not observe a significant correlation of Ki-67 with treatment status; however, we observed that overall read counts in PitNET patients without treatment were higher (**Supplementary Material Table 7**); to identify more pronounced association the larger sample group would be required but the tendency could be observed.

Our data indicated that three genes having pronounced effects on tumorigenesis were strongly downregulated by SSA/DA in somatotropinoma tissues (*MUC16, MACC1*, and *GRHL2*) and confirmed these findings in functional GH3 cell experiments. *MUC16* is a high molecular weight O-glycosylated protein. Elevated levels of *MUC16* in the blood serves as a prognostic biomarker for ovarian cancer (30). *MUC16* has also been implicated in the development of other neoplasms and as a potential marker in pancreatic, breast and lung cancers (31–33).

The second candidate, *MACC1* was involved in epithelial–mesenchymal transition and was potent regulator of cancer metastasis and cell invasion. Increased expression of *MACC1* leads to greater proliferation, induction of angiogenesis, and is antiapoptotic in various cancers (34, 35). Therefore, over the twofold decrease of *MACC1*expression observed in our data due to SSA/DA treatment is in line with previous results in the literature.

The third factor identified in this study *GRHL2* was DNA-binding nuclear protein, containing a highly conserved DNA-binding domain (36). It was involved in the regulation of various developmental processes such as closure of the neural tube and the regulation of progenitor cell functions during the development of the pituitary gland. *GRHL2* is expressed in pituitary progenitor cells and in mice with decreased pituitary progenitor cell numbers (37). *GRHL2* has been implicated in many cancers such as breast, lung, colorectal, gastric, ovarian, and prostate. However, its role as tumor promoter or as an anti-tumor factor appears to be tissue dependent (36).

The three genes that were most strongly upregulated by SSA/DA treatment in somatotropinoma tissues were *MPPED1, TMEM132C*, and *SEPTIN14*, potentially having more indirect evidence for their involvement in anti-tumor effect.

The only study looking at the role for *MPPED1* has found that it is involved in the development of the central nervous system (38). No other functional studies have been performed on this candidate. However, as a member of metallophosphoesterase proteins, the related *MPPED2* has been shown to have pronounced tumor suppressor activities. Downregulation of *MPPED2* has been demonstrated in neuroblastoma and breast cancer (39, 40). *MPPED1* and *MPPED2* show over 71% similarity based on Ensembl.org data, but to determine whether they have related functions needs further investigation.

Information concerning a potential function for the second upregulated candidate, *TMEM132C*, is modest. TMEM132 family proteins are thought to be involved in cell adhesion (41), while other members of this protein family have been associated with insomnia, hearing loss, panic disorder, and anxiety behavior (41). Somatic variants in the related factor *TMEM132D* have been found in small-cell lung and pancreatic cancer (42, 43). One report has implicated the cg04475027 methylation site on *TMEM132C*, as a marker for breast cancer (44).

The third factor, *SEPTIN14* belongs to the septin protein family of GTP-binding membrane-interacting proteins and has a function in cytoskeleton organization, cytokinesis, apoptosis, cell polarity, and cell cycle regulation (45). Studies have found that aberrant expression of septin, may induce antiproliferative and tumor suppressive effects (46, 47), and somatic variants of *SEPTIN14* have been demonstrated in skin and gastrointestinal cancers (48).

As SSA treatment effect induces shrinkage of the tumor, it would be expected that SSA leads to alterations in cell proliferation, growth, apoptosis, and related pathways. Indeed, four of the individual DEGs (*QPRT, SEPTIN14, CLU, PTGS2*) in our study are involved in these pathways (**Supplementary Material Table 5**); it shows that there are perturbed parts of the relevant cell proliferation pathways, but what effect these DEGs constitute on whole pathway in the cells of PitNETs remains to be discovered.

The altered expression of *CLU* gene that encodes for custerin involved in apoptosis and clearance of cell debris has been also identified related to invasiveness traits in two PitNET studies (49, 50). We, however, did not observe this on the pathway level in the results of enrichment analysis and protein–protein interactions, but our data supported ECM related pathways (**Table 4**). Further investigation is needed to elucidate whether the period of SSA/DA treatment could influence the intrinsic transcriptomic regulation of PitNETs over the course of the therapy. As at the very start of the therapy, the signaling pathways could be active that slow the tumor growth, but afterwards, other pathways involved in the reduction of tumor mass that could involve the ECM reorganization could be more pronounced. It has been shown that the most reduction of tumor mass occurred during the first year of SSA treatment (5). These assumptions should be further studied as there is only one study to date considering the impact of SSA preoperative treatment of PitNET transcriptome (29). This study has also found altered expression of one of the mucin family factors *MUC1*, while in our study we found *MUC16* as one of the most significant DEGs.

Other transcriptomic studies that could be compared to our results are reports that have assessed the invasiveness of the PitNETs. The invasiveness could also be related to increased cell growth, and in PitNETs, it has been demonstrated that resistance to SSA is accompanied with reduced tumor shrinkage (51). In the transcriptome studies comparing invasive and non-invasive PitNETs, there are various candidates identified that are related to cell proliferation, growth, apoptosis pathways, but still rarely these candidates overlap between the studies (49, 50, 52–58) (**Supplementary Material Table 5**). These numerous studies demonstrate various intrinsic mechanisms that in large scale studies lead to heterogeneity in the observed DEGs. Mostly this difference arises from variation in design and diagnoses, but also notable is the usage of small groups to obtain conclusions. Small group comparison (which is one of the weaknesses of this study) is more likely to lead to spurious findings, although it’s clear that obtaining large homogeneous groups of the rare heterogeneous condition is a challenging task in itself. The lack of reproducibility in these literature data could also be attributed to sample size variation and use of the different methodologies.

Although the majority of DEGs detected here were upregulated (34 DEGs) after treatment, three of six genes that were downregulated appear to have functions more related to tumor pathogenesis, according to current literature. Thus, we propose that the influence of SSA/DA treatment at the transcriptome level was directed toward suppression of tumor promoting genes (*GRHL2, MUC16, MACC1*), and the protective effects of the upregulated DEGs were small or indirect (*MPPED1, TMEM132C, SEPTIN14*); however, further studies are needed to confirm this hypothesis.

Interestingly, olfactomedin 2 (*OLFM2*) was found to be significantly upregulated in all tumor tissues from the SSA/DA treated group (**Figure 1B**). *OLFM2* is involved in vascular remodeling, scar tissue formation in blood vessels, and smooth muscle differentiation. Elevated levels of *OLFM2* can be found in blood plasma interventional therapy with postoperative restenosis (59–61). Vascular plasticity is an important hallmark of malignant tumors (62), but whether SSA administration can lead to blood vessel remodeling events in PitNET tissue, promoting protective anti-tumoral effects needs to be functionally assessed. Furthermore, *OLFM2* is involved in interactions with the transcription factor Runx2 (59), which has been reported to be involved in the regulation of pituitary tumor growth (63, 64).

Protein–protein interactions and pathway analysis revealed potential interactions between *COL8A2*, *COL16A1*, *SLC8A2*, *COL6A1*, *SLC6A1*, *ADAMTSL2*, *ADAMTS10* (**Figure 3**) and several significantly enriched pathways belonging to the “Cellular component” category (**Table 3**), which again demonstrated the lowest FDR scores for pathways including *COL16A1, COL6A1*, and *COL8A2* suggesting collagen related effects upon SSA/DA treatment (**Table 4**).

Collagens are key components of the ECM and can affect the behavior of tumor cells, for example proliferation, differentiation, motility, and metastasis. Tumor mass usually consists of tumor cells and stroma, which is composed of the ECM. Tumor cells and stroma interact with each other and affect tumorigenesis and cellular characteristics. It has been reported that PitNETs can have elevated fibrotic scar tissue primarily composed of stromal collagens (65–67). Furthermore, research on collagen-producing cells in PitNETs and normal pituitary suggests that properties of collagen production during tumorigenesis can change, leading to the formation of fibrosis in PitNETs (68). Interestingly, collagen type VI alpha 6 (COL6A6) is downregulated in PitNETs, and overexpression of this collagen caused anti-tumoral effects and decreased metastatic capacity (69).

Some studies investigating preoperative SSA administration have demonstrated that this treatment helps to reduce tumor mass and soften the tumor tissue, facilitating tumor resection (70, 71). However, other reports have not observed this (72), or whether the tissue softening could be related to ECM alterations. Overall, our data suggested that ECM remodelling, in particular collagen regulation, might be involved in SSA/DA treatment, but further investigations are still required to determine the underlying interactions.

The ECM is an essential part of the tumor microenvironment, that along with other participating factors, may affect cell signaling, molecular transport, metabolic pathways, and immune resistance mechanisms largely contributing to tumor growth and invasiveness (73). Some tumors are composed of even up to 60% of ECM that is due to fibroblast infiltration that produces excessive amounts of ECM and is associated with worth prognostics (74, 75). Various collagen dysregulation has been linked with tumor properties of many malignant cancers (76, 77). In our study the SSA/DA treatment could impact the expression of these proteins due to remodeling and restructuration of the ECM; both ADAMTS proteins and collagens could be responding to treatment induced reduction of the tumor mass, but further studies are needed to elaborate this hypothesis.

We also discovered “protein digestion and absorption” with both pathway enrichment methods that might imply molecular mechanisms’ underlying effects of SSA/DA treatment. Besides tumor microenvironment, also metabolic reprogramming could affect properties of tumor cells. It has been recognized that alterations in metabolic regulation of tumor cells occur that drives accelerated proliferation, growth, and survival *via* an increase in glycolysis, amino acid, and lipid metabolism, and mitochondrial biogenesis (78, 79). There are many studies considering the role of conventionally administered medications (metformin, sulfasalazine, proton pump inhibitors and other) for non-malignant disorders that target metabolic reprogramming of tumors and have beneficial effects on cancer treatments (78, 79); whether SSA could have a similar effect is an interesting topic for investigation. It is known that SSA has effects on PI3K/Akt signaling pathway (8) that is one of the important factors inducing cancer metabolic reprogramming (78). Therefore, the SSA could exert its activity on beneficial anti-tumoral effects *via* shifts in tumor cell metabolism, but this needs to be investigated further.

From our computational validation of the data from online available data sets of PitNET tissue transcriptomes, from patients with and without therapy (27), we discovered one candidate factor and (*APBA2*) several potentially involved pathways (related to *THBD*, *BCAM* and *APBA2*). *THBD* is expressed on endothelial cells that were found to be involved in blood coagulation (80). Further investigations are still needed, however, in order to determine the role of these factors during SSA/DA treatment. *BCAM* is a plasma membrane glycoprotein that can bind ECM proteins and has been reported to be expressed in both fetal and adult rat pituitary (81), however, the precise role of this protein in the pathogenesis and therapy of PitNET needs further study. The only candidate that was found to be differentially expressed in our results and in validation data is *APBA2*; this protein interacts with amyloid precursor proteins which are involved in the development of Alzheimer’s disease. It has been reported that *APBA2* is involved in gonadotropin-releasing hormone-1 neuronal migration to the pituitary and neurogenesis (82). The decreased expression of *APBA2* has been identified in superior temporal gyrus in schizophrenia patients (83) and gender-specific alterations in the expression of *APBA2* have been found in dopamine neurons (84). According to Protein Atlas information, this protein is involved in signal transduction and vesicular trafficking in the central nervous system, and these actions could be related to different levels of PitNET functioning, development, or progression as well as response to SSA/DA treatment. But the precise functionality of *APBA2* within the nervous system signaling or trafficking as well as the pathogenesis of PitNETs is not clear and needs to be further studied.

The potential candidates involved in SSA/DA effects in our data are not directly linked to previous reports relating to resistance mechanisms of SSA. We did not find any significant profile changes in SSTR, beta-arrestin, or cytoskeleton protein filamin A that was associated with drug resistance mechanisms (85). It has also been reported that SSTR2 and D2DR agonists can reduce migration and invasiveness of PitNET cells that are mediated *via* cofilin and filamin A mechanisms (86–88). We did not observe a significant alteration in expression of these factors, however, in our transcriptome data. Nevertheless, our results indicated novel factors targeted by SSA/DA (*GRHL2, MUC16, MACC1)* that merit further exploration, so as to characterize their role in SSA responder and non-responder groups that could give more insight into the relation of these factors to drug resistance.

The limitation of this study was the small number of samples used; however, our main goal was to demonstrate that SSA/DA could cause distinct alterations in transcriptome profiles from PitNET tumors, and this has been convincingly demonstrated. Although the results indicate proof of principle and highlight some of the novel factors involved in the antiproliferative effects of SSA/DA treatment, it is also clear that an increased sample size would strengthen our observation.

In conclusion, we have detected changes in transcriptional profiles induced by SSA/DA therapy in PitNET tissue. The tumor promoting factors *MUC16, MACC1* and *GRHL2* were downregulated in PitNETs after SSA/DA therapy and in GH3 cell following octreotide treatment. Collagen related interactions were detected after analyses of pathways and enrichment, implicating ECM involvement in the anti-tumoral effects of drug treatment. Further functional analyses are needed to determine the impact of these molecules and their potential role in response to SSA/DA treatment in patients with PitNET.

## Data Availability Statement

The datasets presented in this study can be found in online repositories. The names of the repository/repositories and accession number(s) can be found below: GEO, GSE160195.

## Ethics Statement

The studies involving human participants were reviewed and approved by the Central Medical Ethics Committee, Ministry of Health of the Republic of Latvia. The patients/participants provided their written informed consent to participate in this study.

## Author Contributions

RS performed the data analysis and wrote the manuscript writing. IS performed the data analysis. PL and KM prepared the sequencing library and wrote the manuscript. RPec performed the data analysis and data interpretation. IM, OR, and RPet carried out the functional experiments. IB and LS obtained the clinical data. IK obtained the clinical data and wrote the manuscript. JSt performed PitNET resection and obtained the tumor tissue. AB and JN performed the ICH characterization. JSo participated in the study design and manuscript writing. VP and JK participated in the study design and manuscript editing. VR participated in the study design, data interpretation, and manuscript writing. All authors contributed to the article and approved the submitted version.

## Funding

This research was supported by the European Regional Development Fund within the project “Molecular markers of pituitary tumor development, progression and therapy response” (1.1.1.1/16/A/066). 

## Conflict of Interest

The authors declare that the research was conducted in the absence of any commercial or financial relationships that could be construed as a potential conflict of interest.
