# Supplementary material for: Medication for Acromegaly Reduces Expression of MUC16, MACC1 and GRHL2 in Pituitary Neuroendocrine Tumour Tissue
Source: Front Oncol. 2021 Feb 15;10:593760. doi: 10.3389/fonc.2020.593760 (PMC7928352; doi:10.3389/fonc.2020.593760)
Supplement: Supplementary file 1 [file DataSheet_1.docx]

Supplementary Material 1. Scripts used in the data analysis of the study.

### Loading required packages

library(DESeq2)

library(RColorBrewer)

library(pheatmap)

library(sva)

library(IHW)

library(org.Hs.eg.db)

library(clusterProfiler)

library(fgsea)

library(EnhancedVolcano)

library(ggplot2)

library(grid)

library(gridExtra)

### Read in the count matrix and prepare Deseq2 data object

countdata = read.table('read_counts_18_02_2020.txt', row.names = 'Gene', header = TRUE)

countdata = countdata[ ,order(names(countdata))]

countdata = countdata[complete.cases(countdata), ]

Samples = c(colnames(countdata))

colnames(countdata)

Therapy = as.factor(c('Yes', 'Yes', 'Yes', 'Yes', 'Yes', 'Yes', 'No', 'No', 'No', 'No', 'No', 'No'))

colData = data.frame(Samples, Therapy)

ddsMat = DESeqDataSetFromMatrix(countData = countdata,

colData = colData,

design = ~ Therapy)

ddsMat$Therapy = factor(ddsMat$Therapy, levels=c('No', 'Yes'))

### Filter the count matrix

keep = rowSums(counts(ddsMat) >= 10) >= 3

ddsMat = ddsMat[keep, ]

### Draw sample distance hatmap, MDS and PCA plots

vsd = vst(ddsMat, blind = FALSE)

sampleDists = dist(t(assay(vsd)))

sampleDistMatrix <- as.matrix( sampleDists )

rownames(sampleDistMatrix) <- paste( vsd$Therapy, vsd$Samples, sep = ' - ')

colnames(sampleDistMatrix) <- NULL

colors <- colorRampPalette( rev(brewer.pal(9, "Blues")) )(255)

pheatmap(sampleDistMatrix,

clustering_distance_rows = sampleDists,

clustering_distance_cols = sampleDists,

col = colors)

mds <- as.data.frame(colData(vsd)) %>%

cbind(cmdscale(sampleDistMatrix))

ggplot(mds, aes(x = `1`, y = `2`, color = Samples)) +

geom_point(size = 5) + coord_fixed() + ggtitle("MDS with VST data - therapy") +

theme_grey(base_size = 17) +

labs(x = 'MDS component 1', y = 'MDS component 2')

plotPCA(vsd, intgroup = c("Therapy"))

theme_grey(base_size = 17)

plotPCA(vsd, intgroup = c("Samples"))

theme_grey(base_size = 17)

### Test for unaccounted batch effects using sva and add them to the design model

ddsMat1 = DESeq(ddsMat, minReplicatesForReplace = 6)

p <- ncol(attr(ddsMat1,"modelMatrix"))

m = ncol(ddsMat1)

cooksCutoff = qf(.99, p, m - p)

dat <- counts(ddsMat1, normalized = TRUE)

idx <- rowMeans(dat) > 1

dat <- dat[idx, ]

mod <- model.matrix(~ Therapy, colData(ddsMat1))

mod0 <- model.matrix(~ 1, colData(ddsMat1))

svseq <- svaseq(as.matrix(dat), mod, mod0)

svseq$sv

ddssva = ddsMat1

resultsNames(ddssva)

ddssva$SV1 = svseq$sv[,1]

ddssva$SV2 = svseq$sv[,2]

ddssva$SV3 = svseq$sv[,3]

design(ddssva) = ~ SV1 + SV2 + SV3 + Therapy

### Draw sample distance heatmap and PCA plots for the adjusted model

sampleDistMatrix_sva <- as.matrix( sampleDists_sva )

rownames(sampleDistMatrix_sva) <- paste(vsd_sva$Therapy, vsd_sva$Samples, sep = ' - ')

colnames(sampleDistMatrix_sva) <- NULL

colors <- colorRampPalette( rev(brewer.pal(9, "Blues")) )(255)

pheatmap(sampleDistMatrix_sva,

clustering_distance_rows = sampleDists_sva,

clustering_distance_cols = sampleDists_sva,

col = colors)

plotPCA(vsd_sva, intgroup = c("Therapy"))

plotPCA(vsd_sva, intgroup = c("Samples"))

### Perform differential expression analysis with DESeq2

dds = DESeq(ddssva, minReplicatesForReplace = 6)

p <- ncol(attr(dds,"modelMatrix"))

m = ncol(dds)

cooksCutoff = qf(.99, p, m - p)

res = results(dds, alpha = 0.05, cooksCutoff = cooksCutoff,

pAdjustMethod = 'BH', name = 'Therapy_Yes_vs_No',

filterFun = ihw)

resOrdered = res[order(res$padj), ]

resOrderedSignif = resOrdered[!is.na(resOrdered$padj) & resOrdered$padj < 0.05, ]

resOrderedSignif2 = resOrderedSignif[abs(resOrderedSignif$log2FoldChange) > 1.5, ]

### Perform log2FoldChange shrinkage using the 'apeglm' method

lfc_shrink = lfcShrink(dds, res = res, type = 'apeglm', coef = 'Therapy_Yes_vs_No')

res1Ordered = lfc_shrink[order(lfc_shrink$padj), ]

res1OrderedSignif = res1Ordered[!is.na(res1Ordered$padj) & res1Ordered$padj < 0.05, ]

res1OrderedSignif2 = res1OrderedSignif[abs(res1OrderedSignif$log2FoldChange) > 1.5,]

### Gather statistics for L2FC of upregulated and downregulated DEG`s

up = res1OrderedSignif2[res1OrderedSignif2$log2FoldChange > 0, ]

down = res1OrderedSignif2[res1OrderedSignif2$log2FoldChange < 0, ]

median_up = median(up$log2FoldChange)

median_down = median(down$log2FoldChange)

iqr_up = IQR(up$log2FoldChange)

iqr_down = IQR(down$log2FoldChange)

### Convert ENSEMBL keys to SYMBOL keys

ens.str = substr(res1OrderedSignif2[, 'Genes'], 1, 18266)

res1OrderedSignif2$Symbol = mapIds(org.Hs.eg.db,

keys = ens.str,

column = "SYMBOL",

keytype = "ENSEMBL",

multiVals = "first")

### Draw p-value histogram

hist(lfc_shrink$pvalue, col = "lavender", main = "Terapija: Ir vs Nav", xlab = "p-values")

### Perform GSEA analysis using the fgsea package and MSigDB Hallmark gene sets

lfc_shrink_table = as.data.frame(lfc_shrink)

lfc_shrink_table$ENSEMBL = rownames(lfc_shrink_table)

lfc_shrink_table = lfc_shrink_table[complete.cases(lfc_shrink_table), ]

translation_entrez = as.data.frame(bitr(c(lfc_shrink_table$ENSEMBL),

fromType = 'ENSEMBL', toType = 'ENTREZID', OrgDb = 'org.Hs.eg.db'))

translation_symbol = as.data.frame(bitr(c(lfc_shrink_table$ENSEMBL),

fromType = 'ENSEMBL', toType = 'SYMBOL', OrgDb = 'org.Hs.eg.db'))

lfc_shrink1 = merge(lfc_shrink, translation_entrez, by = 'ENSEMBL')

lfc_shrink2 = merge(lfc_shrink1, translation_symbol, by = 'ENSEMBL')

gseaDat = lfc_shrink2[which(duplicated(lfc_shrink2$ENTREZID) == F), ]

ranks = gseaDat$log2FoldChange

names(ranks) = gseaDat$ENTREZID

ranks = sort(ranks)

gmt_path = gmtPathways('msigdb.v7.1.entrez.gmt')

msig = fgsea(pathways = gmt_path, stats = ranks, minSize = 10, maxSize = 1000, eps = 0)

msig_df_sig = msig_df[msig_df$padj < 0.05, ]

### Perform GSEA analysis using the KEGG pathway set

ranks = sort(ranks, decreasing = TRUE)

gseaRes_KEGG = gseKEGG(geneList = ranks,

organism = 'hsa',

minGSSize = 10,

maxGSSize = 1000,

pvalueCutoff = 0.05)

gseaKEGG_results <- gseaRes_KEGG@result

gseaKEGG_results = as.data.frame(gseaKEGG_results)

### Create volcano plot of the relationship between logFoldChange and p-value

EnhancedVolcano(lfc_shrink,

lab = lfc_shrink$Symbol,

x = 'log2FoldChange',

y = 'pvalue',

FCcutoff = 1.5,

pCutoff = 7.68e-4,

xlim = c(-6.5, 6.5),

ylim = c(0, 11),

pointSize = 3.5,

labSize = 3.5,

caption = "",

title = "",

subtitle = "",

gridlines.major = FALSE,

gridlines.minor = FALSE)

### Create a heatmap to cluster samples and DEG`s by their expression similiarity.

draw_colnames_45 <- function (coln, gaps, ...) {

coord = pheatmap:::find_coordinates(length(coln), gaps)

x = coord$coord - 0.5 * coord$size

res = textGrob(coln, x = x, y = unit(1, "npc") - unit(3,"bigpts"), vjust = 1, hjust = 0.5, rot = 0, gp = gpar(...))

return(res)

}

assignInNamespace(x="draw_colnames", value="draw_colnames_45",

ns=asNamespace("pheatmap"))

heat_colors <- brewer.pal(9, "YlOrRd")

labels_row = c(res1OrderedSignif2$Symbol)

degs = c(res1OrderedSignif2$Genes)

mat <- assay(vsd_sva)[degs,]

mat = mat - rowMeans(mat)

anno <- as.data.frame(colData(vsd_sva)[, c("Samples","Therapy")])

pheatmap(mat, annotation_col = anno, fontsize = 10,

fontsize_row = 13, border_color = FALSE,

fontsize_col = 16, labels_row = labels_row, color = heat_colors, annotation_legend = FALSE)

### Draw boxplots for raw read count distribution for significant DEG`s

plot_sva_boxplots = function (dati) {

m <- estimateSizeFactors(dati)

sva_counts = as.data.frame(counts(m, normalized=TRUE))

head(sva_counts)

for (gene in res1OrderedSignif2$Genes) {

df = as.data.frame(t(sva_counts[gene, ]))

padj = formatC(res1OrderedSignif2[gene, 5], format = 'e', digits = 2)

log2fold = round(res1OrderedSignif2[gene, 2], 2)

symbol = res1OrderedSignif2[gene, 7]

names(df)[1] = 'Count'

df$Therapy = Therapy

df$Label = rownames(df)

df[, 1] = df[, 1] + 0.5

print(res1OrderedSignif2[gene, 7])

par(mfrow=c(1,1))

boxplots = ggplot(df, aes(x = Therapy, y = Count, color = Therapy)) +

geom_boxplot(alpha = 0.8, outlier.colour = NA, coef = 500) +

scale_y_log10() +

geom_point(aes(fill = Therapy), size = 5, shape = 21, position = position_jitterdodge()) +

theme(text = element_text(size = 18),

axis.title.x = element_blank(),

axis.title.y = element_blank(),

panel.grid.minor.x = element_blank(),

panel.grid.major.x = element_blank()) +

annotate('text', x = -Inf, y = Inf, hjust = -0.01, vjust = 1,

label = paste0('P-adjusted: ', padj)) +

annotate('text', x = -Inf, y = Inf, hjust = -0.01, vjust = 2.3,

label = paste0('L2FC: ', log2fold))

ggsave(boxplots, filename = paste0('graphs/raw_boxplots/', symbol, '_by_therapy_raw.png'),

height = 7, width = 7)

}

}

plot_sva_boxplots(ddsMat1)

### Draw SVA weighted/unweighted model comparison barplots for significant DEG`s

plot_mod_diff = function (no, yes) {

m1 = estimateSizeFactors(no)

m2 = estimateSizeFactors(yes)

m1_counts = as.data.frame(counts(m1, normalized = TRUE))

m2_counts = as.data.frame(counts(m2, normalized = TRUE))

#print(head(m1_counts, 3))

#print(head(m2_counts, 3))

for (gene in res1OrderedSignif2$Genes) {

m1_t = as.data.frame(t(m1_counts[gene,]))

m2_t = as.data.frame(t(m2_counts[gene,]))

m1_t$Sample = rownames(m1_t)

m2_t$Sample = rownames(m2_t)

names(m1_t)[1] = 'Count'

names(m2_t)[1] = 'Count'

m1_t$model = 'mod0'

m2_t$model = 'mod1'

m1_m2 = as.data.frame(rbind(m1_t, m2_t))

plots = ggplot(m1_m2, aes(x=Sample, y=Count, fill=model)) +

geom_bar(stat='identity', position='dodge')

ggsave(plots, filename = paste0('graphs/publication_graphs/model_barplots/', gene, '_test.png'))

}

}

plot_mod_diff(ddsMat, ddssva)

### Create black and white boxplots for the seven most differentially

expressed genes based on their L2FC values

list_of_genes = c('ENSG00000183742', 'ENSG00000083307', 'ENSG00000181143', 'ENSG00000181234', 'ENSG00000154997', 'ENSG00000186732', 'ENSG00000105088')

res1OrderedSignif3 = res1OrderedSignif2[which(rownames(res1OrderedSignif2) %in% list_of_genes), ]

plot_sva_boxplots_top = function (data) {

m <- estimateSizeFactors(data)

sva_counts = as.data.frame(counts(m, normalized=TRUE))

sva_counts = sva_counts[which(rownames(sva_counts) %in% list_of_genes), ]

p = list()

for (gene in res1OrderedSignif3$Genes) {

df = as.data.frame(t(sva_counts[gene, ]))

padj = formatC(res1OrderedSignif3[gene, 5], format = 'e', digits = 2)

log2fold = round(res1OrderedSignif3[gene, 2], 2)

symbol = res1OrderedSignif3[gene, 7]

names(df)[1] = 'Count'

df$Therapy = Therapy

df$Label = rownames(df)

df[, 1] = df[, 1] + 0.5

print(res1OrderedSignif2[gene, 7])

p[[gene]] = ggplot(df, aes(x = Therapy, y = Count, group = Therapy)) +

geom_boxplot(alpha = 0.8, outlier.colour = NA, coef = 500) +

scale_y_log10() +

geom_point(aes(color = Therapy), size = 5, shape = 16, position = position_jitterdodge(),

alpha = 0.5) +

scale_colour_manual(values=rep("black",length(Therapy))) +

#geom_point(aes(fill = Therapy, color = '#000000'), size = 5, shape = 21,

# position = position_jitterdodge(), color = '#000000') +

labs(title = symbol) +

theme_bw() +

theme(text = element_text(size = 18),

axis.title.x = element_blank(),

axis.title.y = element_blank(),

panel.grid.minor.x = element_blank(),

panel.grid.major.x = element_blank(),

panel.grid.major.y = element_blank(),

panel.grid.minor.y = element_blank(),

legend.position = 'none',

plot.title = element_text(size = 15)) +

annotate('text', x = -Inf, y = Inf, hjust = -0.01, vjust = 1,

label = paste0('P-adjusted: ', padj)) +

annotate('text', x = -Inf, y = Inf, hjust = -0.01, vjust = 2.3,

label = paste0('L2FC: ', log2fold))

}

tiff('graphs/publication_graphs/bw_boxplots/combined_bw_boxplots_lzw.tif',

units = 'in', w = 15, h = 15, compression = 'lzw', res = 1200)

do.call(grid.arrange, p)

dev.off()

}

plot_sva_boxplots_top(ddsMat1)

### Calculate Kendall`s correlation between Ki-67/Knosp index and read counts for the significant DEG`s

knosp_index = c(2, 1, 4, 1, 0, 4, 1, 1, 4, 2, 3, 1)

ki_index = c(1, 2, 1, 4, 4, 1, 3, 1, 2, 3, 1, 2)

index_correlation = function(x, y, z) {

for (i in rownames(x)) {

gene = z[i, ][, 7]

counts = as.numeric(x[i, ])

factor = c(y)

data_frame = data.frame(read_counts = counts, knosp_grade = factor, Therapy = Therapy)

frame_up = as.data.frame(data_frame[1:6, ])

frame_down = as.data.frame(data_frame[7:12, ])

corr_yes = cor.test(frame_up$knosp_grade, frame_up$read_counts, method = 'kendall')

corr_no = cor.test(frame_down$knosp_grade, frame_down$read_counts, method = 'kendall')

coef_yes = round(as.numeric(corr_yes$estimate), 2)

pval_yes = formatC(as.numeric(corr_yes$p.value), format = 'e', digits = 2)

coef_no = round(as.numeric(corr_no$estimate), 2)

pval_no = formatC(as.numeric(corr_no$p.value), format = 'e', digits = 2)

Therapy = as.factor(Therapy)

plots = ggplot(data_frame, aes(x = knosp_grade, y = read_counts, color = Therapy,

shape = Therapy)) +

scale_color_manual(values = c('red', 'blue')) +

geom_point(size = 4) +

geom_smooth(aes(group = Therapy, color = Therapy, fill = Therapy), method = lm, linetype = 'dashed', fullrange = T) +

guides(

shape = guide_legend(

override.aes = list(color = rep(c('red', 'blue'), each = 1))), color = F) +

annotate(geom = 'text', x = -Inf, y = Inf, hjust = -0.01, vjust = 1,

label = paste0('Gene: ', gene)) +

annotate(geom = 'text', x = -Inf, y = Inf, hjust = -0.01, vjust = 2.2,

label = paste0('Tau Yes: ', coef_yes)) +

annotate(geom = 'text', x = -Inf, y = Inf, hjust = -0.01, vjust = 3.6,

label = paste0('Pval - Yes: ', pval_yes)) +

annotate(geom = 'text', x = -Inf, y = Inf, hjust = -0.01, vjust = 55,

label = paste0('Tau - No: ', coef_no)) +

annotate(geom = 'text', x = -Inf, y = Inf, hjust = -0.01, vjust = 56.4,

label = paste0('Pval - No: ', pval_no)) +

xlab('Ki - 67 index group') + ylab('Read count')

ggsave(plots, filename = paste0('correlation_graphs_6_k_index/', gene,

'_corr_k_index.png'))

}

}

index_correlation(sva_counts_deg, ki_index, res1OrderedSignif2)

index_correlation(sva_counts_deg, knosp_index, res1OrderedSignif2)

Supplementary Figure 1. The staining of (A, B, C) prolactin and (D, E) growth hormone in formalin-fixed paraffin-embedded sections of the tumour of the patient PA11, in 200× (A, D) or 400× (B, C, E) magnification.


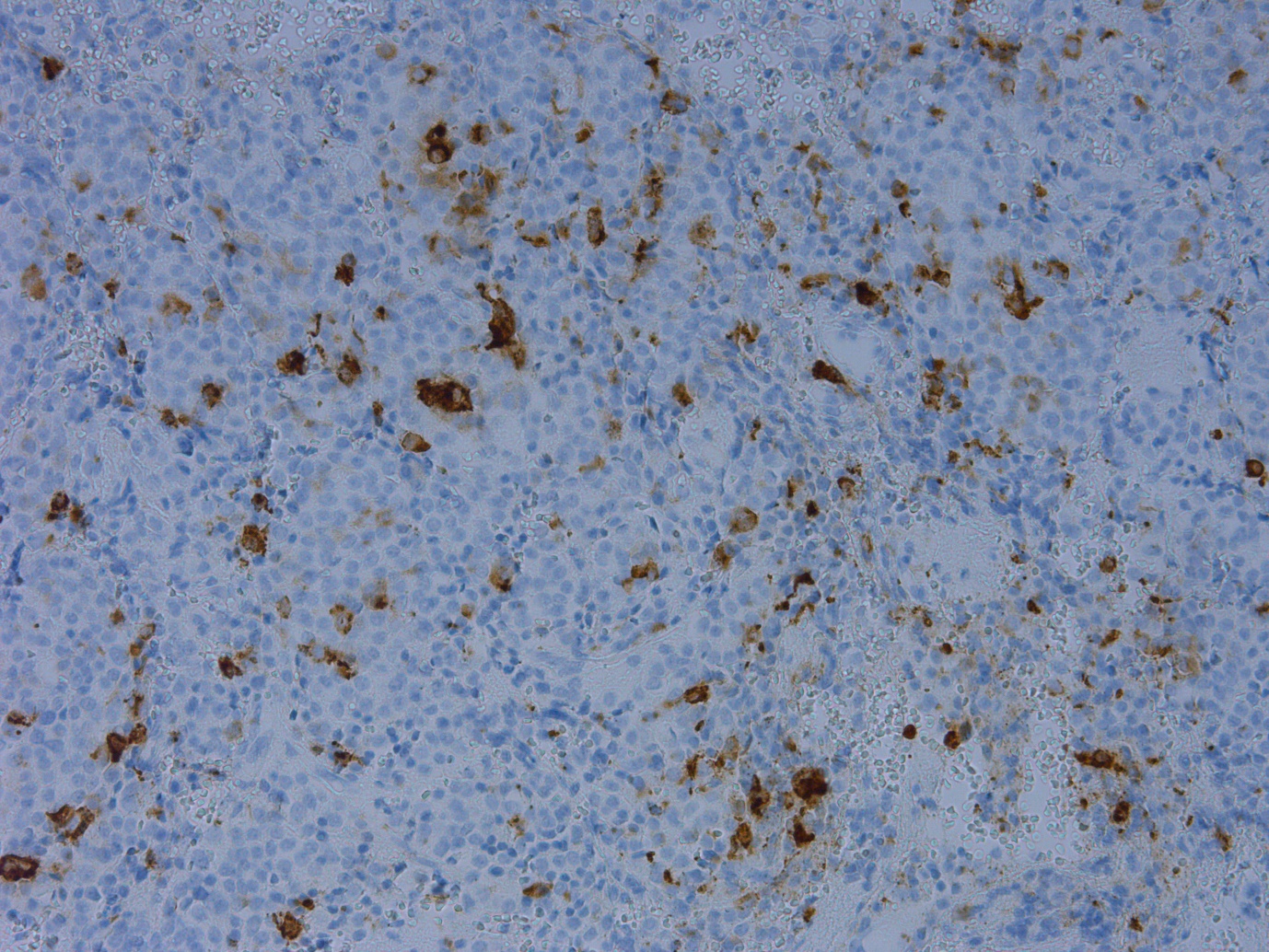
A.

Supplementary Figure 1. The staining of (A, B, C) prolactin and (D, E) growth hormone in formalin-fixed paraffin-embedded sections of the tumour of the patient PA11, in 200× (A, D) or 400× (B, C, E) magnification.

B.


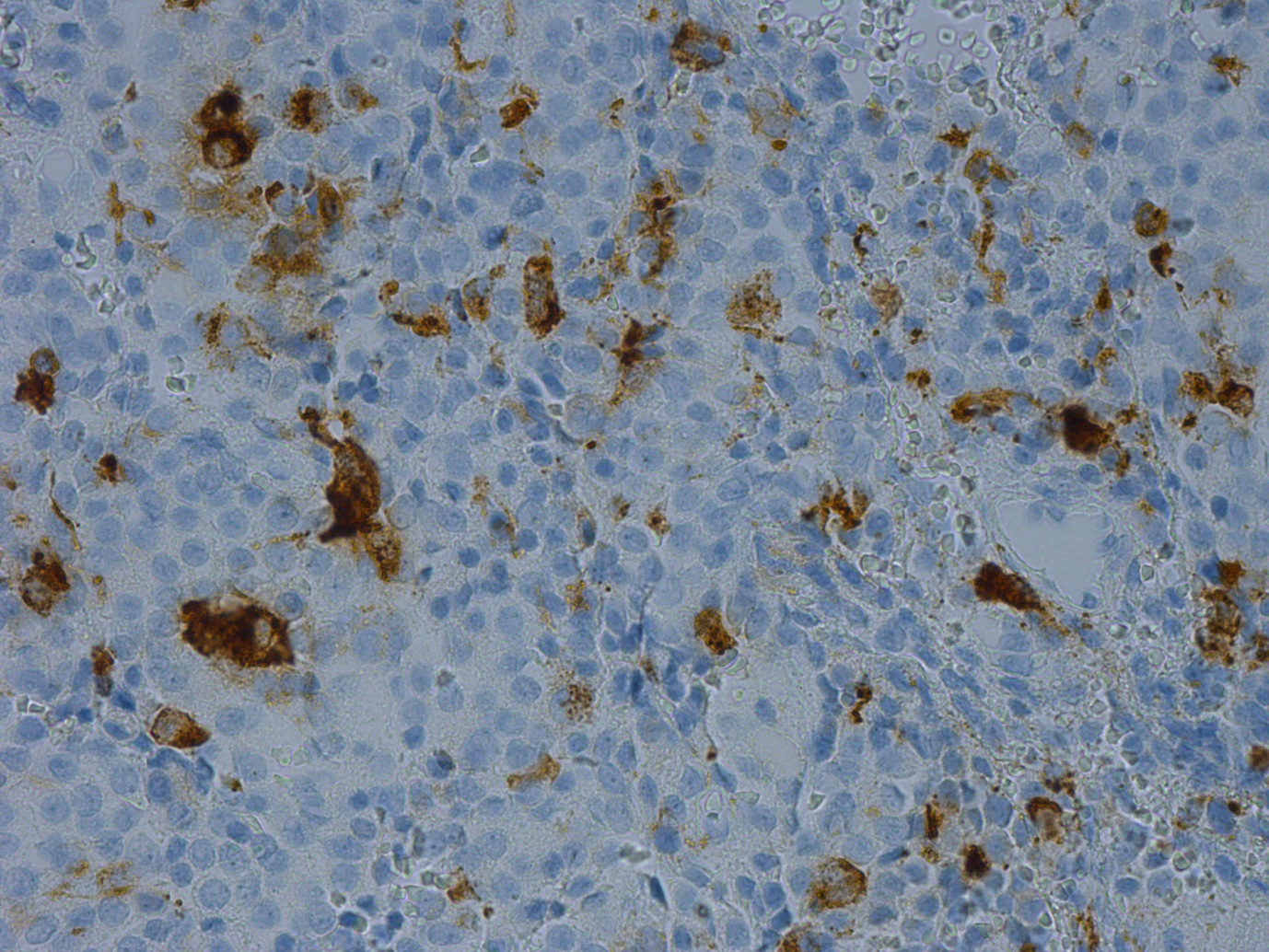


Supplementary Figure 1. The staining of (A, B, C) prolactin and (D, E) growth hormone in formalin-fixed paraffin-embedded sections of the tumour of the patient PA11, in 200× (A, D) or 400× (B, C, E) magnification.

C.


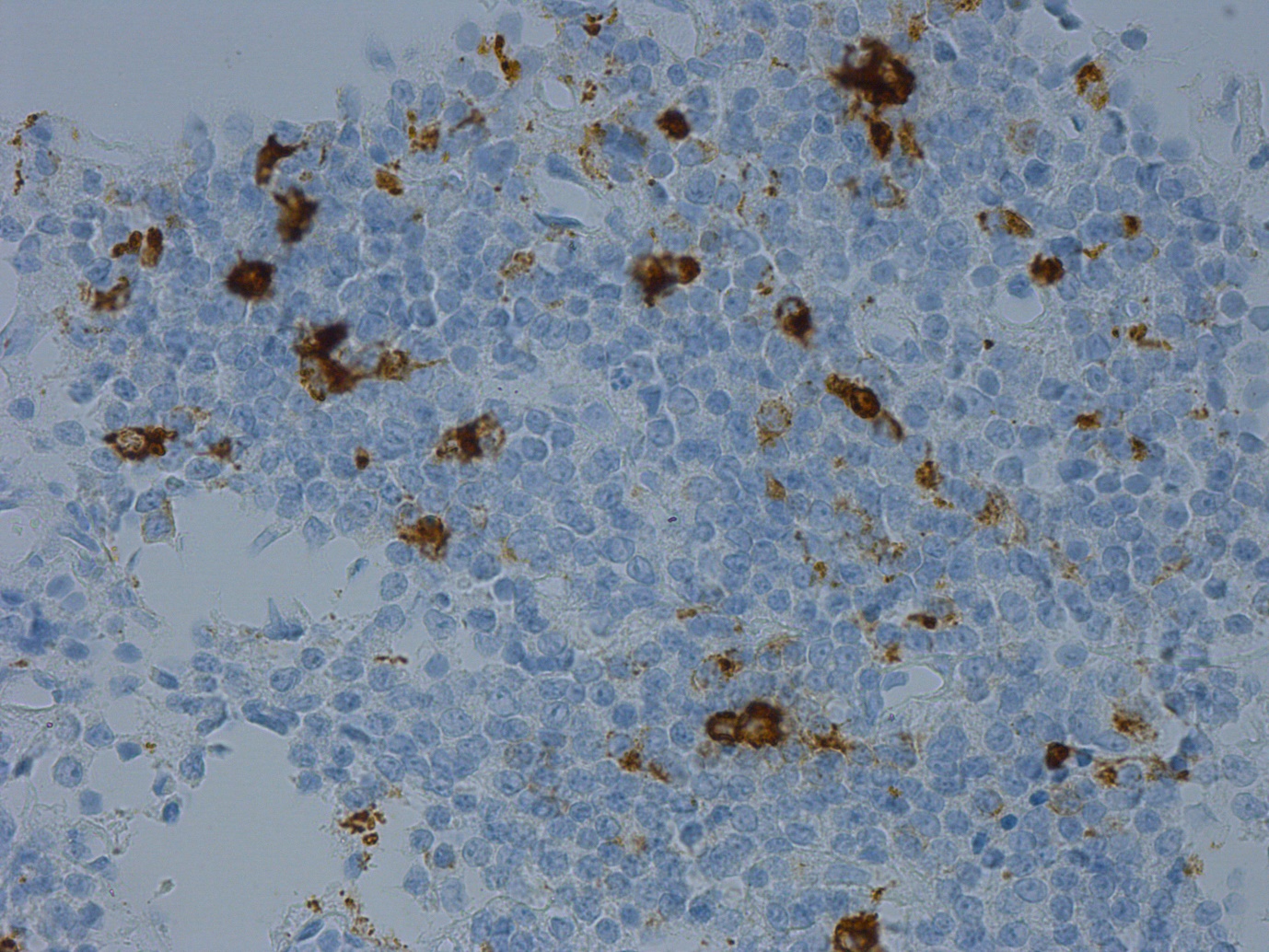


Supplementary Figure 1. The staining of (A, B, C) prolactin and (D, E) growth hormone in formalin-fixed paraffin-embedded sections of the tumour of the patient PA11, in 200× (A, D) or 400× (B, C, E) magnification.

D.


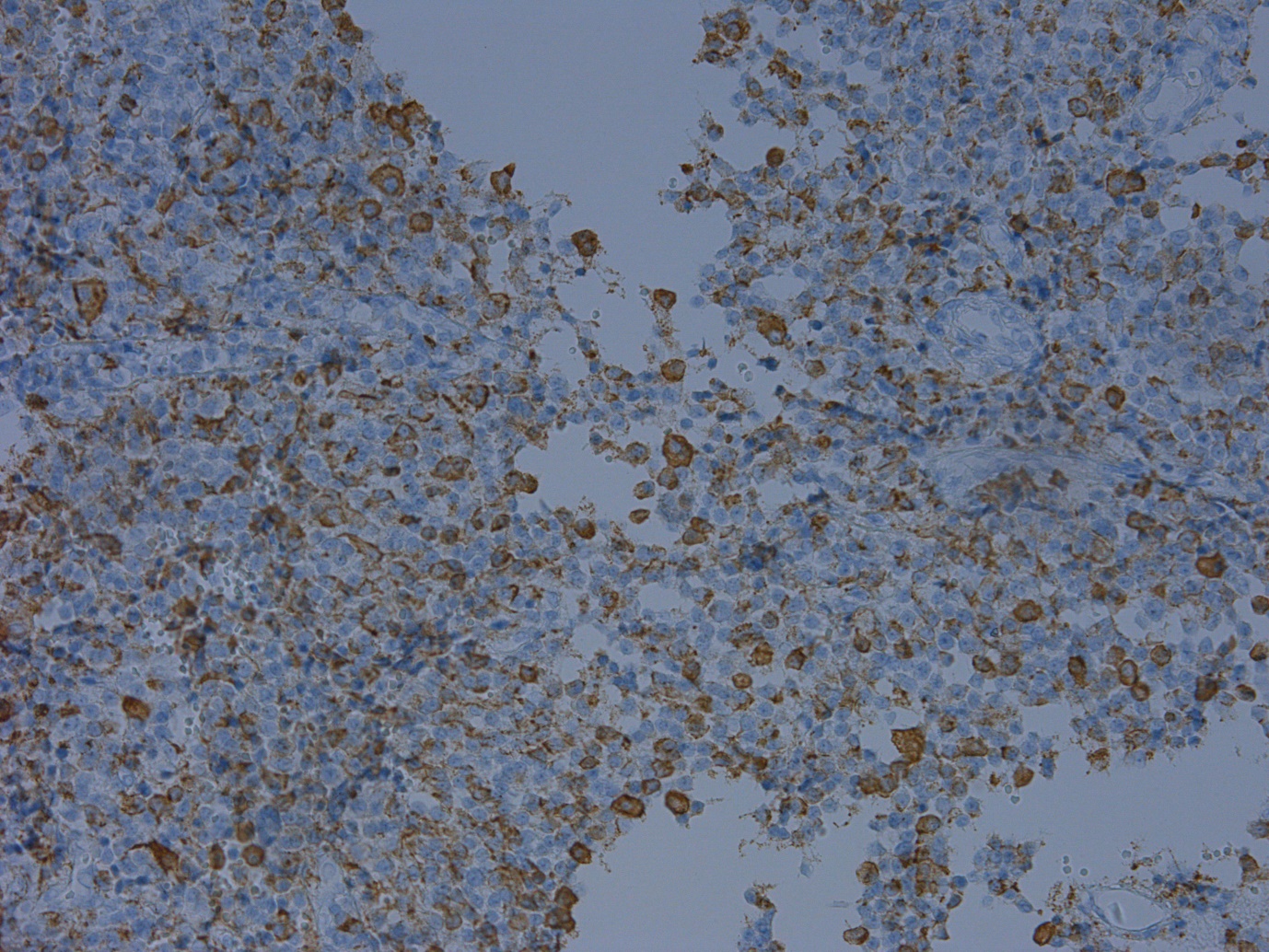


Supplementary Figure 1. The staining of (A, B, C) prolactin and (D, E) growth hormone in formalin-fixed paraffin-embedded sections of the tumour of the patient PA11, in 200× (A, D) or 400× (B, C, E) magnification.


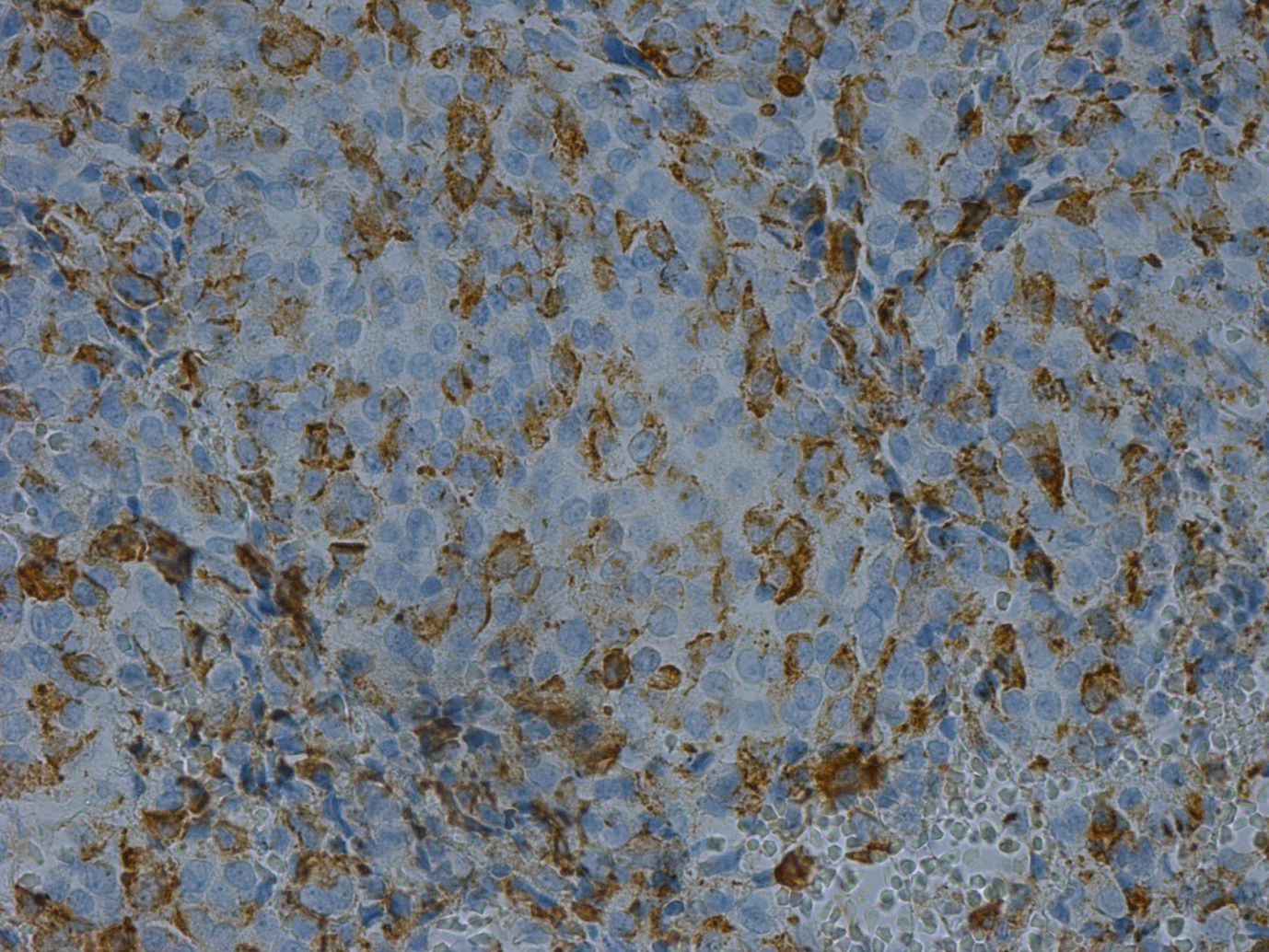
E.

**
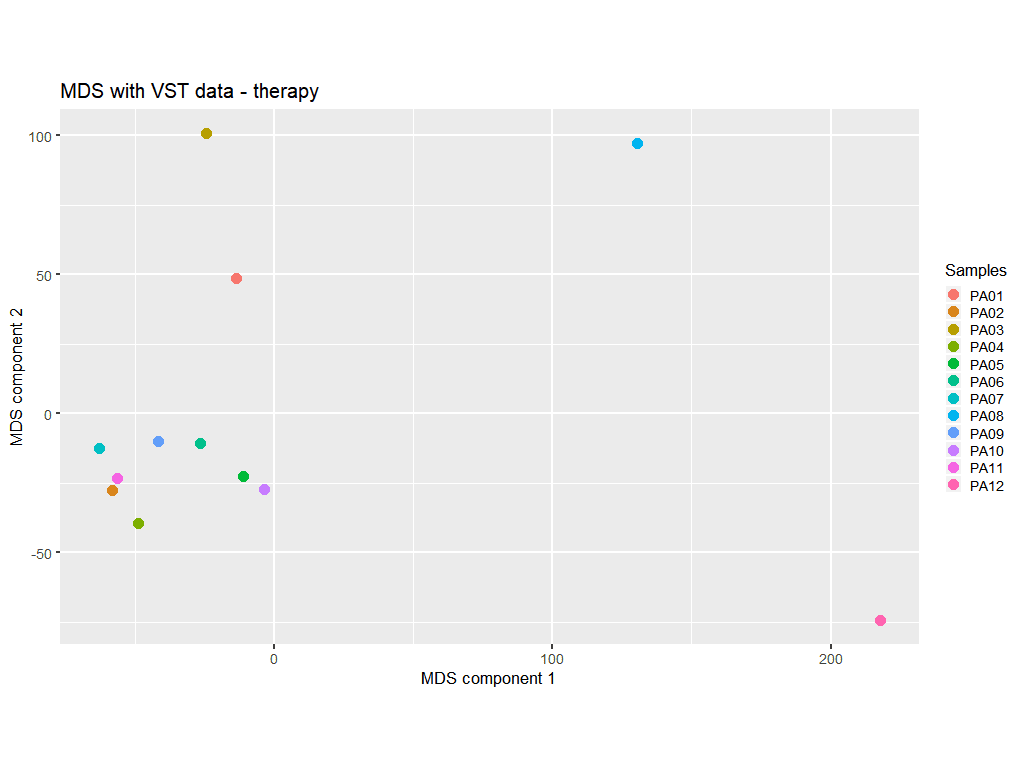
**

Supplementary Figure 2. MDS plot, representing the distances between samples attained by variance stabilizing transformation (VST), while considering the design of the experiment.

**
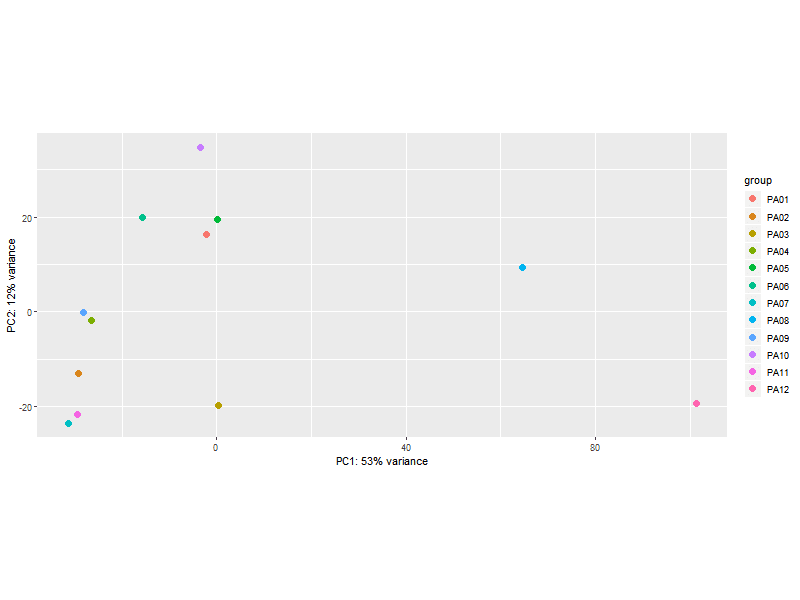
**

Supplementary Figure 3. PCA plot, representing the distances between samples, attained by variance stabilizing transformation (VST), for the 500 most variable genes, while considering the design of the experiment.


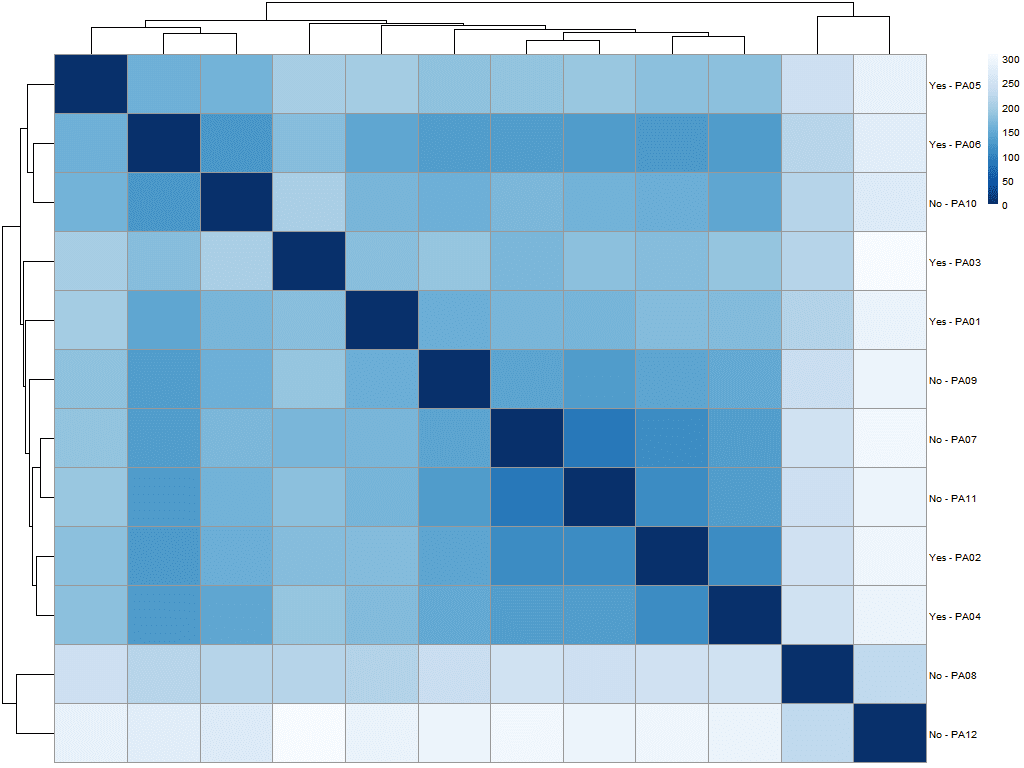


Supplementary Figure 4. Sample distance heatmap attained by variance stabilizing transformation (VST), while considering the design of the experiment.

Supplementary Table 1. Statistically significant differential expression results sorted by logFC values (FDR < 0.05, LFC > 1.5) after logFC value shrinkage with “apeglm” method.

| **Gene symbol** | **Gene name** | **logFC** | **lfcSE** | **P-adjusted** |
| --- | --- | --- | --- | --- |
| *OLFM2* | Olfactomedin 2 | 3.861992495 | 0.4063966529 | 2.34E-18 |
| *SLC6A1* | Solute Carrier Family 6 Member 1 | -1.812466232 | 0.3467972963 | 2.46E-05 |
| *PDE4A* | Phosphodiesterase 4A | 2.36590717 | 0.4439366807 | 2.46E-05 |
| *COL16A1* | Collagen Type XVI Alpha 1 Chain | 3.964580043 | 0.9217541217 | 0.0002327896974 |
| *TMEM132C* | Transmembrane Protein 132C | 6.109170512 | 1.32571399 | 0.0003255397239 |
| *QPRT* | Quinolinate Phosphoribosyltransferase | 2.569262955 | 0.574450348 | 0.0003255397239 |
| *ATP1B2* | ATPase Na+/K+ Transporting Subunit Beta 2 | 2.886842721 | 0.6621209123 | 0.0005443237691 |
| *SEPTIN14* | Septin 14 | 5.134807694 | 1.242063898 | 0.0007138682988 |
| *BCAM* | Basal Cell Adhesion Molecule (Lutheran Blood Group) | 2.030611835 | 0.467608193 | 0.0007138682988 |
| *COL6A1* | Collagen Type VI Alpha 1 Chain | 2.05643287 | 0.4727030263 | 0.0007138682988 |
| *MACC1* | MET Transcriptional Regulator MACC1 | -2.181777812 | 0.5126578126 | 0.001019007771 |
| *CILP* | Cartilage Intermediate Layer Protein | 4.469986799 | 1.055636603 | 0.001120757708 |
| *ARC* | Activity Regulated Cytoskeleton Associated Protein | 3.763610637 | 1.317855846 | 0.001695034186 |
| *STUM* | Stum, Mechanosensory Transduction Mediator Homolog | 3.091475666 | 0.8434317222 | 0.002382015373 |
| *NPTX1* | Neuronal Pentraxin 1 | 3.567108865 | 0.9417904296 | 0.002382015373 |
| *CLU* | Clusterin | 1.811606574 | 0.5293514384 | 0.005520102777 |
| *MPPED1* | Metallophosphoesterase Domain Containing 1 | 6.236890011 | 1.8984953 | 0.005810564833 |
| *COL8A2* | Collagen Type VIII Alpha 2 Chain | 3.264420542 | 1.022101646 | 0.006380542227 |
| *GRHL2* | Grainyhead Like Transcription Factor 2 | -4.017230199 | 1.126700714 | 0.007754540601 |
| *SSC5D* | Scavenger Receptor Cysteine Rich Family Member With 5 Domains | 2.327782704 | 0.6810738418 | 0.008112312279 |
| *GRIK3* | Glutamate Ionotropic Receptor Kainate Type Subunit 3 | 4.008811693 | 1.343351586 | 0.009553464439 |
| *ADAMTSL2* | ADAMTS Like 2 | 1.929330369 | 0.5946071698 | 0.01144677113 |
| *PDE6A* | Phosphodiesterase 6A | 3.849065135 | 1.481947861 | 0.01842002315 |
| *SLC8A2* | Solute Carrier Family 8 Member A2 | 4.31868302 | 1.639976348 | 0.01880507978 |
| *PCP4L1* | Purkinje Cell Protein 4 Like 1 | 4.553756447 | 1.875125251 | 0.02199574097 |
| *DIRAS2* | DIRAS Family GTPase 2 | 3.908240681 | 1.567411038 | 0.02199574097 |
| *B4GALNT4* | Beta-1,4-N-Acetyl-Galactosaminyltransferase 4 | 2.119435095 | 0.7344768223 | 0.02199574097 |
| *APBA2* | Amyloid Beta Precursor Protein Binding Family A Member 2 | 2.826636724 | 0.9856762967 | 0.02199574097 |
| *THBD* | Thrombomodulin | -1.633266162 | 0.5589933792 | 0.02199574097 |
| *PTGS2* | Prostaglandin-Endoperoxide Synthase 2 | -1.75238353 | 0.5748906512 | 0.02246772161 |
| *SV2B* | Synaptic Vesicle Glycoprotein 2B | 2.922091878 | 0.9922489463 | 0.02318657143 |
| *LINC01529* | Long Intergenic Non-Protein Coding RNA 1529 | 2.947785061 | 1.038510215 | 0.02318657143 |
| *ST6GALNAC5* | ST6 N-Acetylgalactosaminide Alpha-2,6-Sialyltransferase 5 | 1.547760437 | 0.5716935941 | 0.03218321574 |
| *CALB2* | Calbindin 2 | 3.115647682 | 1.309175191 | 0.03392320221 |
| *MUC16* | Mucin 16, Cell Surface Associated | -2.485472247 | 0.8393167033 | 0.03392320221 |
| *TMEM184A* | Transmembrane Protein 184A | 1.654024699 | 0.613803635 | 0.03525008905 |
| *AHNAK2* | AHNAK Nucleoprotein 2 | 3.384162547 | 1.156057088 | 0.04073123451 |
| *ADAMTS10* | ADAM Metallopeptidase With Thrombospondin Type 1 Motif 10 | 2.441498092 | 0.9978281413 | 0.04073123451 |
| *CPNE7* | Copine 7 | 1.940151923 | 0.723539291 | 0.04210495127 |
| *DTNA* | Dystrobrevin Alpha | 1.527279584 | 0.6082263941 | 0.04776935295 |

Supplementary Table 2. Box plot diagrams displaying raw read count distribution and surrogate variable adjusted read (SVA) count distribution on the log10 scale for all 40 statistically significant DEGs. Bar plot diagrams displaying sample read counts in regard to their “DESeq2” model, where mod0 - raw read counts and mod1 - read counts where “SVA” covariates used as weights.

| **Gene symbol** | **Gene name** | **Raw read count distribution** | **SVA weighted/unweighted model comparison** |
| --- | --- | --- | --- |
| *OLFM2* | Olfactomedin 2 | 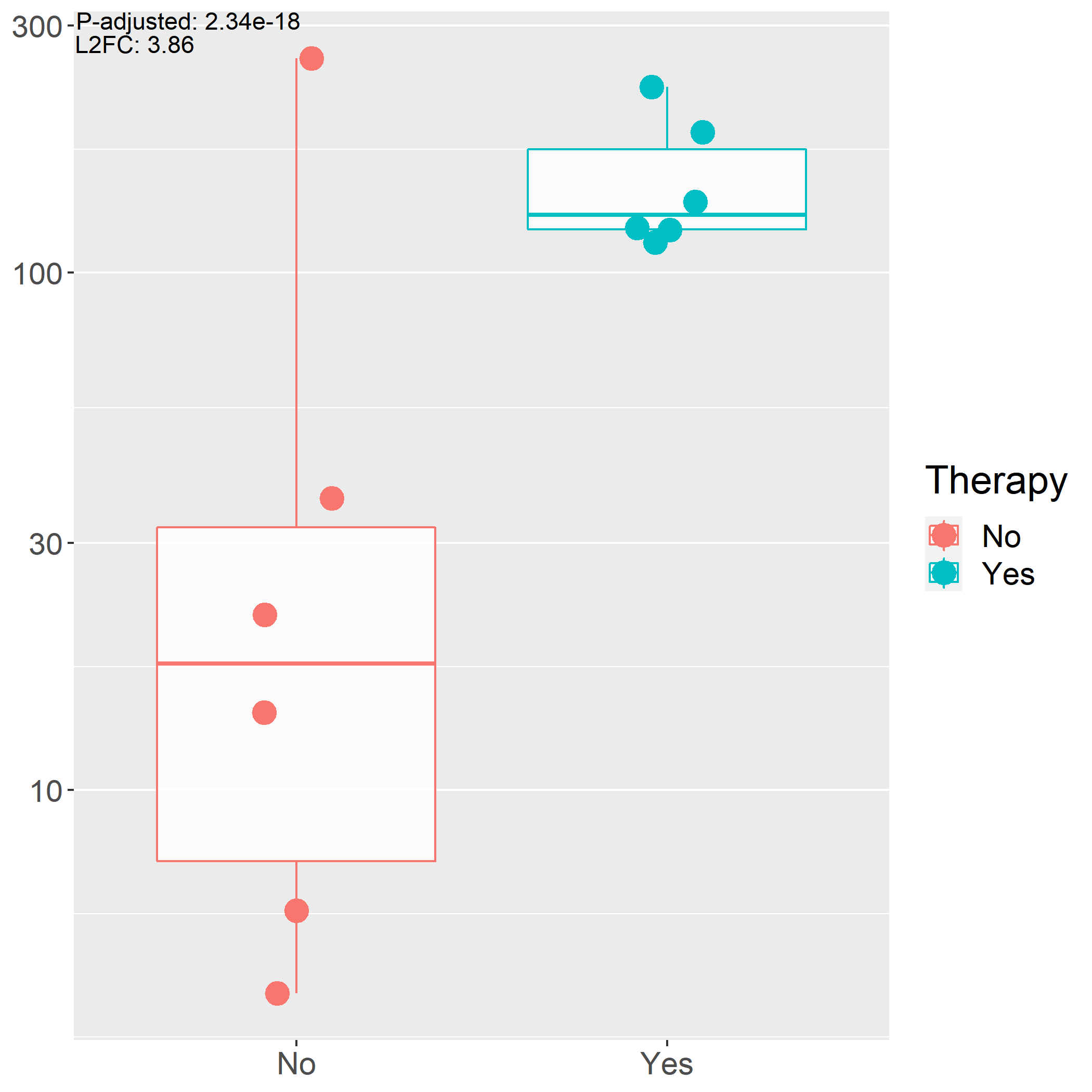 | 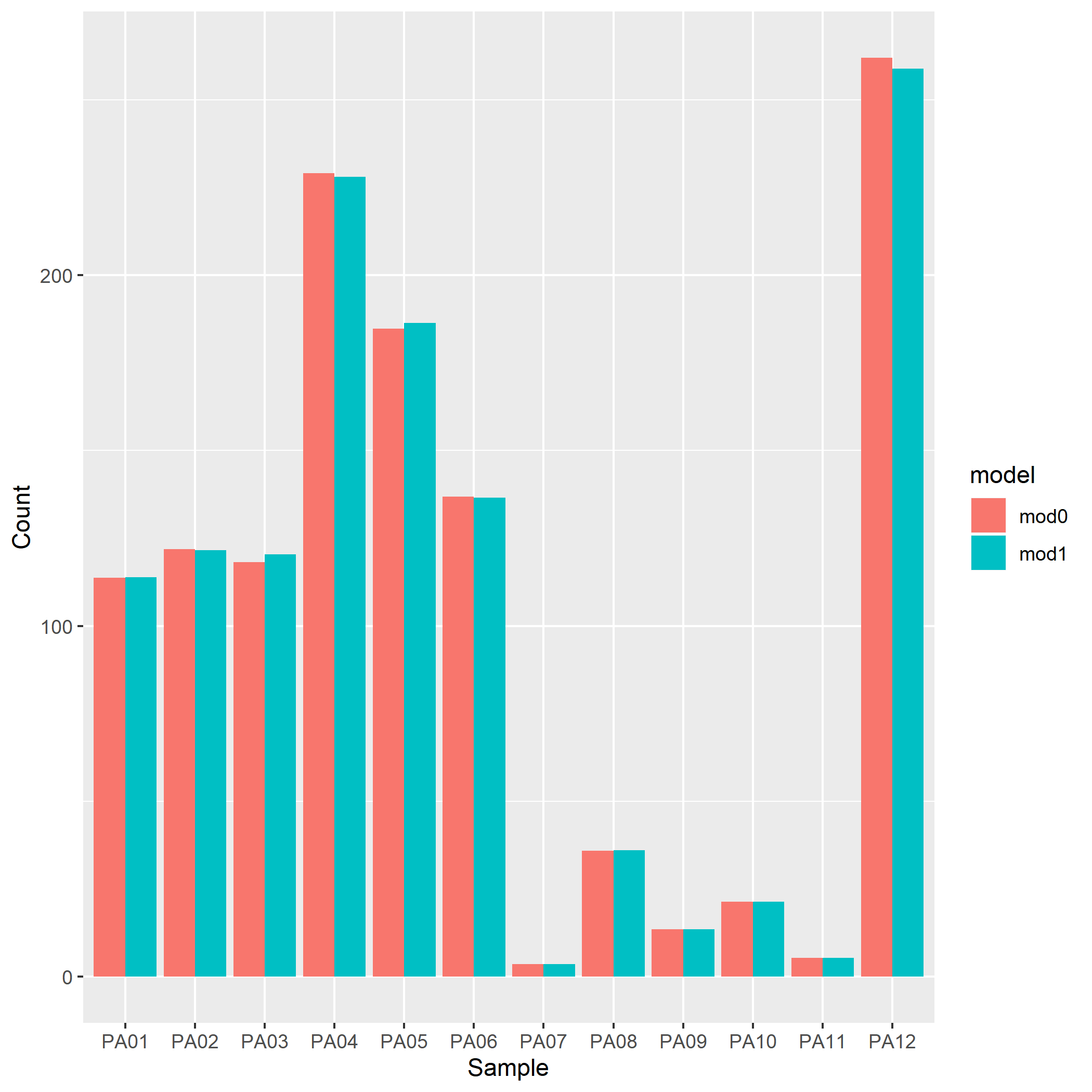 |
| *SLC6A1* | Solute Carrier Family 6 Member 1 | 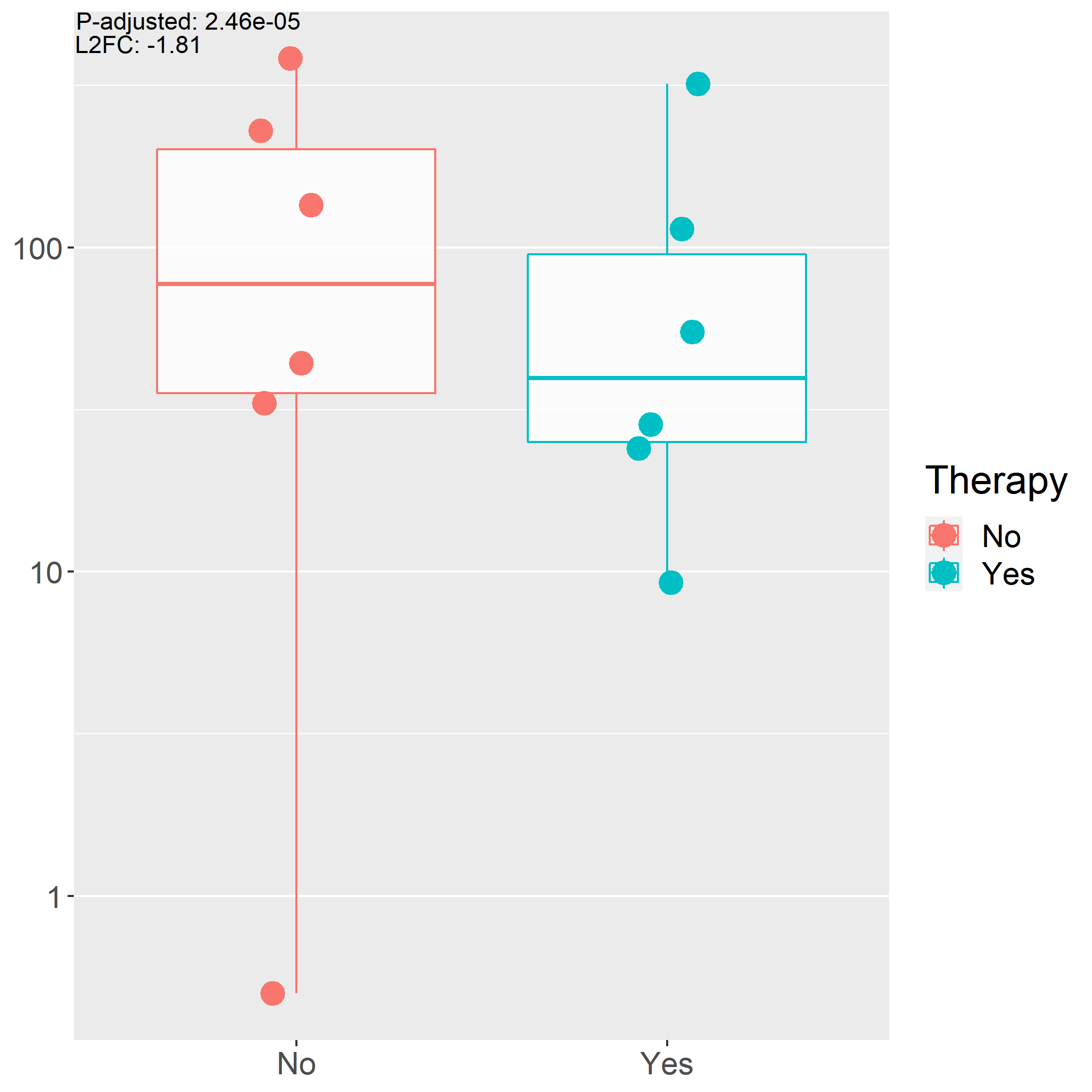 | 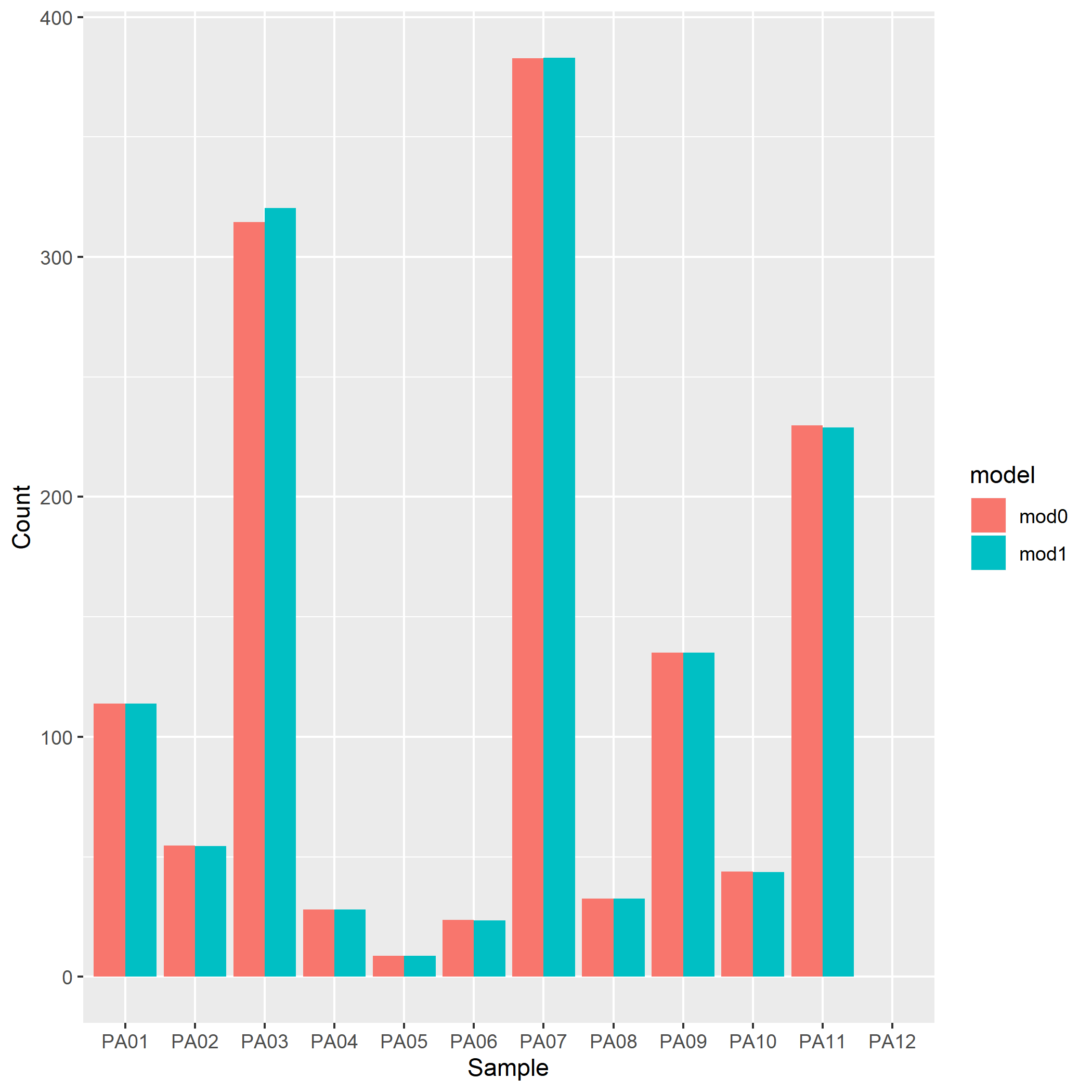 |
| *PDE4A* | Phosphodiesterase 4A | 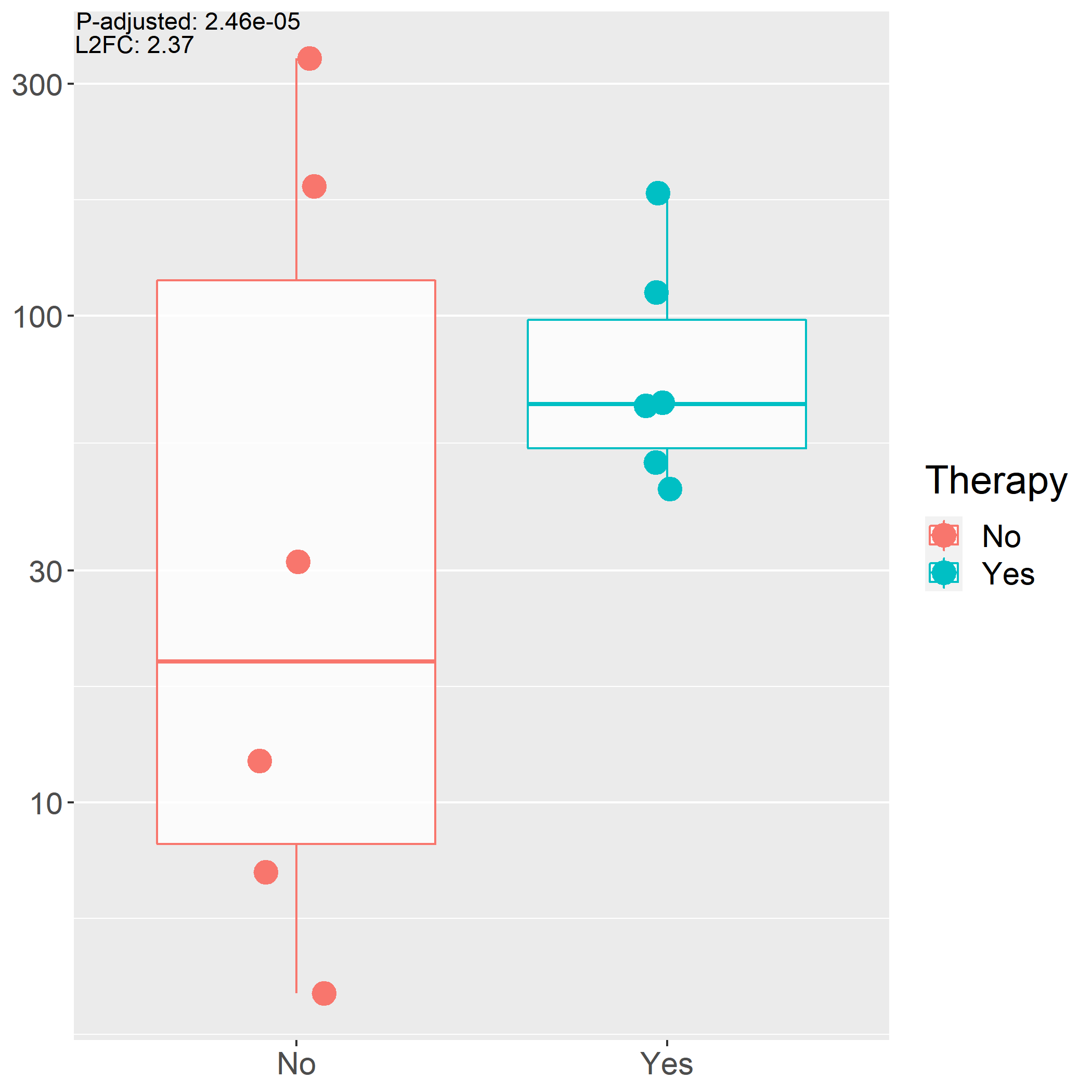 | 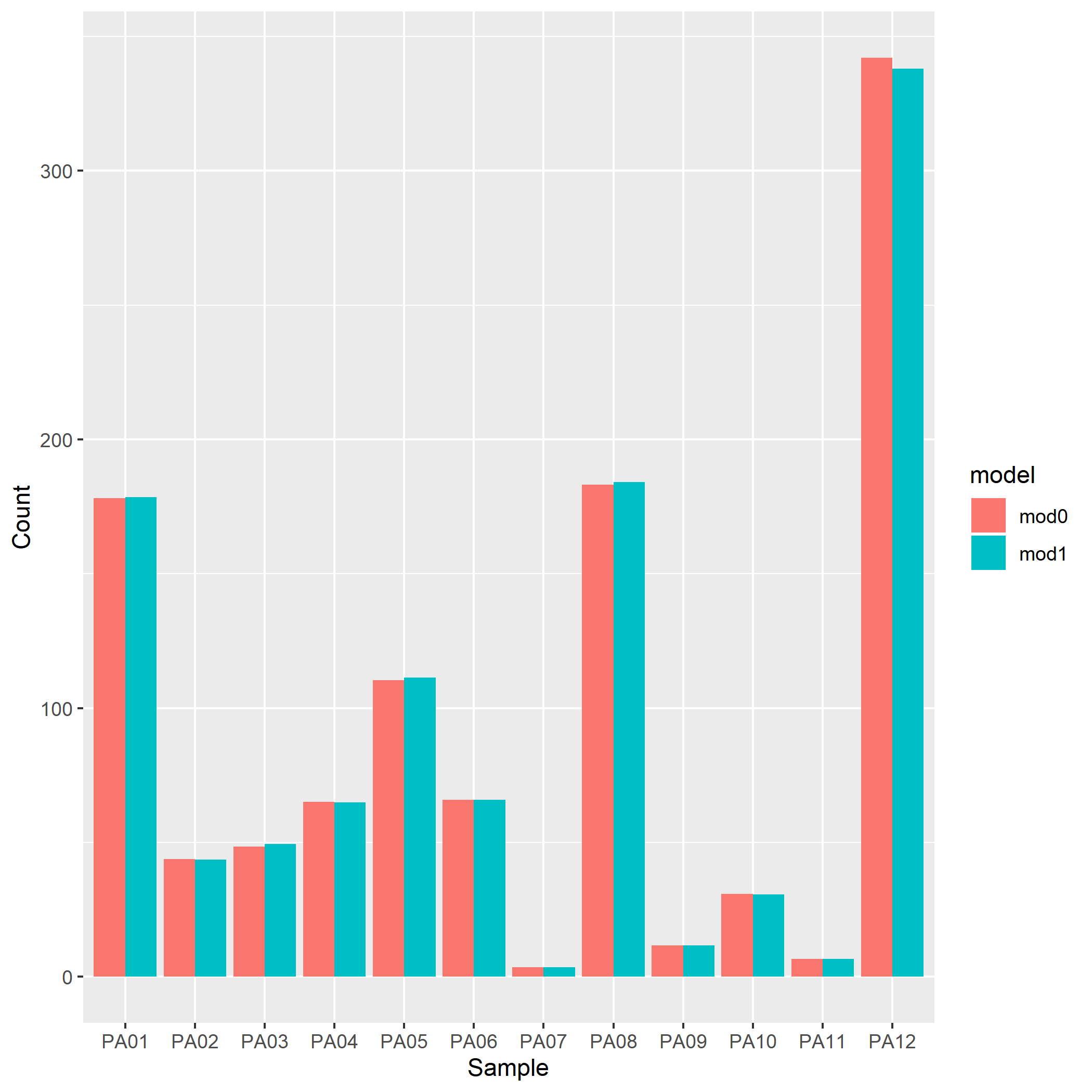 |
| *COL16A1* | Collagen Type XVI Alpha 1 Chain | 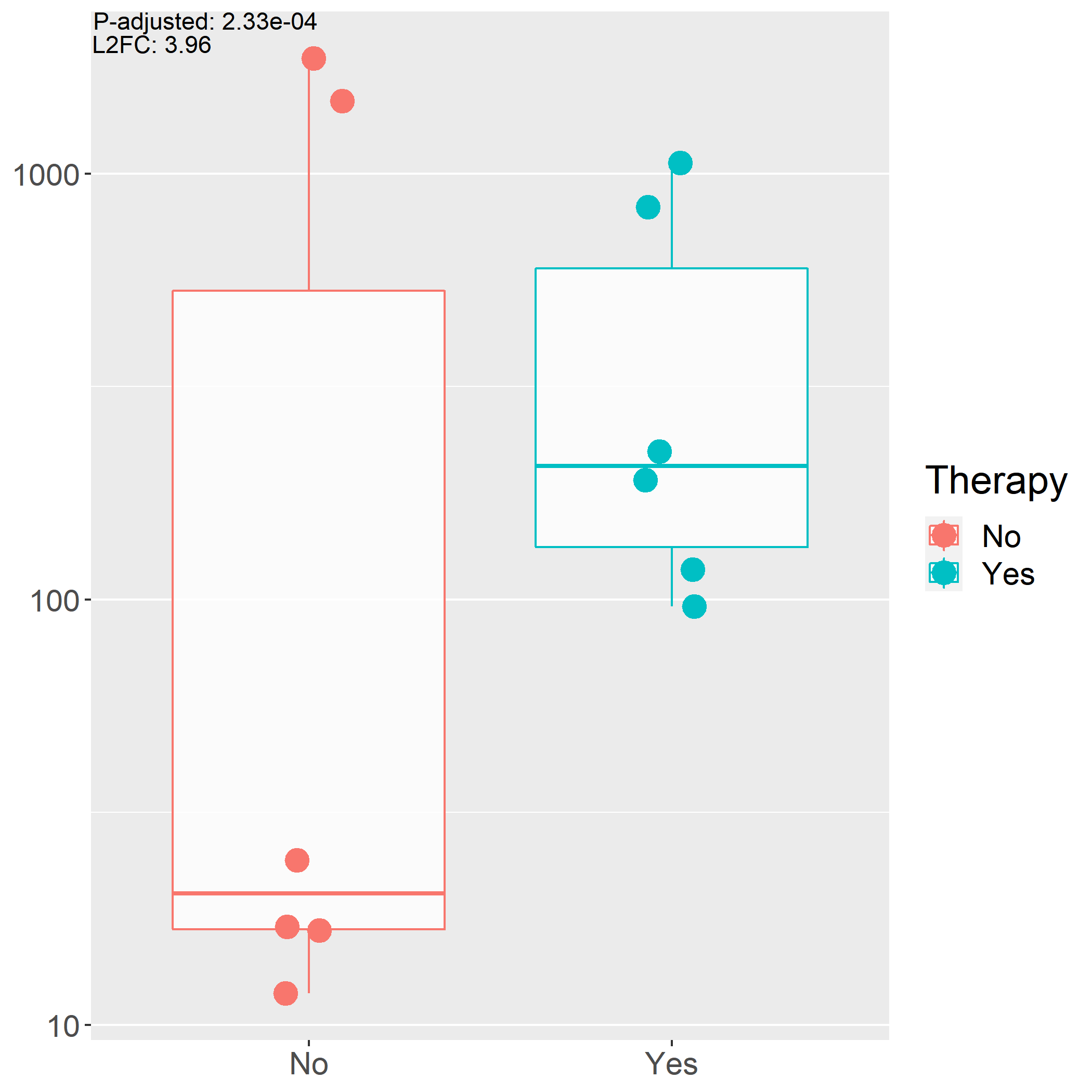 | 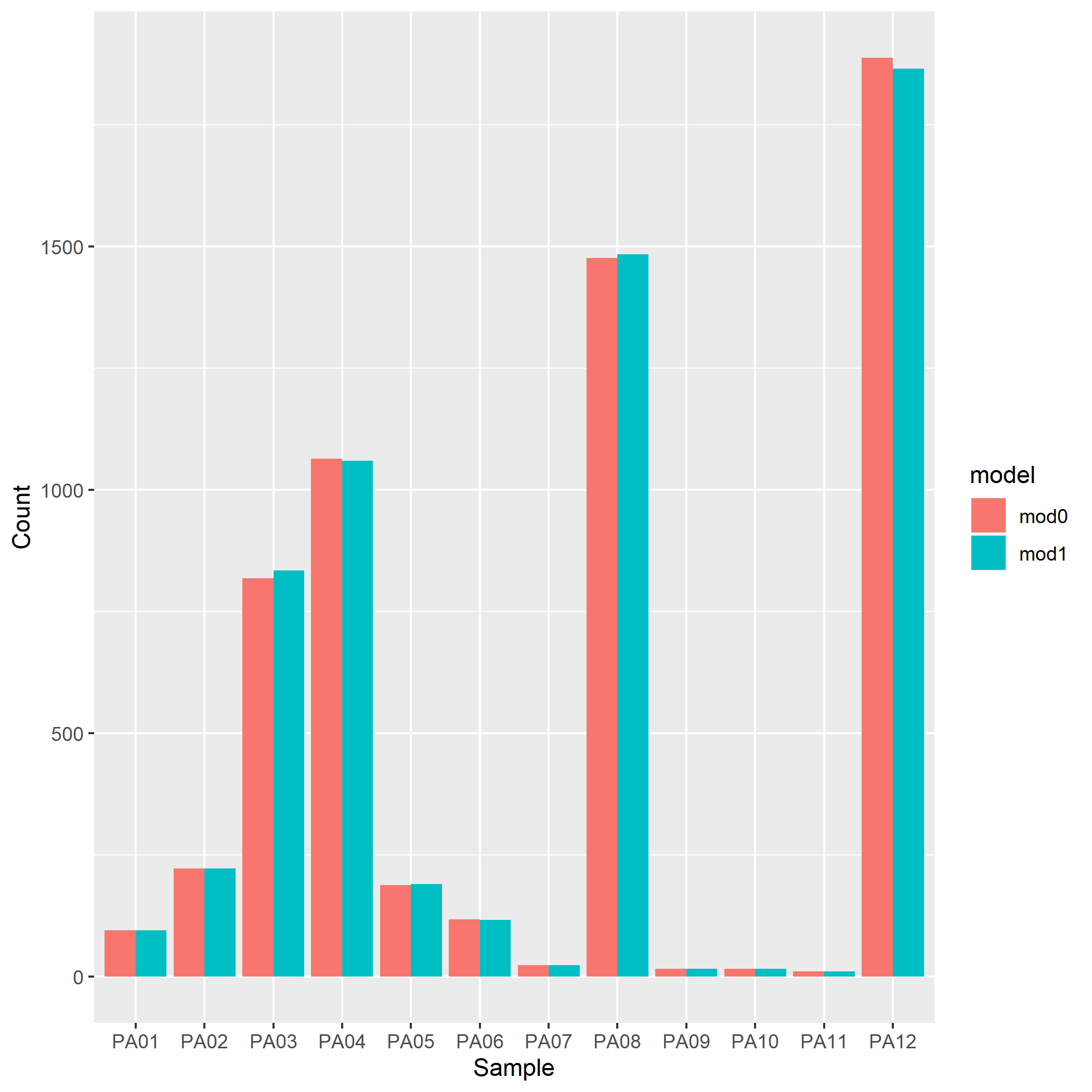 |
| *TMEM132C* | Transmembrane Protein 132C | 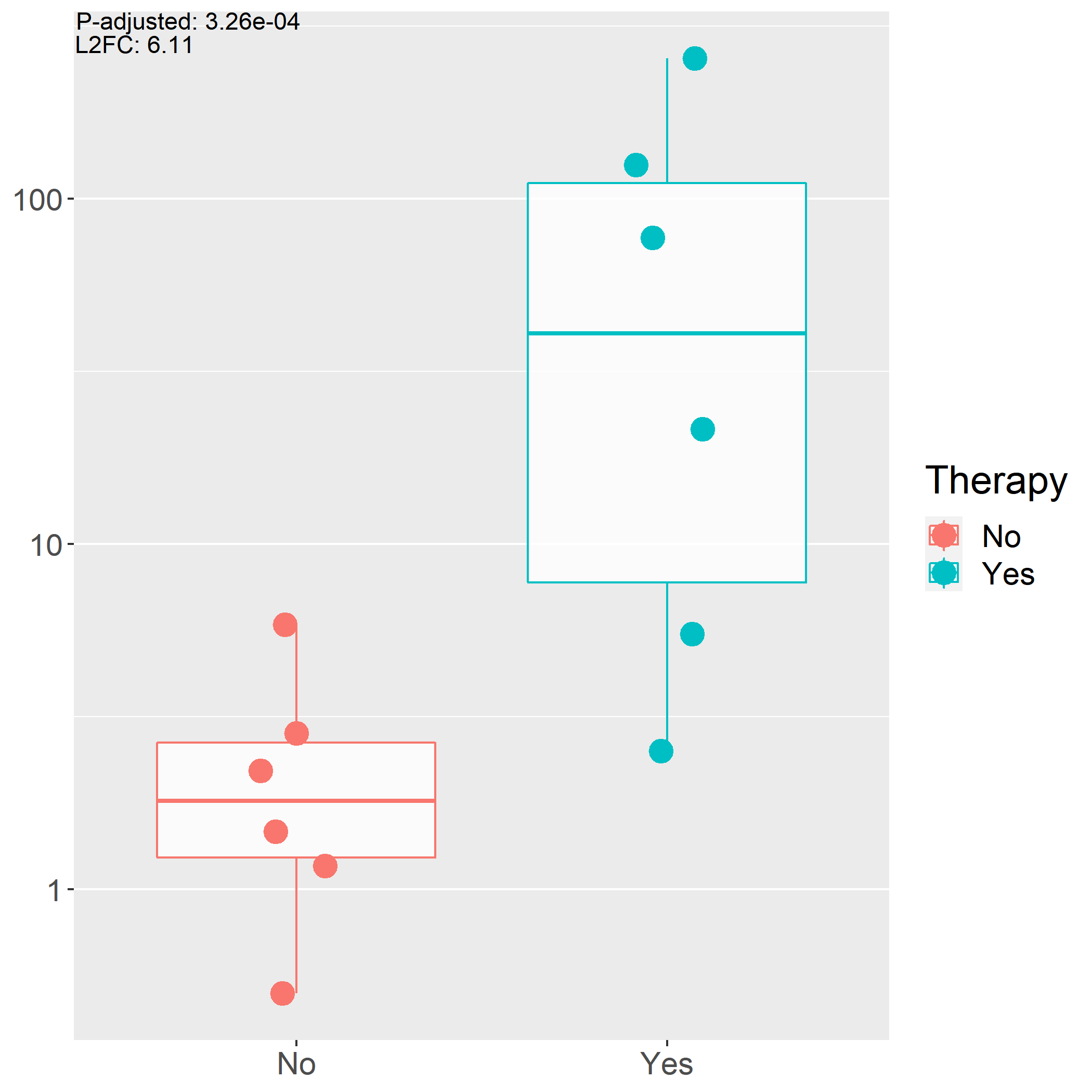 | 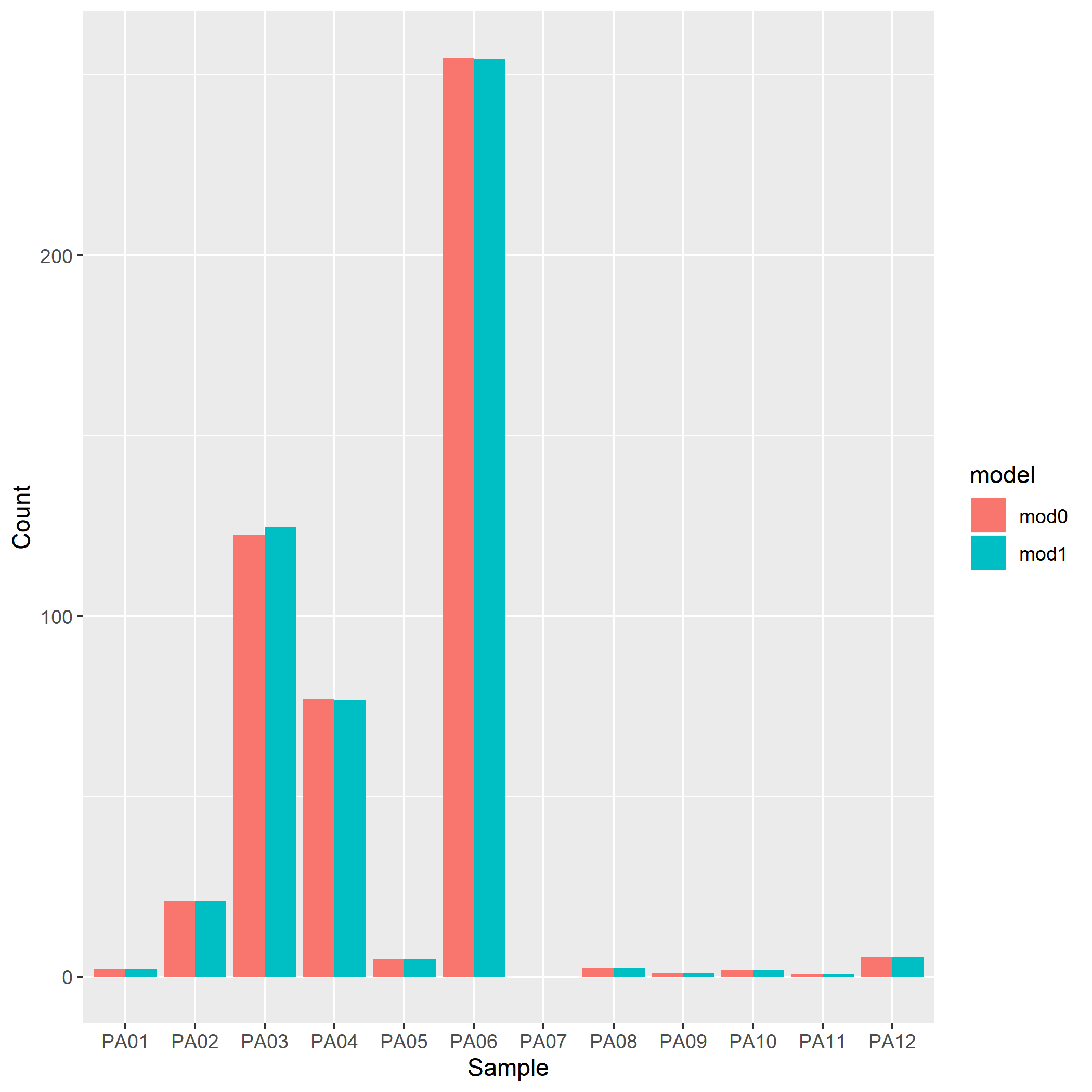 |
| *QPRT* | Quinolinate Phosphoribosyltransferase | 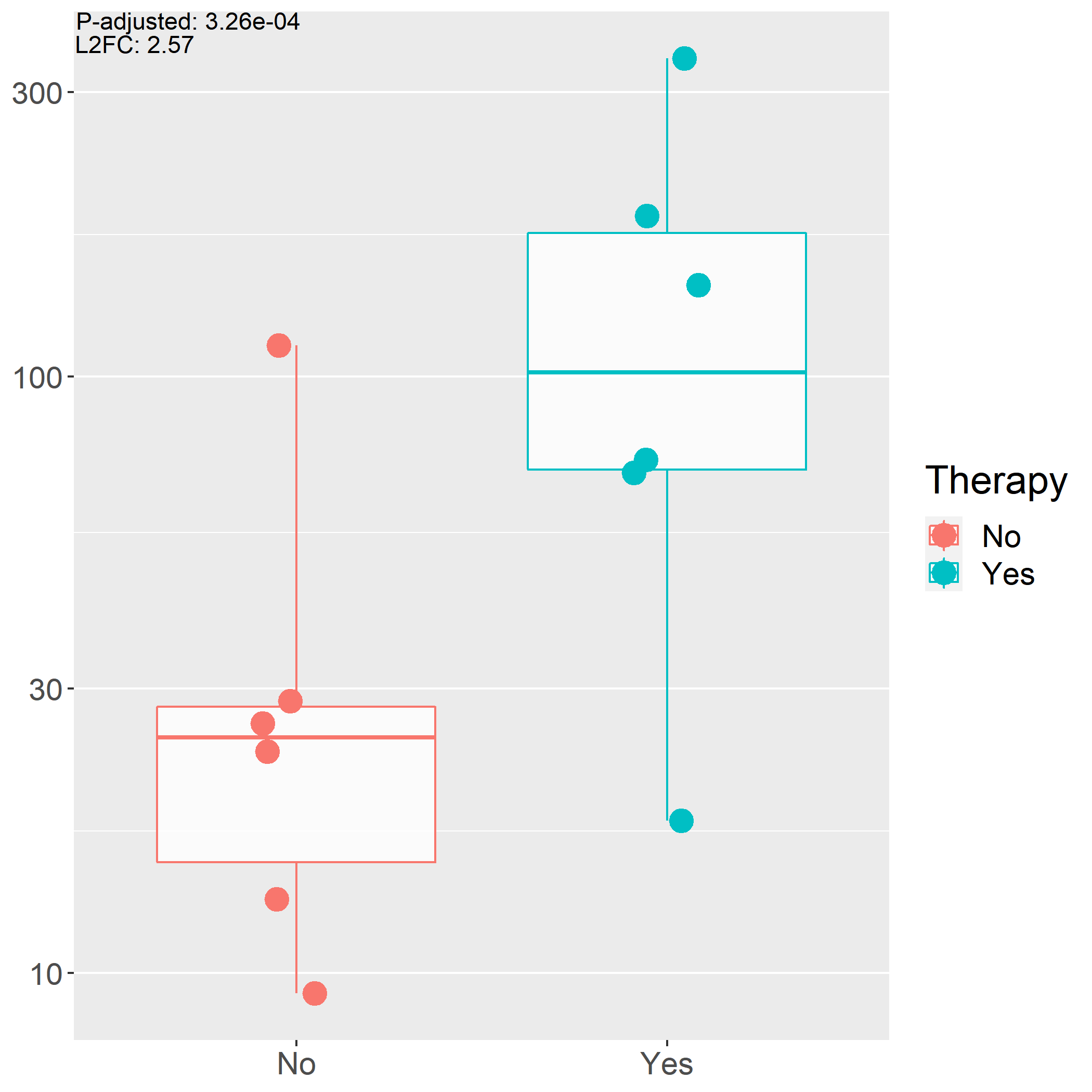 | 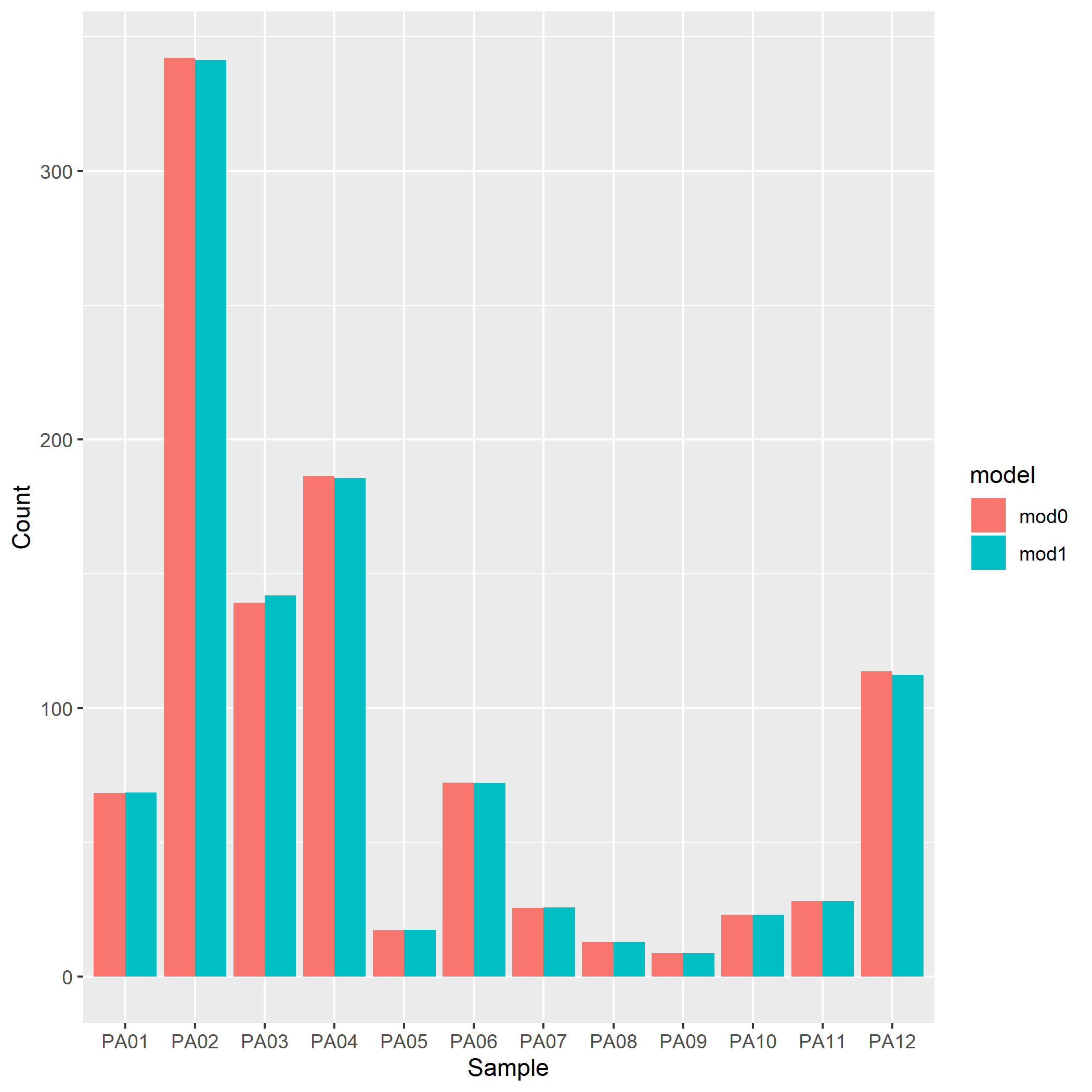 |
| *ATP1B2* | ATPase Na+/K+ Transporting Subunit Beta 2 | 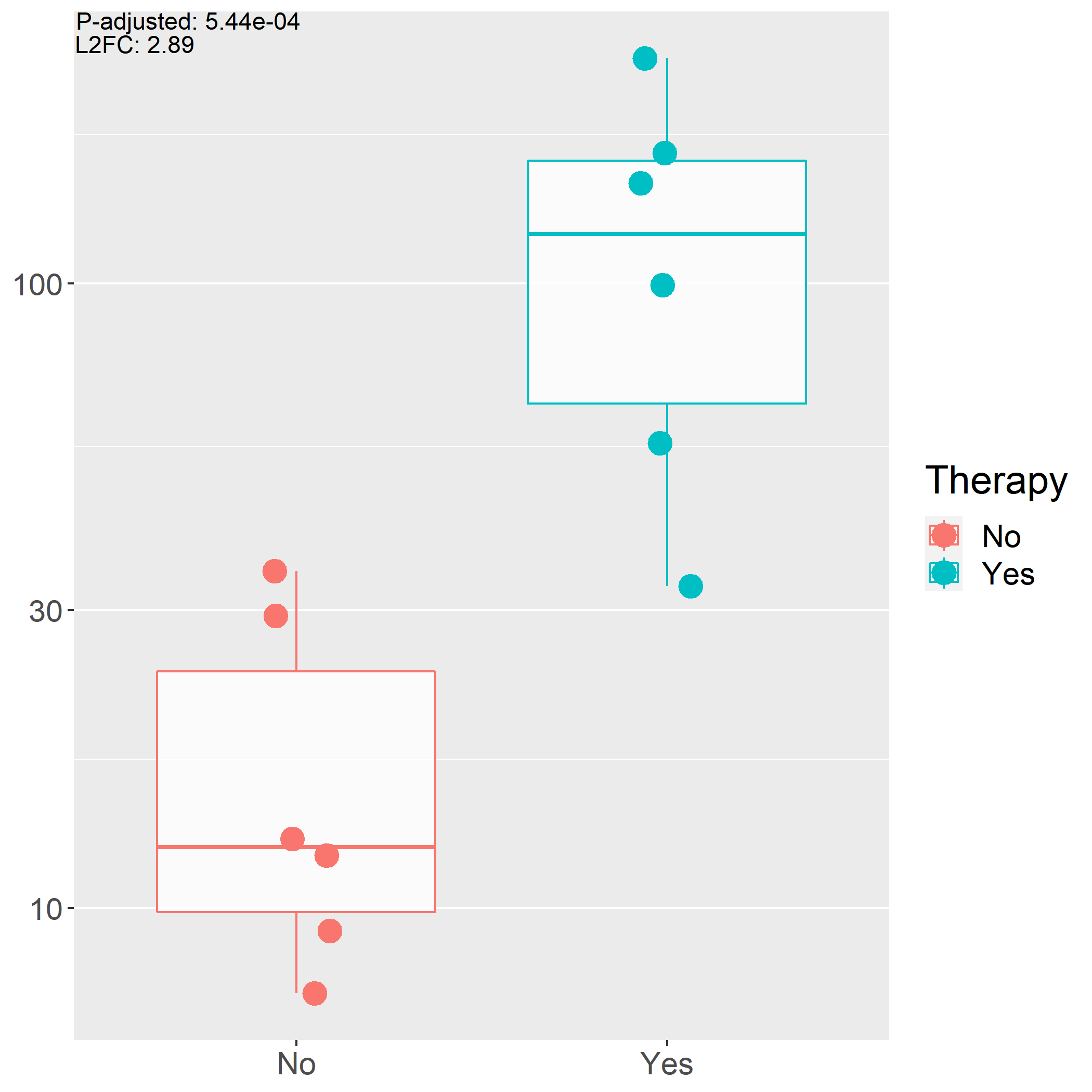 | 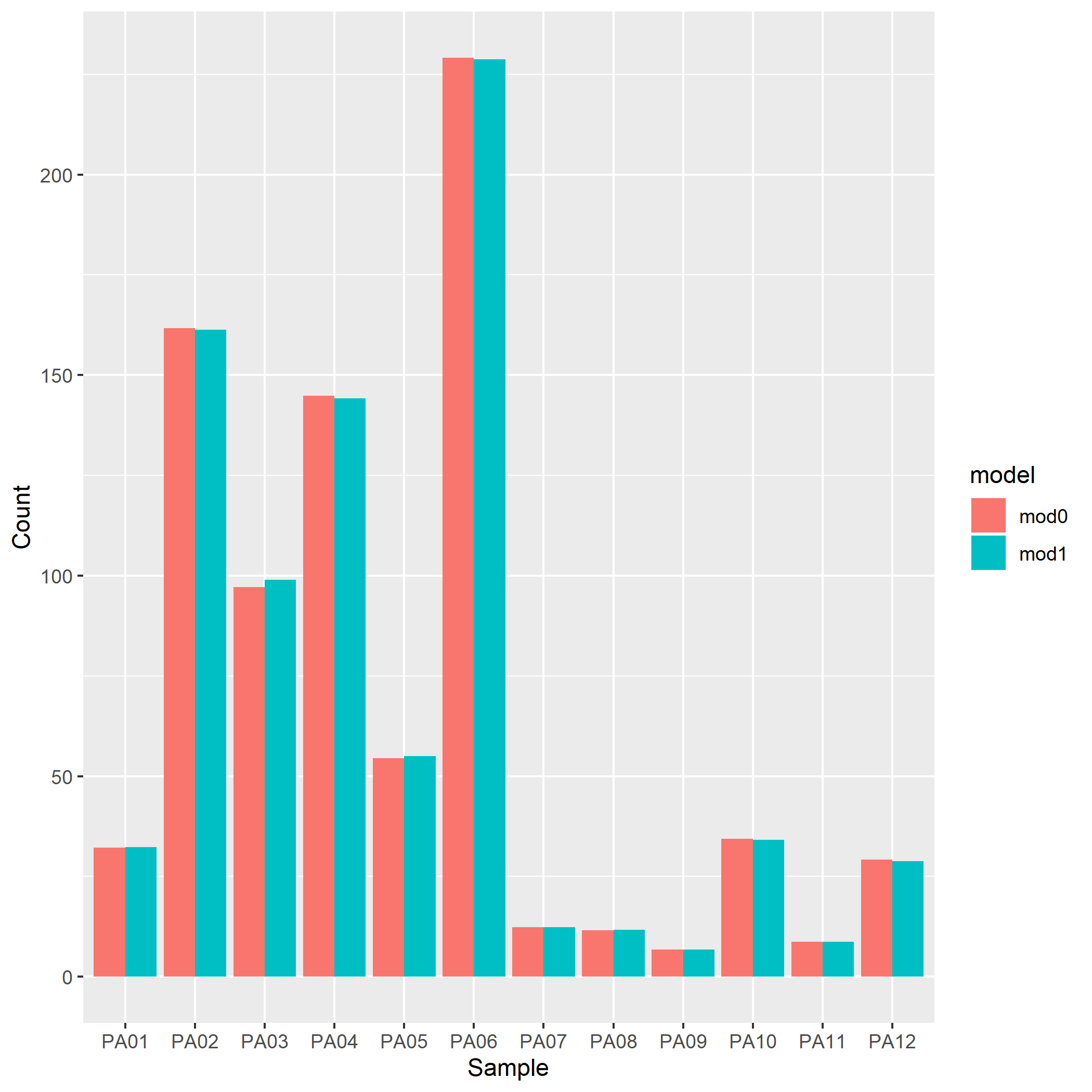 |
| *SEPTIN14* | Septin 14 | 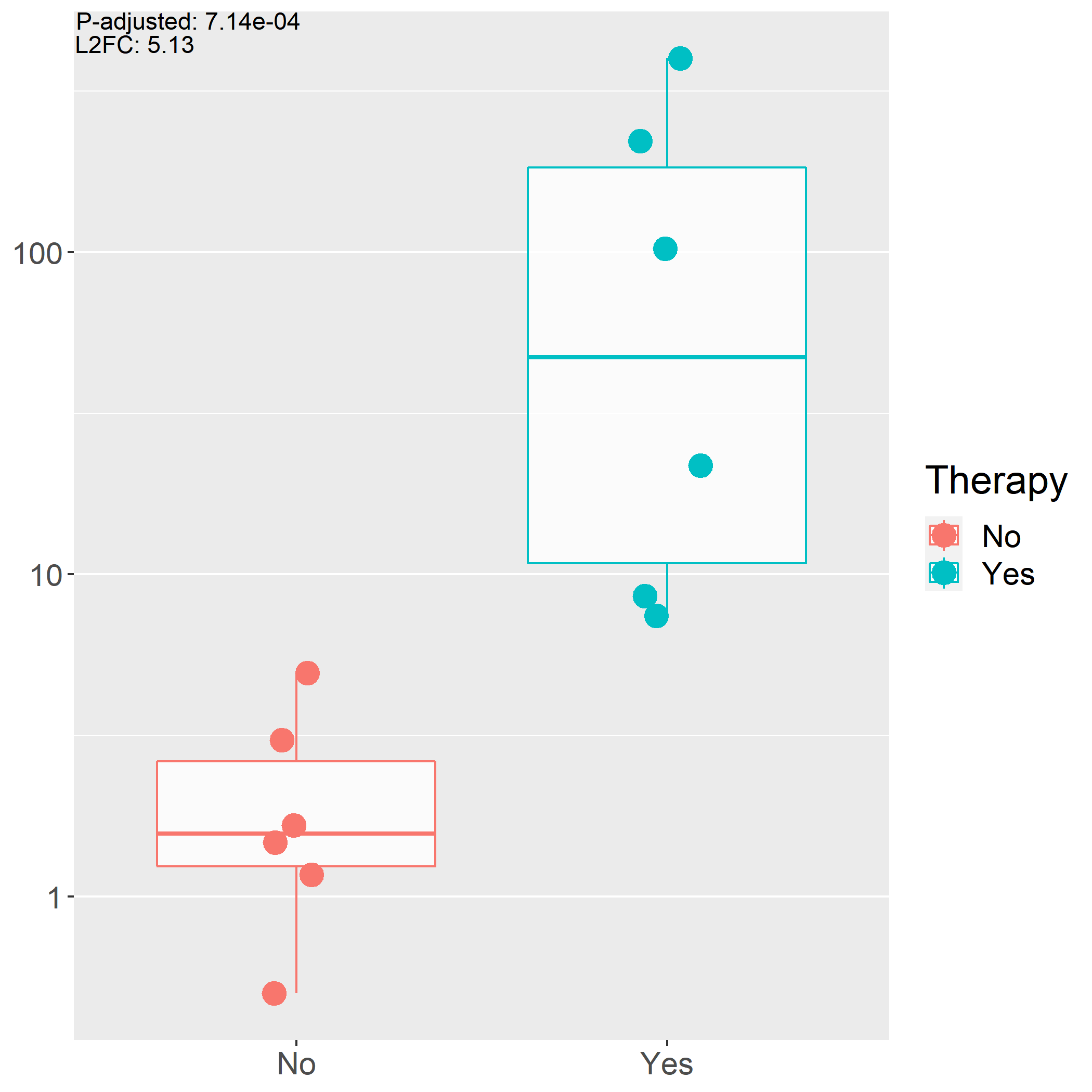 | 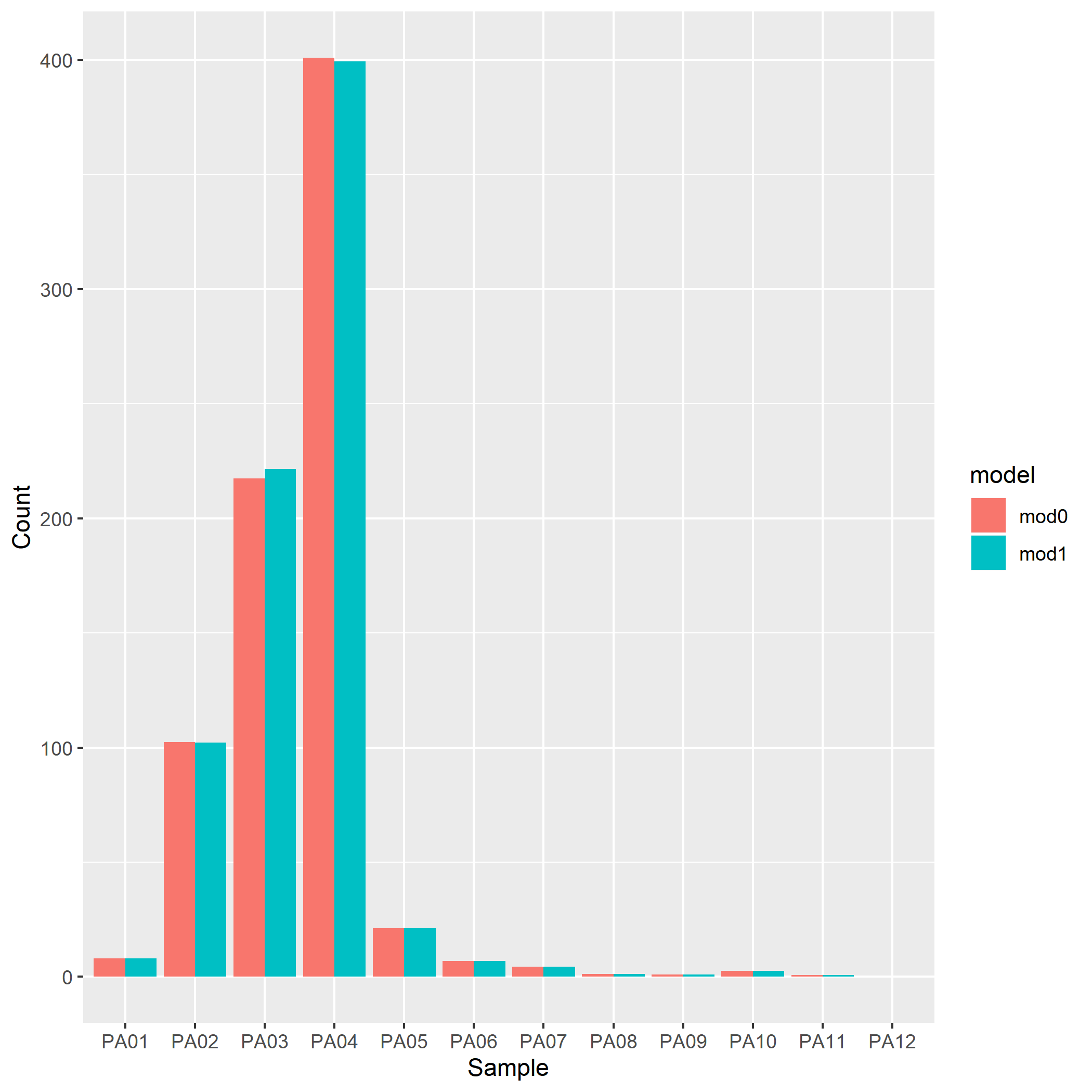 |
| *BCAM* | Basal Cell Adhesion Molecule (Lutheran Blood Group) | 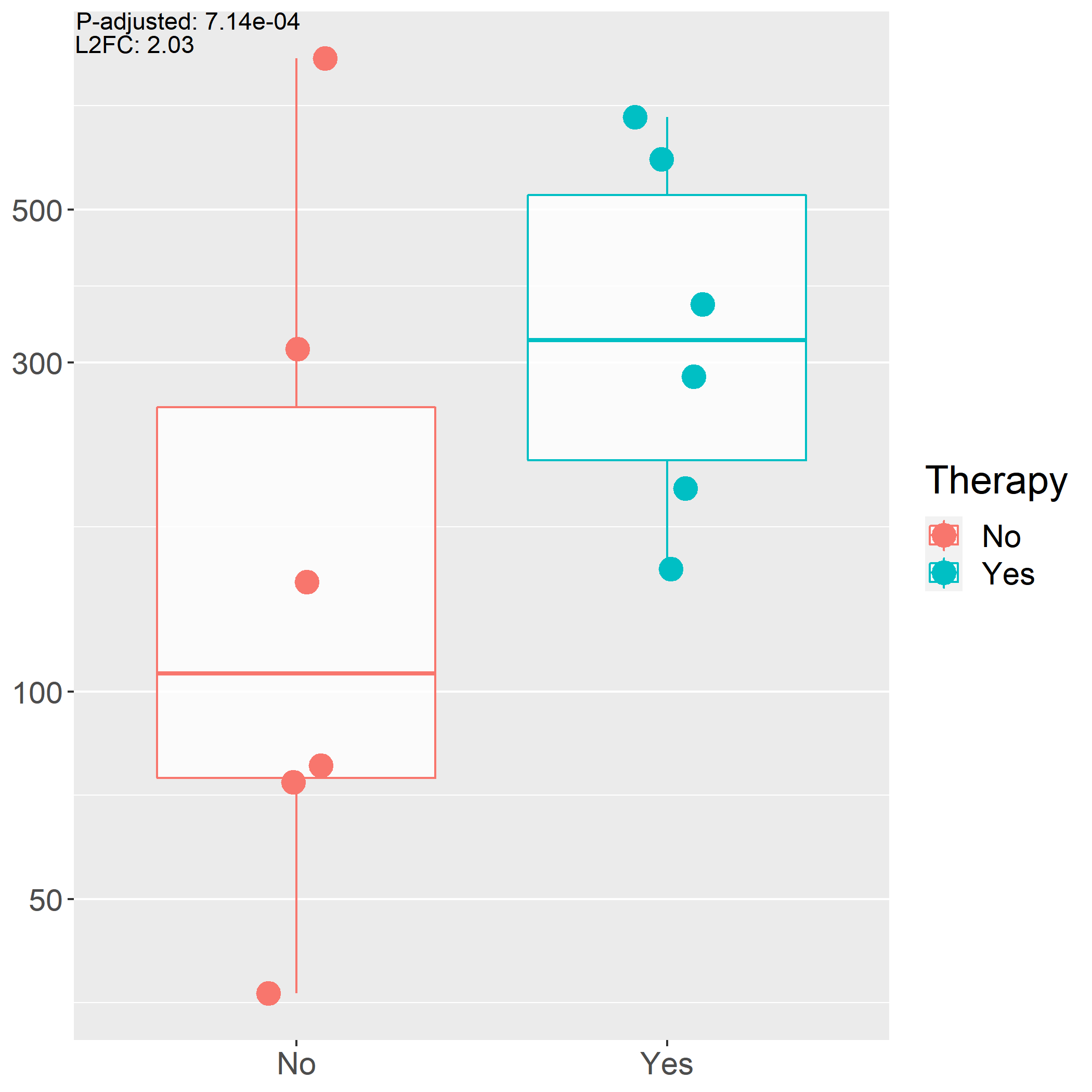 | 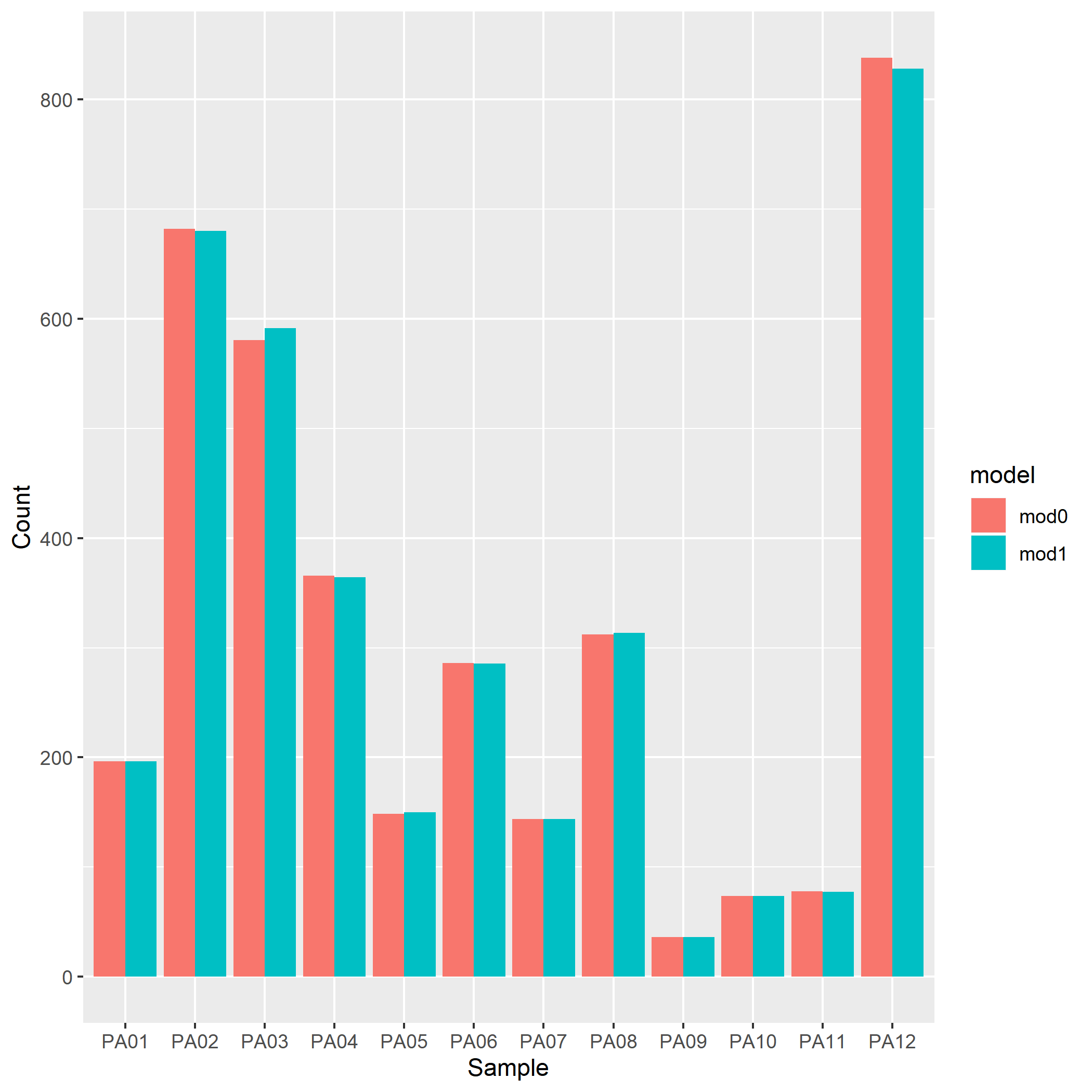 |
| *COL6A1* | Collagen Type VI Alpha 1 Chain | 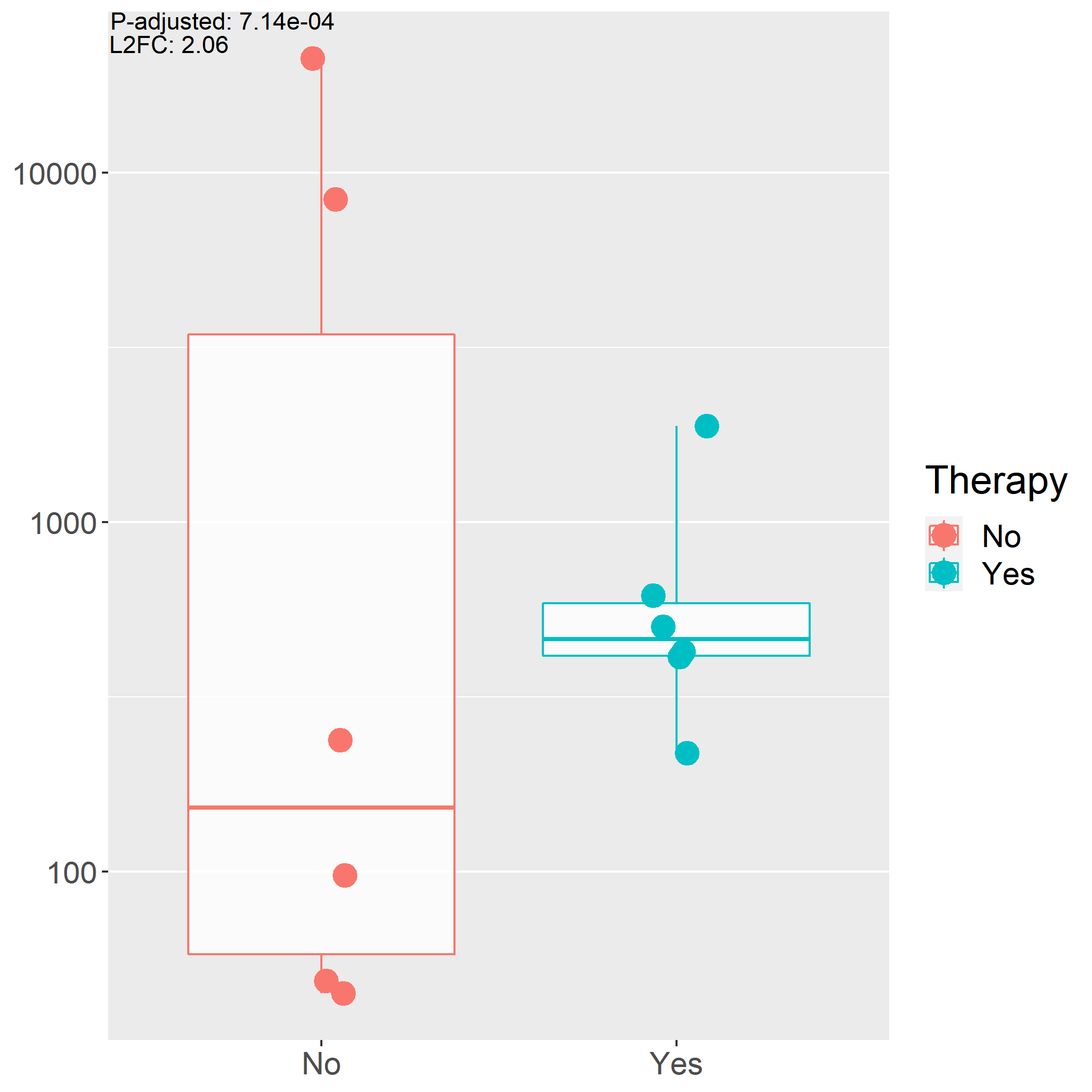 | 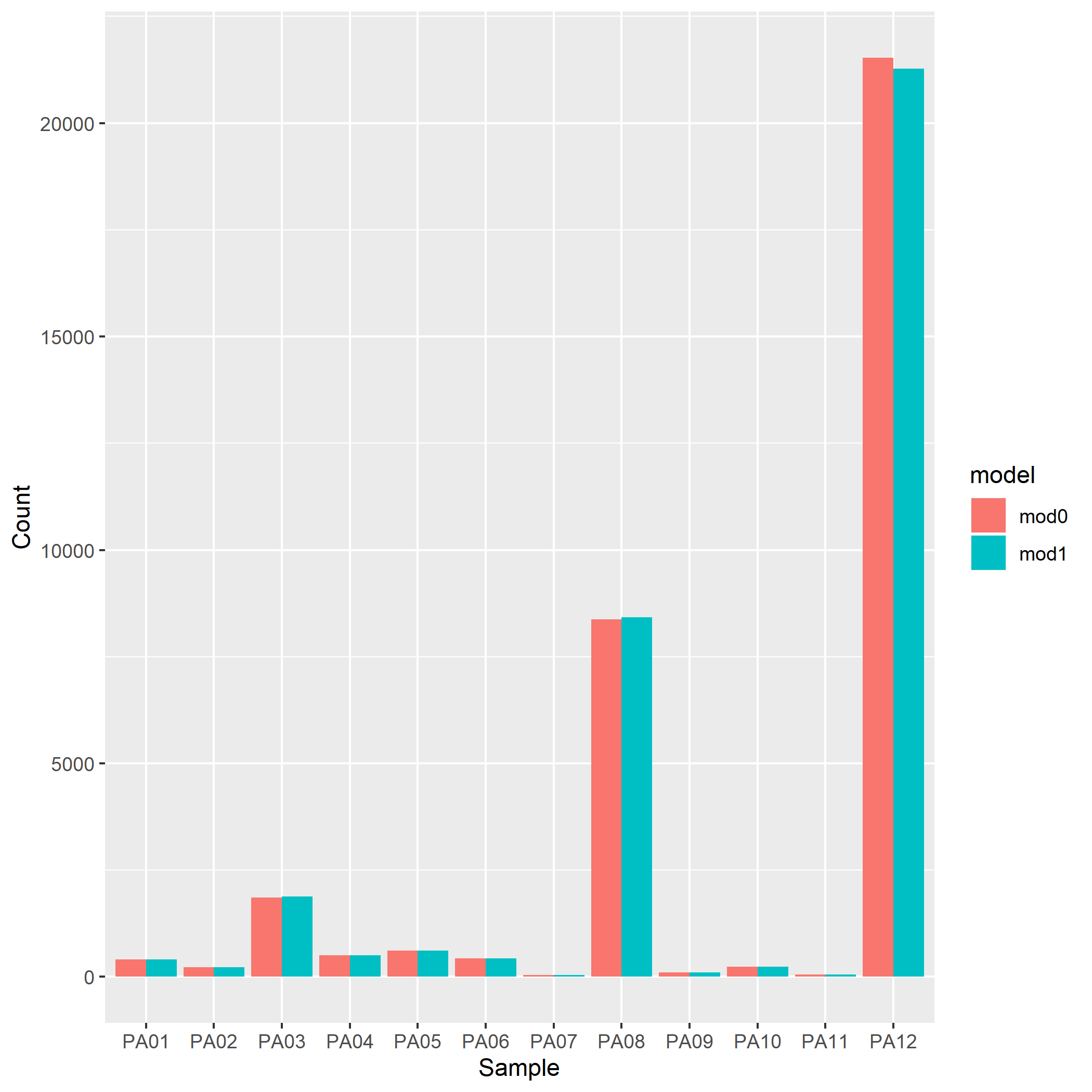 |
| *MACC1* | MET Transcriptional Regulator MACC1 | 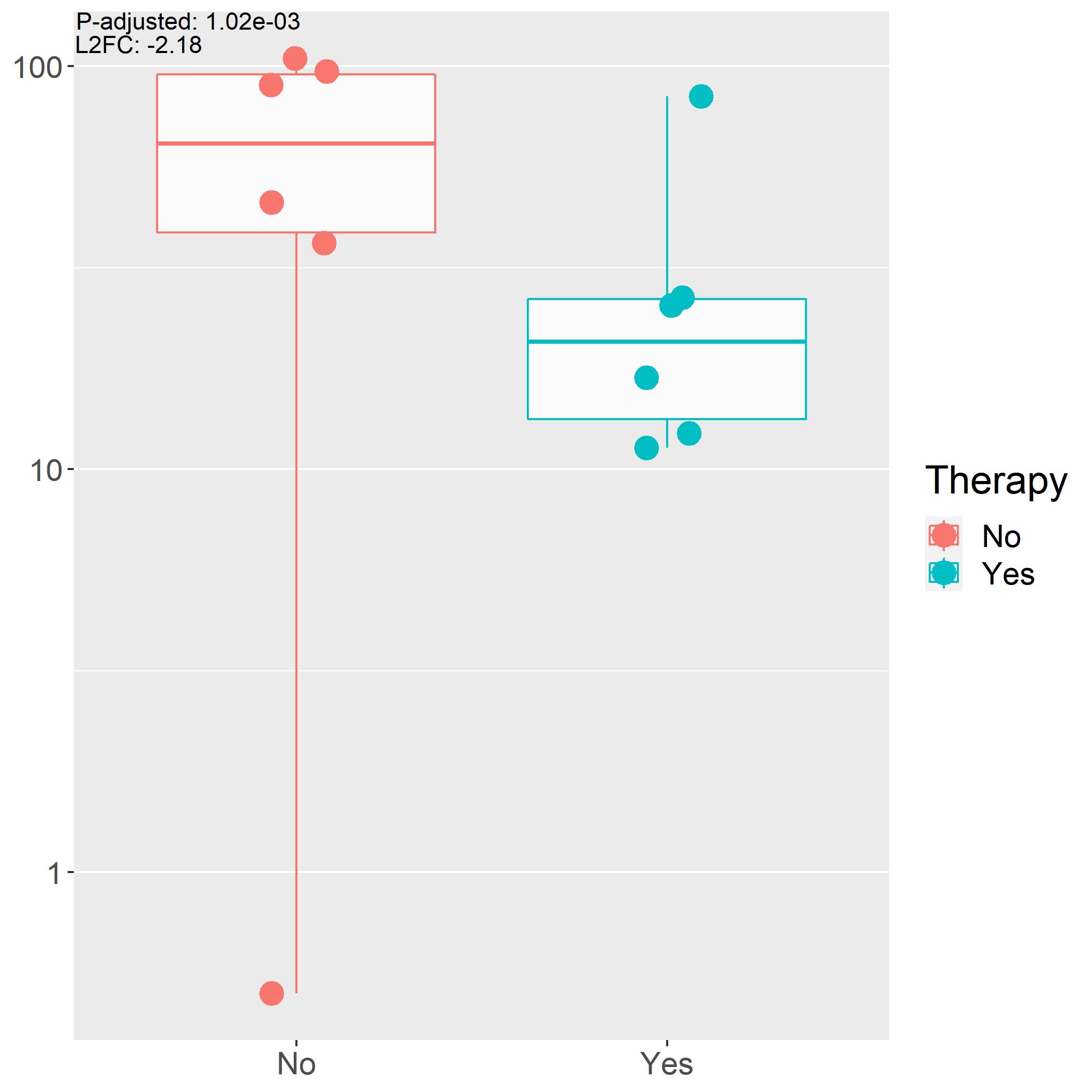 | 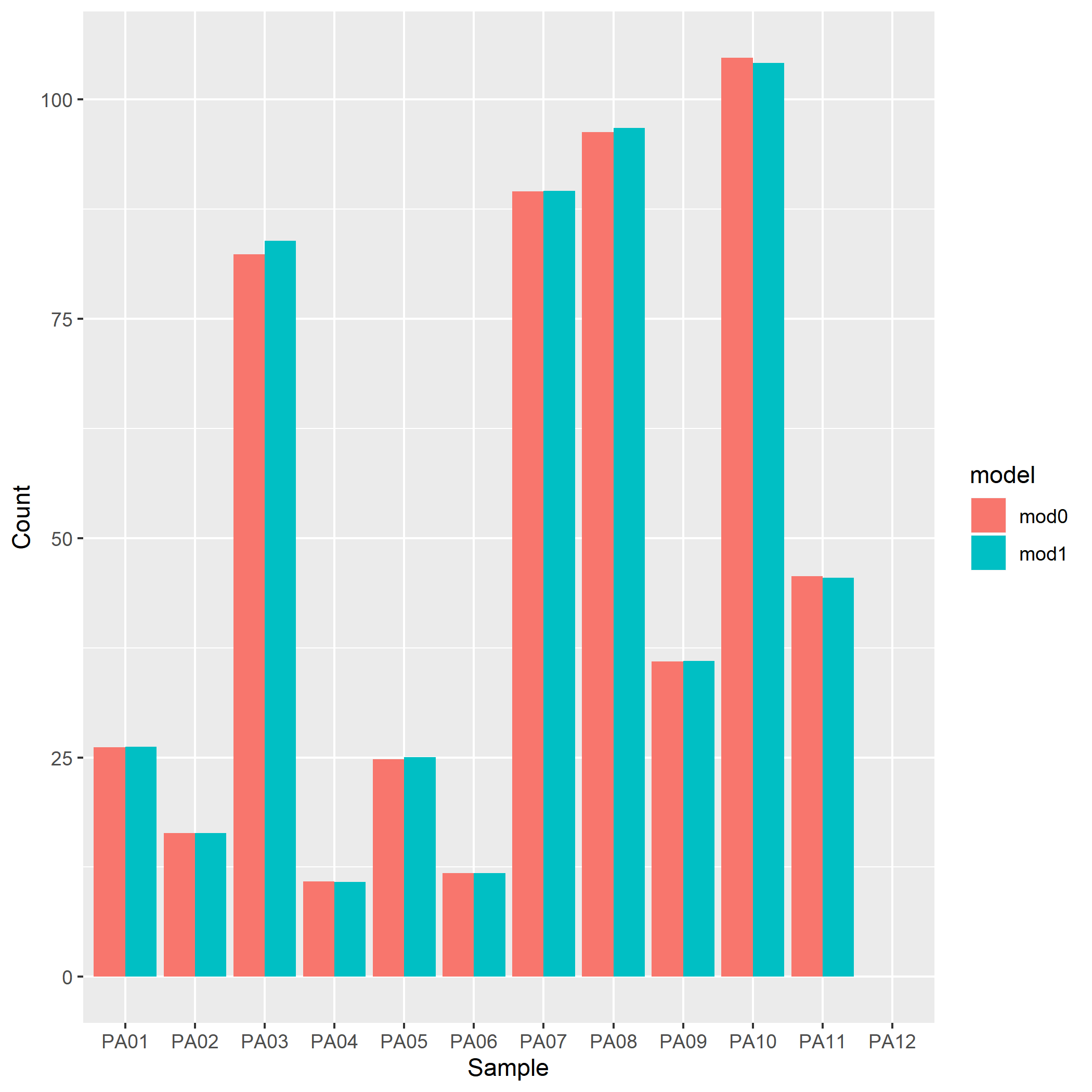 |
| *CILP* | Cartilage Intermediate Layer Protein | 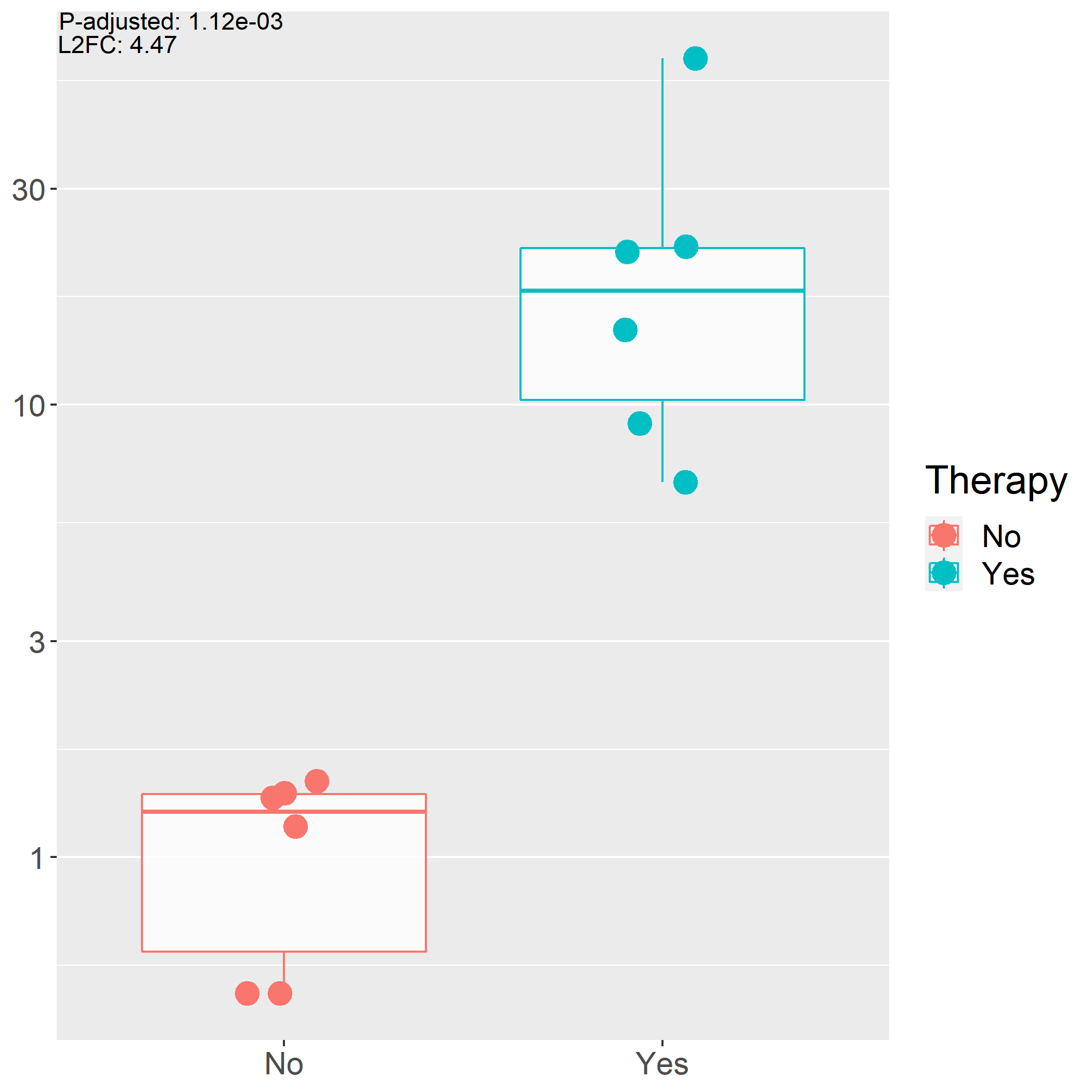 | 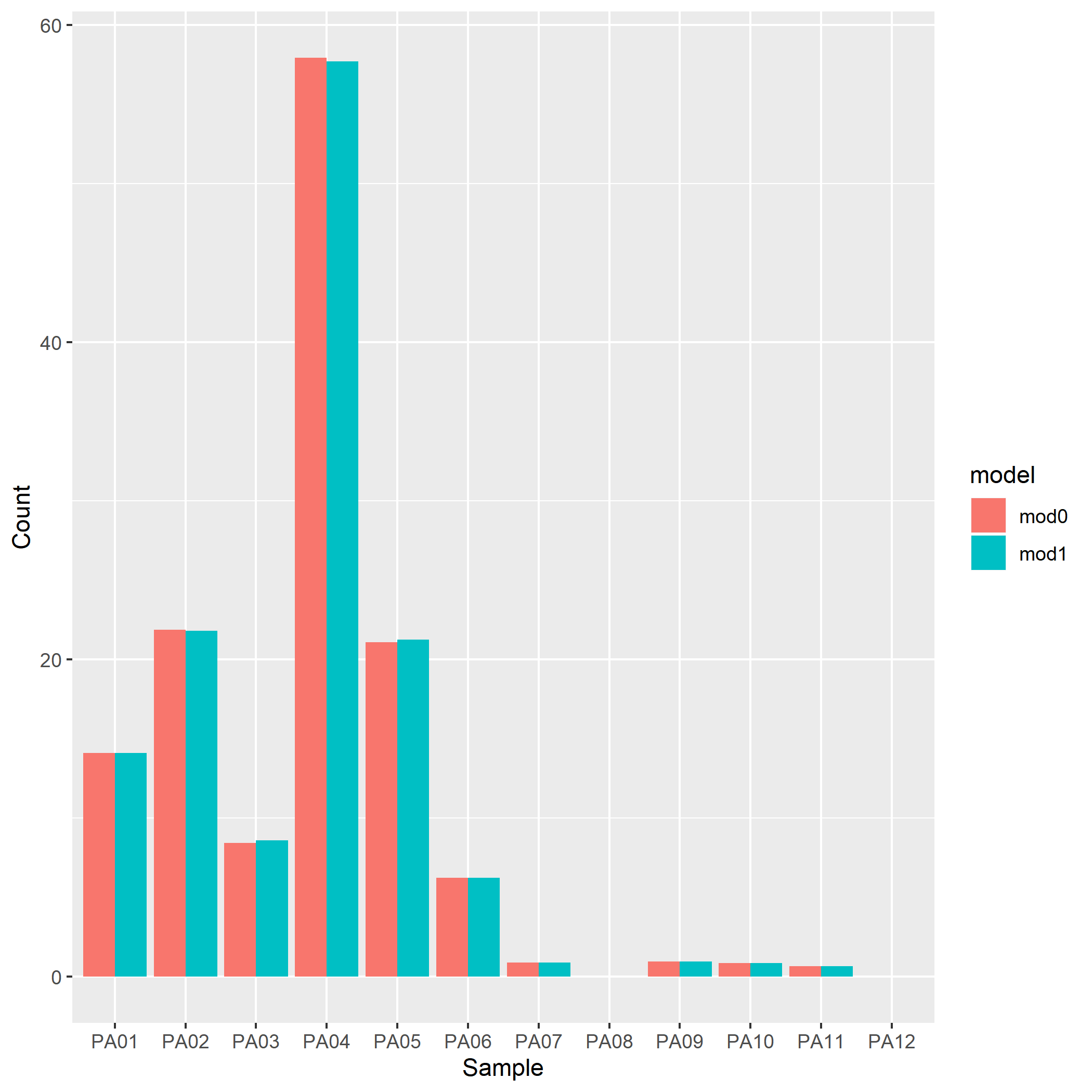 |
| *ARC* | Activity Regulated Cytoskeleton Associated Protein | 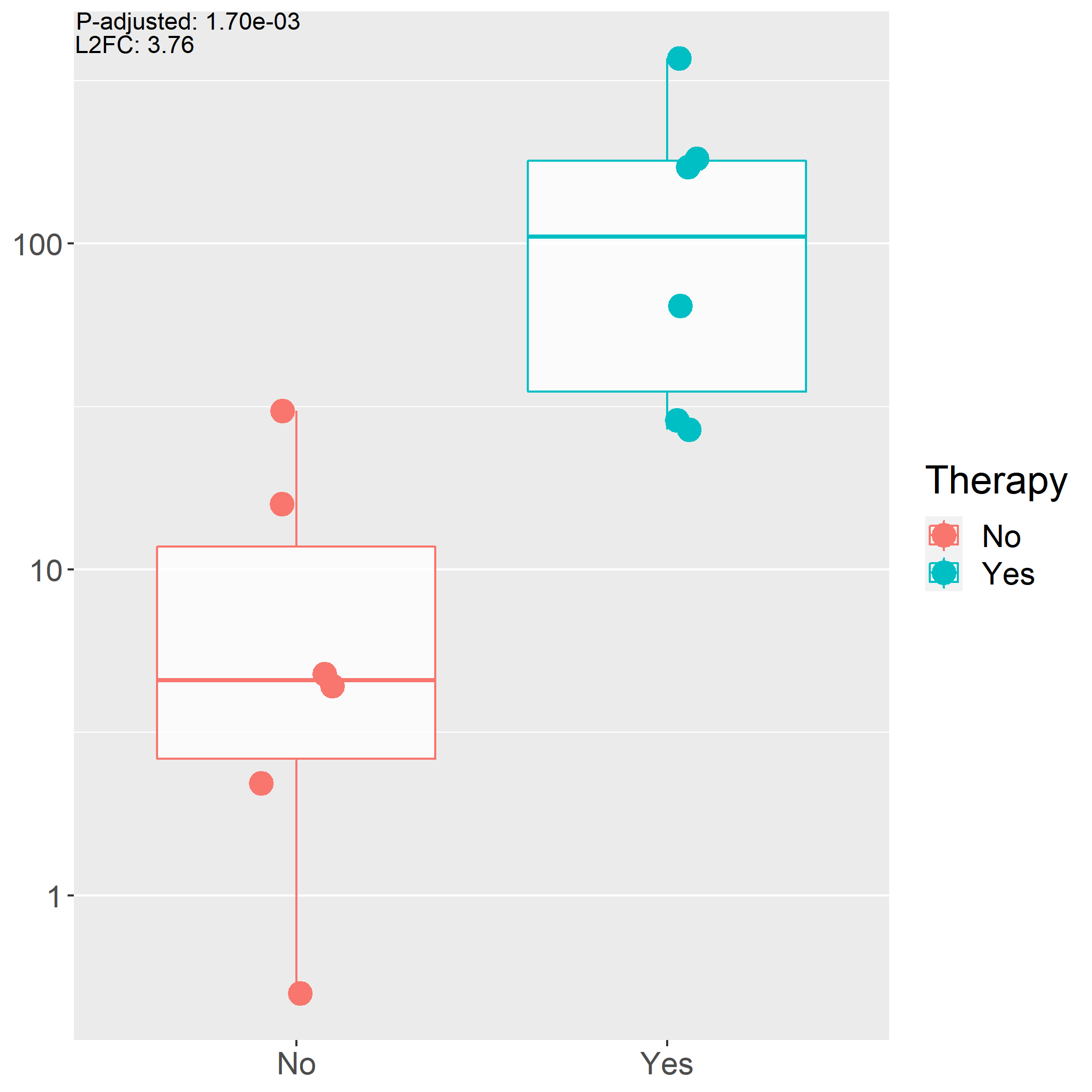 | 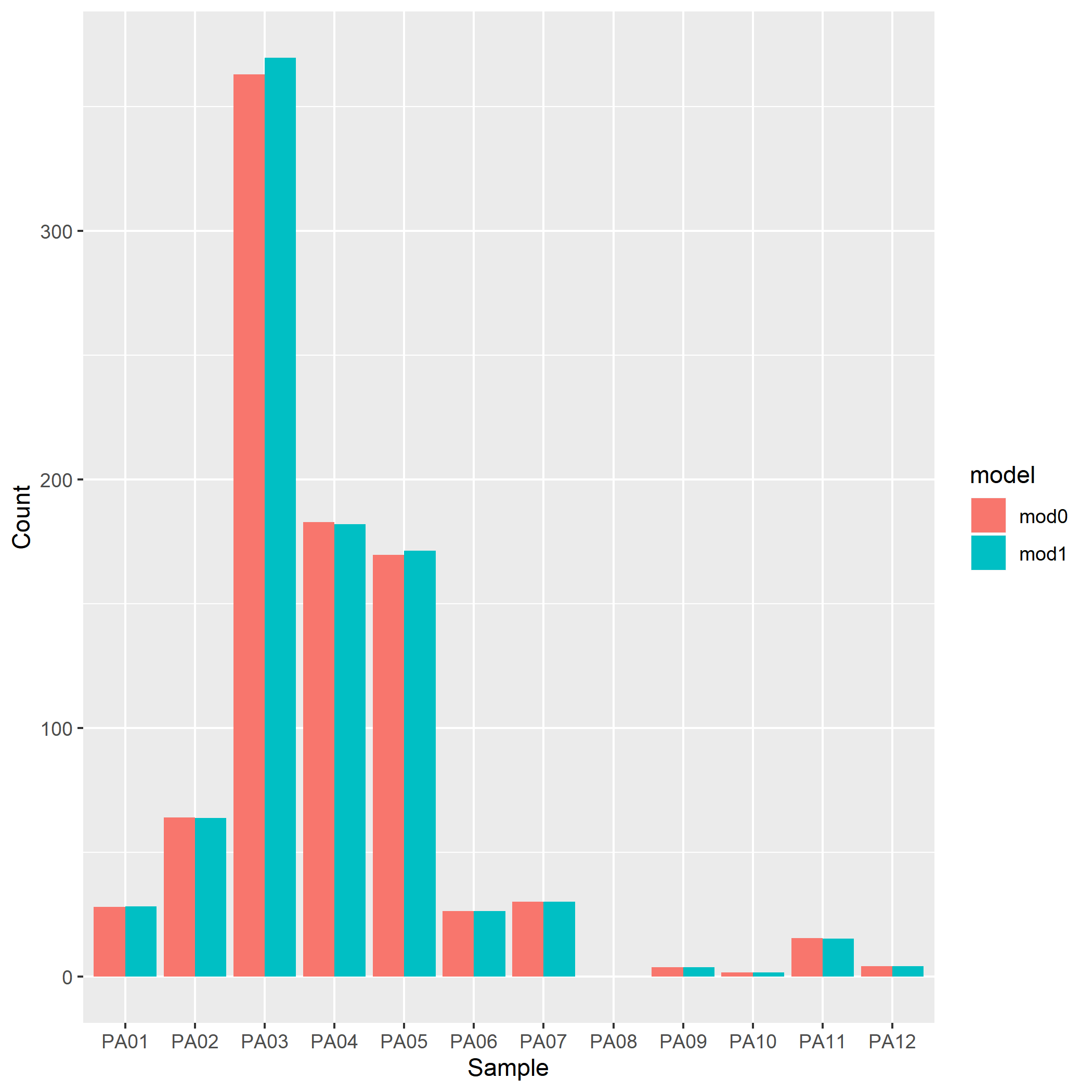 |
| *STUM* | Stum, Mechanosensory Transduction Mediator Homolog | 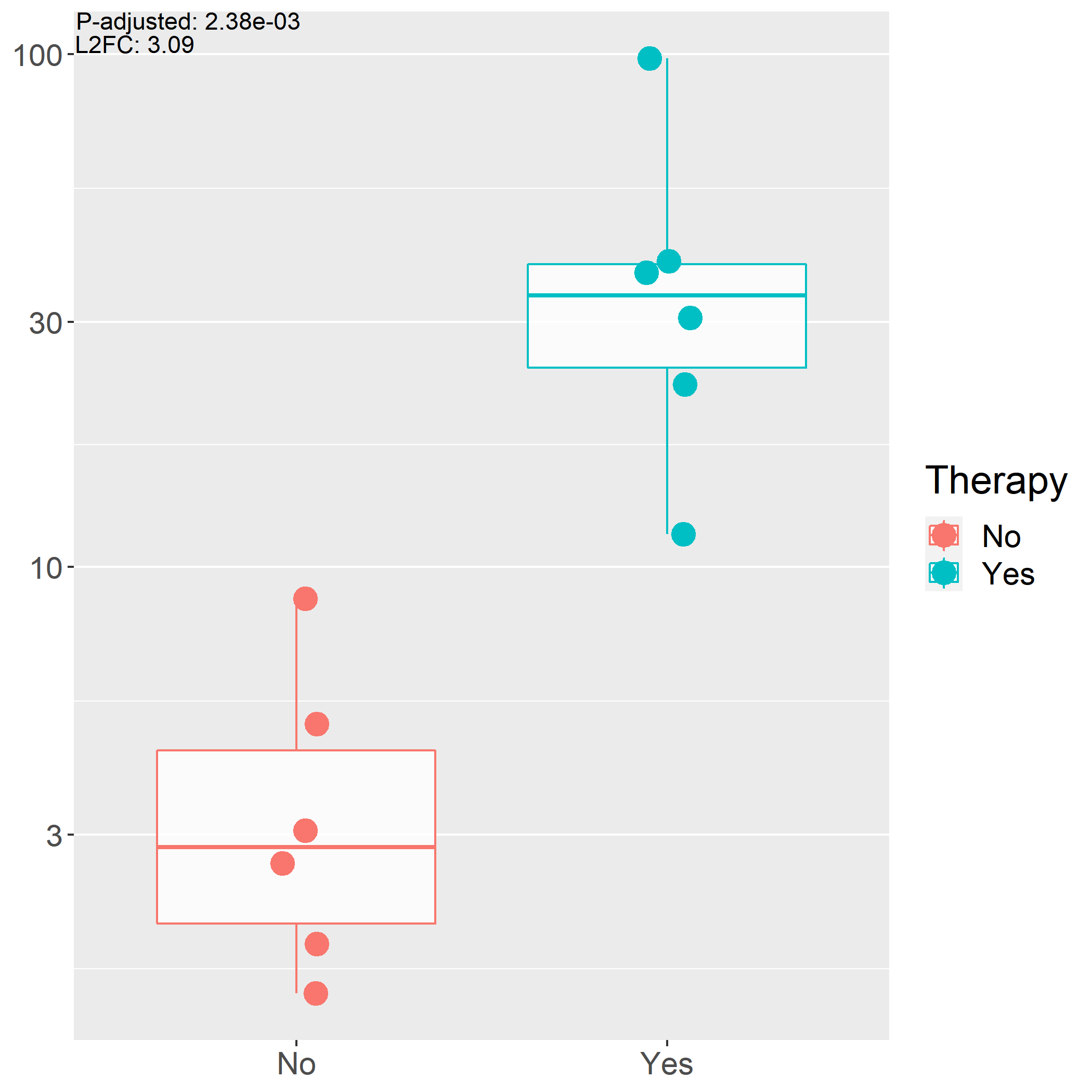 | 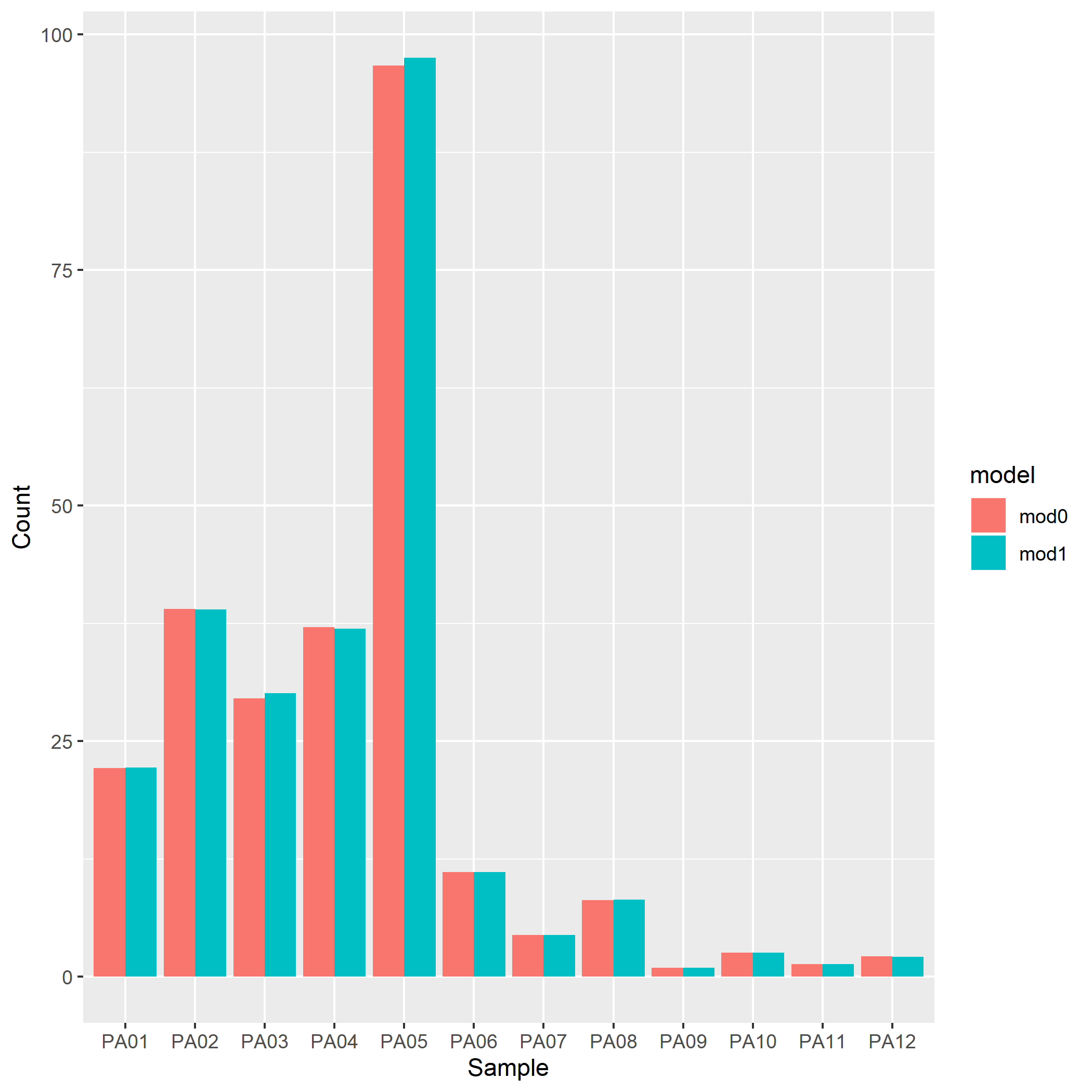 |
| *NPTX1* | Neuronal Pentraxin 1 | 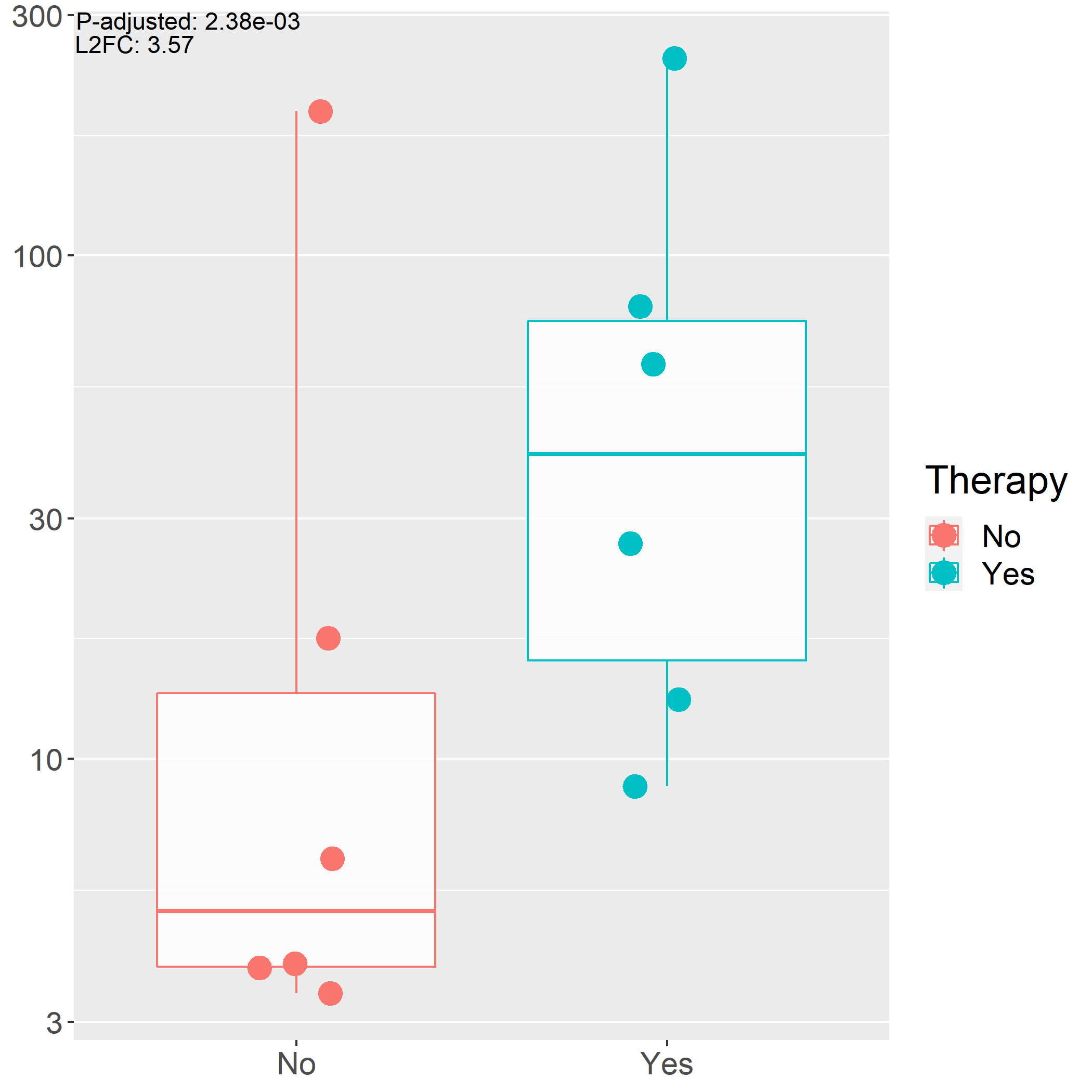 | 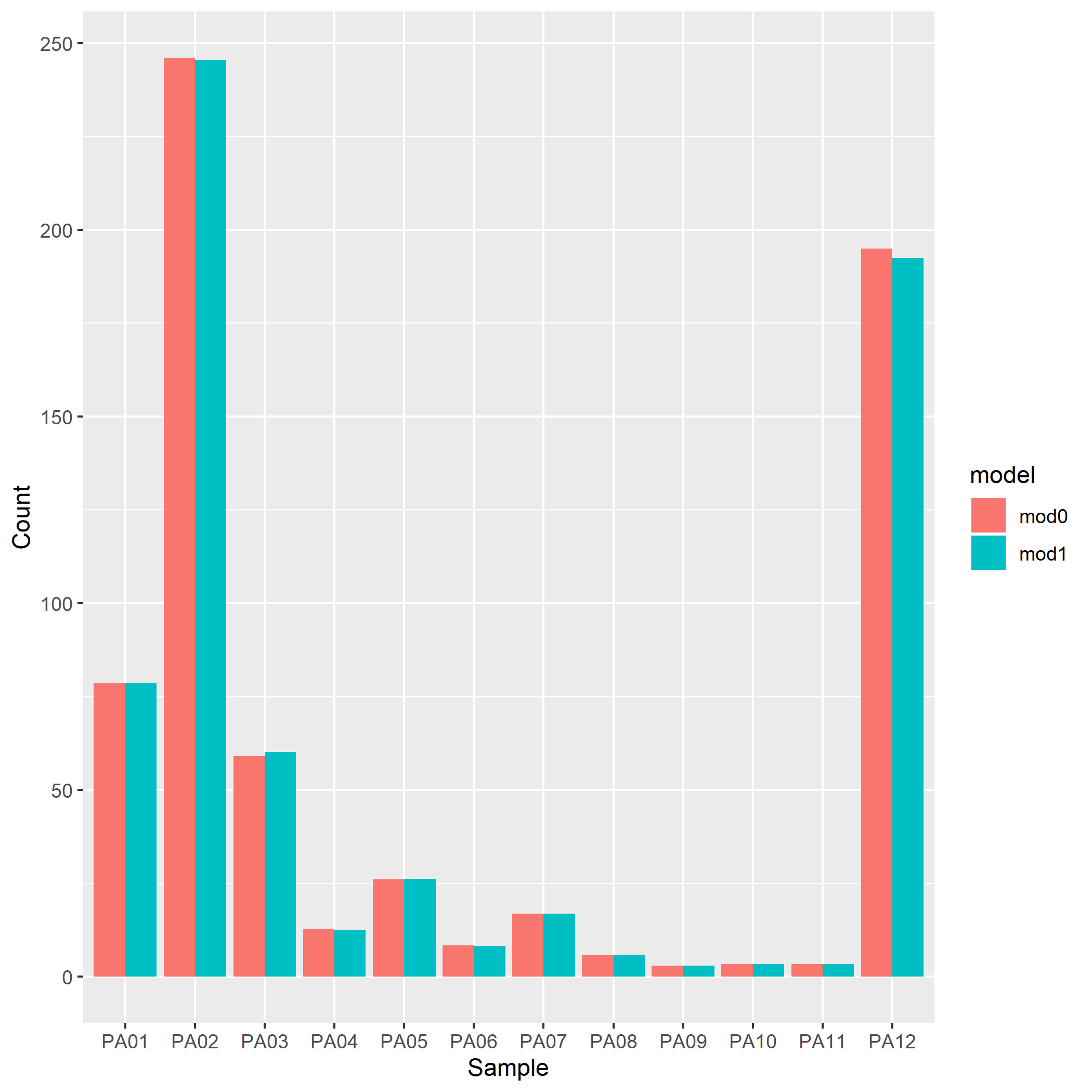 |
| *CLU* | Clusterin | 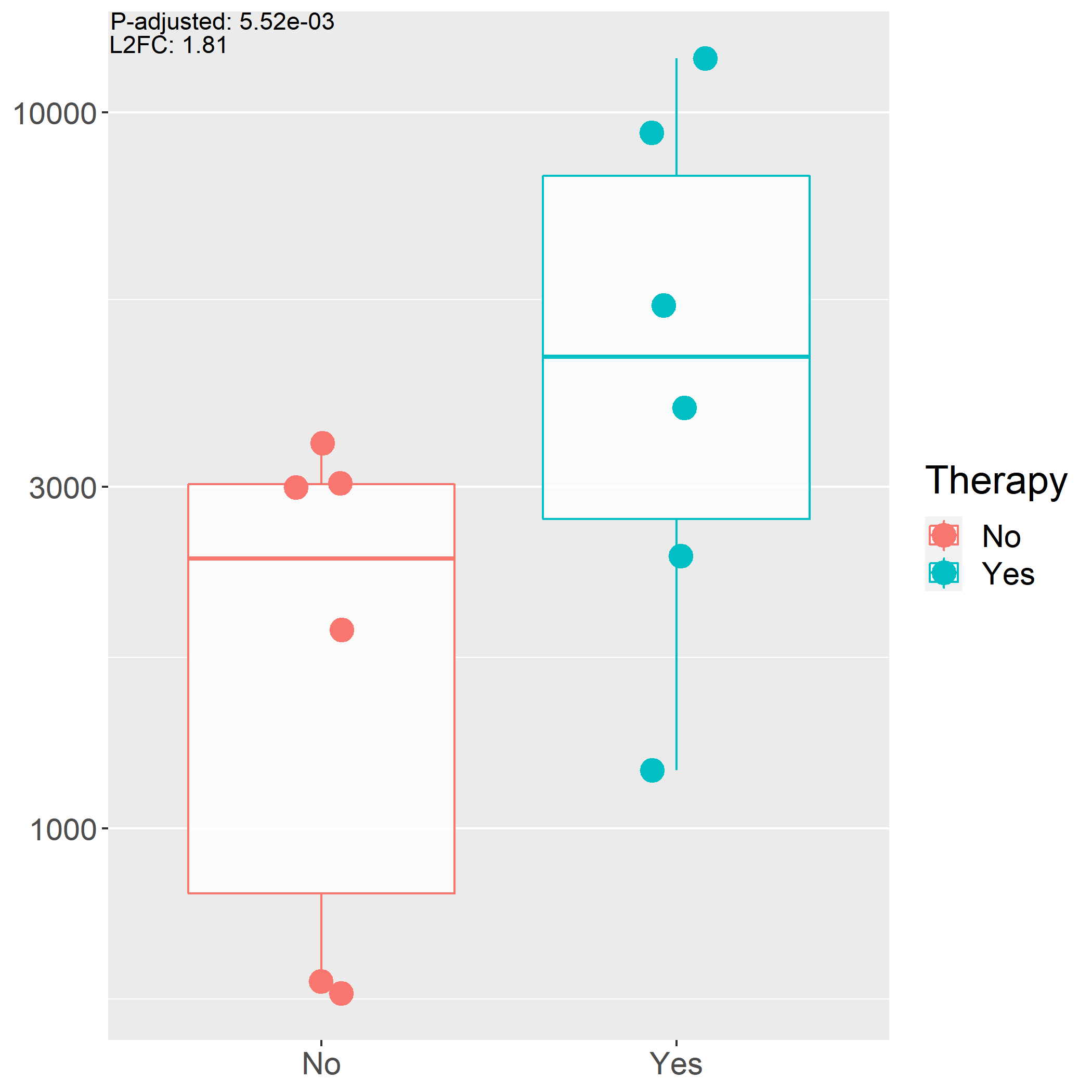 | 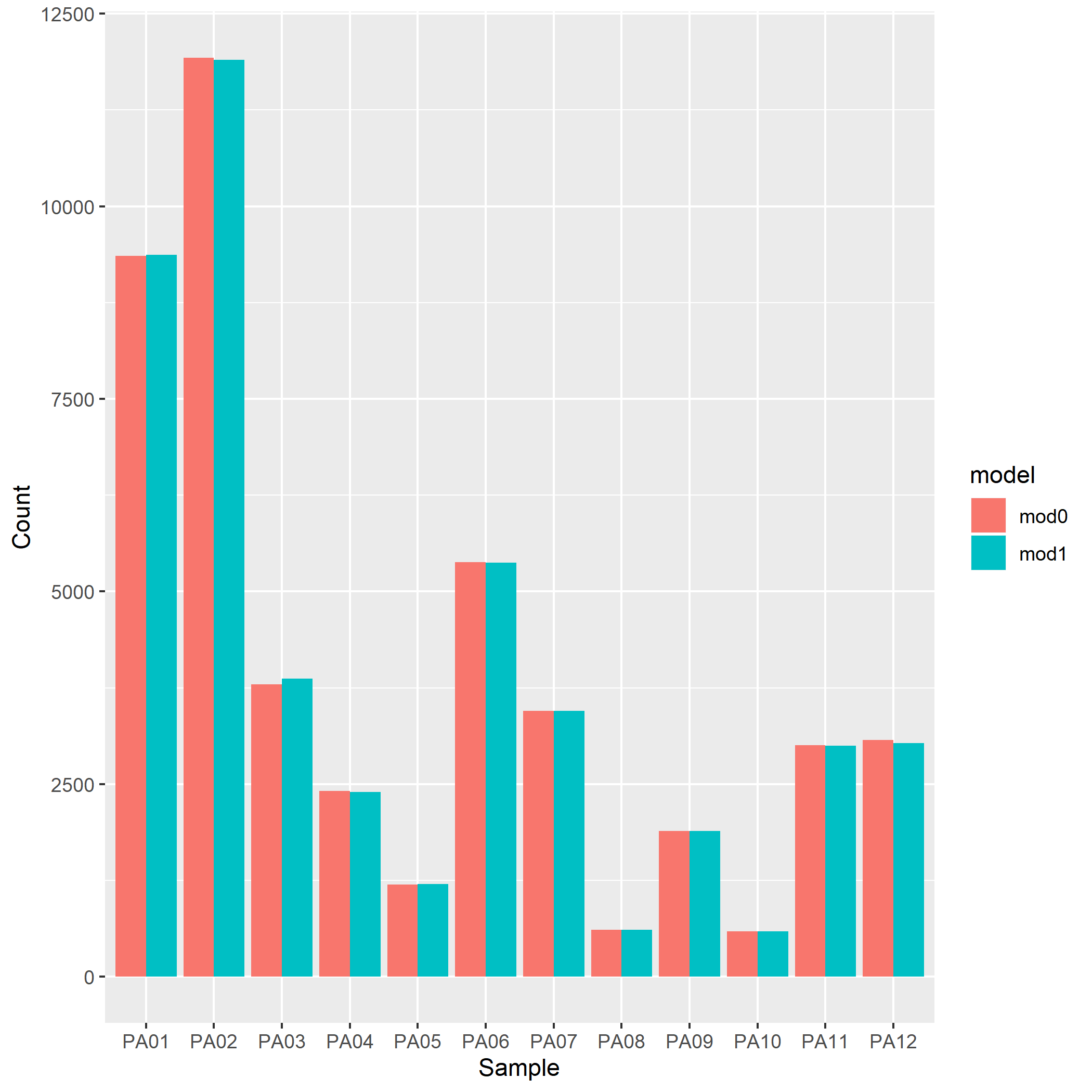 |
| *MPPED1* | Metallophosphoesterase Domain Containing 1 | 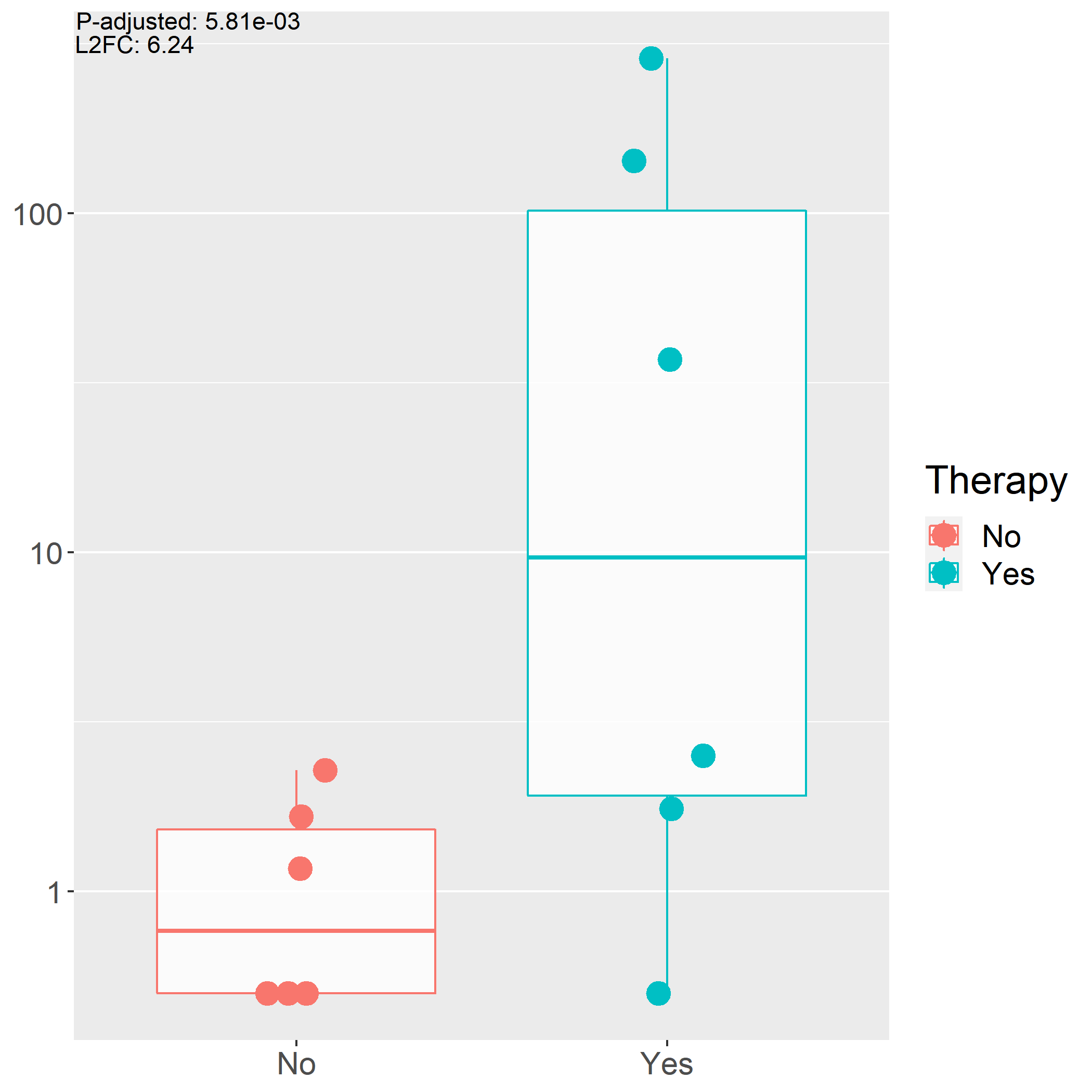 | 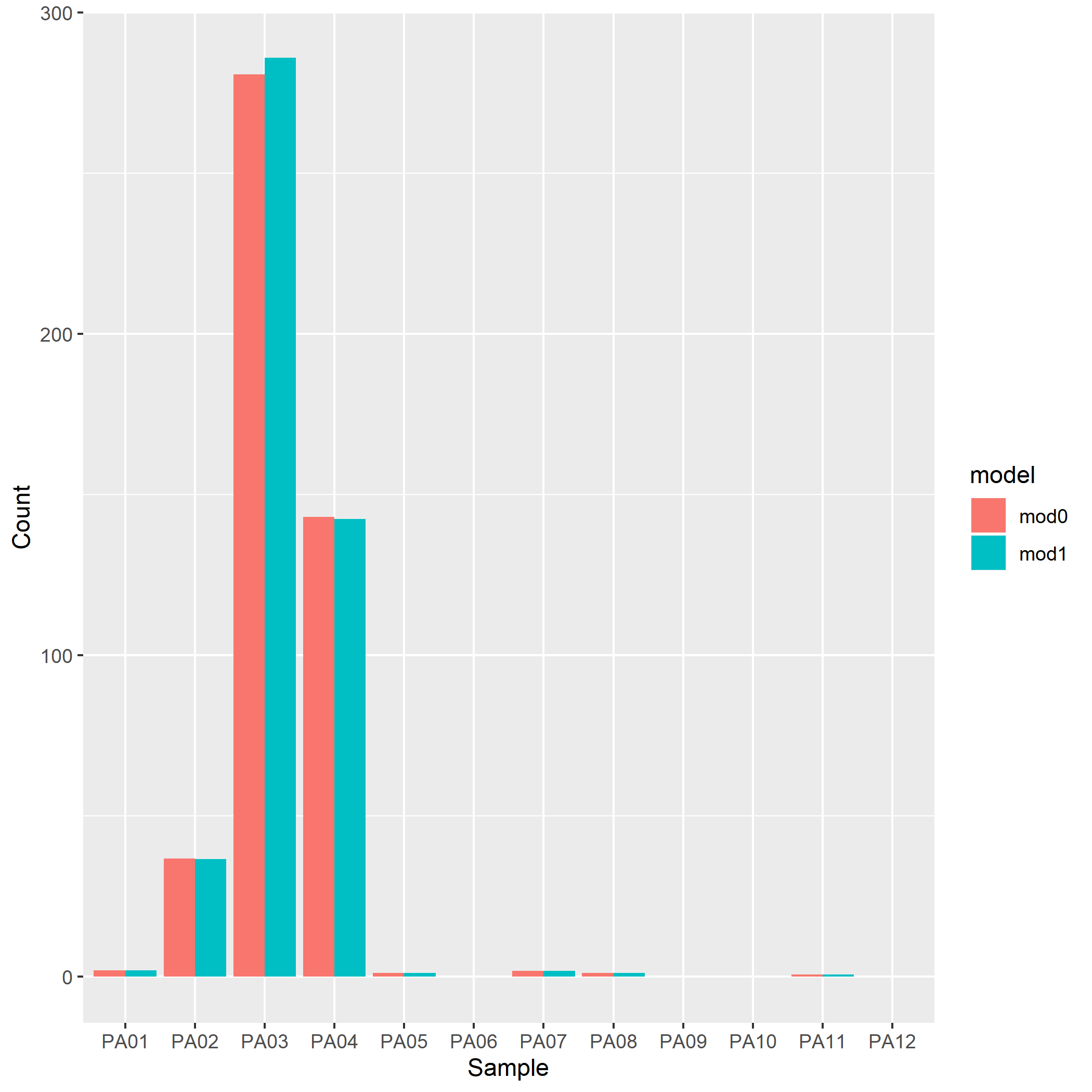 |
| *COL8A2* | Collagen Type VIII Alpha 2 Chain | 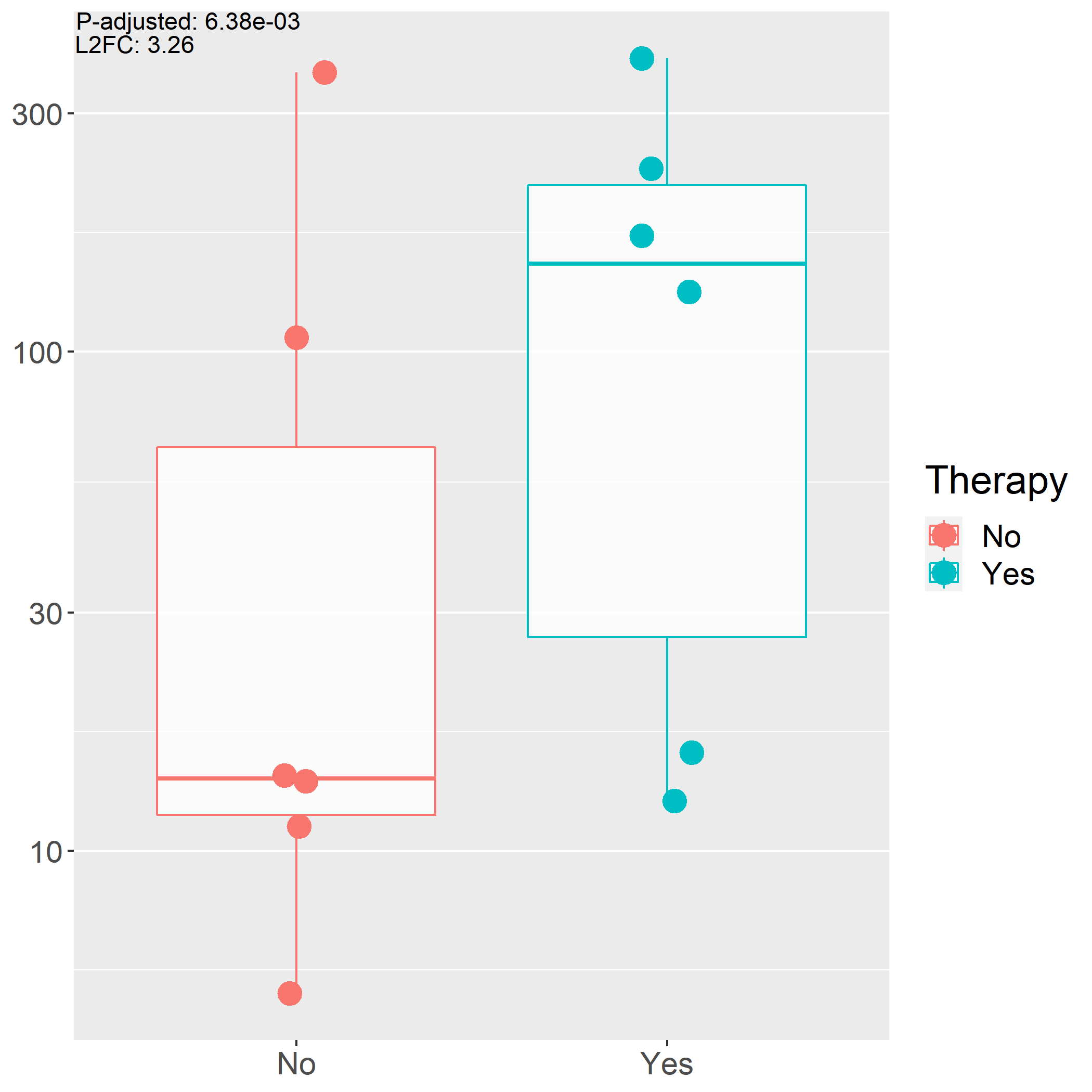 | 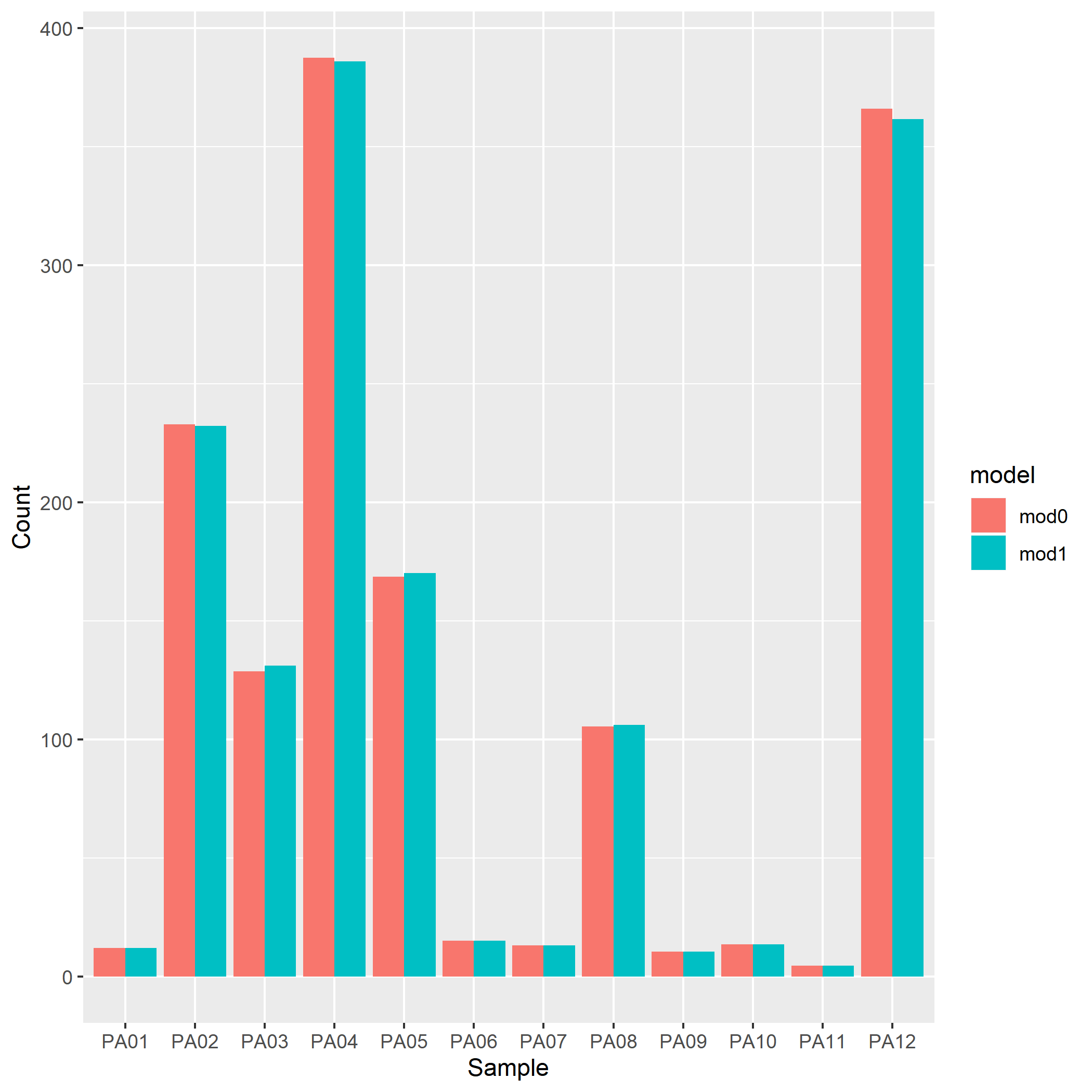 |
| *GRHL2* | Grainyhead Like Transcription Factor 2 | 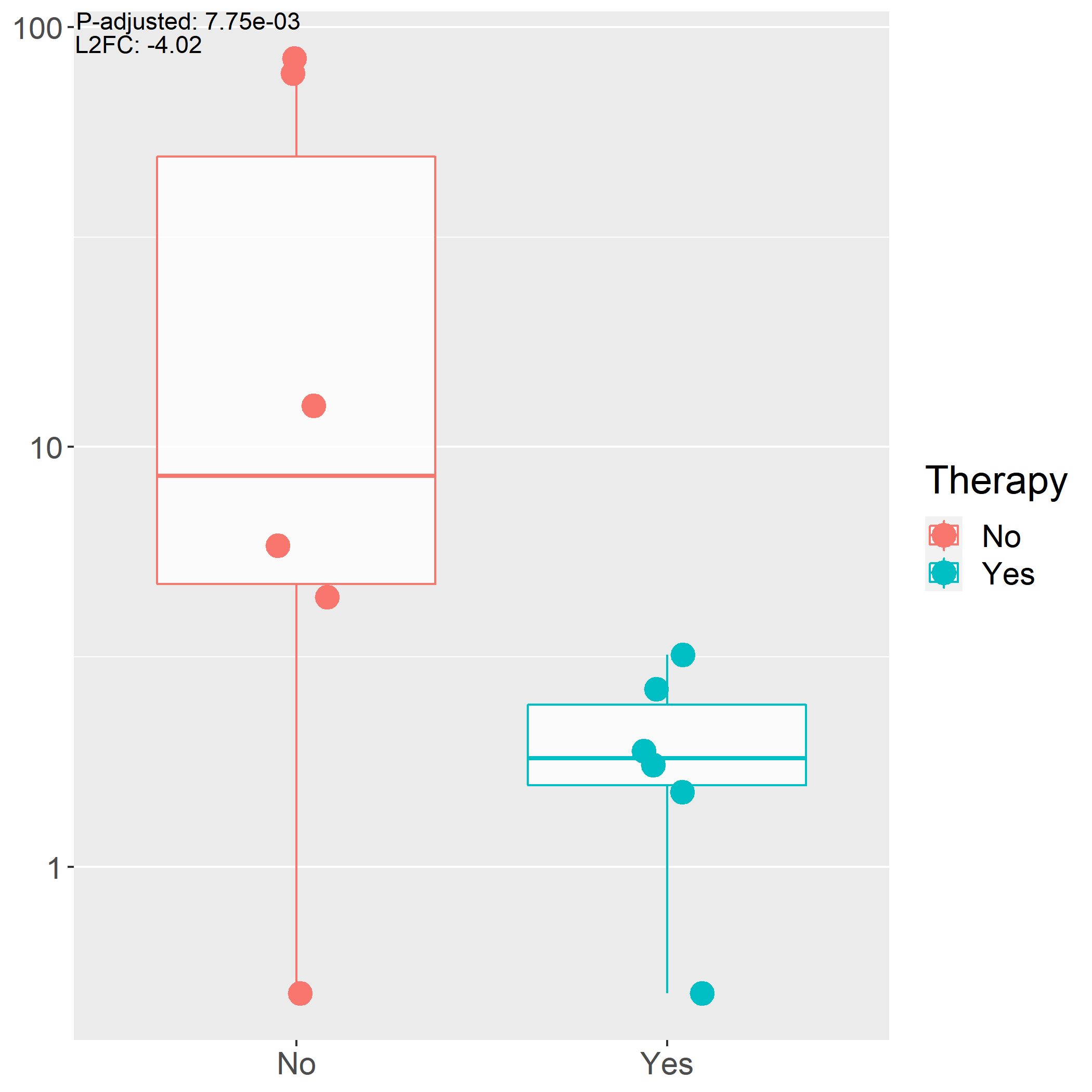 | 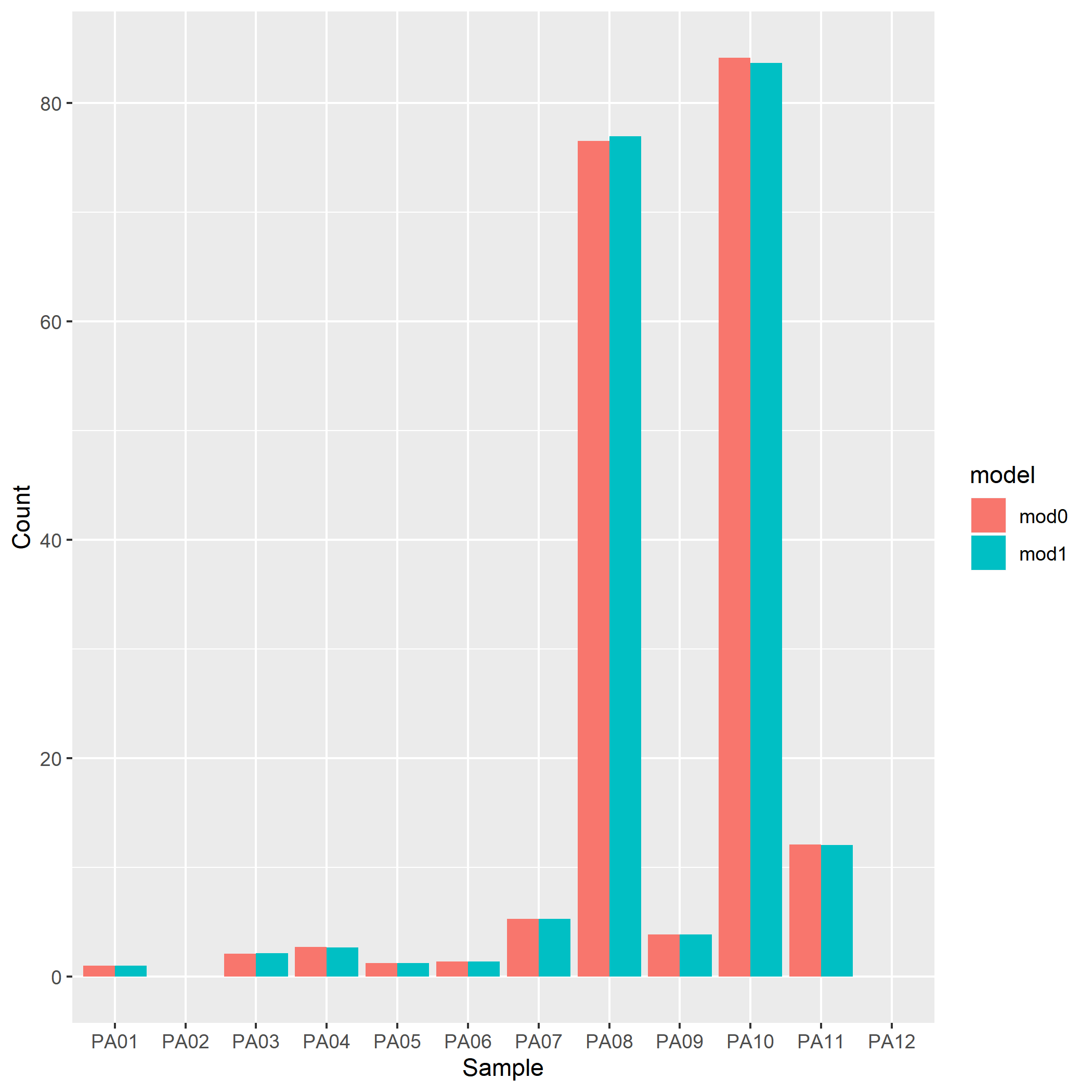 |
| *SSC5D* | Scavenger Receptor Cysteine Rich Family Member With 5 Domains | 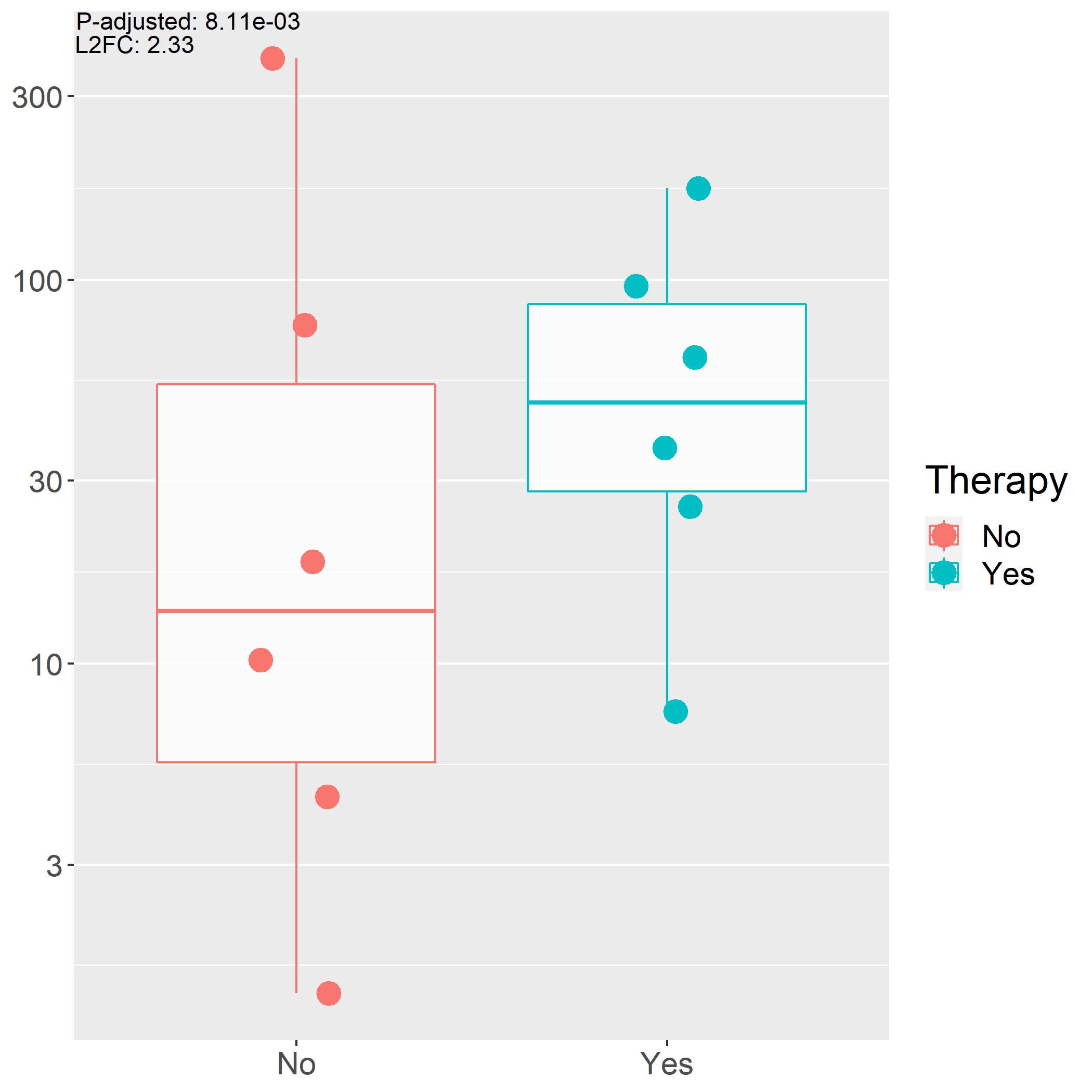 | 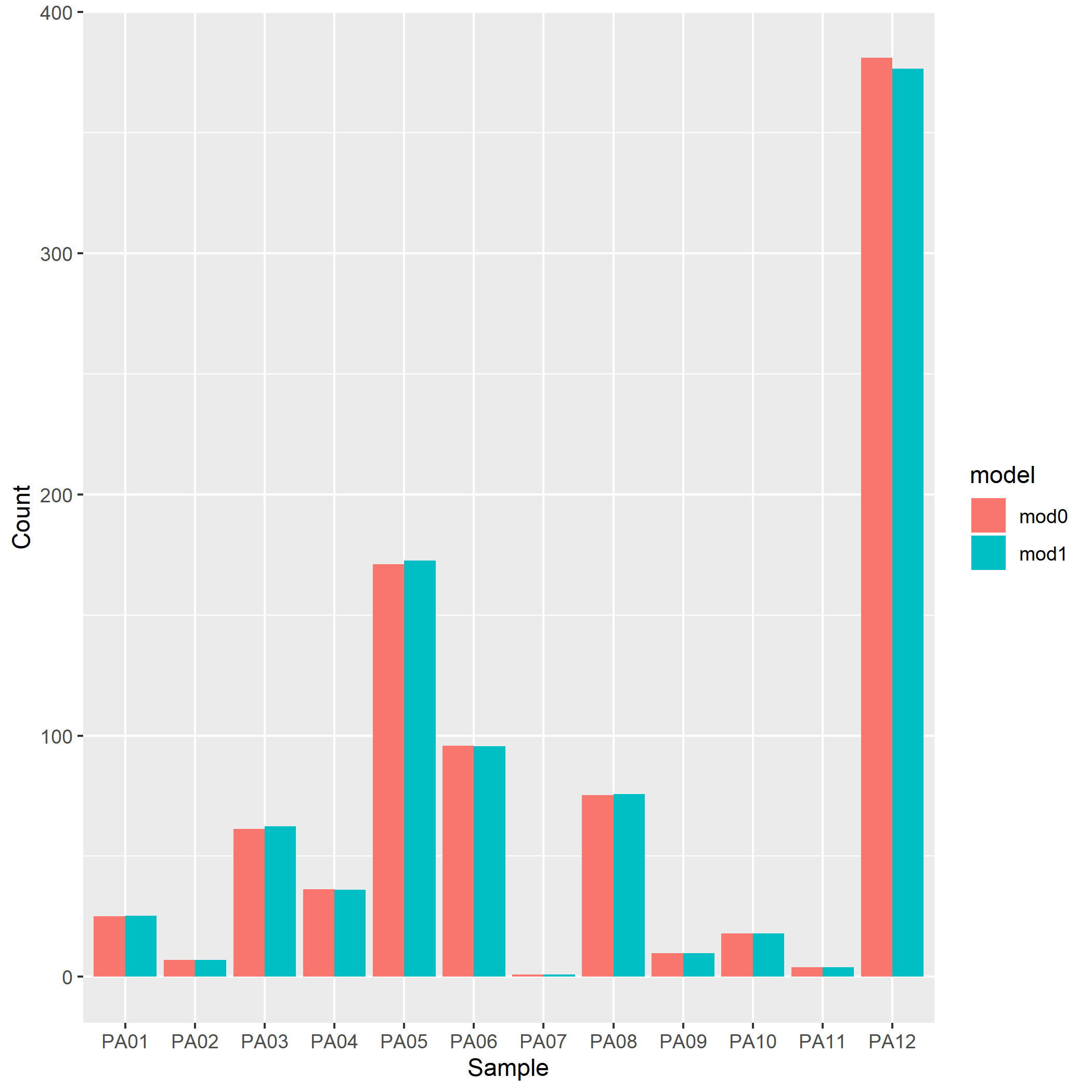 |
| *GRIK3* | Glutamate Ionotropic Receptor Kainate Type Subunit 3 | 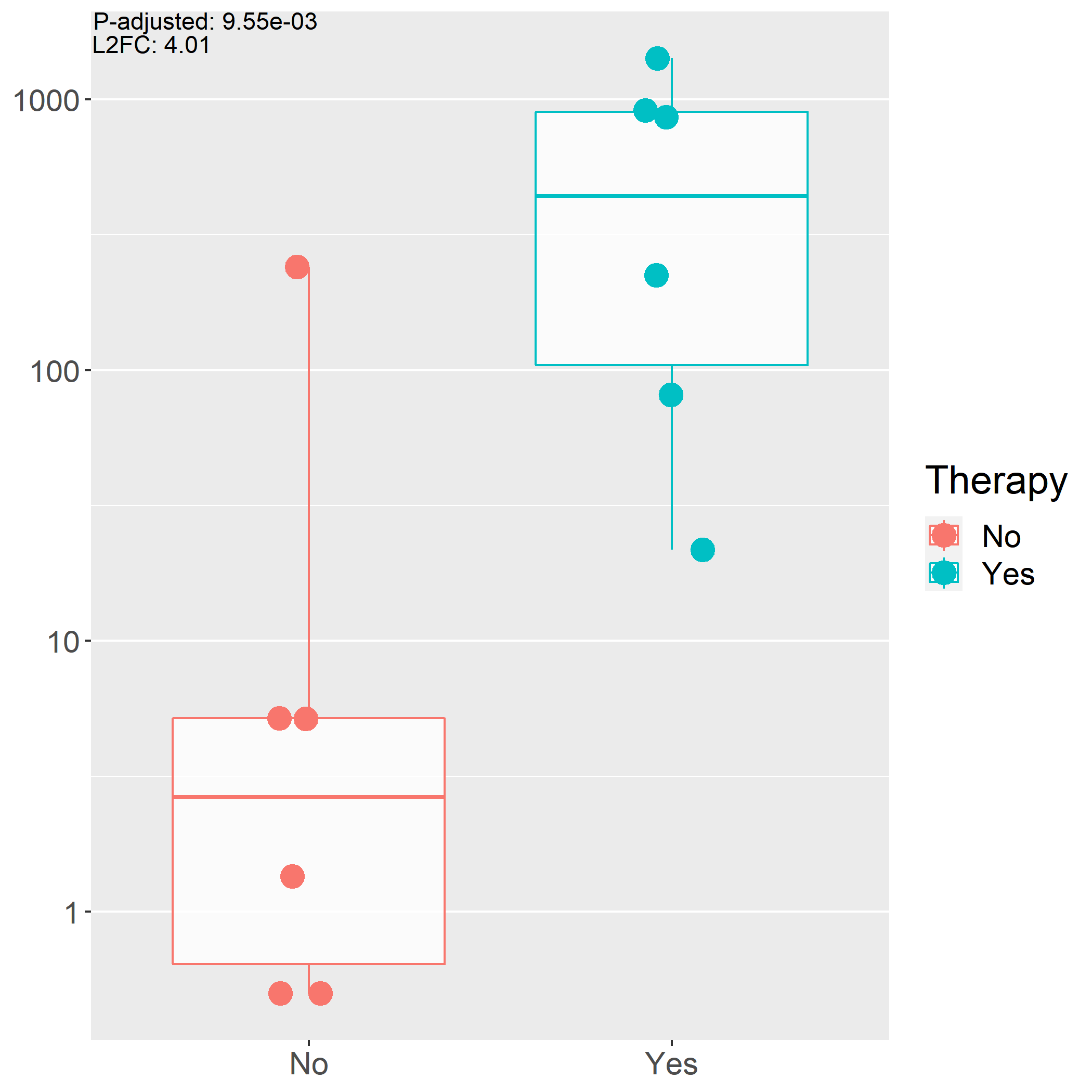 | 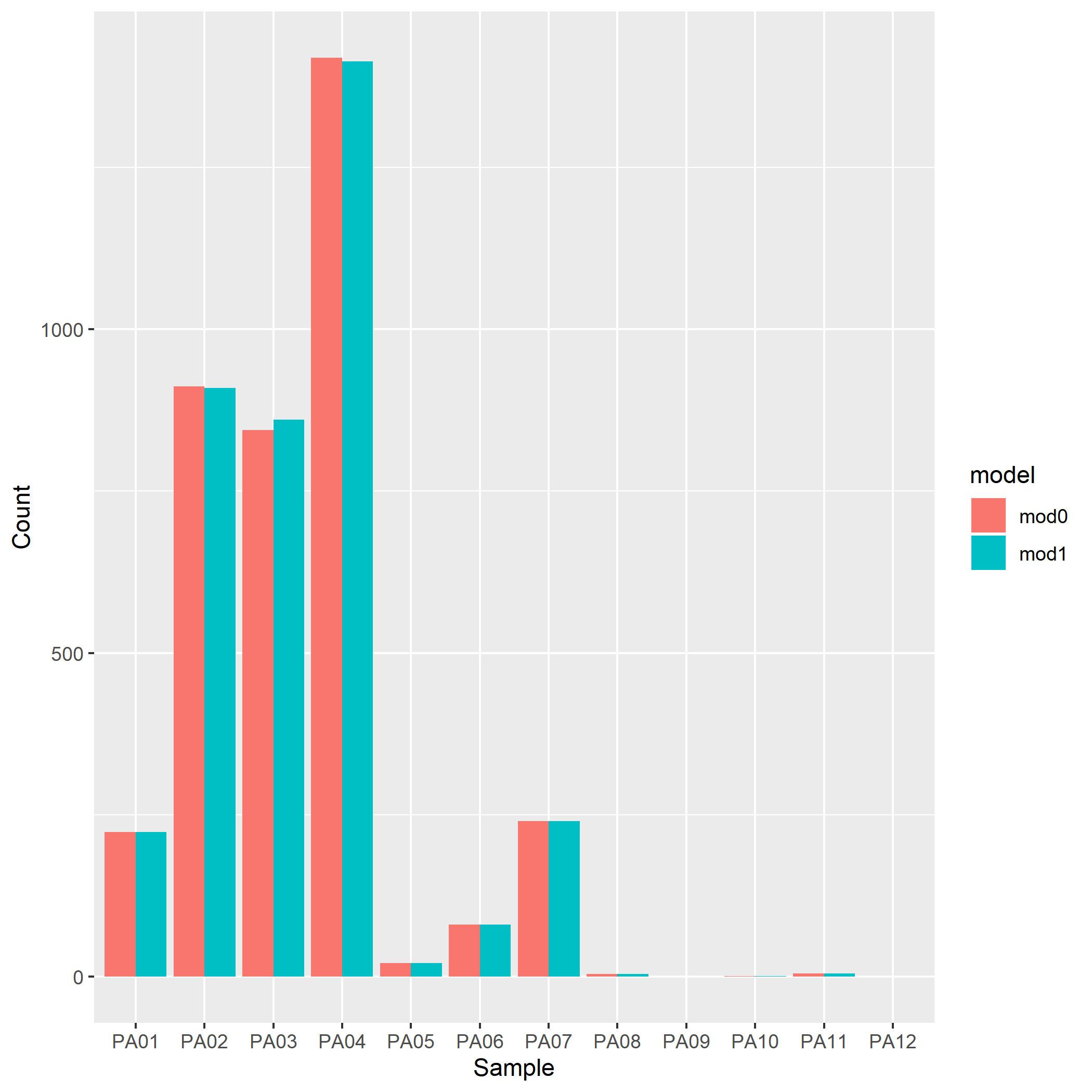 |
| *ADAMTSL2* | ADAMTS Like 2 | 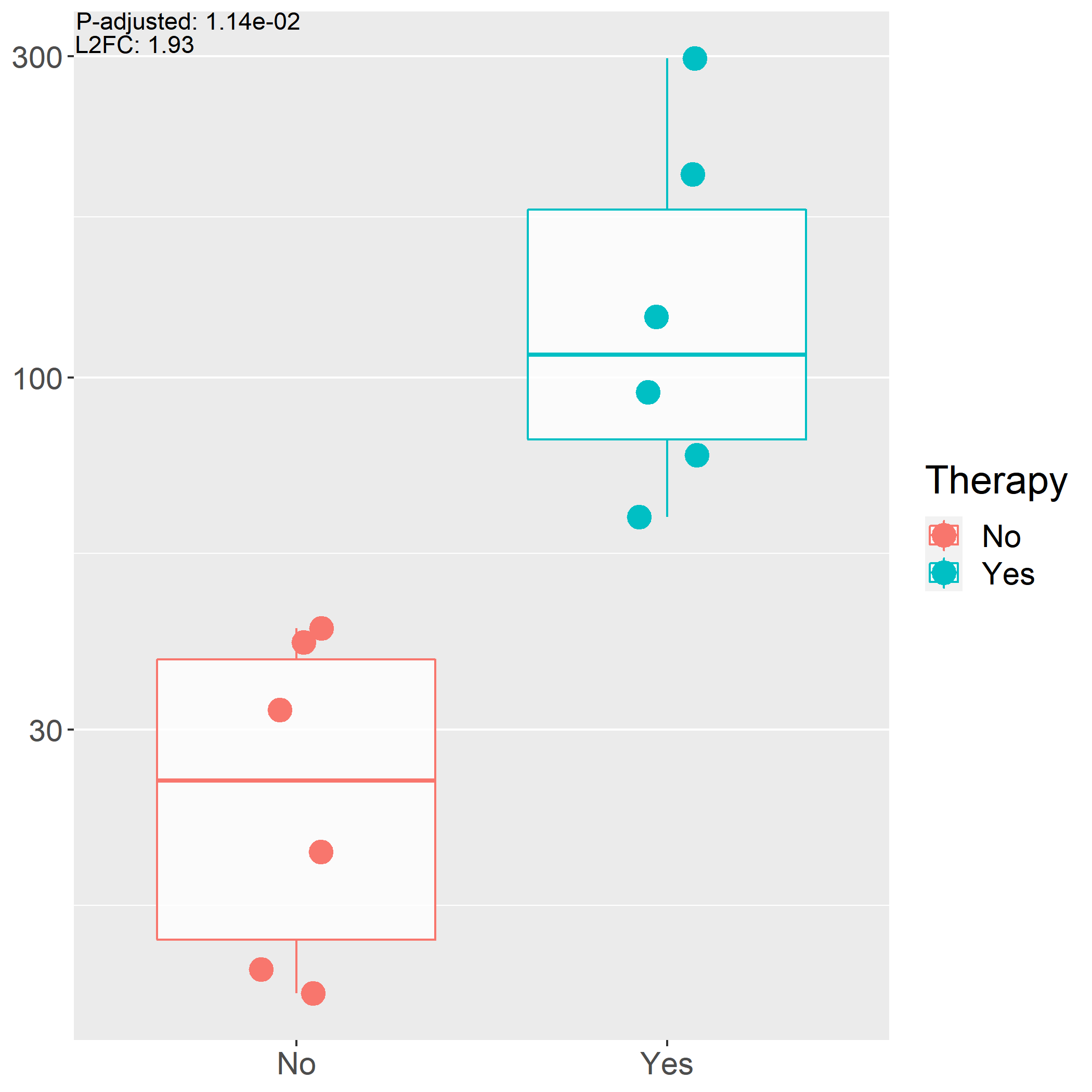 | 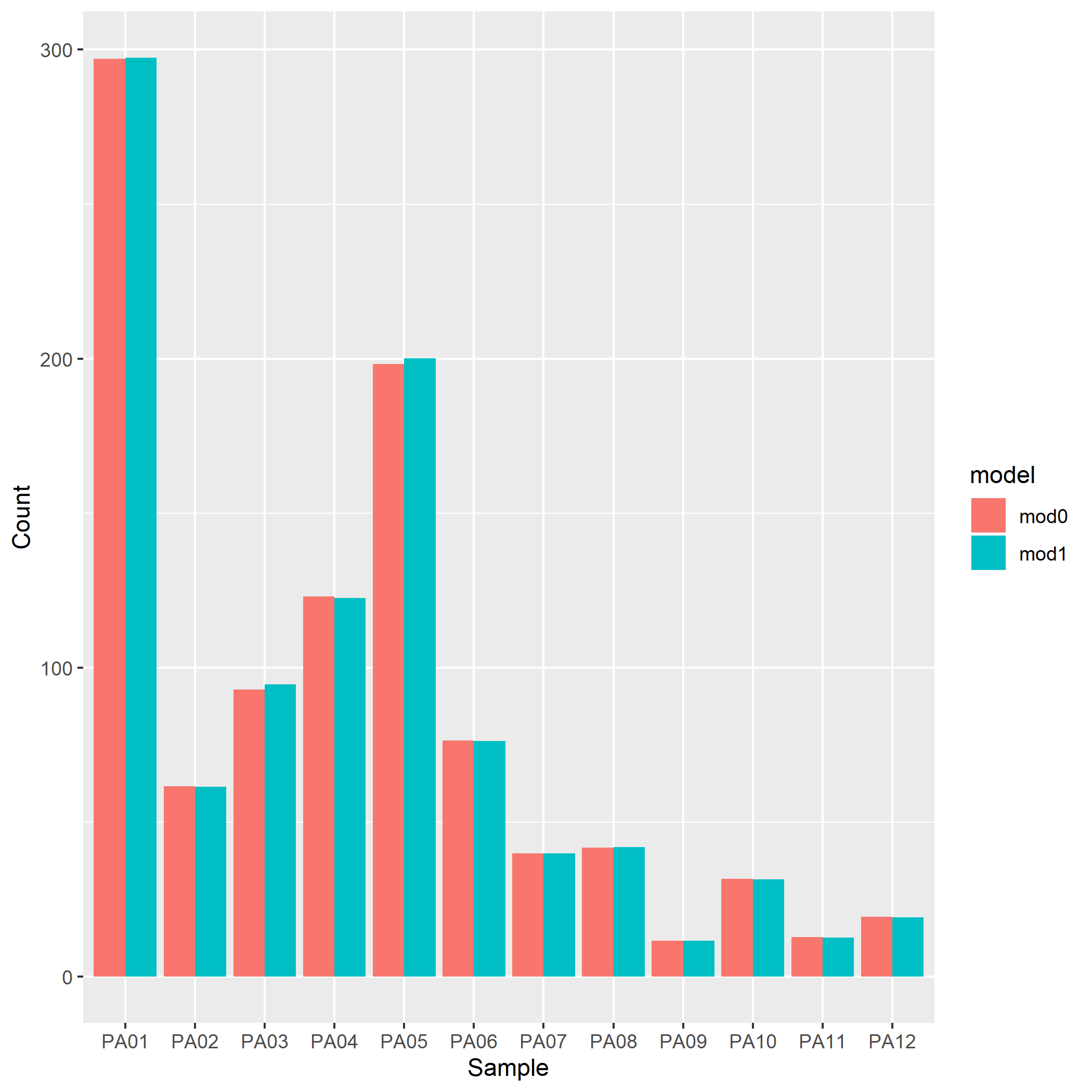 |
| *PDE6A* | Phosphodiesterase 6A | 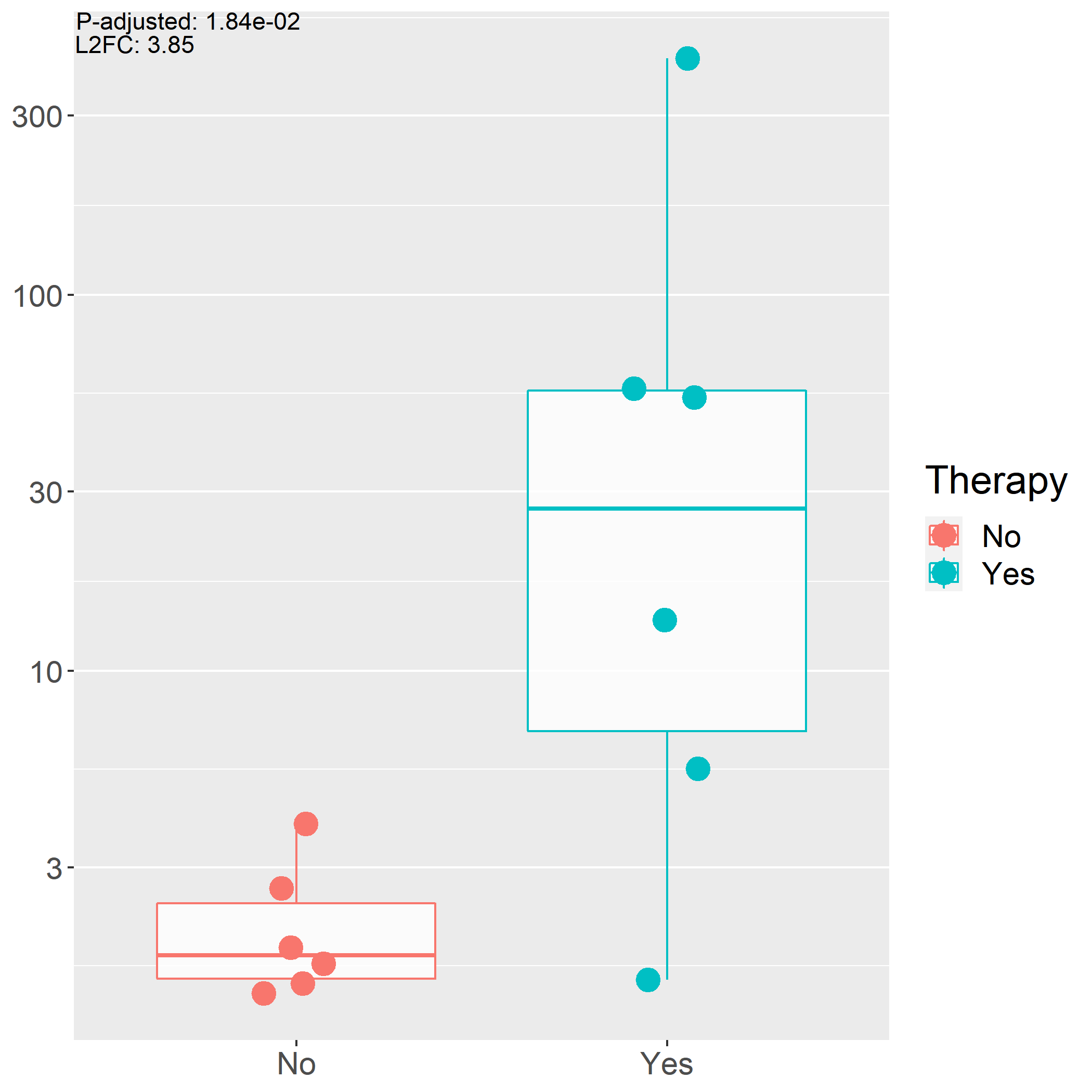 | 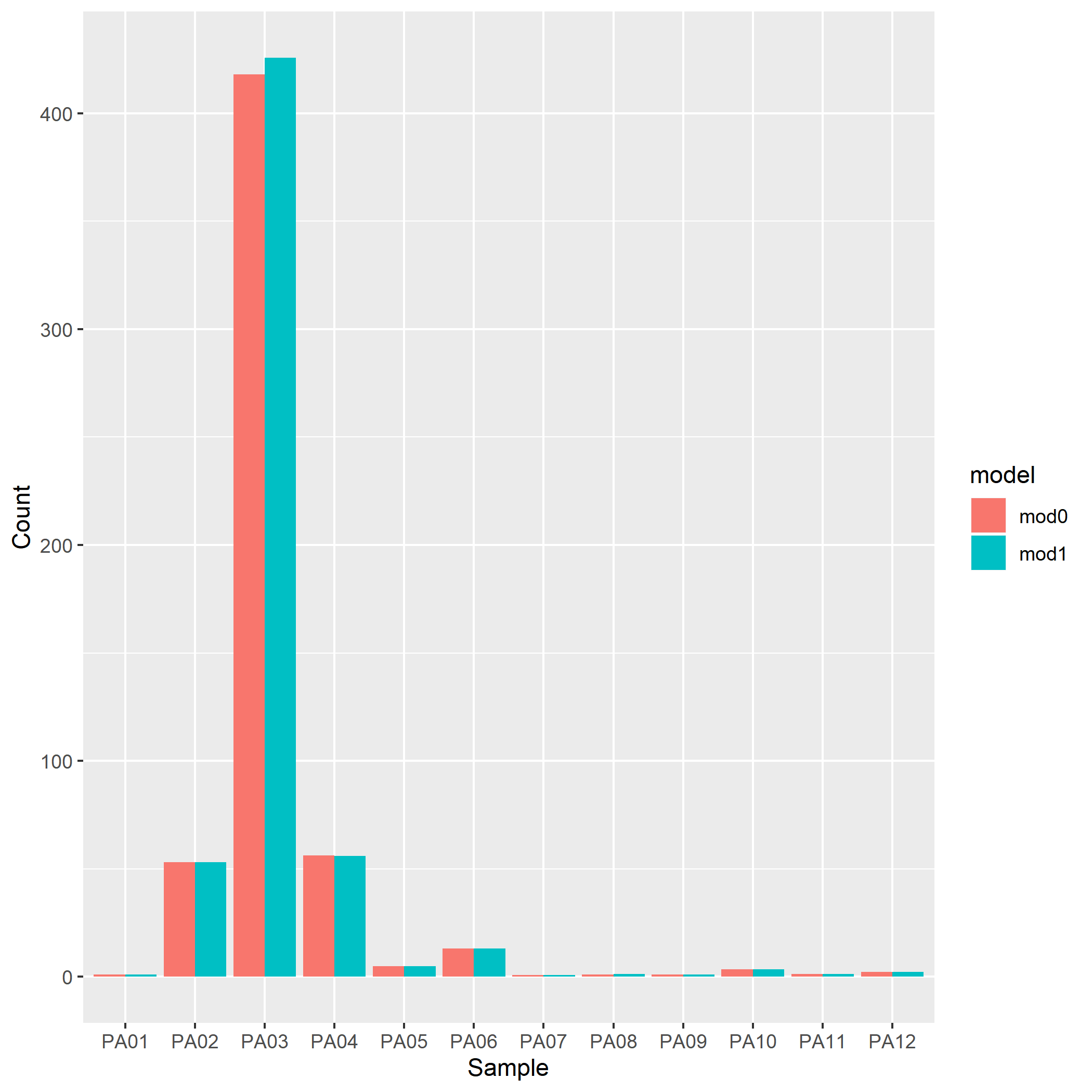 |
| *SLC8A2* | Solute Carrier Family 8 Member A2 | 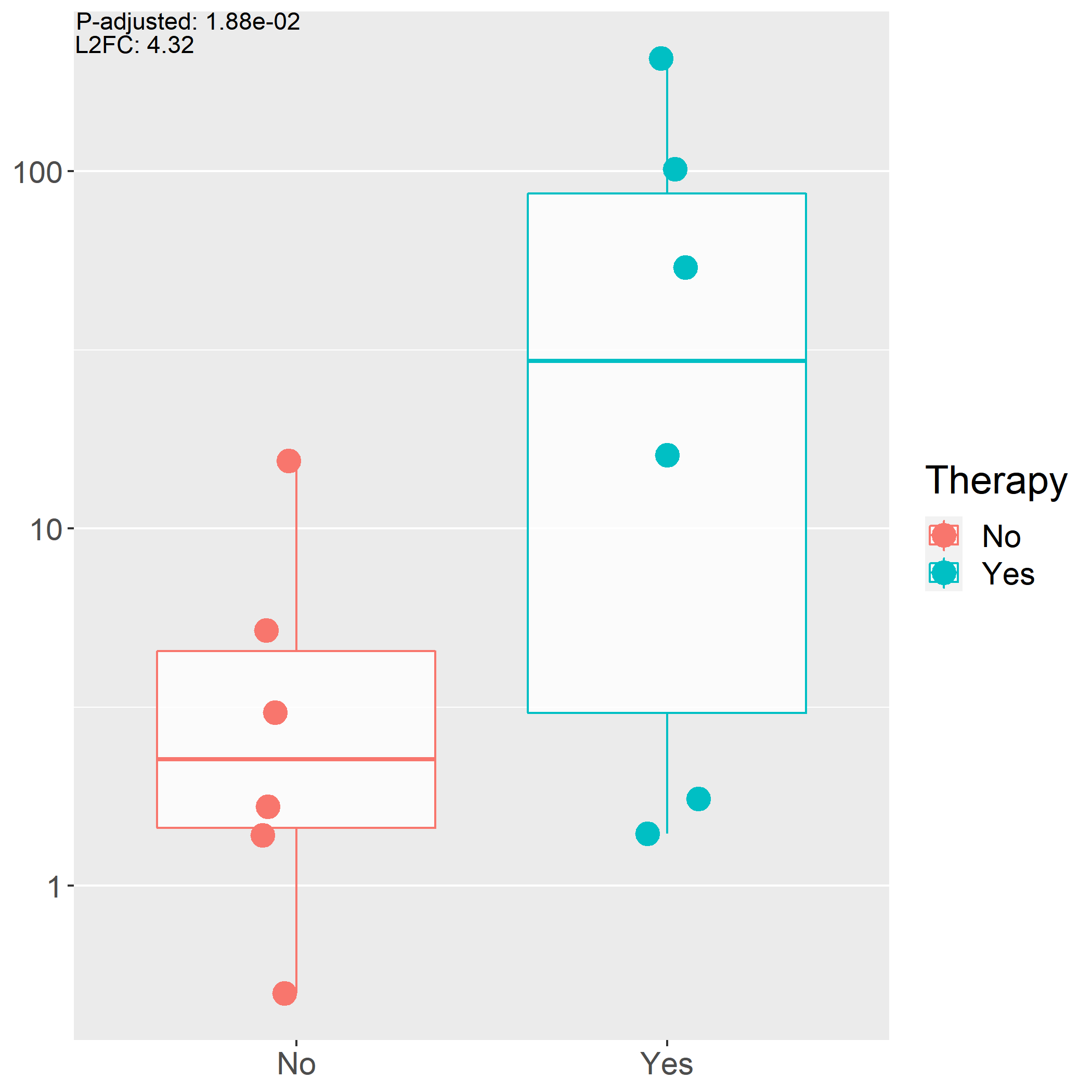 | 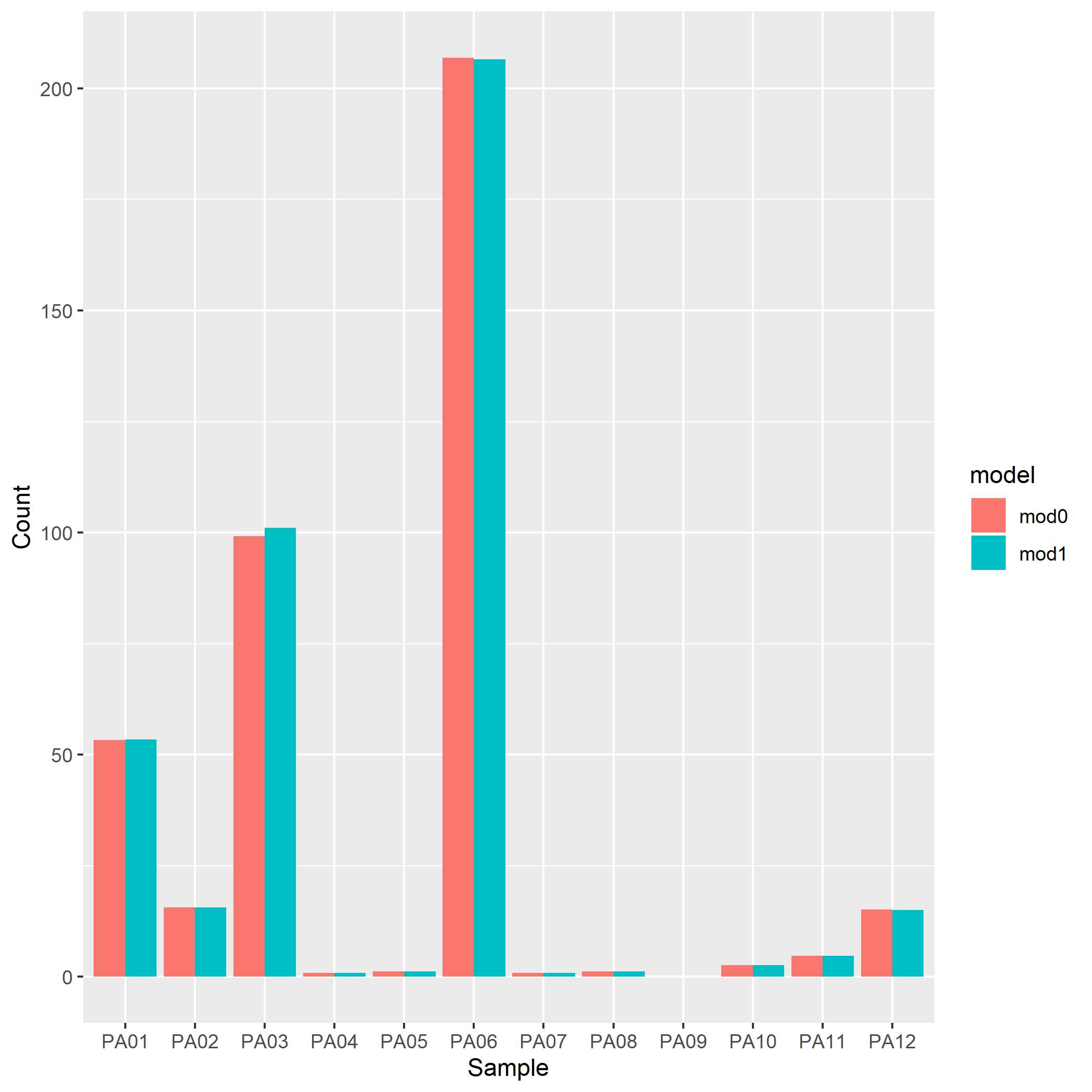 |
| *PCP4L1* | Purkinje Cell Protein 4 Like 1 | 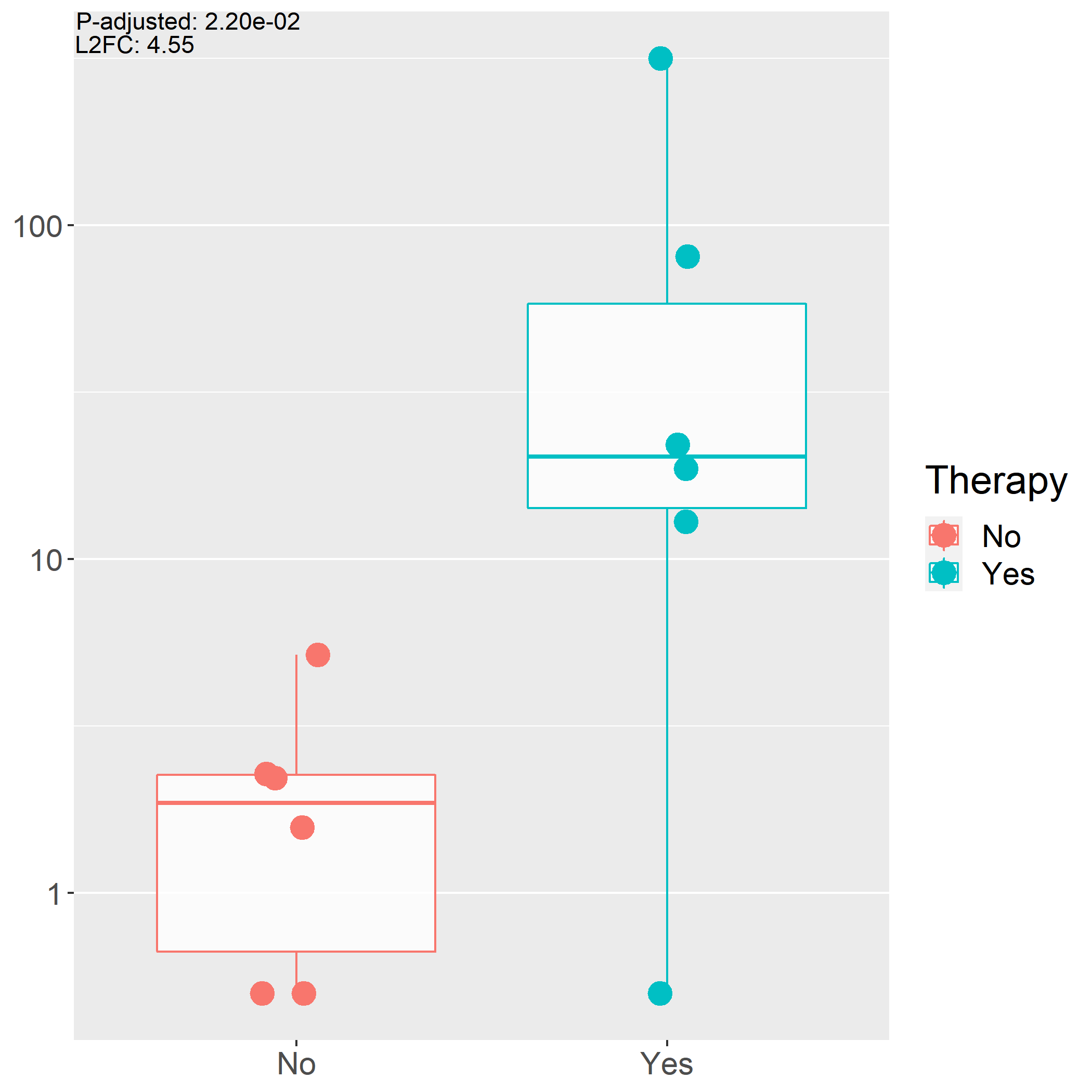 | 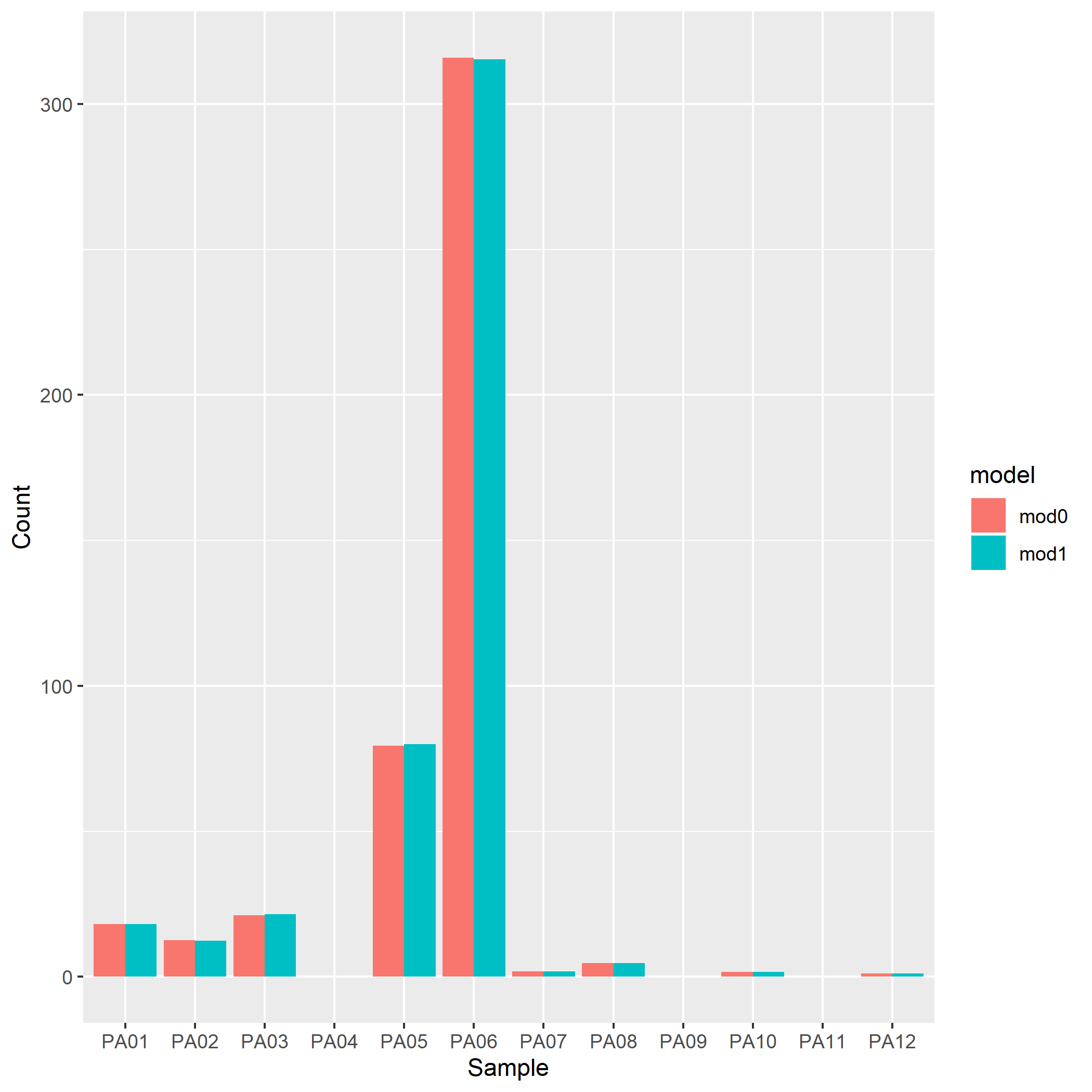 |
| *DIRAS2* | DIRAS Family GTPase 2 | 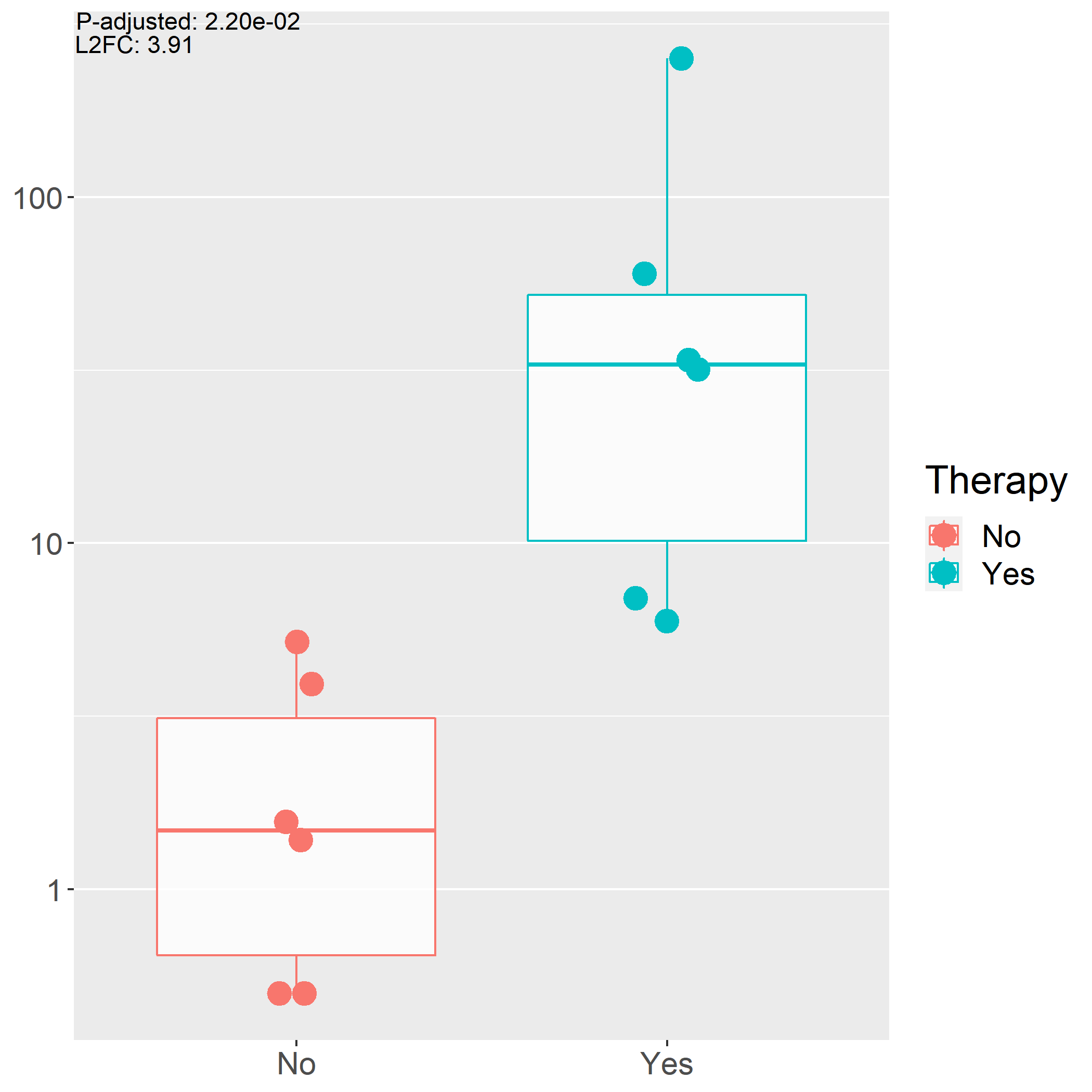 | 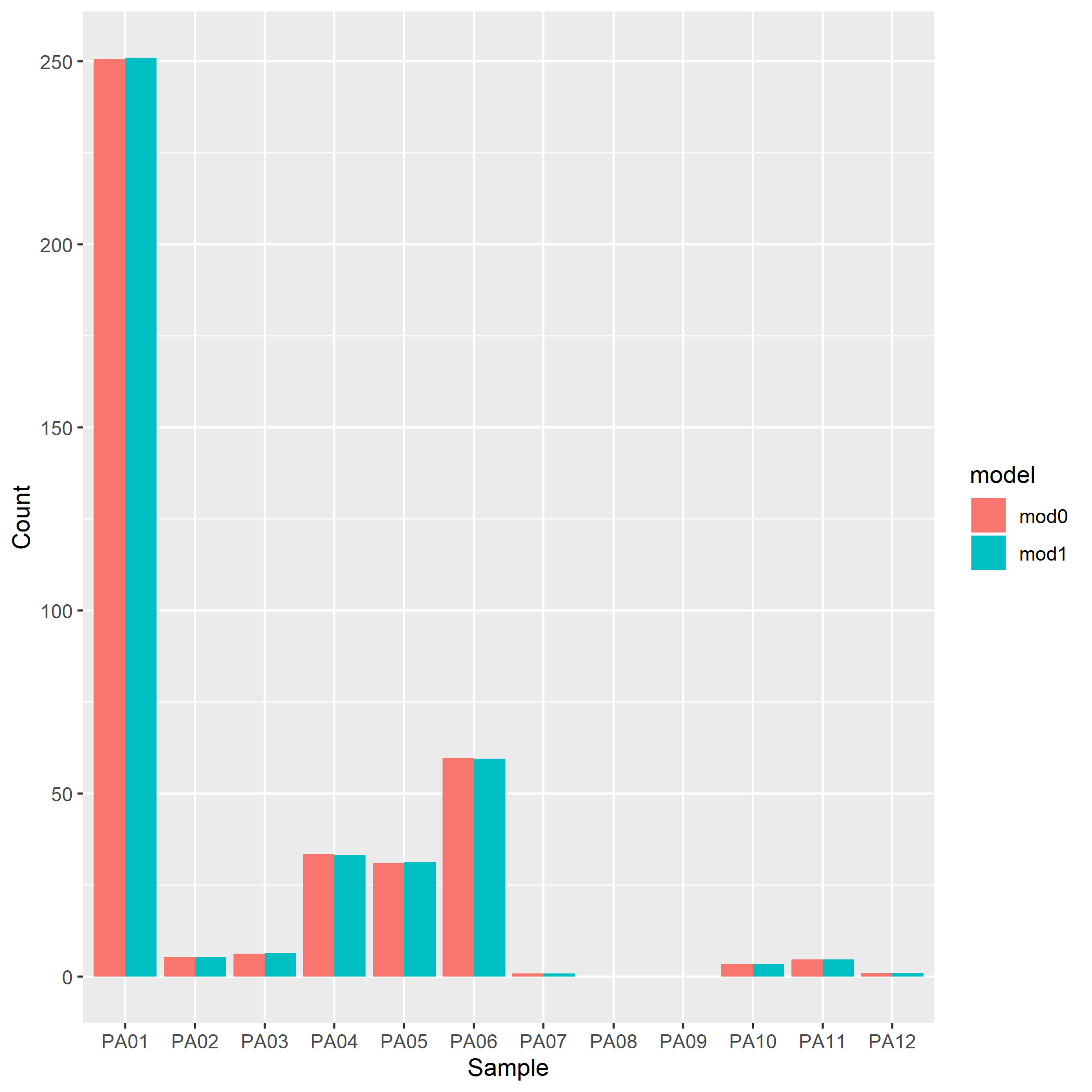 |
| *B4GALNT4* | Beta-1,4-N-Acetyl-Galactosaminyltransferase 4 | 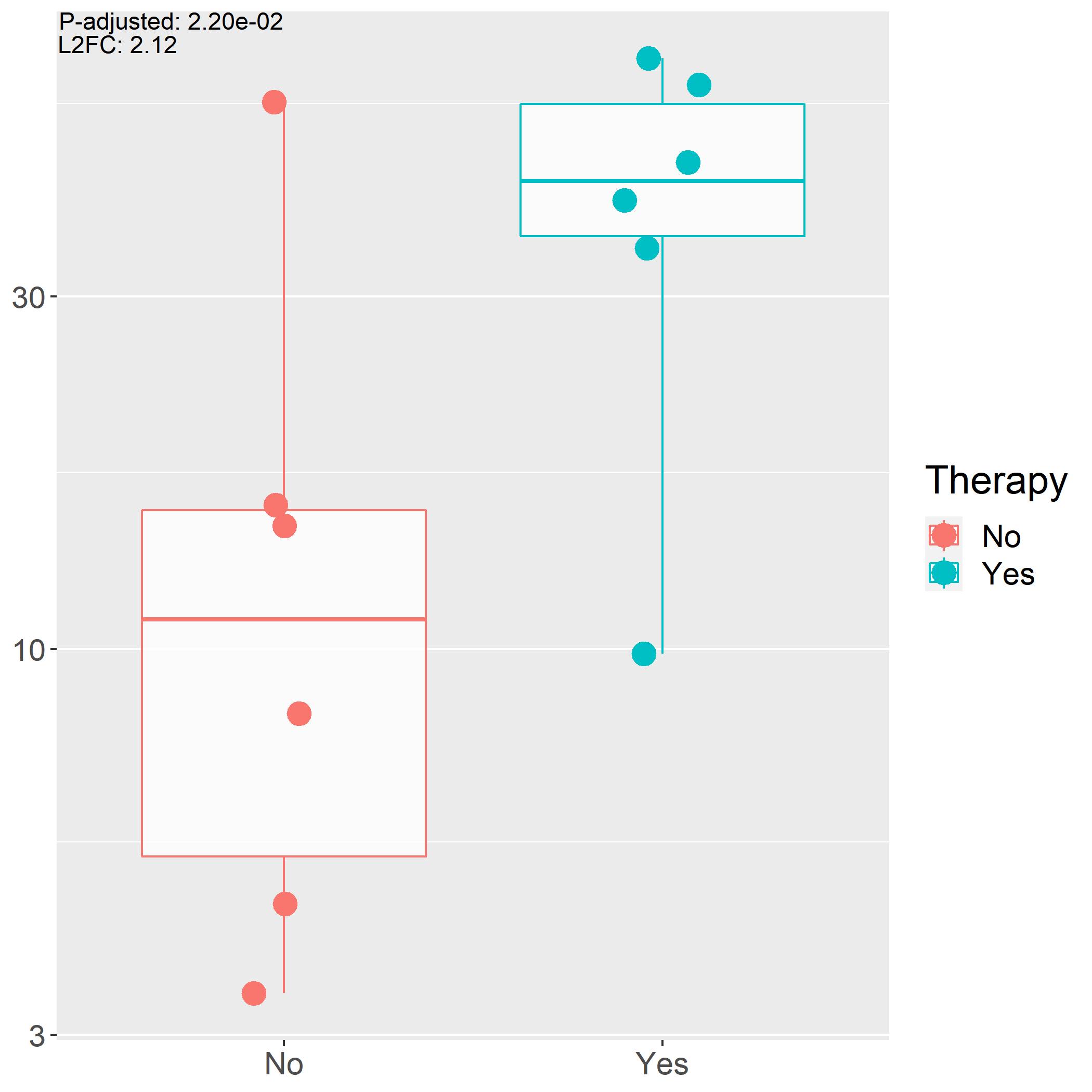 | 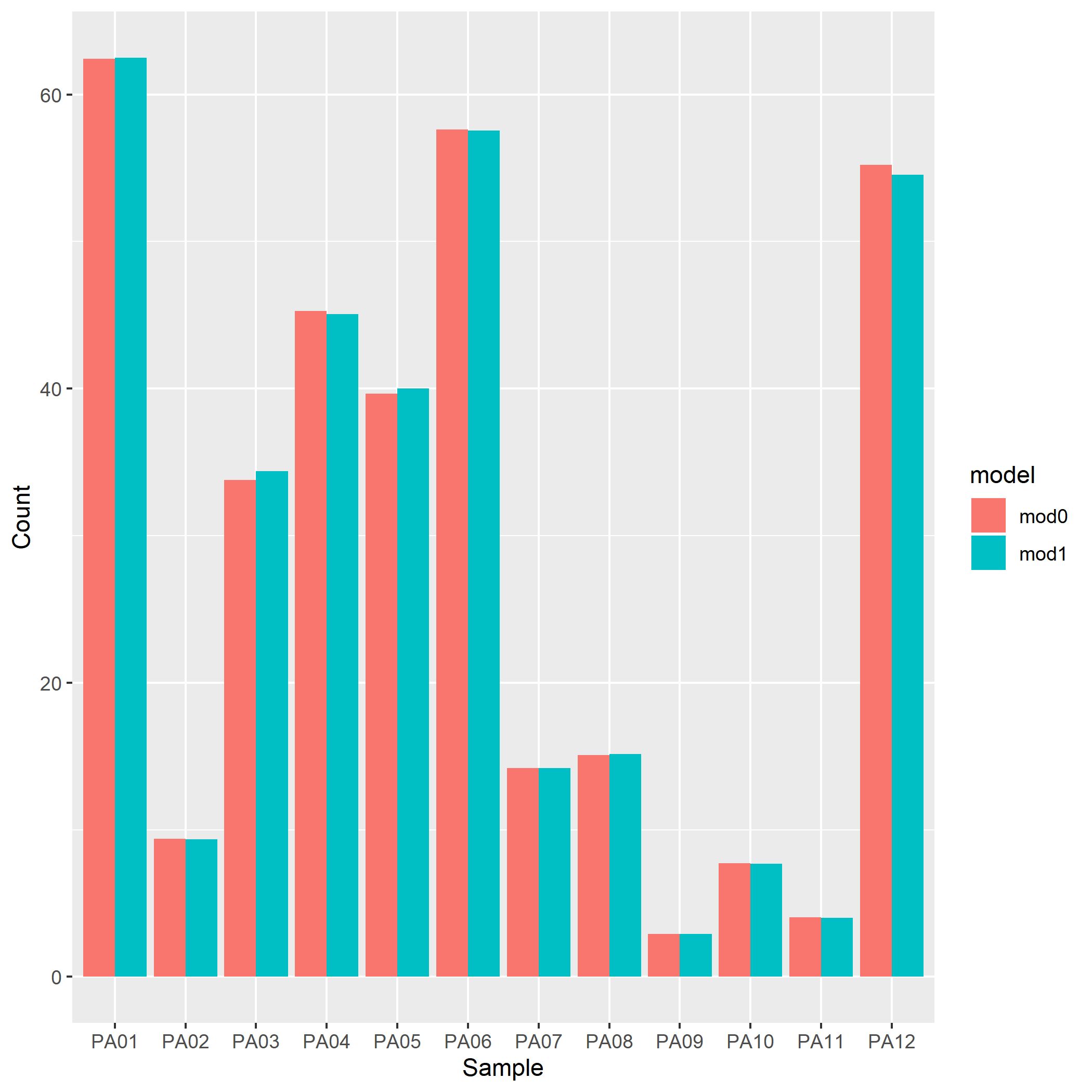 |
| *APBA2* | Amyloid Beta Precursor Protein Binding Family A Member 2 | 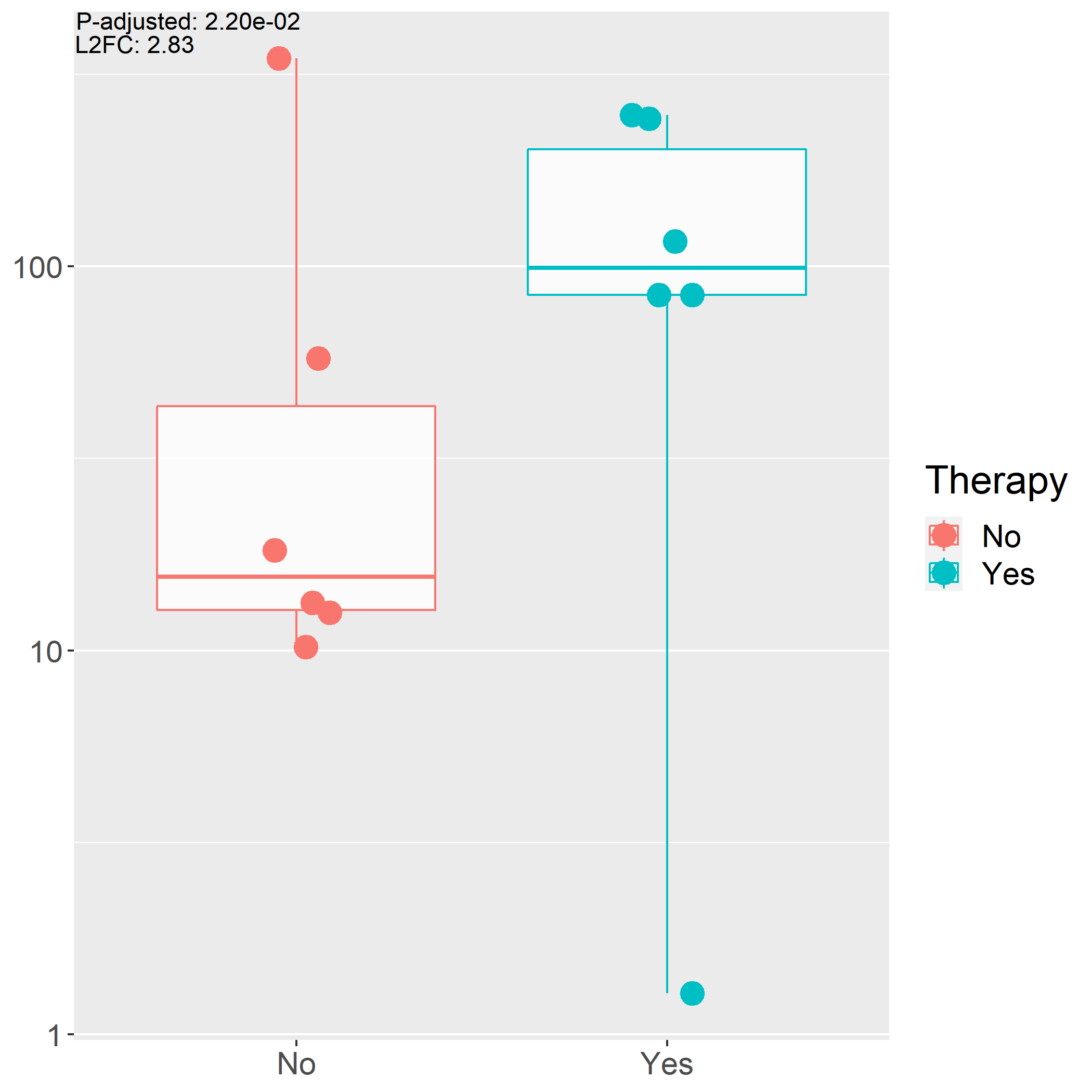 | 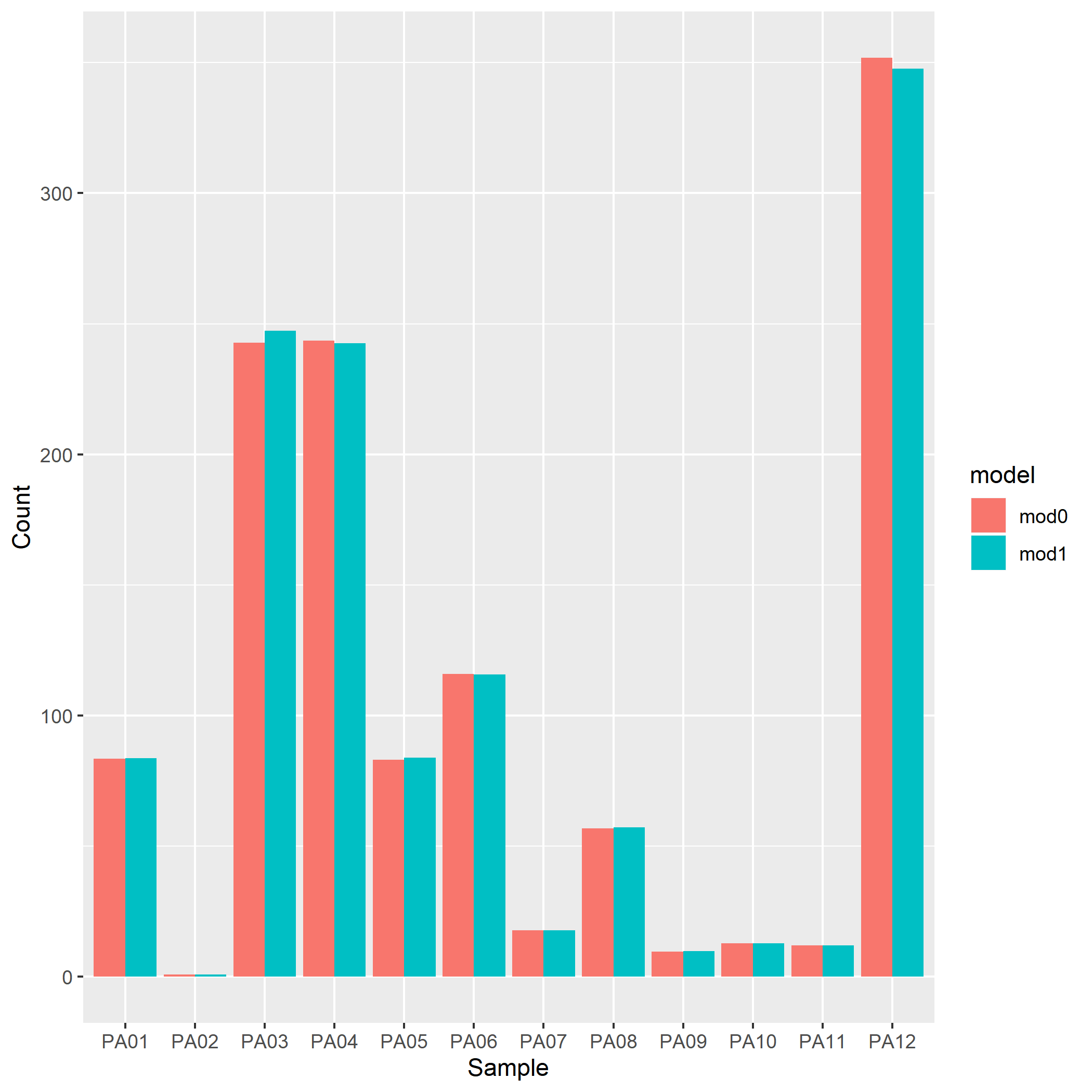 |
| *THBD* | Thrombomodulin | 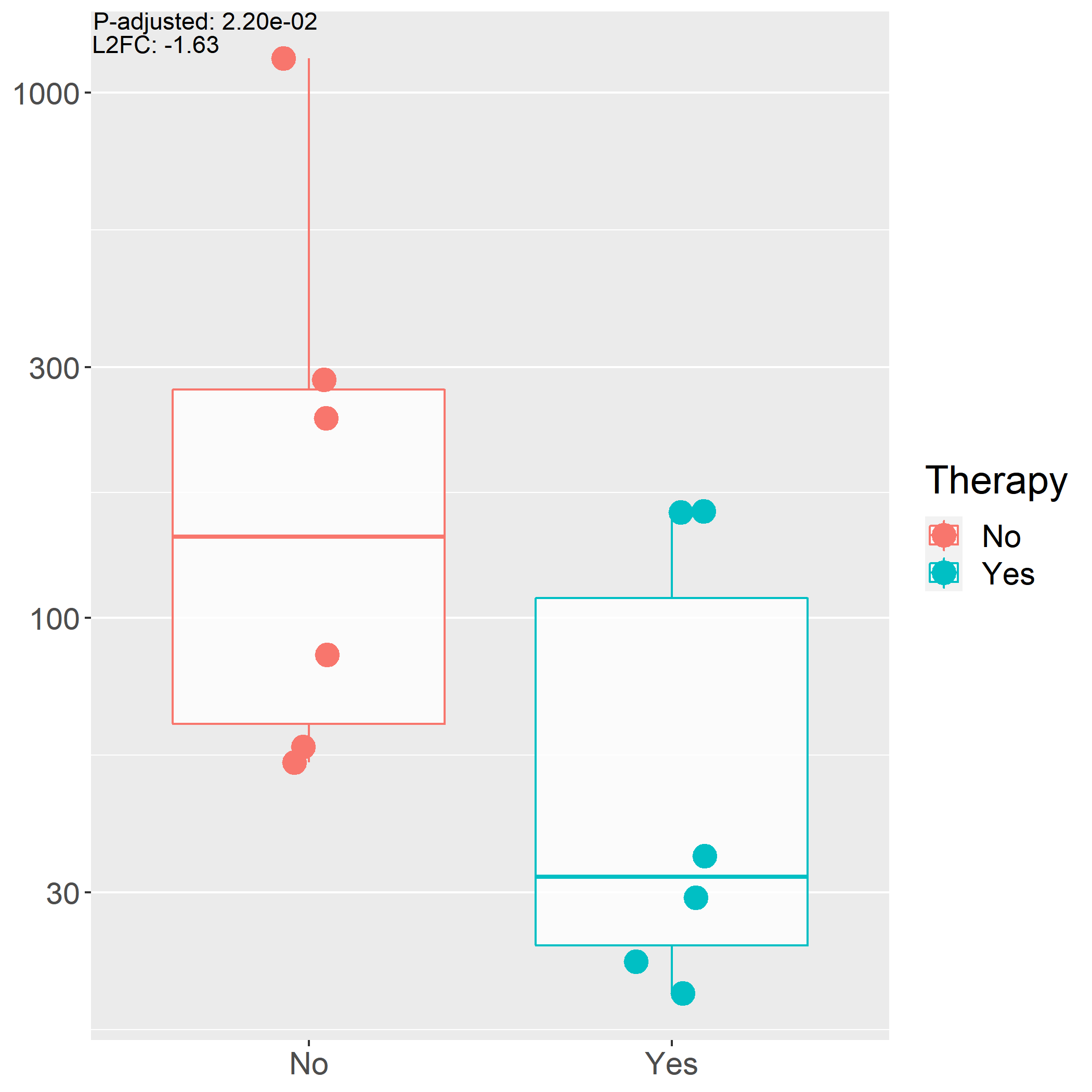 | 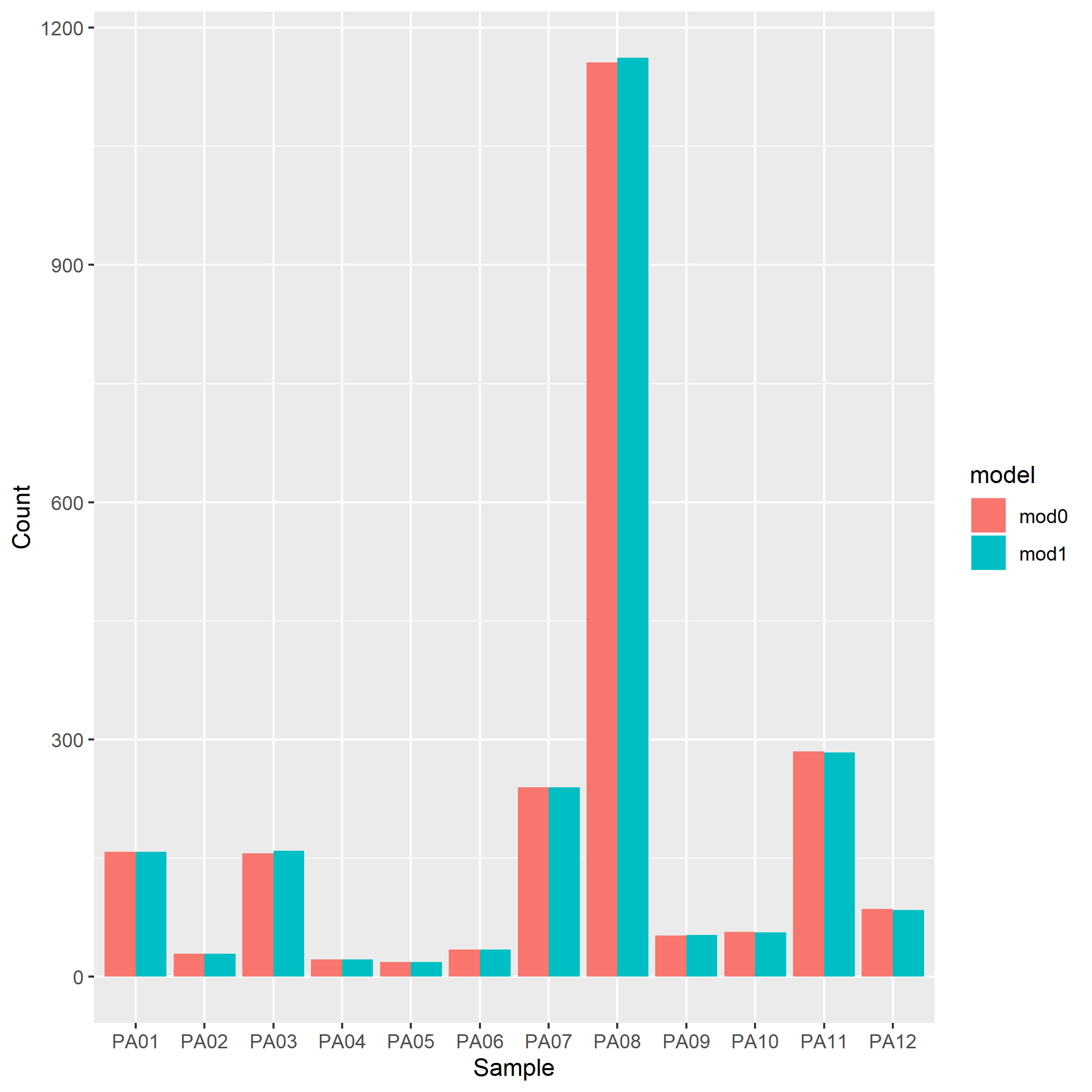 |
| *PTGS2* | Prostaglandin-Endoperoxide Synthase 2 | 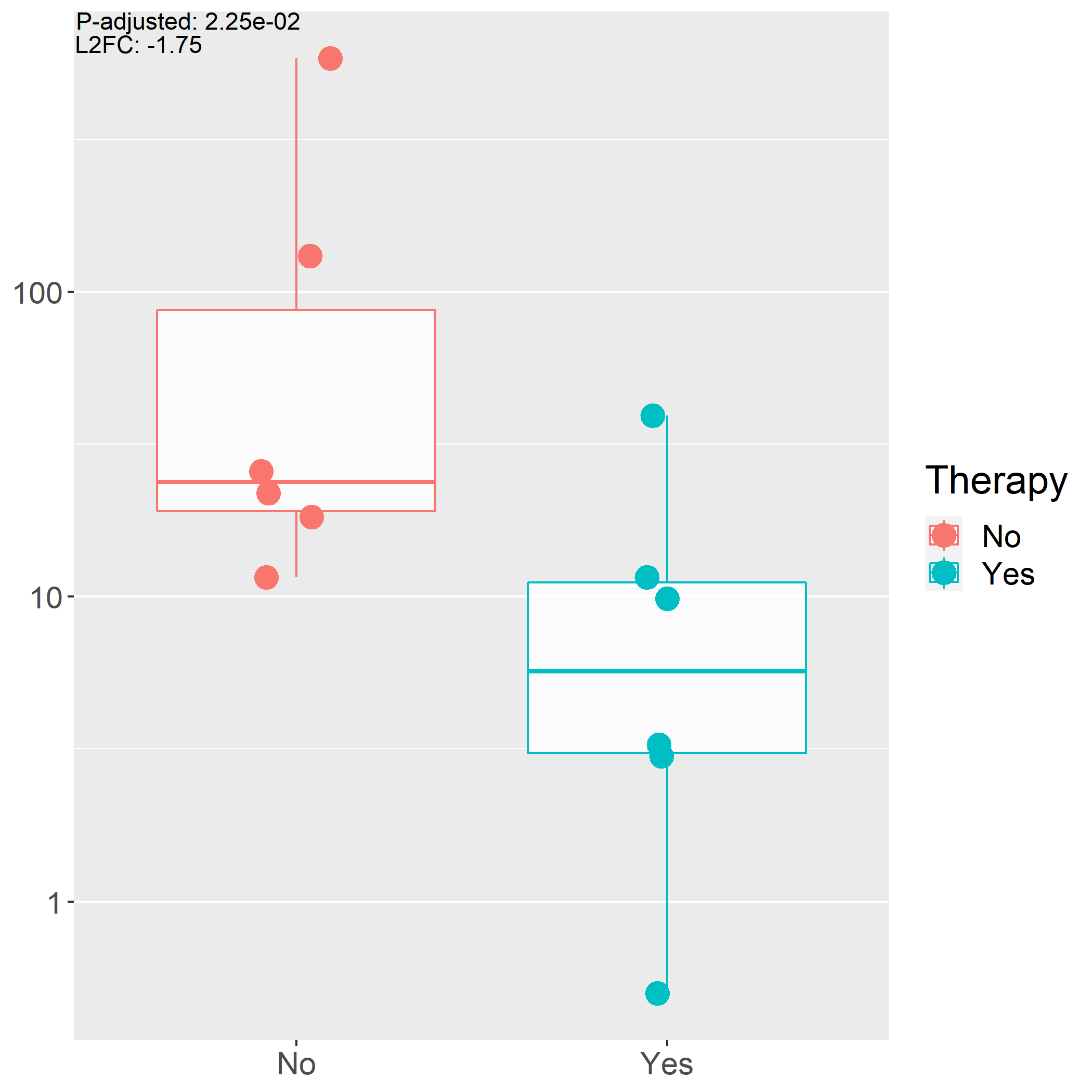 | 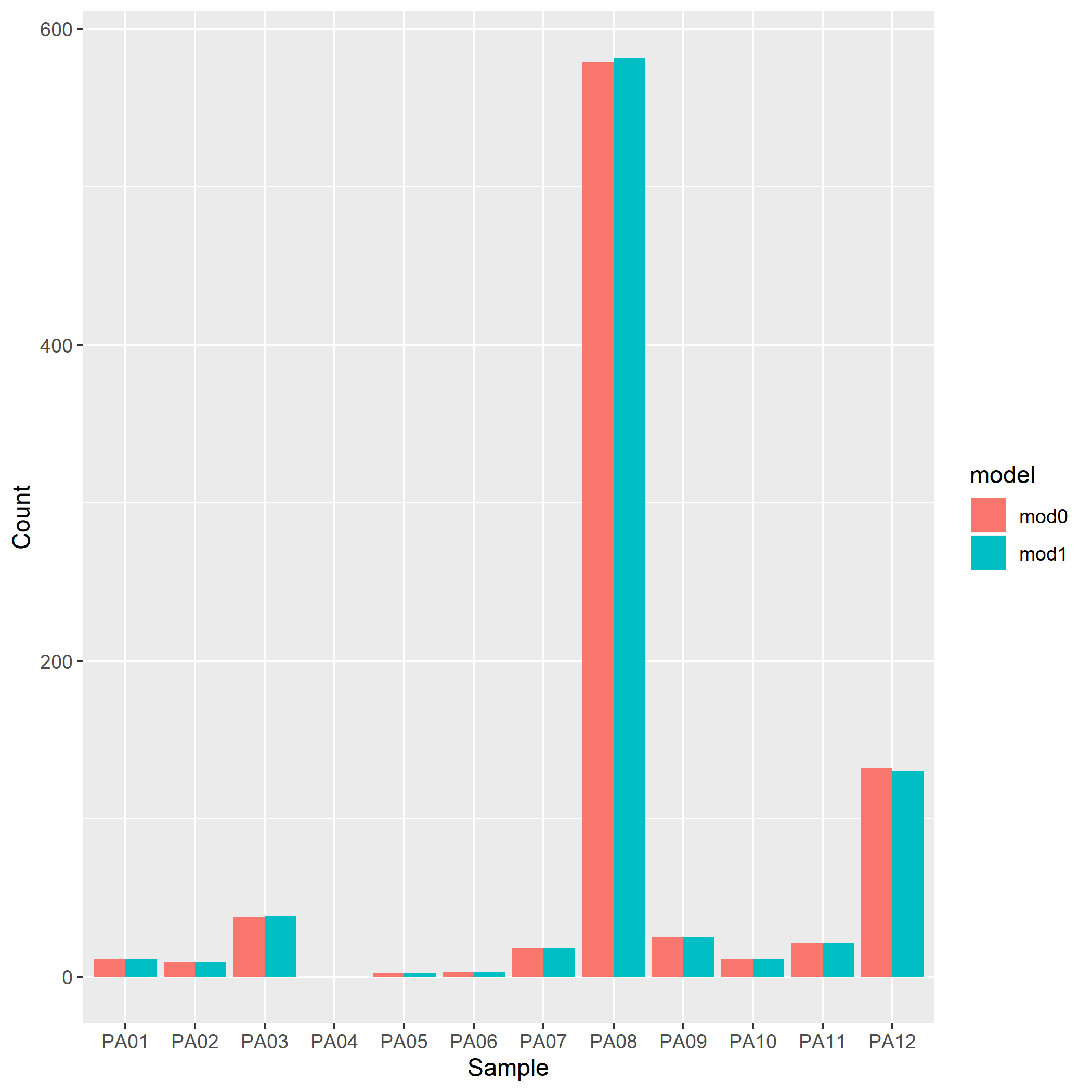 |
| *SV2B* | Synaptic Vesicle Glycoprotein 2B | 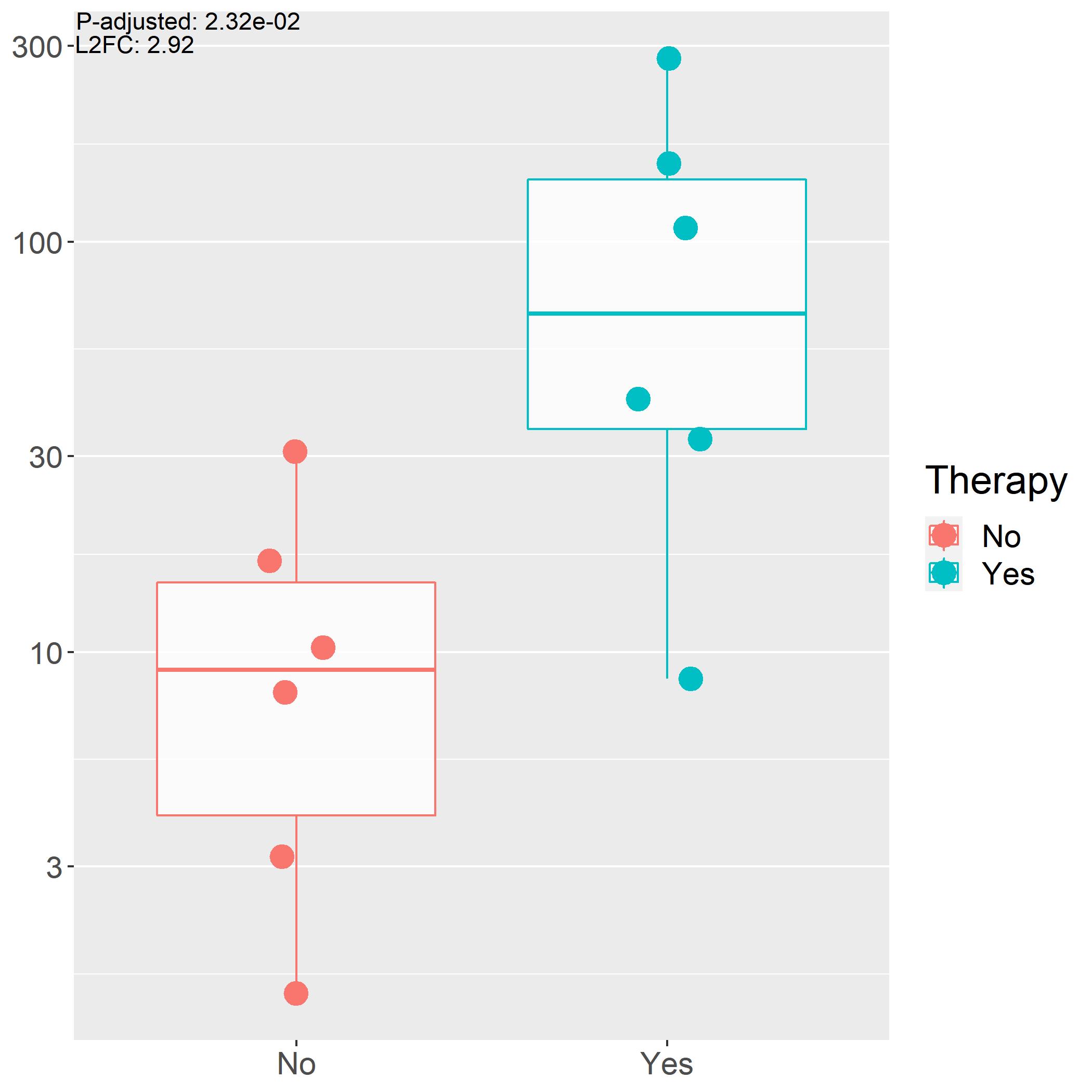 | 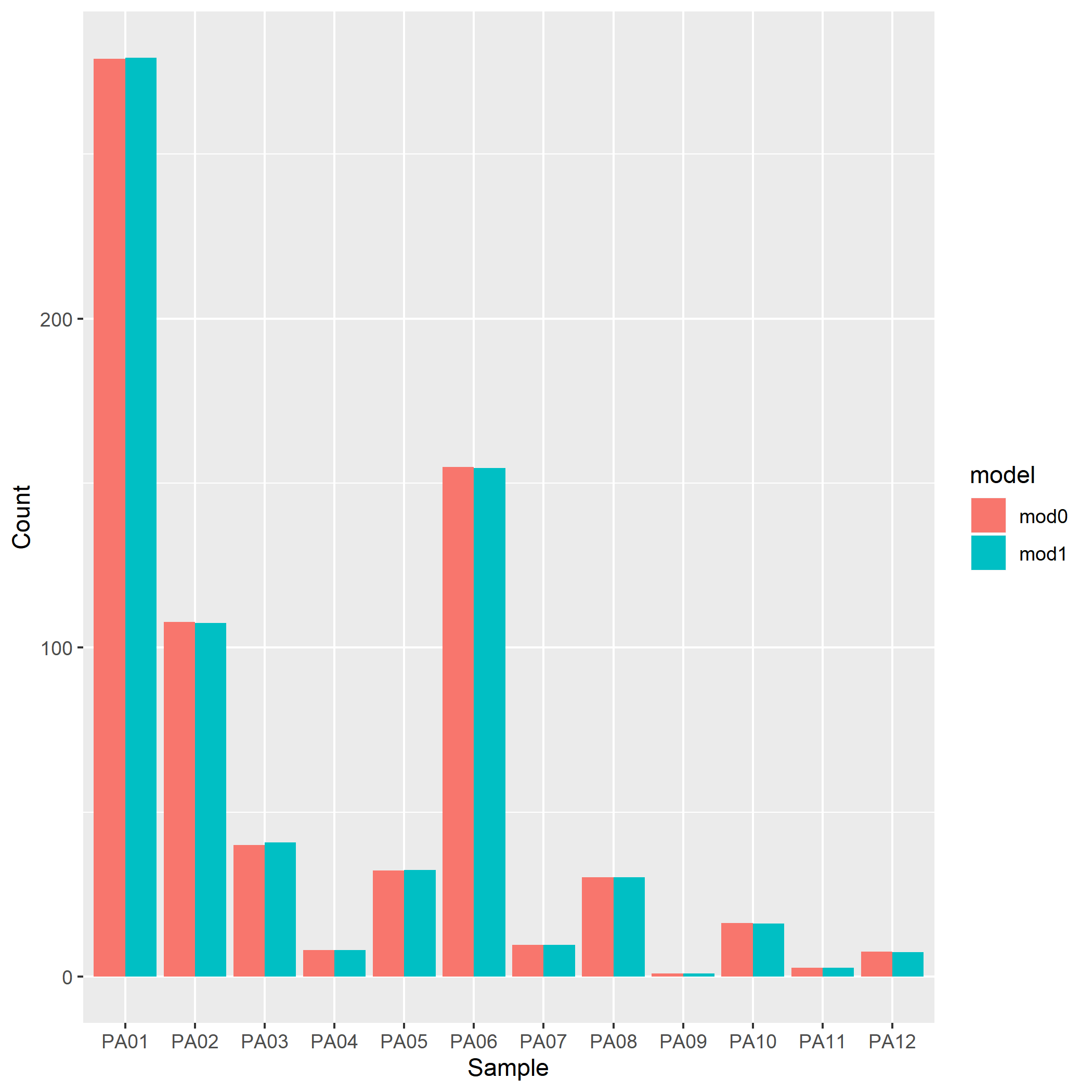 |
| *LINC01529* | Long Intergenic Non-Protein Coding RNA 1529 | 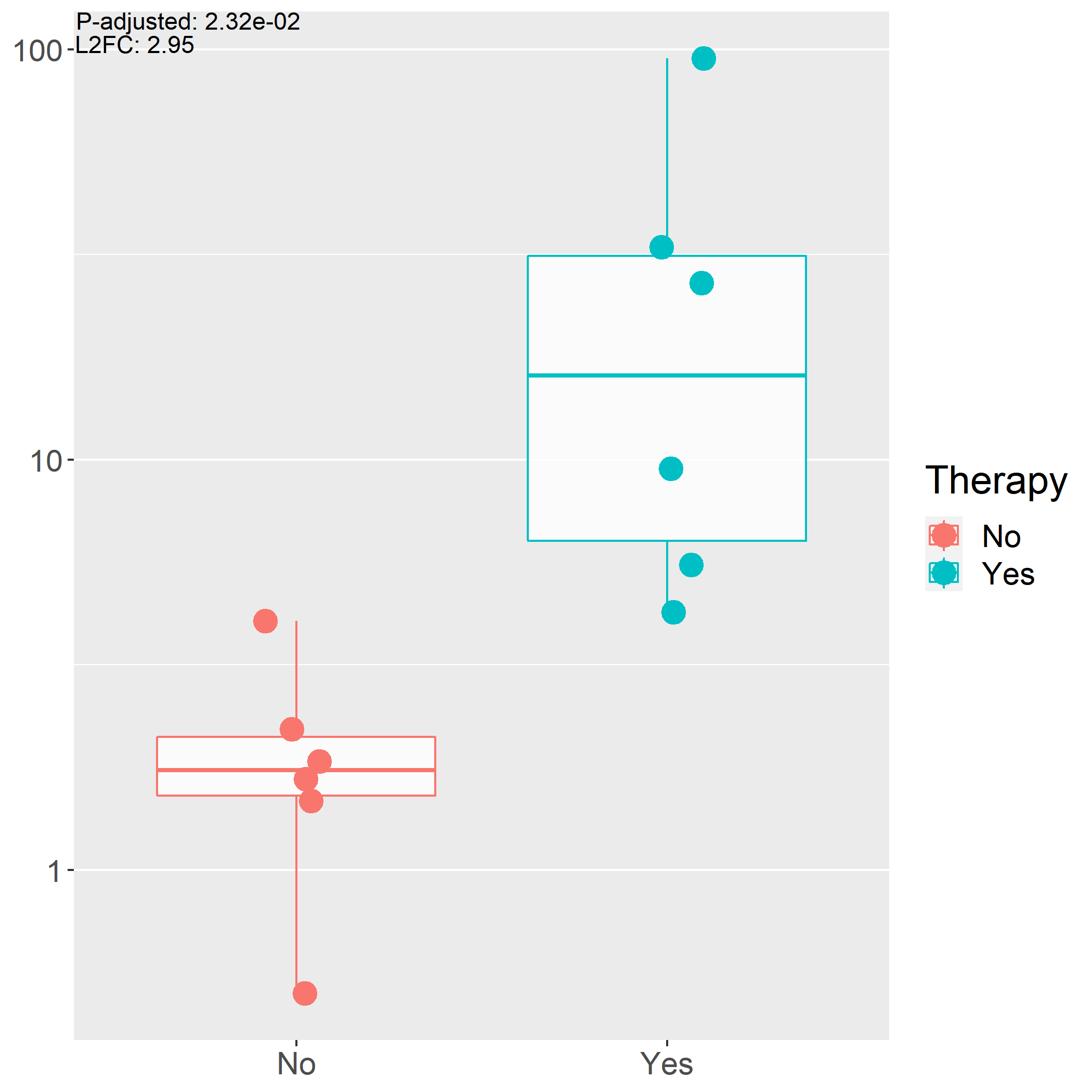 | 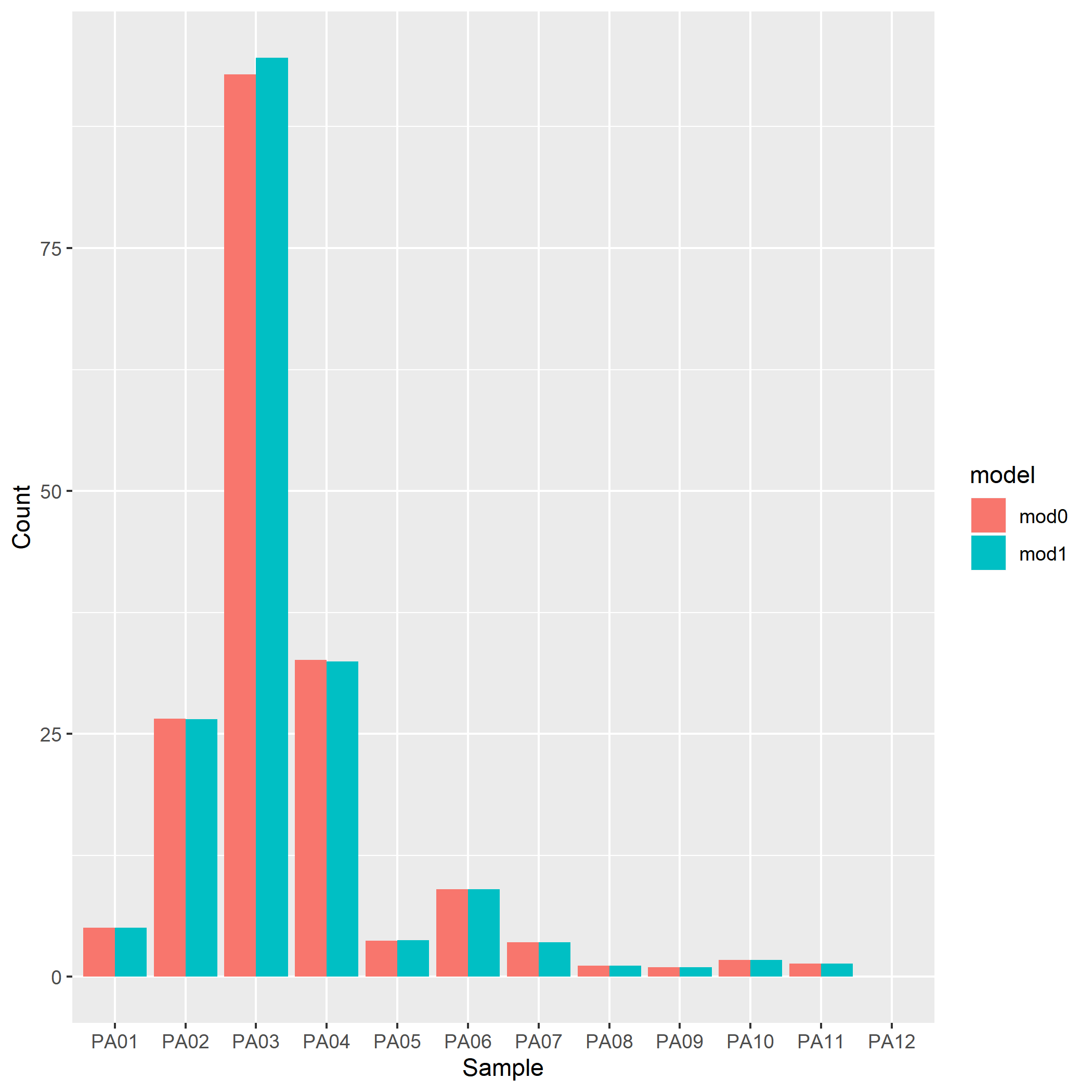 |
| *ST6GALNAC5* | ST6 N-Acetylgalactosaminide Alpha-2,6-Sialyltransferase 5 | 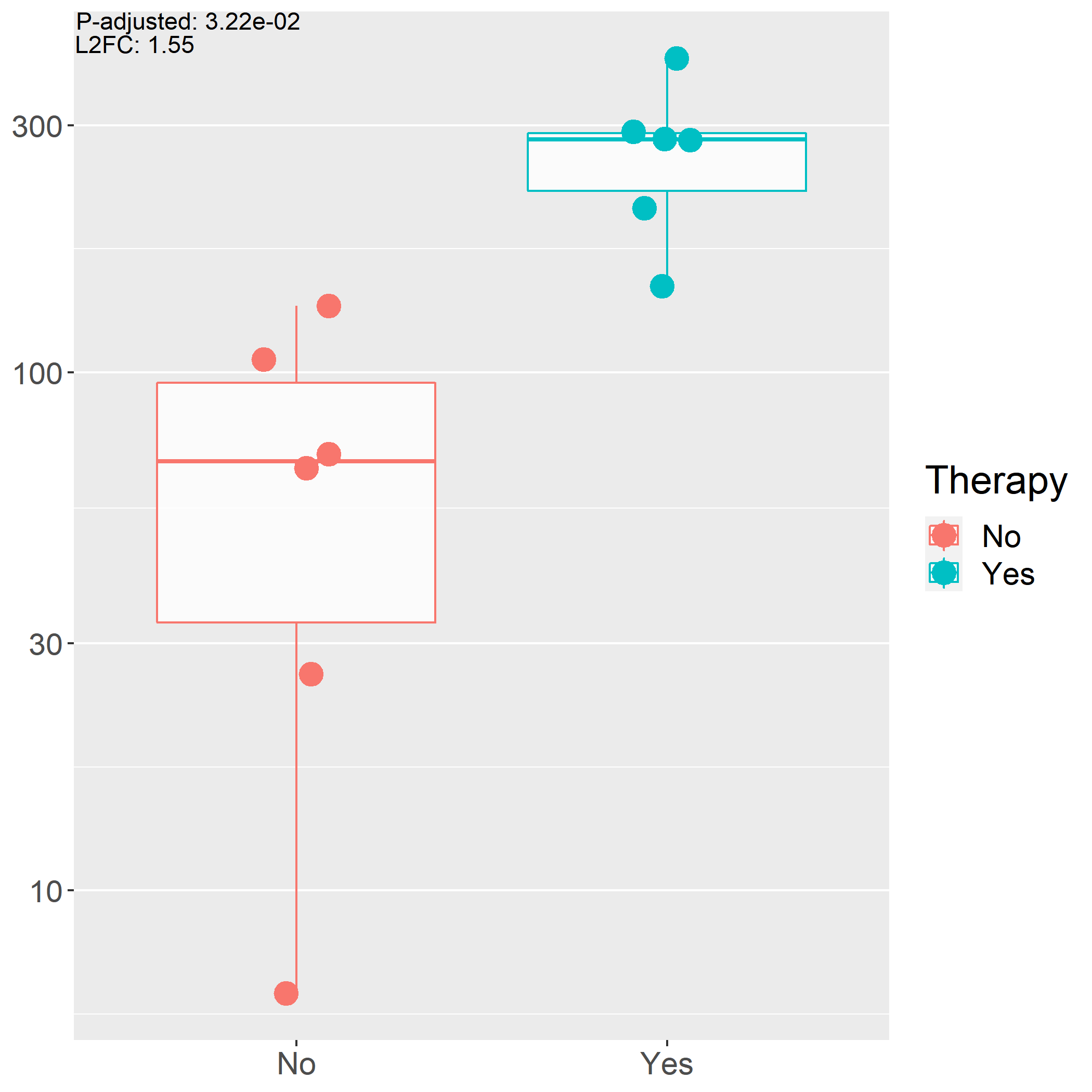 | 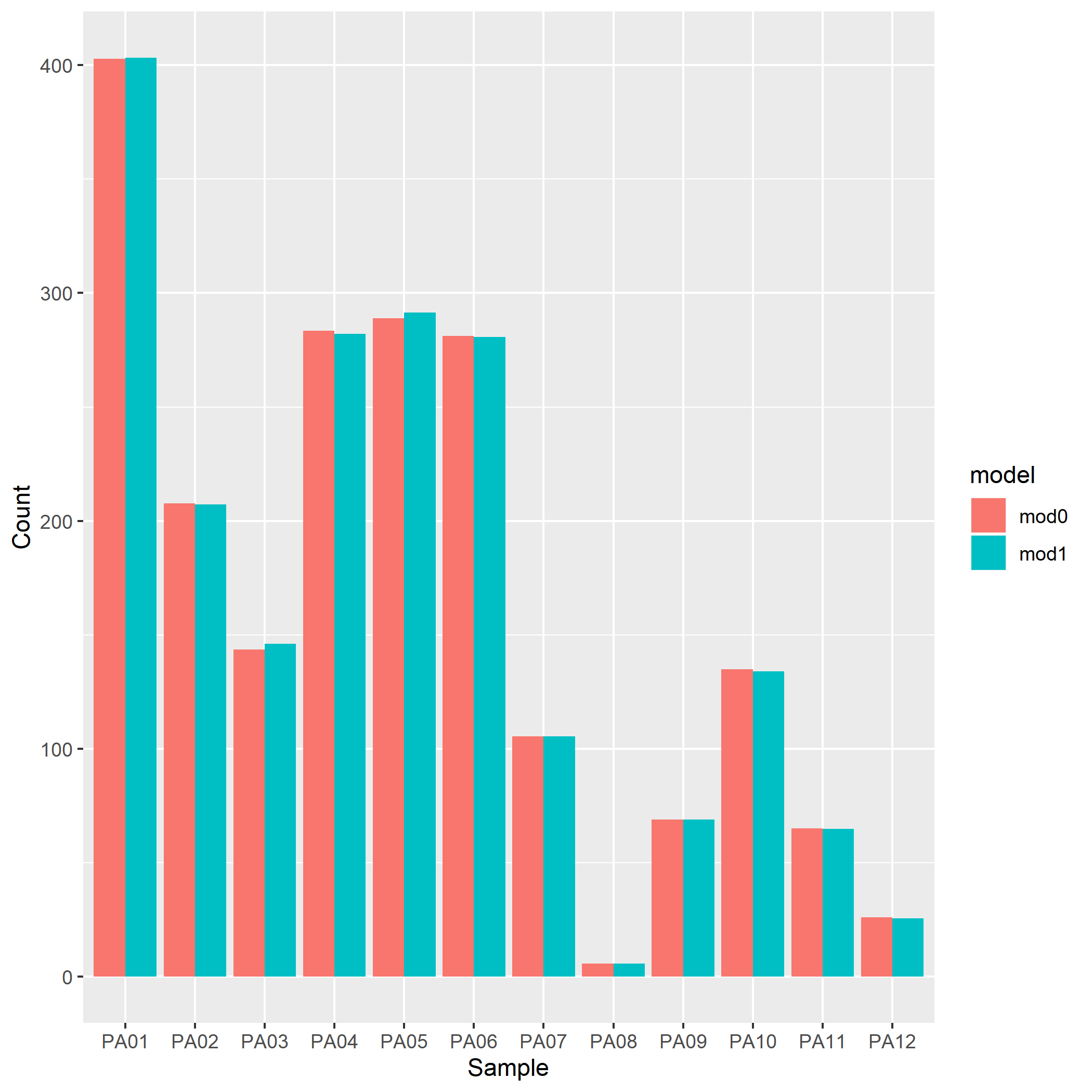 |
| *CALB2* | Calbindin 2 | 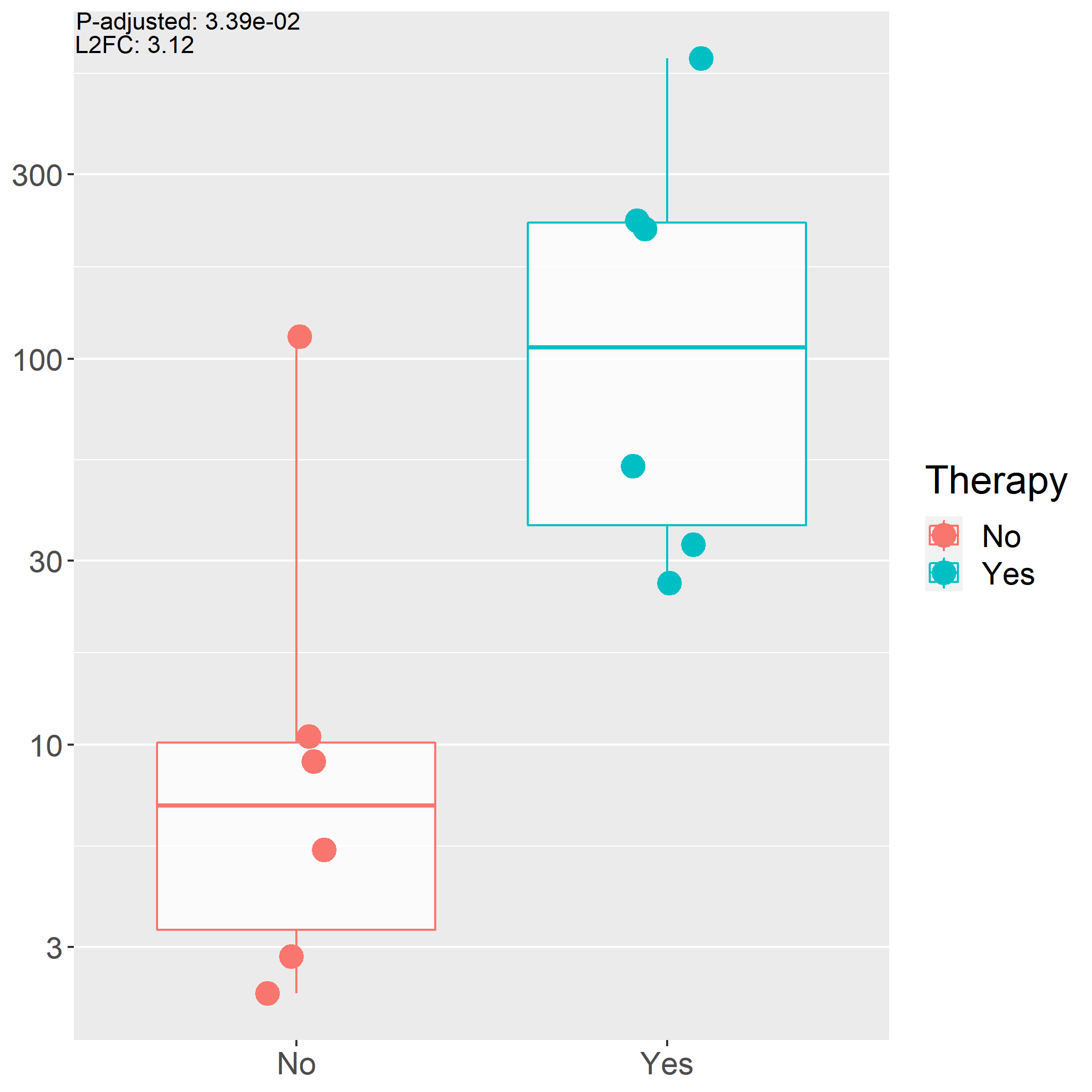 | 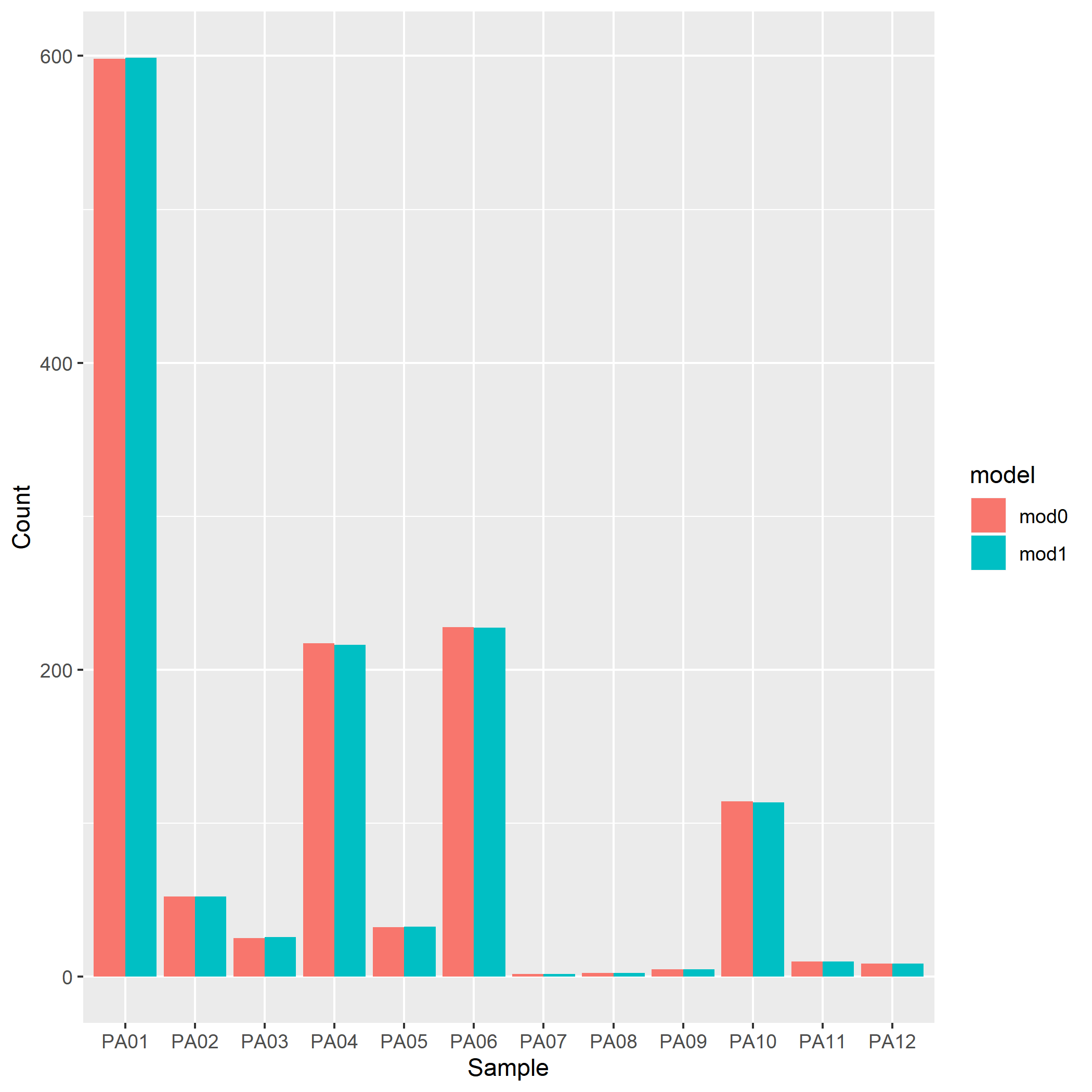 |
| *MUC16* | Mucin 16, Cell Surface Associated | 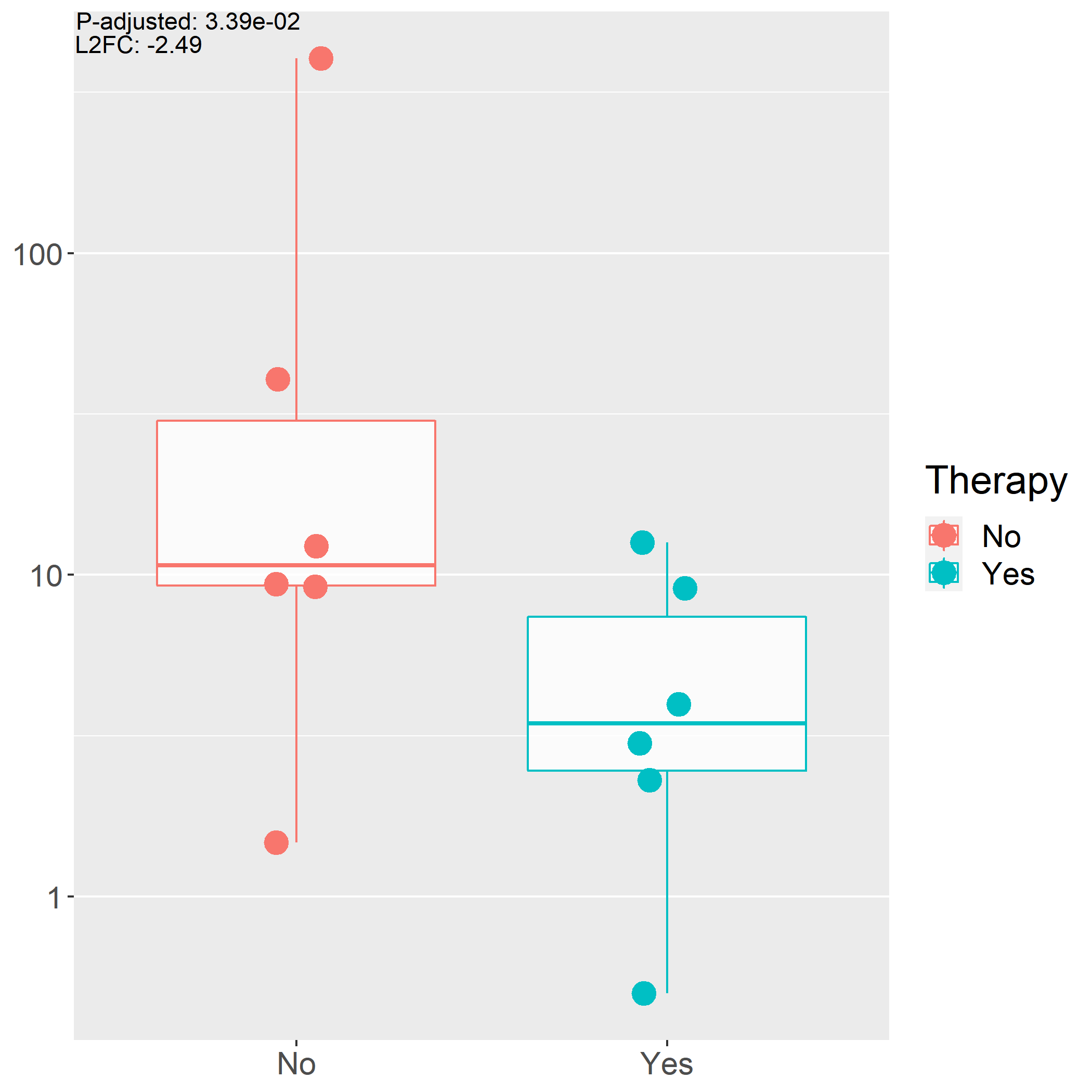 | 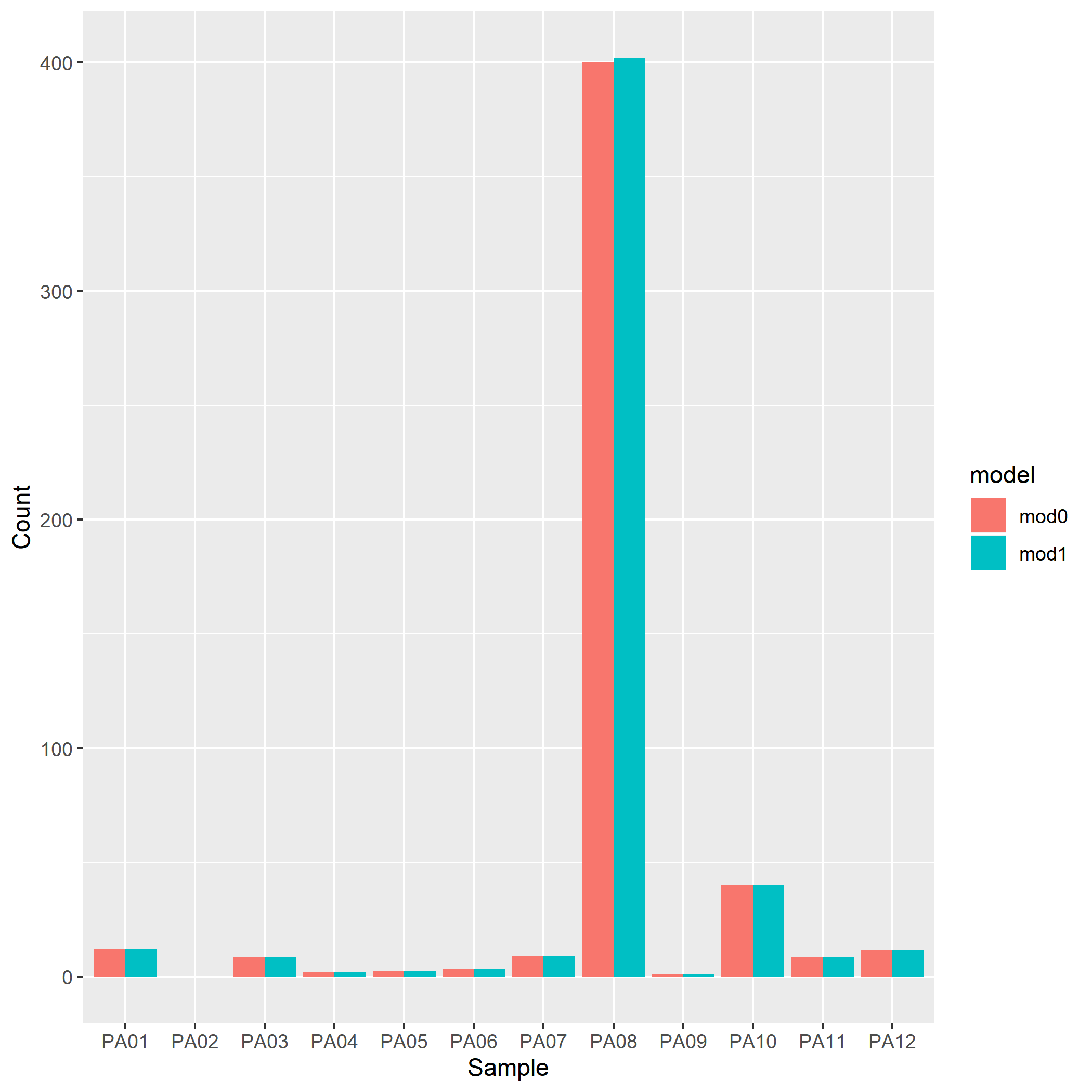 |
| *TMEM184A* | Transmembrane Protein 184A | 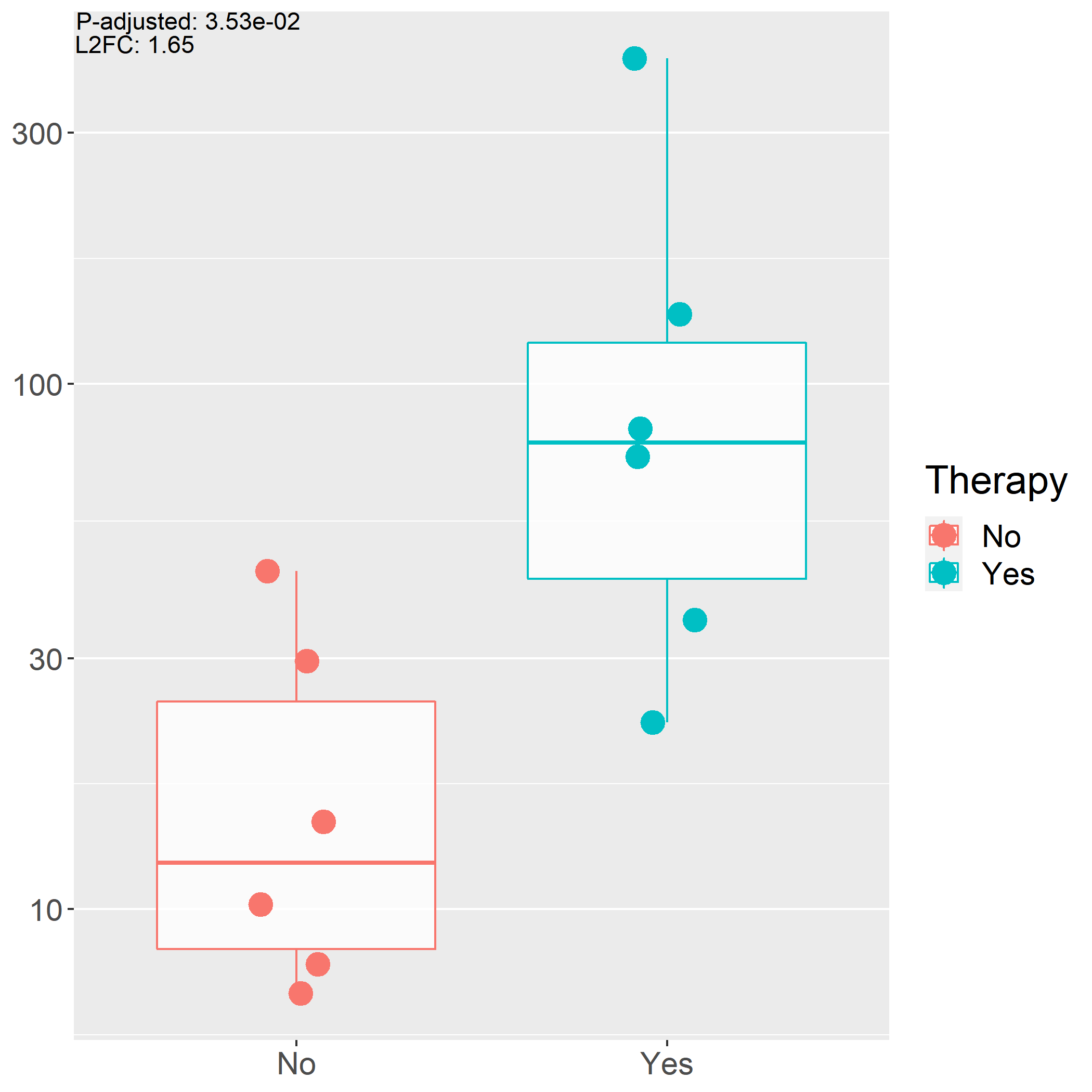 | 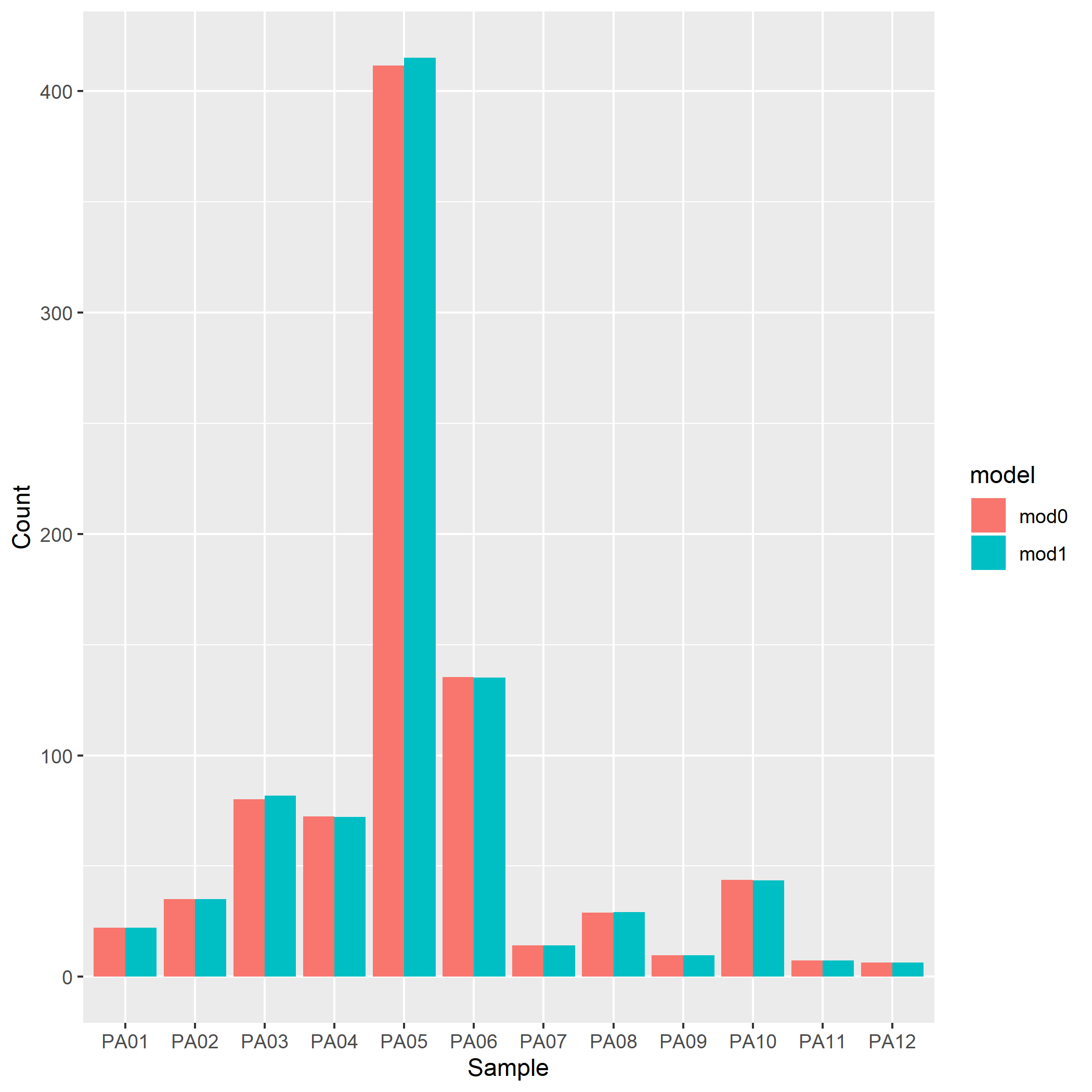 |
| *AHNAK2* | AHNAK Nucleoprotein 2 | 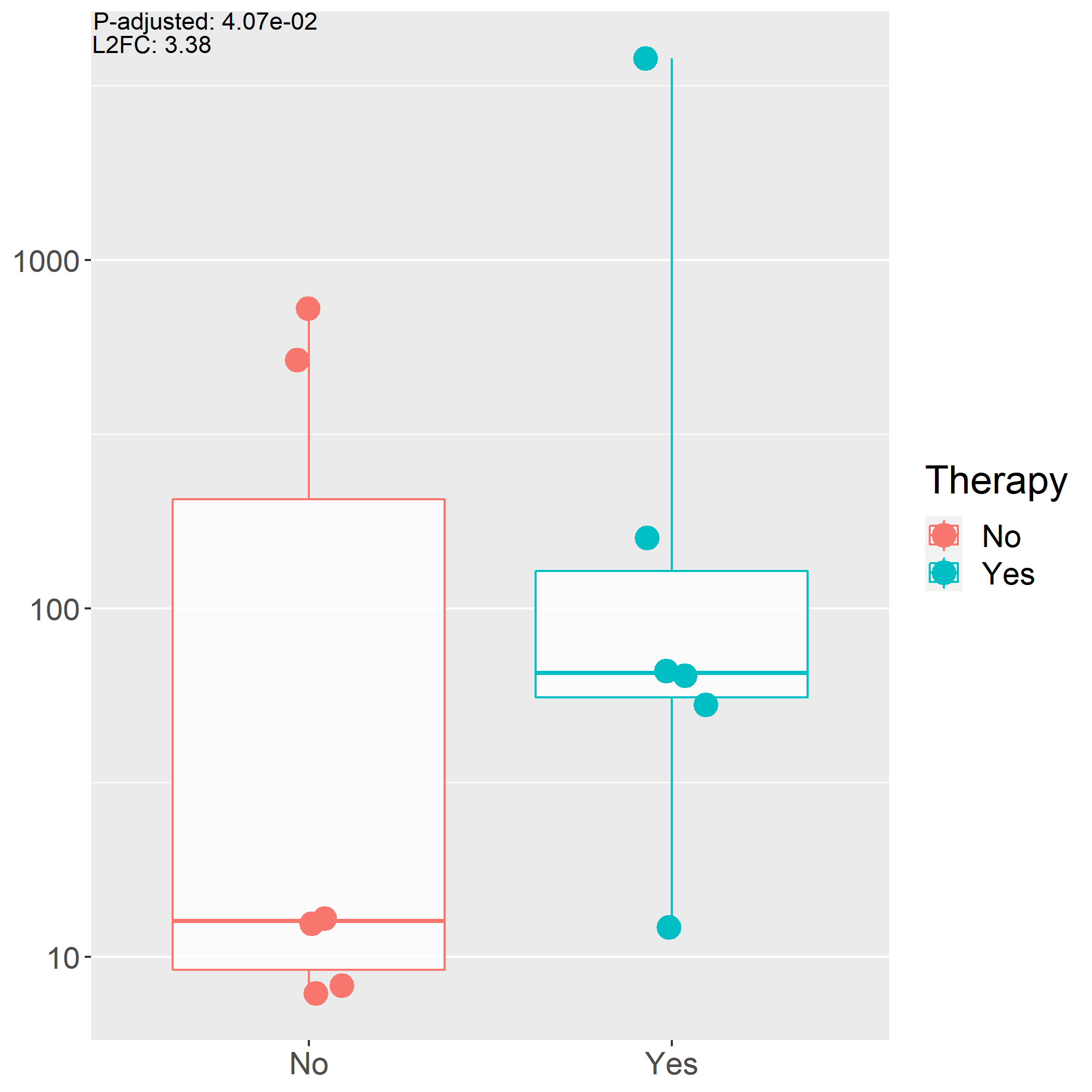 | 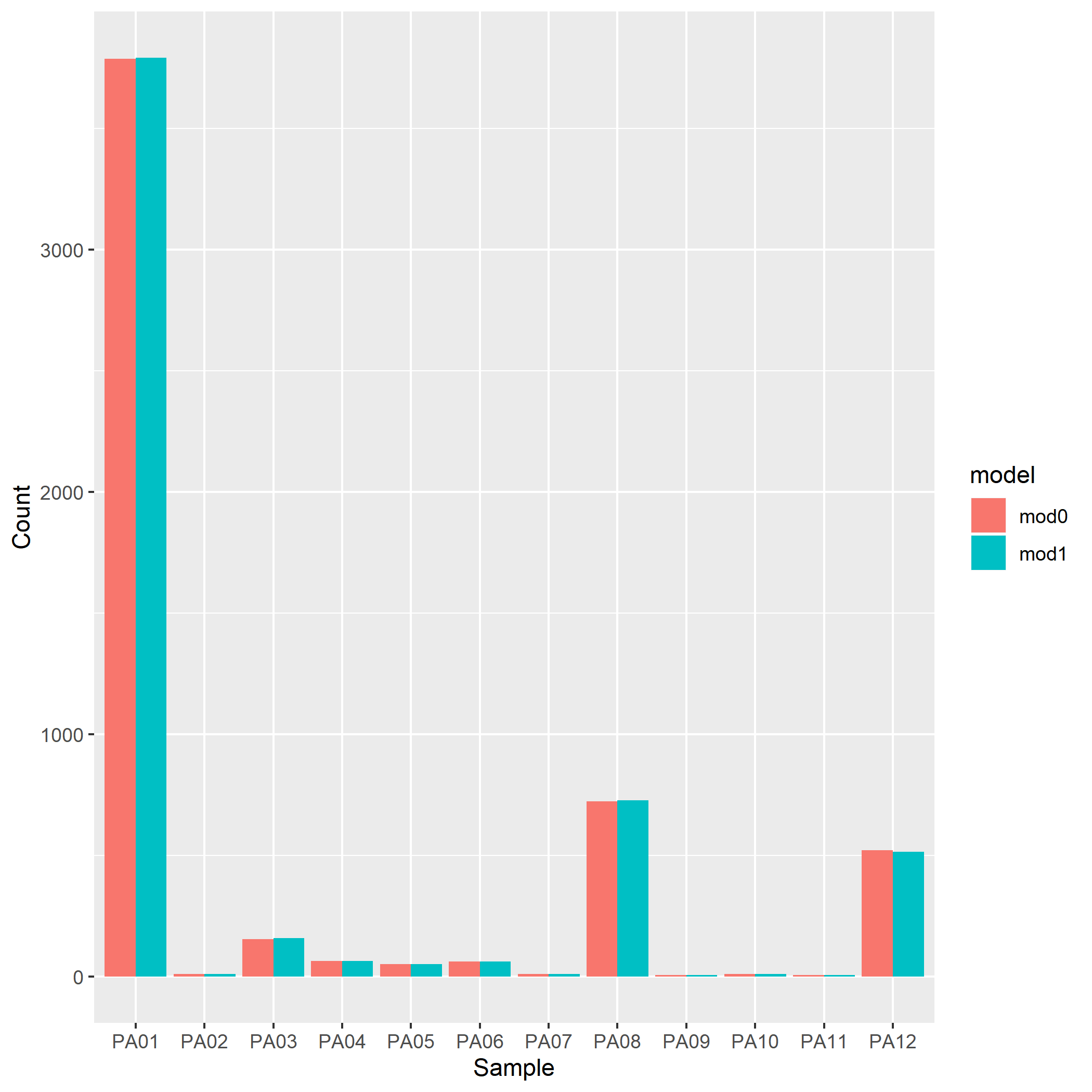 |
| *ADAMTS10* | ADAM Metallopeptidase With Thrombospondin Type 1 Motif 10 | 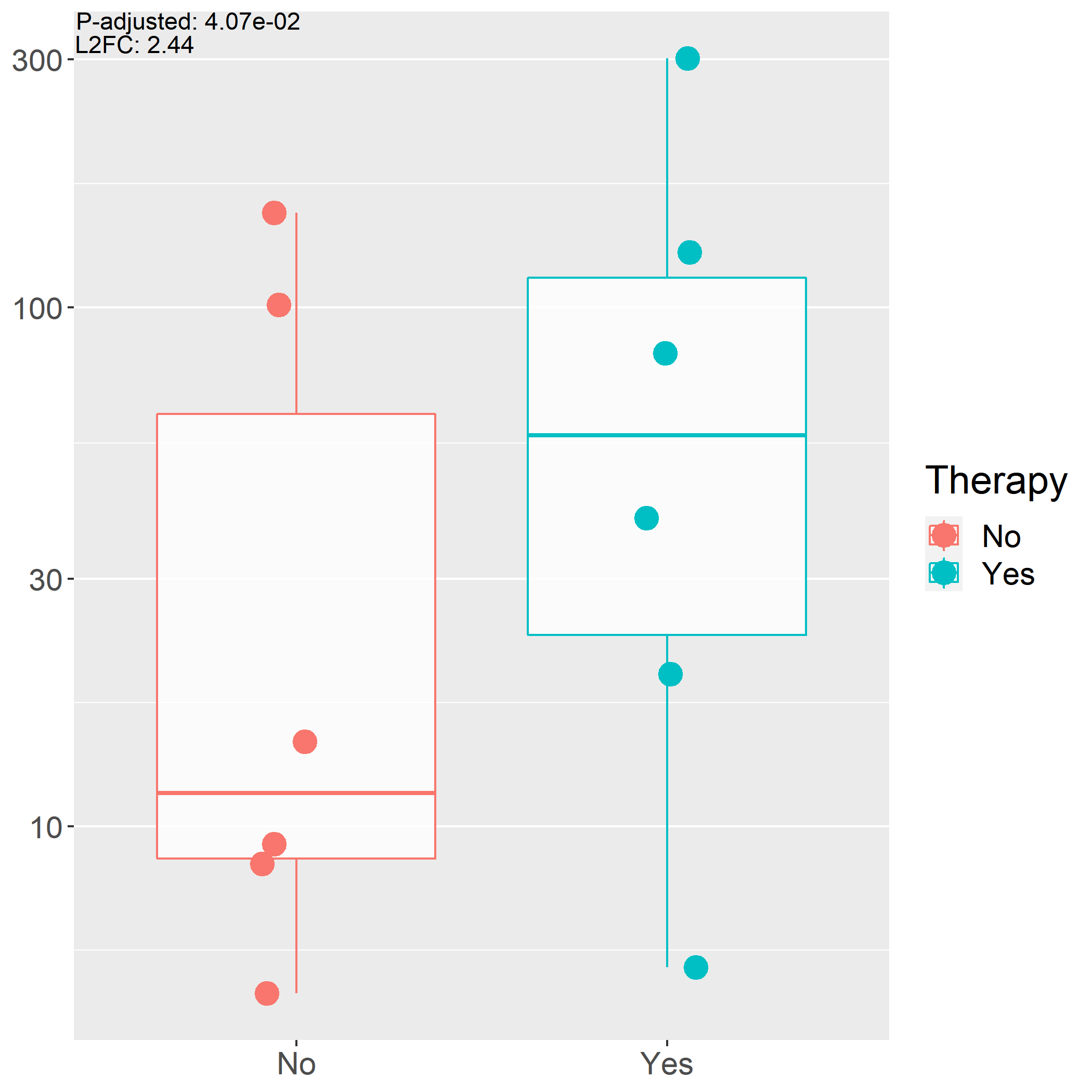 | 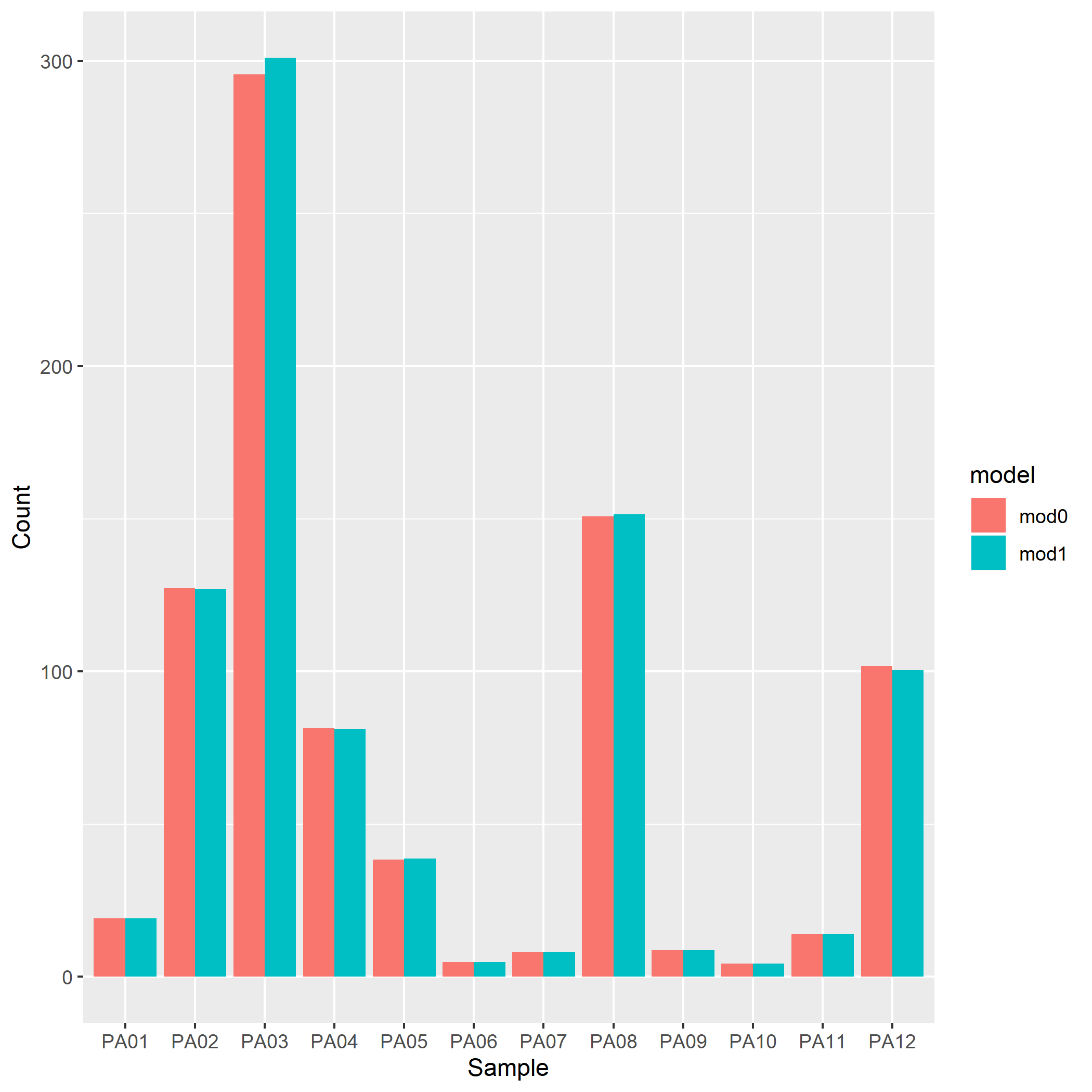 |
| *CPNE7* | Copine 7 | 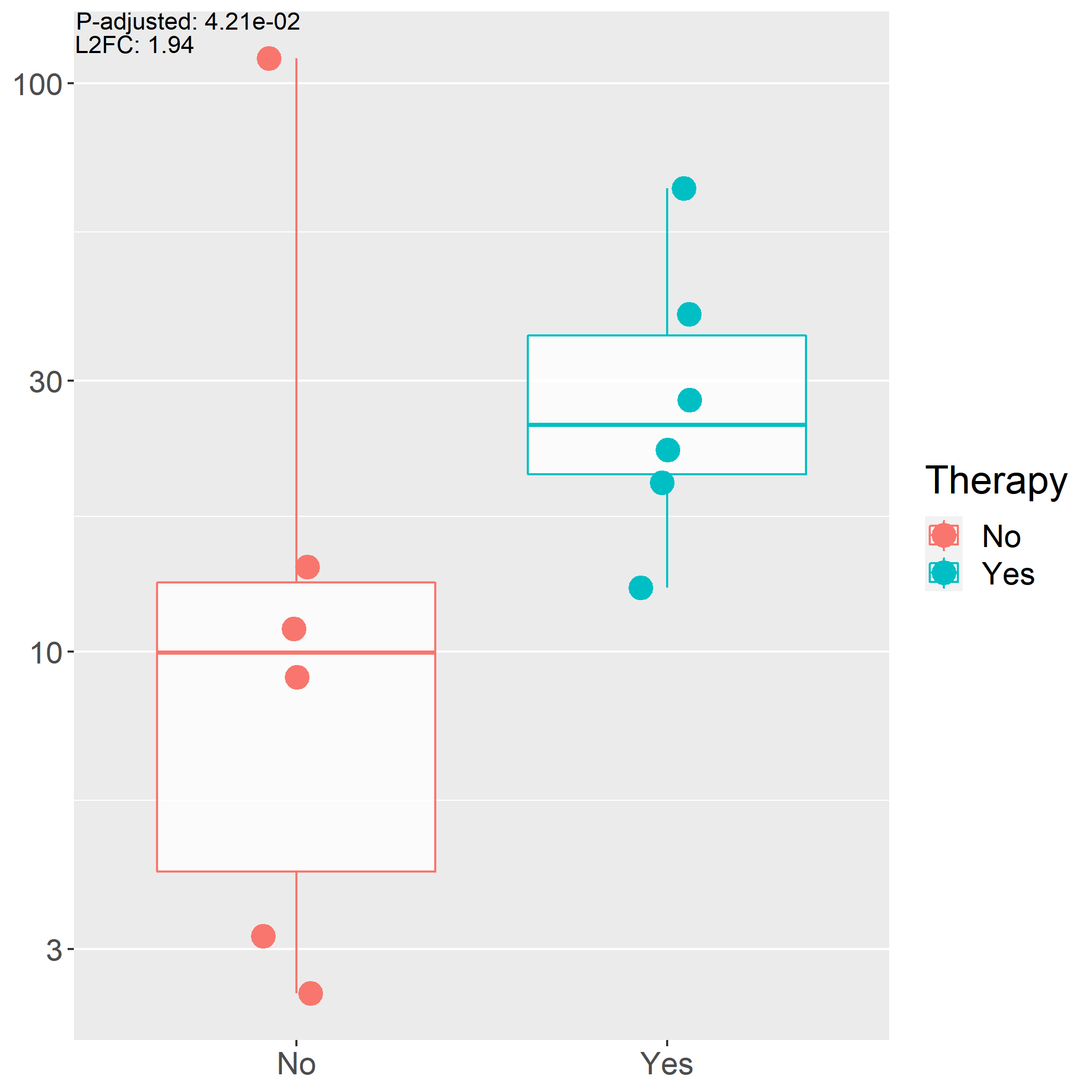 | 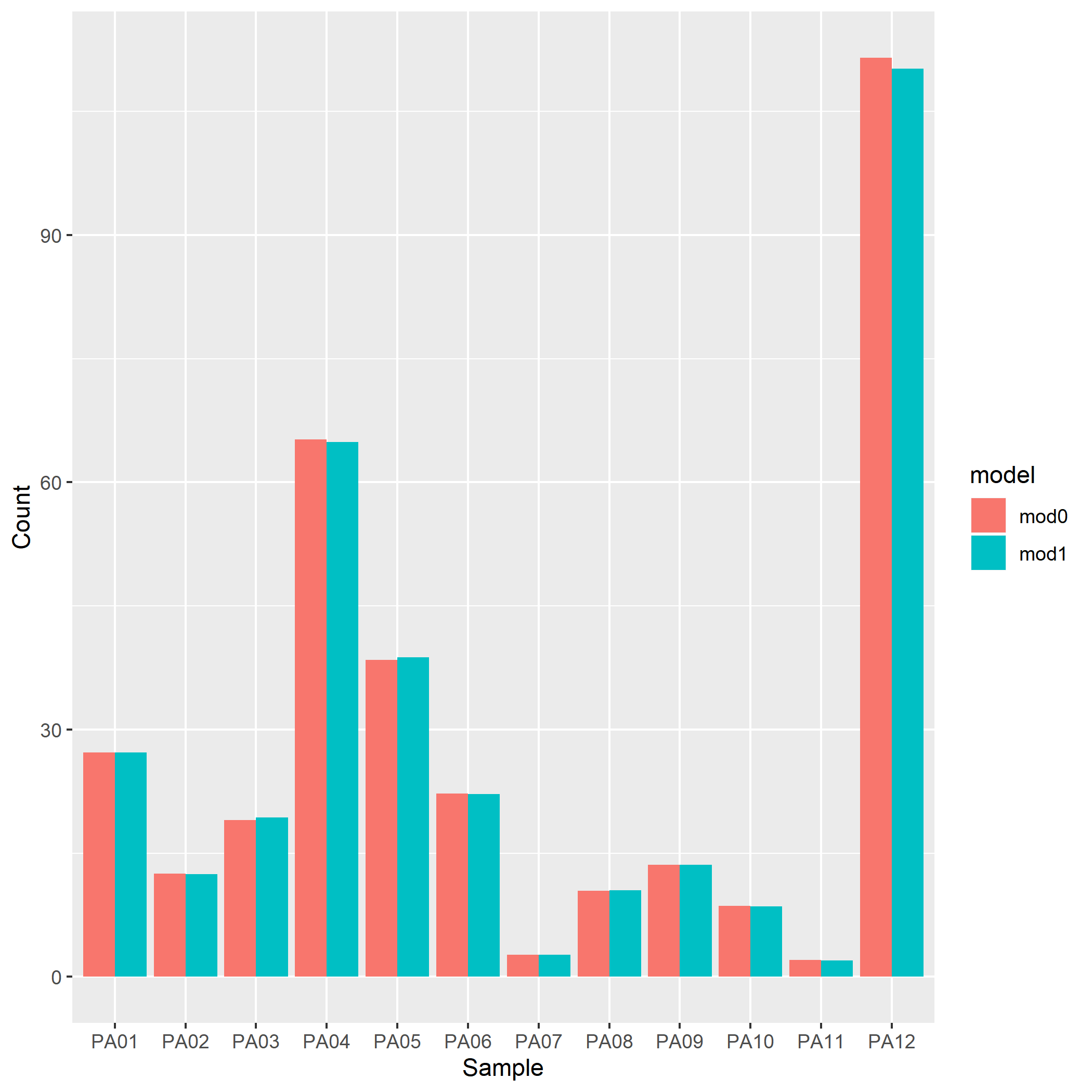 |
| *DTNA* | Dystrobrevin Alpha | 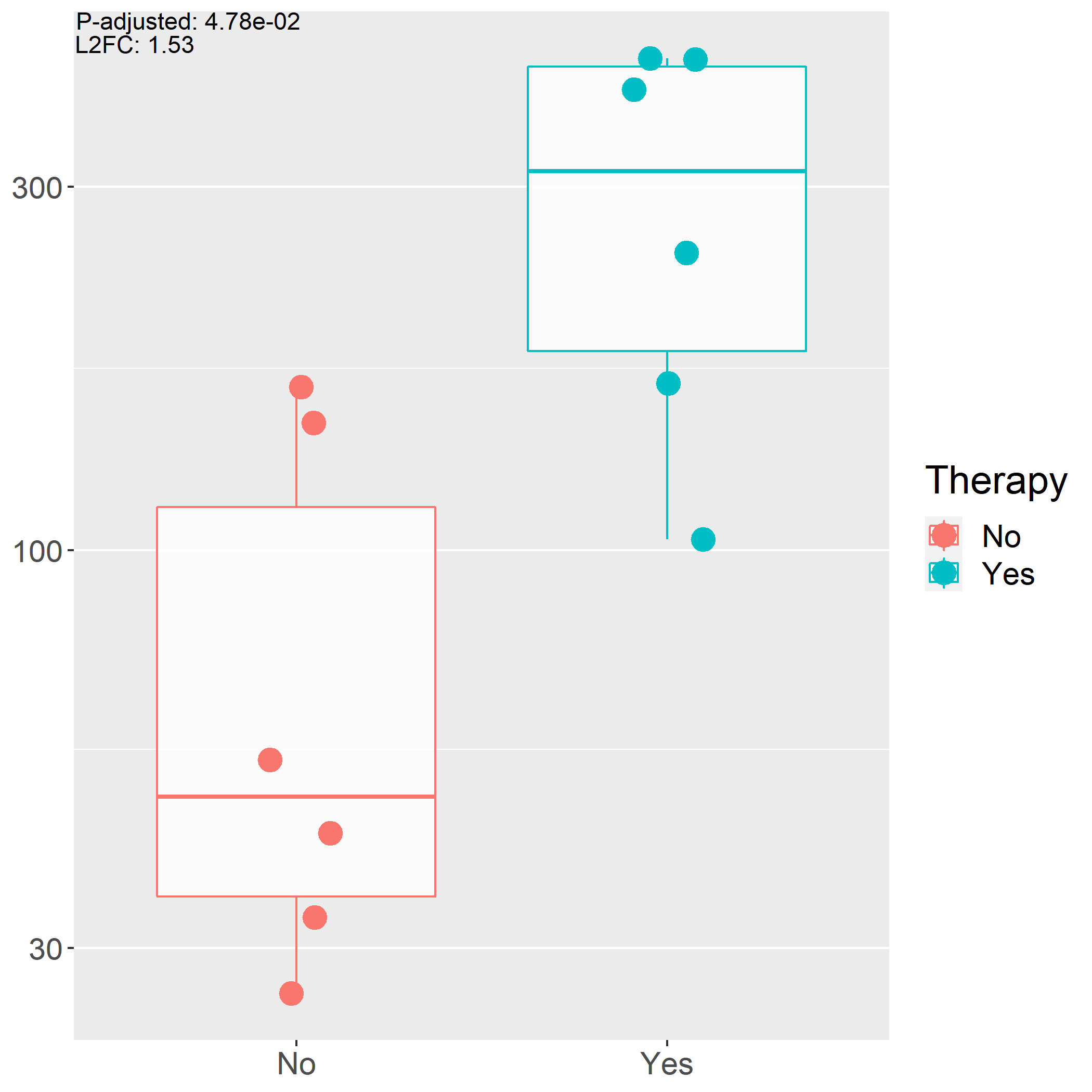 | 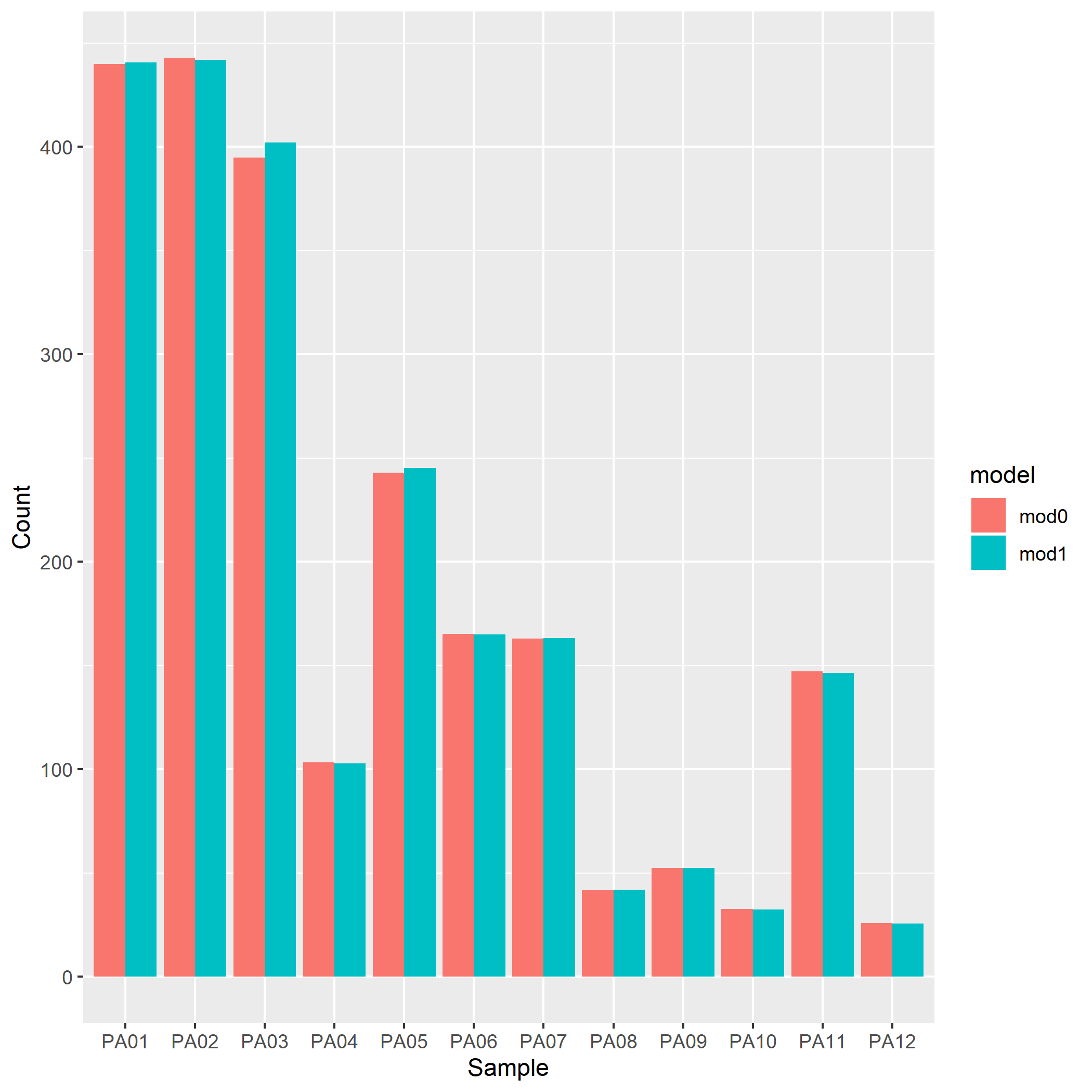 |

Supplementary Table 3. Scatter plots displaying data distribution for Kendall's Tau correlation between the read count matrix and Knosp, Ki-67 indexes. Displayed trend line and confidence intervals serve as a purely suggestive visual aid for the direction of the correlation - they are not connected to the actual correlation estimate values which are also displayed in the plot.

| **Gene symbol** | **Gene name** | **Correlation with Knosp index** | **Correlation with Ki - 67 index** |
| --- | --- | --- | --- |
| *OLFM2* | Olfactomedin 2 | 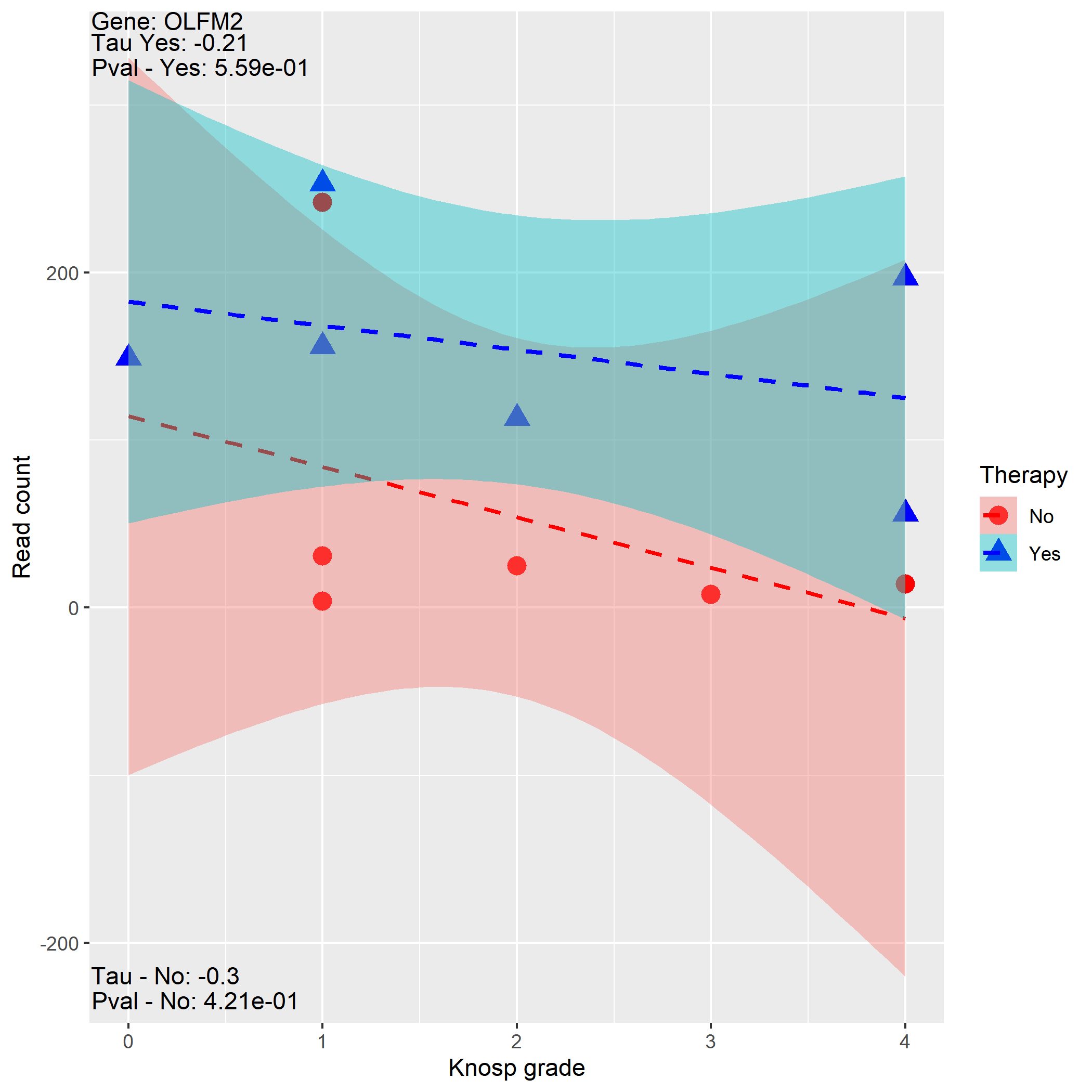 | 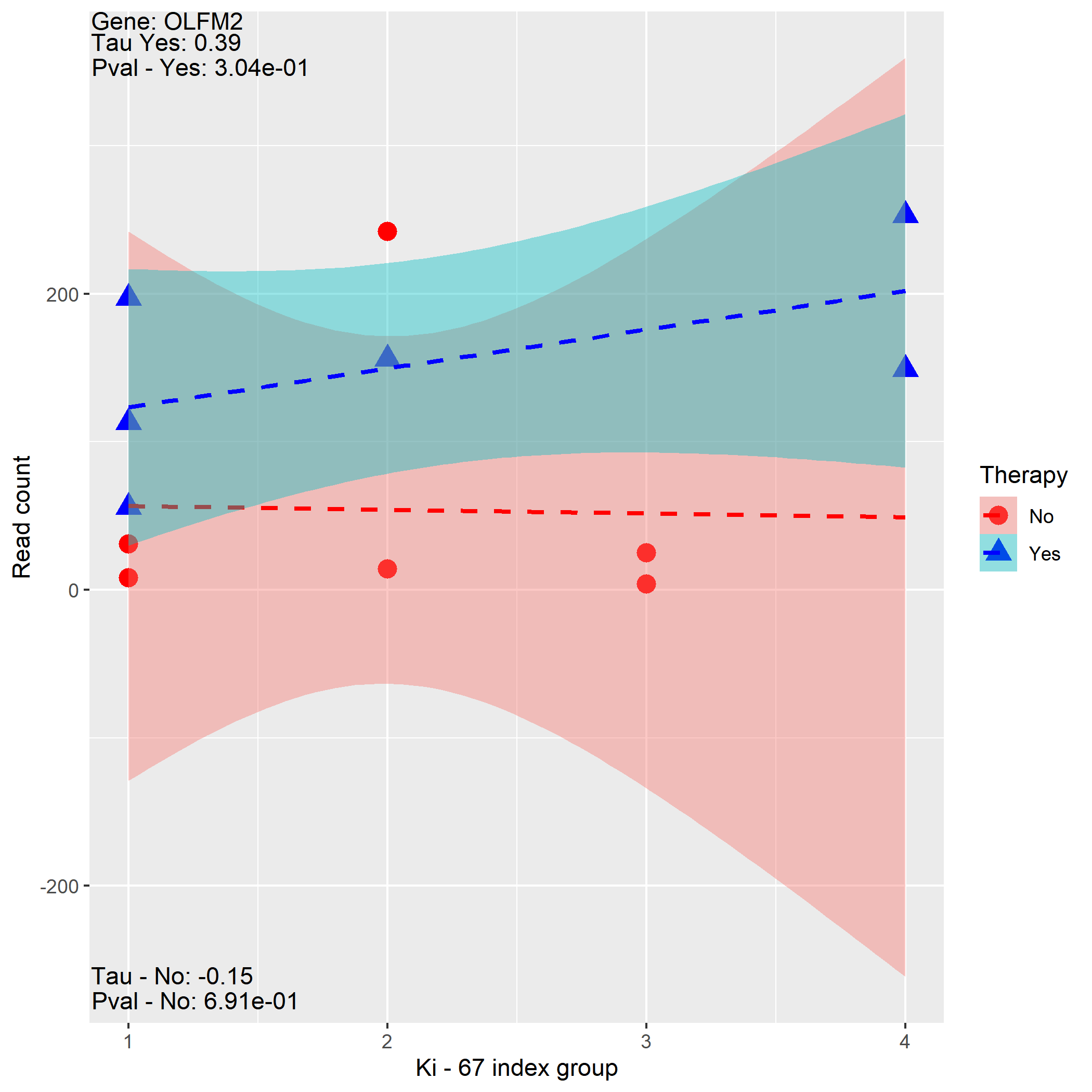 |
| *SLC6A1* | Solute Carrier Family 6 Member 1 | 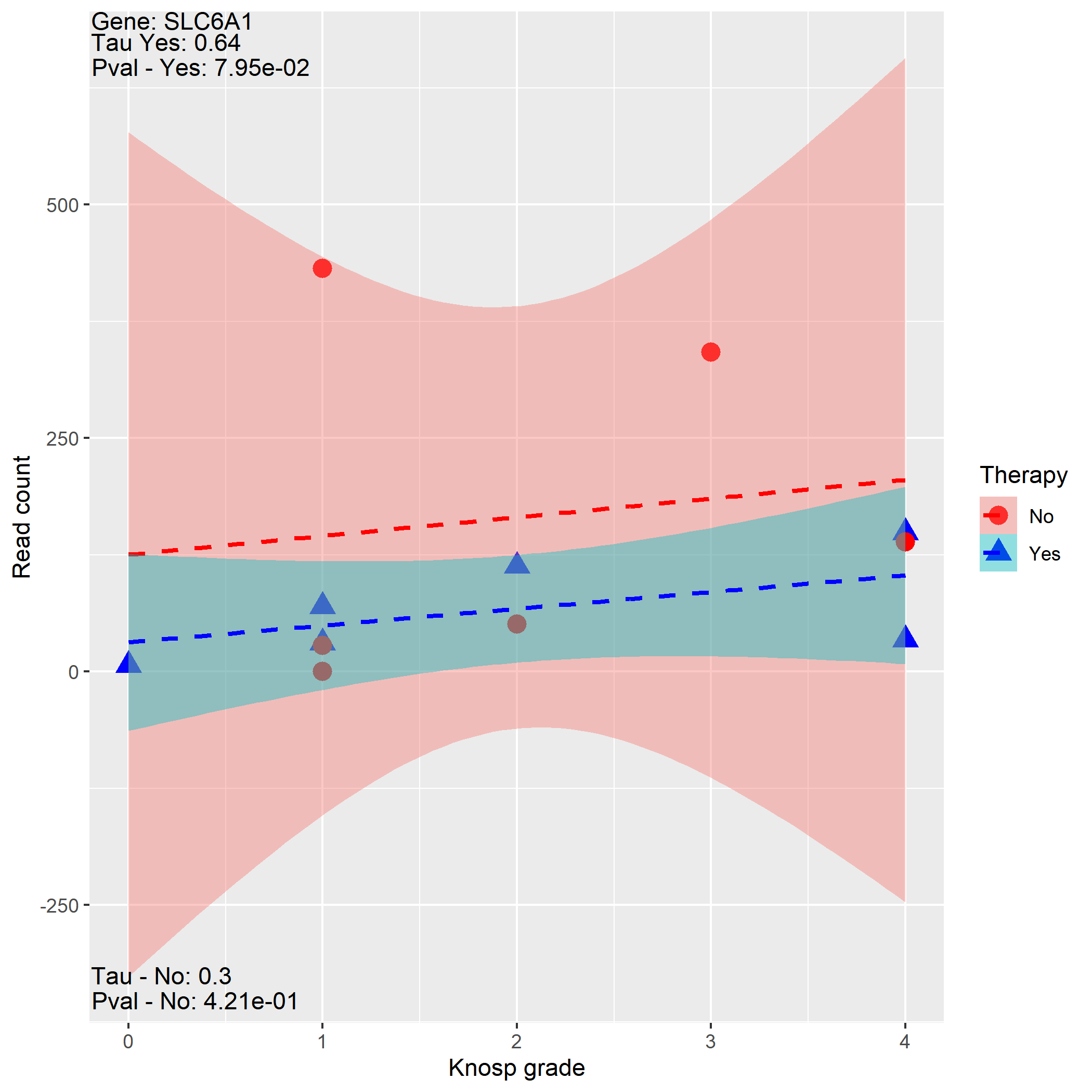 | 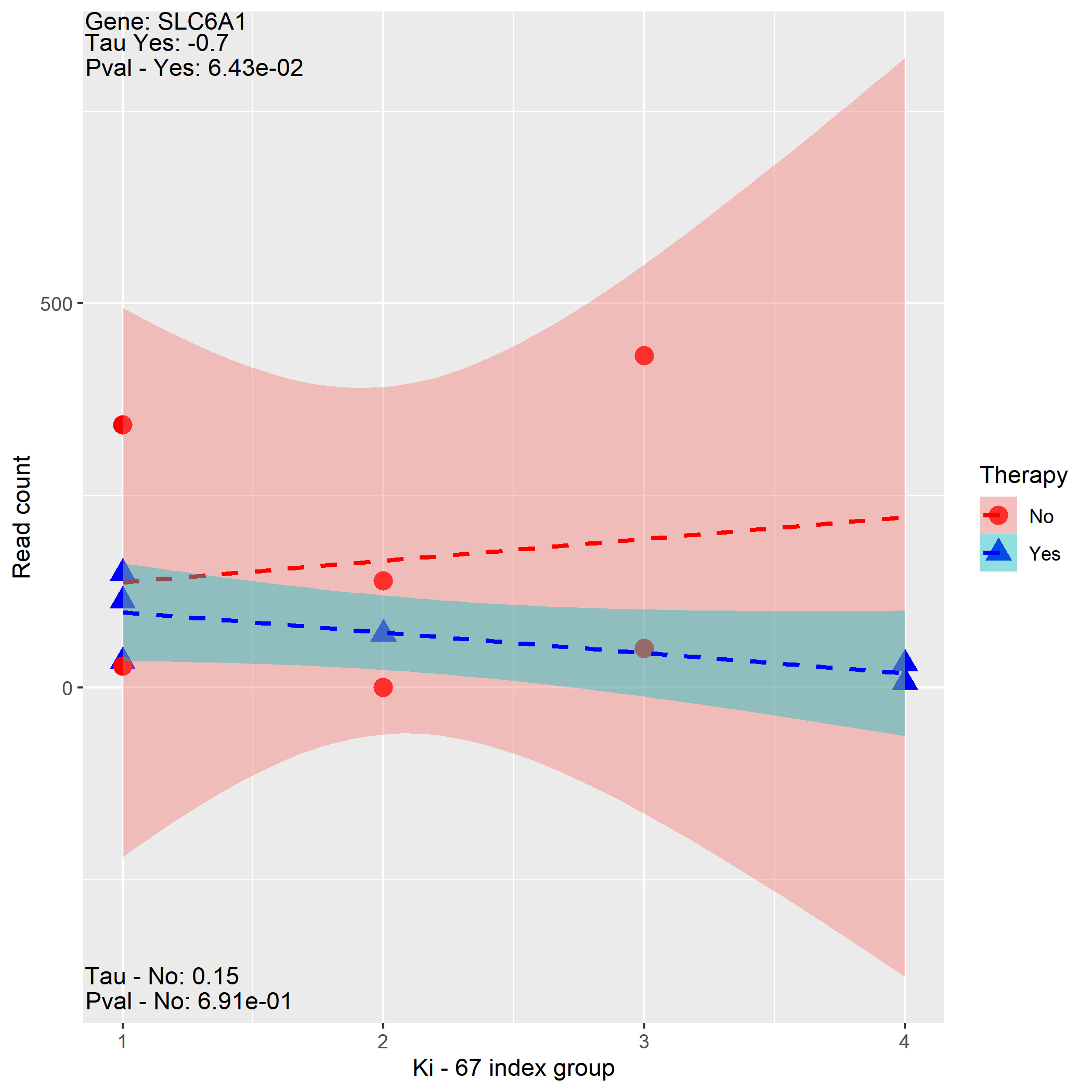 |
| *PDE4A* | Phosphodiesterase 4A | 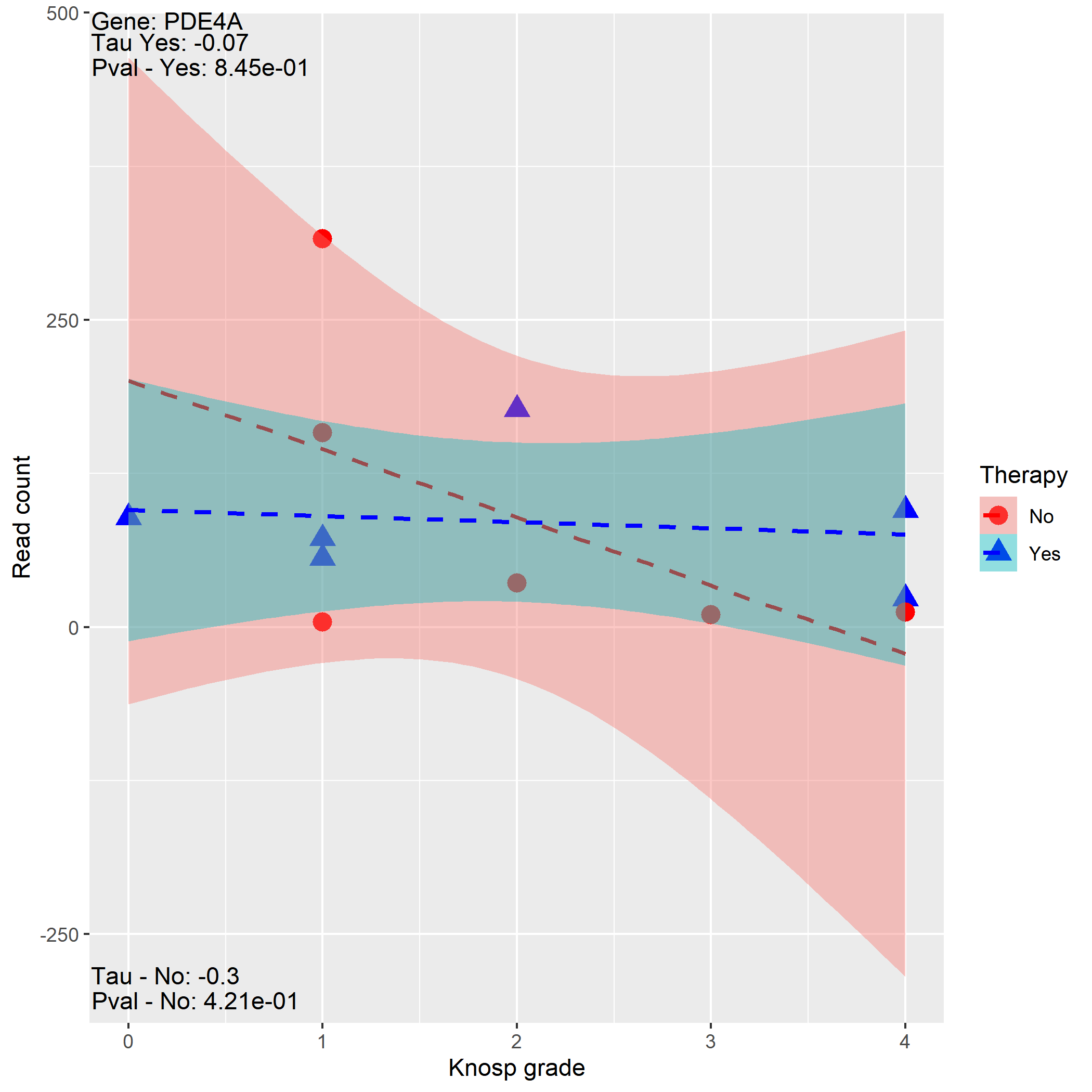 | 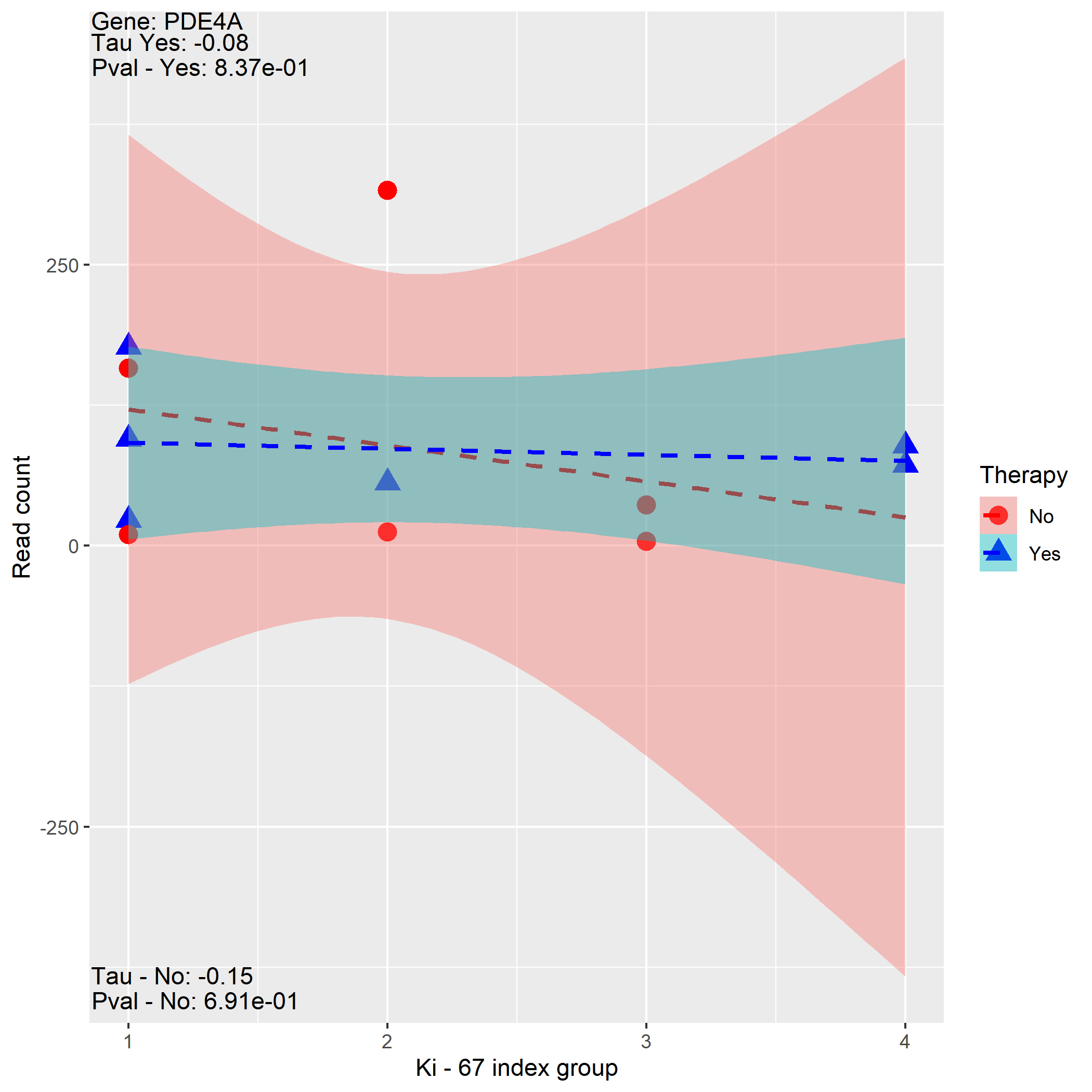 |
| *COL16A1* | Collagen Type XVI Alpha 1 Chain | 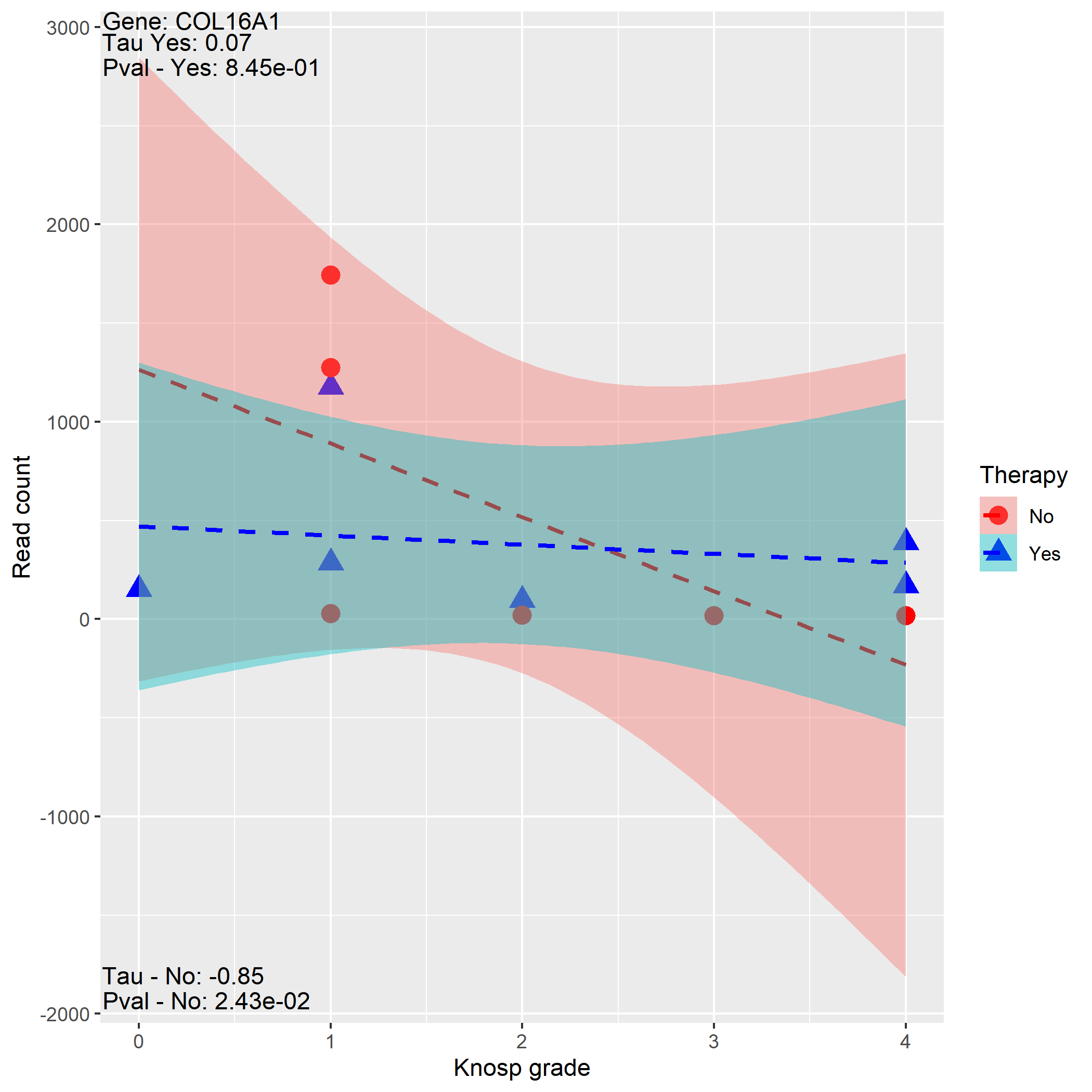 | 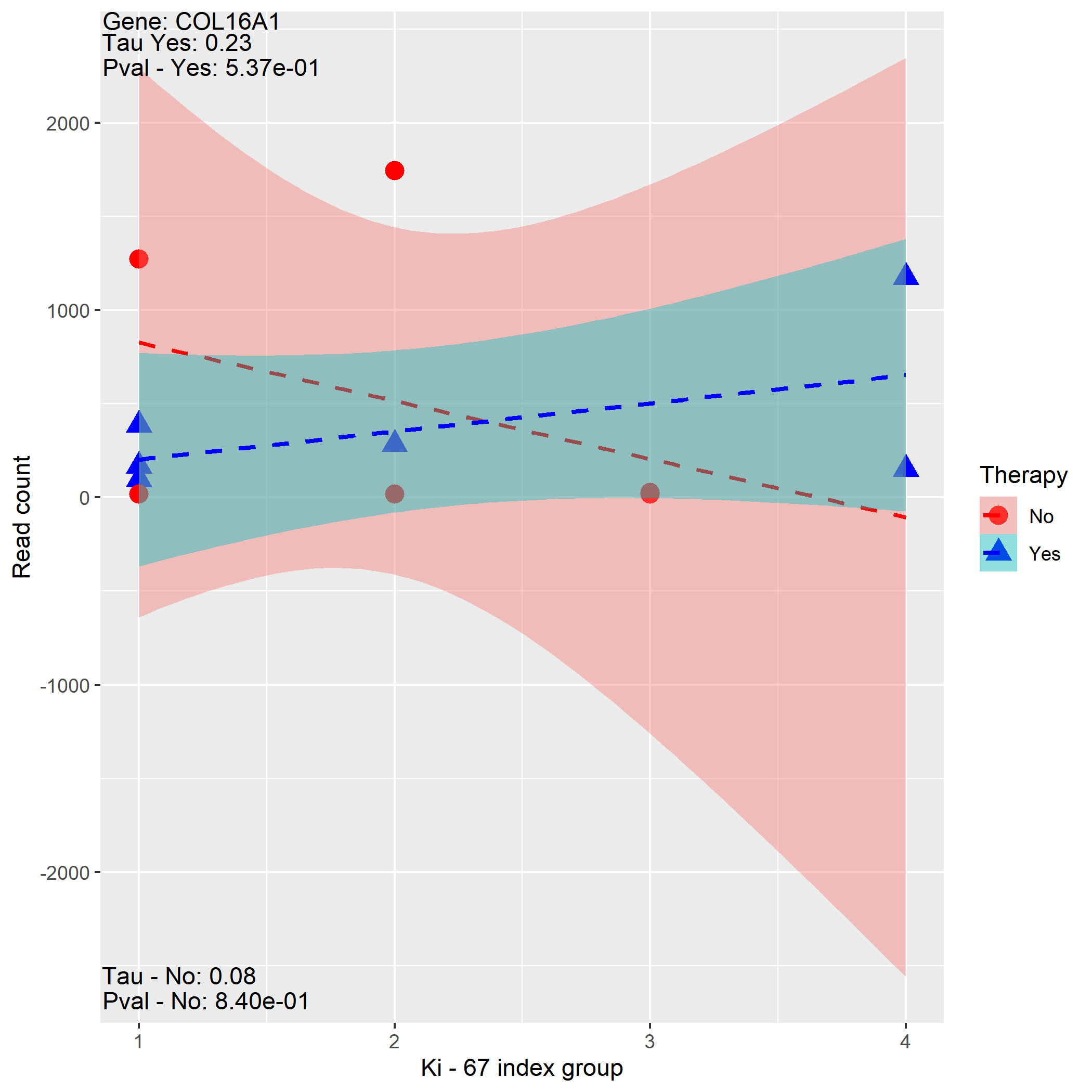 |
| *TMEM132C* | Transmembrane Protein 132C | 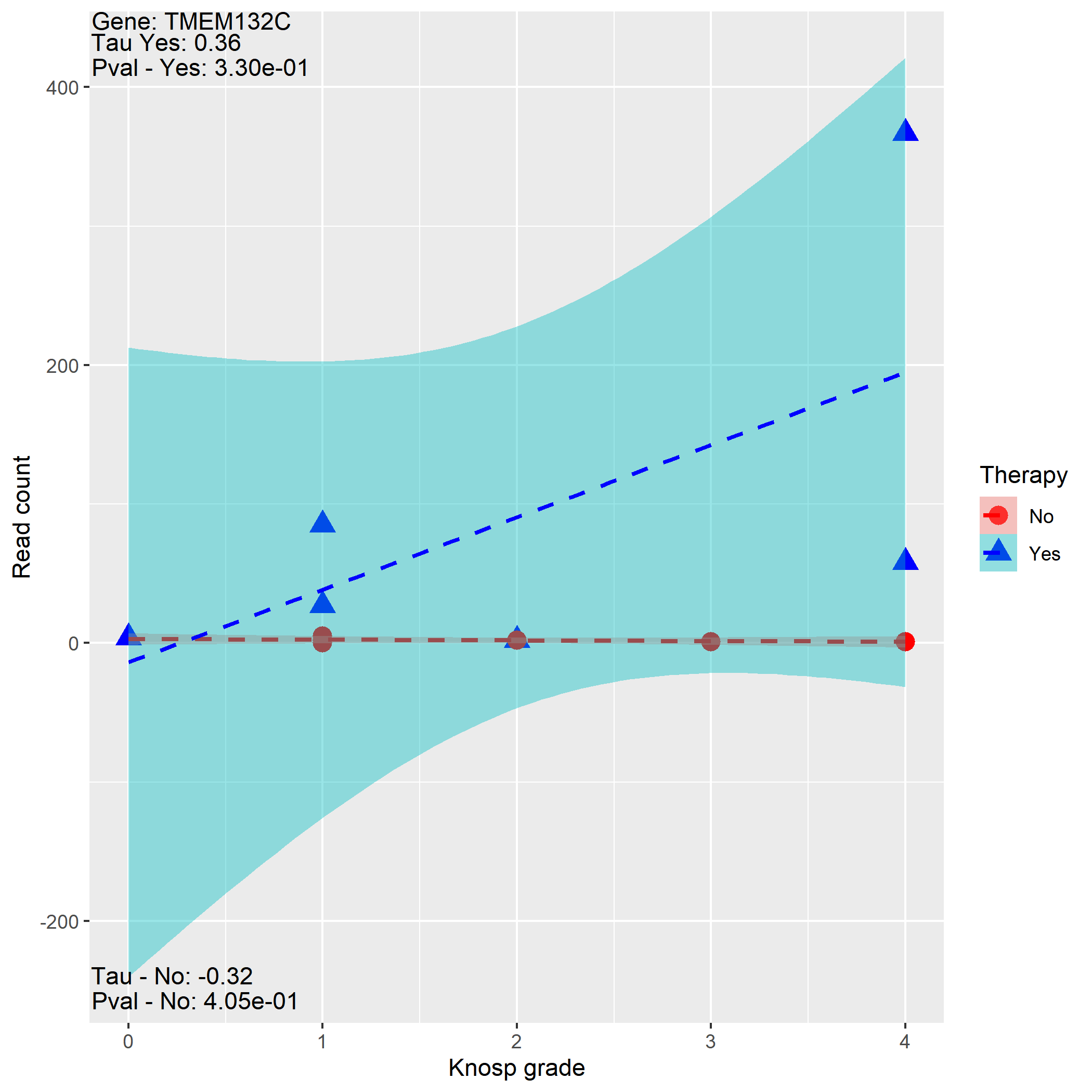 | 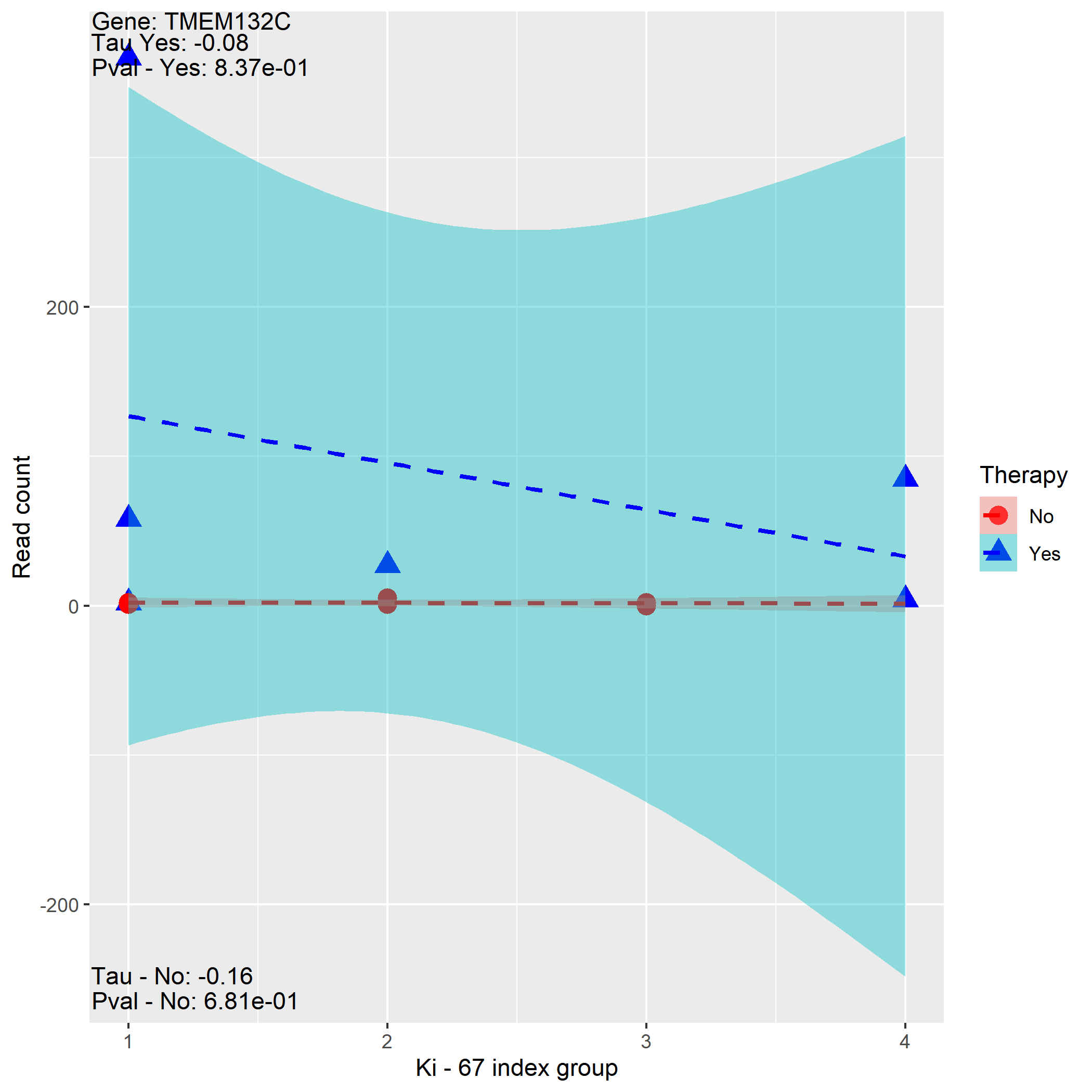 |
| *QPRT* | Quinolinate Phosphoribosyltransferase | 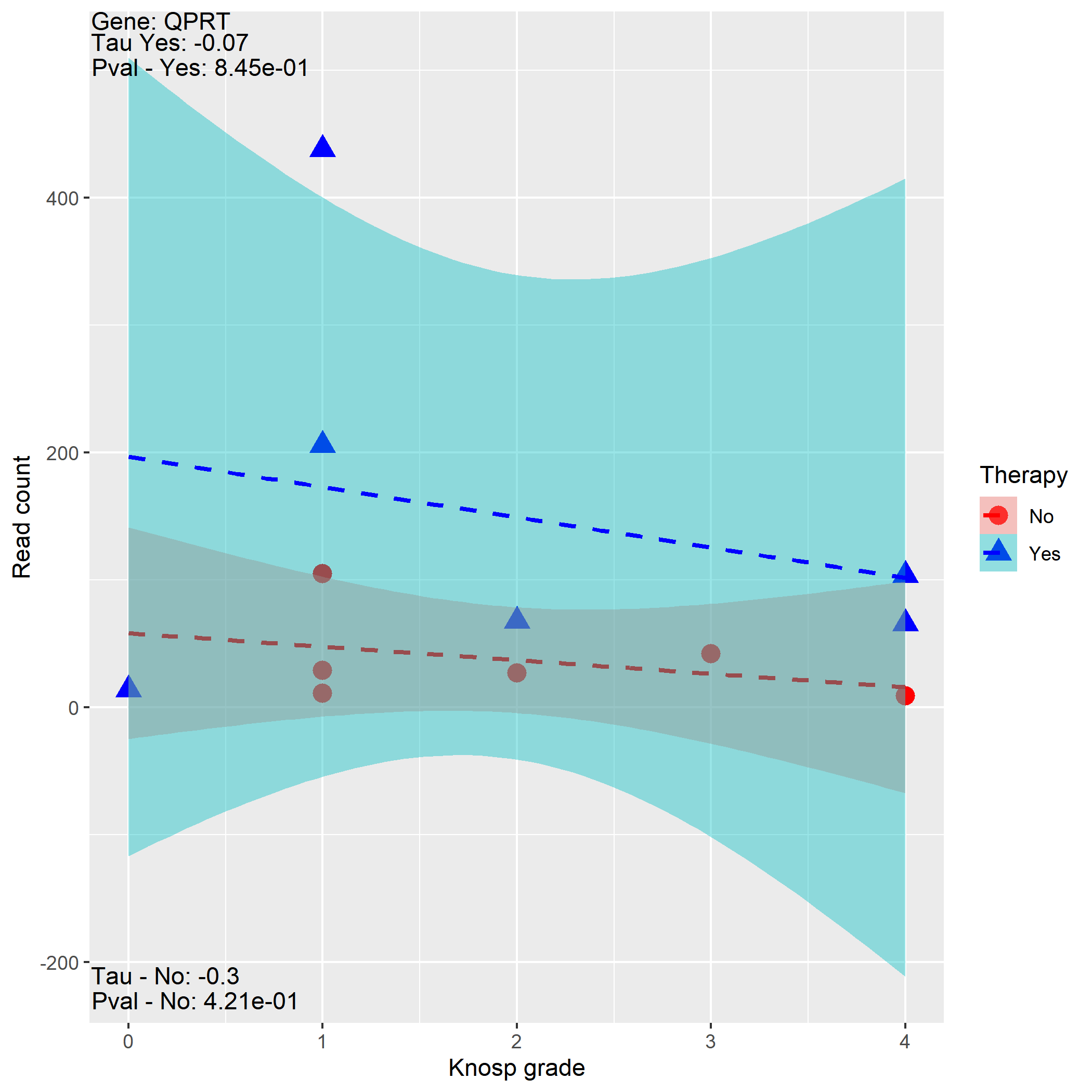 | 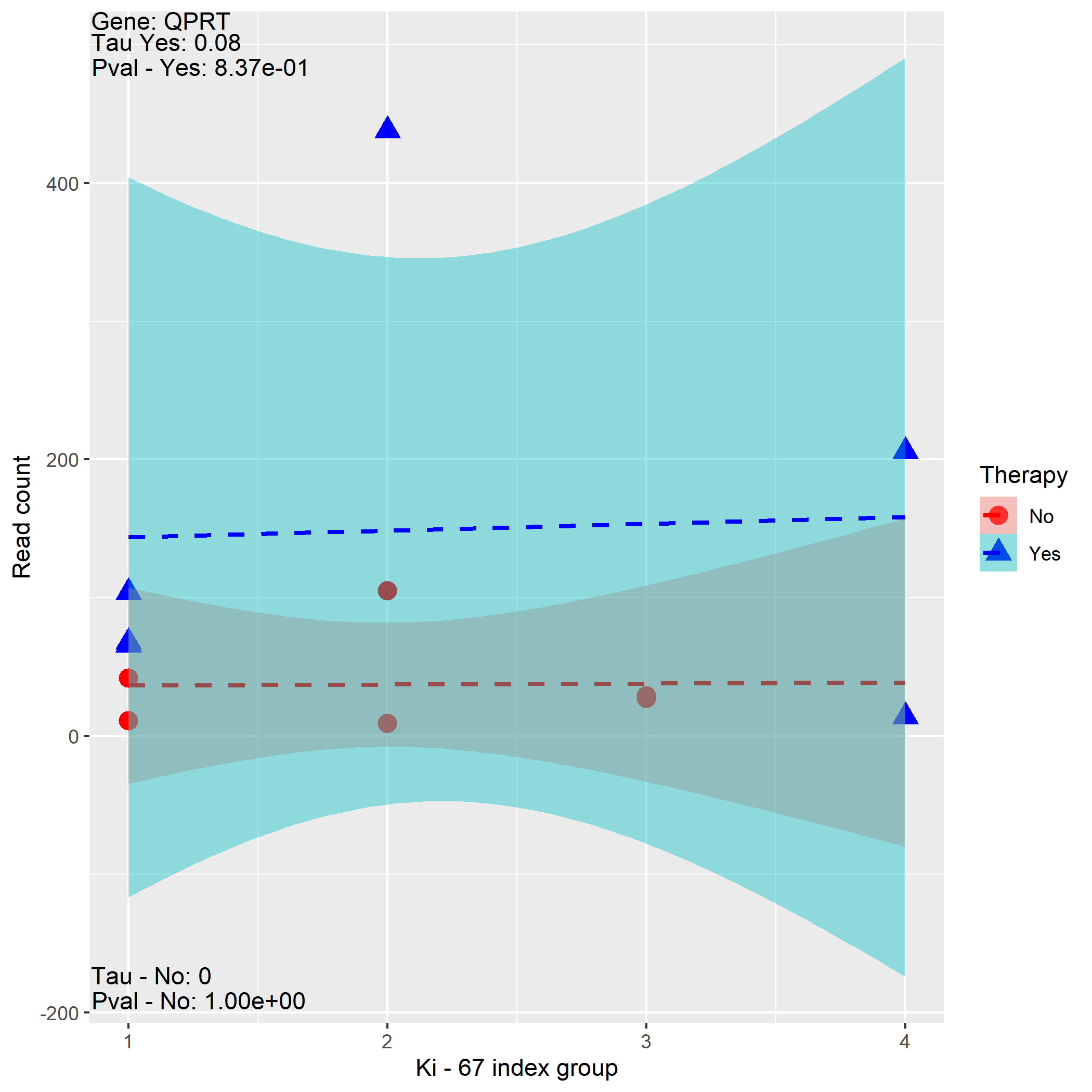 |
| *ATP1B2* | ATPase Na+/K+ Transporting Subunit Beta 2 |  |  |
| *SEPTIN14* | Septin 14 |  |  |
| *BCAM* | Basal Cell Adhesion Molecule (Lutheran Blood Group) |  |  |
| *COL6A1* | Collagen Type VI Alpha 1 Chain |  |  |
| *MACC1* | MET Transcriptional Regulator MACC1 |  |  |
| *CILP* | Cartilage Intermediate Layer Protein |  |  |
| *ARC* | Activity Regulated Cytoskeleton Associated Protein |  |  |
| *STUM* | Stum, Mechanosensory Transduction Mediator Homolog |  |  |
| *NPTX1* | Neuronal Pentraxin 1 |  |  |
| *CLU* | Clusterin |  |  |
| *MPPED1* | Metallophosphoesterase Domain Containing 1 |  |  |
| *COL8A2* | Collagen Type VIII Alpha 2 Chain |  |  |
| *GRHL2* | Grainyhead Like Transcription Factor 2 |  |  |
| *SSC5D* | Scavenger Receptor Cysteine Rich Family Member With 5 Domains |  |  |
| *GRIK3* | Glutamate Ionotropic Receptor Kainate Type Subunit 3 |  |  |
| *ADAMTSL2* | ADAMTS Like 2 |  |  |
| *PDE6A* | Phosphodiesterase 6A |  |  |
| *SLC8A2* | Solute Carrier Family 8 Member A2 |  |  |
| *PCP4L1* | Purkinje Cell Protein 4 Like 1 |  |  |
| *DIRAS2* | DIRAS Family GTPase 2 |  |  |
| *B4GALNT4* | Beta-1,4-N-Acetyl-Galactosaminyltransferase 4 |  |  |
| *APBA2* | Amyloid Beta Precursor Protein Binding Family A Member 2 |  |  |
| *THBD* | Thrombomodulin |  |  |
| *PTGS2* | Prostaglandin-Endoperoxide Synthase 2 |  |  |
| *SV2B* | Synaptic Vesicle Glycoprotein 2B |  |  |
| *LINC01529* | Long Intergenic Non-Protein Coding RNA 1529 |  |  |
| *ST6GALNAC5* | ST6 N-Acetylgalactosaminide Alpha-2,6-Sialyltransferase 5 |  |  |
| *CALB2* | Calbindin 2 |  |  |
| *MUC16* | Mucin 16, Cell Surface Associated |  |  |
| *TMEM184A* | Transmembrane Protein 184A |  |  |
| *AHNAK2* | AHNAK Nucleoprotein 2 |  |  |
| *ADAMTS10* | ADAM Metallopeptidase With Thrombospondin Type 1 Motif 10 |  |  |
| *CPNE7* | Copine 7 |  |  |
| *DTNA* | Dystrobrevin Alpha |  |  |

Supplementary Table 4. Combined interaction scores for all of the detected protein-protein pairs from “STRING” database along with interaction types and their contribution to the combined score.

| **DEGs with interacting proteins** | **Interaction types** | **Combined interaction score** |
| --- | --- | --- |
| *COL16A1*, *COL6A1* | Association in curated databases (0.900);  Coexpression (0.214);  Putative homologue coexpression in other species (0.229); | 0.934 |
| *COL8A2*, *COL16A1* | Association in curated databases (0.900); Co-occurrences across genomes (0.287); Coexpression (0.120);  Putative homologue coexpression in other species (0.064) | 0.917 |
| *COL8A2*, *COL6A1* | Association in curated databases (0.900); Coexpression (0.093);  Putative homologue coexpression in other species (0.055) | 0.906 |
| *ADAMTSL2*, *ADAMTS10* | Association in curated databases (0.900) | 0.900 |
| *MPPED1*, *SLC6A1* | Co-expression (0.490);  Putative homologue coexpression in other species (0.062) | 0.501 |

Supplementary Table 5. Individual level DEGs related to cell cycle, proliferation and apoptosis signalling pathways in this study and reports that analysed invasive vs non-invasive PitNETs.

a, b, c, d, e, f – indicate overlapping candidates.

To understand the involvement of factors for distinct molecular pathways related to cell cycle, proliferation and apoptosis we obtained lists of pathway factors from publicly available databases and compared these with our results and results in literature where transcriptome analysis have been carried out in relation to invasiveness phenotype of PitNETs. DEGs from the publications were selected and used for comparison with logFC over 2 and P_adjusted_ over 0.005, in publications where no table format information where available we choose the DEGs indicated in the text of the manuscript.

*The assessed signalling pathways:*

ALCALA_APOPTOSIS, GO_CELL_CYCLE, GO_CELL_GROWTH, CELL_PROLIFERATION_GO_0008283, HALLMARK_APOPTOSIS, GO_IMMUNE_RESPONSE_TO_TUMOR_CELL, GO_NEGATIVE_REGULATION_OF_CELL_GROWTH, GO_NEGATIVE_REGULATION_OF_TUMOR_NECROSIS_FACTOR_BIOSYNTHETIC_PROCESS, GO_NEGATIVE_REGULATION_OF_TUMOR_NECROSIS_FACTOR_MEDIATED_SIGNALING_PATHWAY, GO_POSITIVE_REGULATION_OF_CELL_GROWTH, GO_POSITIVE_REGULATION_OF_RESPONSE_TO_TUMOR_CELL, GO_POSITIVE_REGULATION_OF_TUMOR_NECROSIS_FACTOR_BIOSYNTHETIC_PROCESS, GO_POSITIVE_REGULATION_OF_TUMOR_NECROSIS_FACTOR_MEDIATED_SIGNALING_PATHWAY, GO_POSITIVE_REGULATION_OF_TUMOR_NECROSIS_FACTOR_SECRETION, REGULATION_OF_CELL_GROWTH, GO_REGULATION_OF_EXTENT_OF_CELL_GROWTH, GO_REGULATION_OF_RESPONSE_TO_TUMOR_CELL, GO_REGULATION_OF_TUMOR_NECROSIS_FACTOR_MEDIATED_SIGNALING_PATHWAY, GO_RESPONSE_TO_TUMOR_CELL, GO_RESPONSE_TO_TUMOR_NECROSIS_FACTOR, KEGG_TGF_BETA_SIGNALING_PATHWAY,

| **Publication** | **Type of PitNET and study design** | **DEGs related to cell cycle, proliferation and apoptosis signalling pathways** | **Involved pathway** |
| --- | --- | --- | --- |
| *The results of this manuscript* | 6 somatotropinomas with preoperative SSA/DA treatment *vs*. 6 somatotropinomas without preoperative SSA/DA treatment; RNA-seq | QPRT | ALCALA_APOPTOSIS |
|  |  | SEPTIN14 | GO_CELL_CYCLE |
|  |  | CLU^a^ | HALLMARK_APOPTOSIS |
|  |  | PTGS2 | GO_CELL_CYCLE; GO_RESPONSE_TO_TUMOR_NECROSIS_FACTOR |
| *Kim et al. 2019*  *PMID: [31565884]* | 3 noninvasive non-functioning PitNET *vs*. 11 invasive non-functioning PitNET;  RNA-seq | EPHA7 | GO_CELL_GROWTH; GO_NEGATIVE_REGULATION_OF_CELL_GROWTH; GO_REGULATION_OF_EXTENT_OF_CELL_GROWTH |
|  |  | DCN | HALLMARK_APOPTOSIS; KEGG_TGF_BETA_SIGNALING_PATHWAY |
|  |  | IGFBP6 | CELL_PROLIFERATION_GO_0008522; HALLMARK_APOPTOSIS |
|  |  | BGN | HALLMARK_APOPTOSIS |
|  |  | IGFBP4 | GO_CELL_GROWTH; CELL_PROLIFERATION_GO_0008521 |
|  |  | IFITM1 | CELL_PROLIFERATION_GO_0008516 |
|  |  | ANXA1 | GO_CELL_CYCLE; HALLMARK_APOPTOSIS |
| *Falch et al. 2018*  *PMID:*  *[29259037]* | 4 fast growing gonadotroph PitNET *vs*. 4 slow growing gonadotroph PitNET; RNA-seq | GHRL | CELL_PROLIFERATION_GO_0008497; GO_NEGATIVE_REGULATION_OF_TUMOR_NECROSIS_FACTOR_BIOSYNTHETIC_PROCESS |
|  |  | EME1 | GO_CELL_CYCLE |
|  |  | ANXA11 | GO_CELL_CYCLE |
|  |  | CHTF18 | GO_CELL_CYCLE |
| *Cao et al. 2015*  *PMID: [25824863]* | 8 invasive PitNET *vs*. 8 non-invasive PitNET; gene expression array | WBP2NL | GO_CELL_CYCLE |
|  |  | SEMA6D | GO_CELL_GROWTH; GO_NEGATIVE_REGULATION_OF_CELL_GROWTH; GO_REGULATION_OF_EXTENT_OF_CELL_GROWTH |
|  |  | BCAT1 | GO_CELL_CYCLE; CELL_PROLIFERATION_GO_0008311 |
|  |  | COL18A1 | CELL_PROLIFERATION_GO_0008402 |
| *Wierinckx et al. 2007*  *PMID:*  *[17914117]* | 3 non-invasive prolactinomas *vs*. 4 invasive prolactinomas vs. 3 aggressive prolactinomas; gene expression array | PTTG1 | GO_CELL_CYCLE |
|  |  | CCNB1 | GO_CELL_CYCLE |
|  |  | TRIM36^b^ | GO_CELL_CYCLE |
|  |  | DBF4 | GO_CELL_CYCLE |
|  |  | RACGAP1 | GO_CELL_CYCLE; CELL_PROLIFERATION_GO_0008673 |
|  |  | LRRCC1 | GO_CELL_CYCLE |
|  |  | CENPE | GO_CELL_CYCLE |
|  |  | AURKB | GO_CELL_CYCLE |
| *Chen et al. 2017*  *PMID: [28093347]* | 3 invasive non-functioning PitNET vs. 4 invasive non-functioning PitNET; gene expression array | CLU^a^ | HALLMARK_APOPTOSIS |
|  |  | EZR^c^ | GO_CELL_CYCLE |
|  |  | KRT8 | GO_RESPONSE_TO_TUMOR_NECROSIS_FACTOR |
| *Yu et al. 2016*  *PMID: [26753958]* | 3 invasive non-functioning PitNET *vs*. 4 invasive non-functioning PitNET; gene expression array | EZR^c^ | GO_CELL_CYCLE |
| *De Araujo et al. 2017*  *PMID: [28382019]* | 4 non-invasive micro corticotroph PitNET vs. 5 non-invasive macro corticotroph PitNET *vs*. 3 invasive macro corticotroph PitNET; gene expression array | BMPR1B | KEGG_TGF_BETA_SIGNALING_PATHWAY |
|  |  | DBN1 | GO_CELL_GROWTH; GO_REGULATION_OF_EXTENT_OF_CELL_GROWTH; GO_POSITIVE_REGULATION_OF_CELL_GROWTH |
|  |  | JUN | HALLMARK_APOPTOSIS |
|  |  | TUBB2B | GO_CELL_CYCLE |
|  |  | CCND2^d^ | GO_CELL_CYCLE; HALLMARK_APOPTOSIS |
|  |  | DHCR24 | GO_CELL_CYCLE |
|  |  | CDKN1B^e^ | GO_CELL_CYCLE; GO_CELL_GROWTH; HALLMARK_APOPTOSIS; GO_NEGATIVE_REGULATION_OF_CELL_GROWTH; REGULATION_OF_CELL_GROWTH |
|  |  | NUPR1 | GO_CELL_CYCLE |
|  |  | NIN^f^ | GO_CELL_CYCLE; GO_CELL_GROWTH |
|  |  | CARD16 | GO_NEGATIVE_REGULATION_OF_TUMOR_NECROSIS_FACTOR_MEDIATED_SIGNALING_PATHWAY; GO_REGULATION_OF_TUMOR_NECROSIS_FACTOR_MEDIATED_SIGNALING_PATHWAY; GO_RESPONSE_TO_TUMOR_NECROSIS_FACTOR |
|  |  | STK35 | GO_CELL_CYCLE |
|  |  | FAS | ALCALA_APOPTOSIS; HALLMARK_APOPTOSIS; GO_RESPONSE_TO_TUMOR_NECROSIS_FACTOR |
|  |  | GPNMB | GO_CELL_CYCLE; CELL_PROLIFERATION_GO_0008506 |
|  |  | ZFYVE16 | KEGG_TGF_BETA_SIGNALING_PATHWAY |
|  |  | BRCC3 | GO_CELL_CYCLE |
|  |  | CDKN2A | GO_CELL_CYCLE; GO_CELL_GROWTH; CELL_PROLIFERATION_GO_0008381; GO_NEGATIVE_REGULATION_OF_CELL_GROWTH; REGULATION_OF_CELL_GROWTH |
|  |  | PRKACB | GO_CELL_CYCLE |
|  |  | CCDC88A | CELL_PROLIFERATION_GO_0008336 |
|  |  | PRPF19 | GO_CELL_CYCLE |
|  |  | CAMK1 | GO_CELL_CYCLE |
|  |  | AKAP12 | GO_POSITIVE_REGULATION_OF_TUMOR_NECROSIS_FACTOR_BIOSYNTHETIC_PROCESS; GO_POSITIVE_REGULATION_OF_TUMOR_NECROSIS_FACTOR_SECRETION; GO_RESPONSE_TO_TUMOR_NECROSIS_FACTOR |
|  |  | TIMP2 | GO_CELL_CYCLE; HALLMARK_APOPTOSIS |
|  |  | FHL1 | GO_CELL_CYCLE; GO_CELL_GROWTH; GO_NEGATIVE_REGULATION_OF_CELL_GROWTH |
|  |  | TCF7L2 | GO_CELL_CYCLE |
|  |  | PTPRJ | GO_CELL_GROWTH; GO_NEGATIVE_REGULATION_OF_CELL_GROWTH |
|  |  | TUBB4A | GO_CELL_CYCLE |
|  |  | CAV1 | HALLMARK_APOPTOSIS |
|  |  | GHSR | GO_NEGATIVE_REGULATION_OF_TUMOR_NECROSIS_FACTOR_BIOSYNTHETIC_PROCESS |
| *Galland et al. 2010*  *PMID: [20228124]* | 22 invasive gonadotroph PitNET *vs*. 18 non-invasive gonadotroph PitNET; gene expression array | UBE2L3 | GO_CELL_CYCLE |
|  |  | PPM1A | GO_CELL_CYCLE |
|  |  | SESN1 | CELL_PROLIFERATION_GO_0008697 |
|  |  | CLOCK | GO_CELL_CYCLE |
|  |  | CDKL5 | GO_CELL_CYCLE; GO_CELL_GROWTH; GO_POSITIVE_REGULATION_OF_CELL_GROWTH; GO_REGULATION_OF_EXTENT_OF_CELL_GROWTH |
|  |  | PRKAR1A | GO_CELL_CYCLE |
|  |  | FMN2 | GO_CELL_CYCLE |
|  |  | CHMP5 | GO_CELL_CYCLE |
|  |  | EEF1A1 | ALCALA_APOPTOSIS |
|  |  | THAP5 | GO_CELL_CYCLE |
| *Levka et al. 2012*  *PMID: [22585092]* | 8 low cadherin somatotroph PitNET *vs*. 8 high cadherin somatotroph PitNET; gene expression array | SLC44A4 | GO_CELL_GROWTH; GO_POSITIVE_REGULATION_OF_CELL_GROWTH |
|  |  | COBL | GO_CELL_GROWTH |
|  |  | CLU^a^ | HALLMARK_APOPTOSIS |
|  |  | CDKN1B^e^ | GO_CELL_CYCLE; GO_CELL_GROWTH; HALLMARK_APOPTOSIS; GO_NEGATIVE_REGULATION_OF_CELL_GROWTH; REGULATION_OF_CELL_GROWTH |
|  |  | GBP1 | GO_RESPONSE_TO_TUMOR_NECROSIS_FACTOR |
|  |  | SLIT3 | GO_CELL_GROWTH; GO_NEGATIVE_REGULATION_OF_CELL_GROWTH |
|  |  | CDK8 | GO_CELL_CYCLE |
|  |  | GBP2 | GO_RESPONSE_TO_TUMOR_NECROSIS_FACTOR |
|  |  | SMAD3 | GO_CELL_CYCLE; GO_CELL_GROWTH; GO_NEGATIVE_REGULATION_OF_CELL_GROWTH; REGULATION_OF_CELL_GROWTH; KEGG_TGF_BETA_SIGNALING_PATHWAY |
|  |  | ANLN | GO_CELL_CYCLE |
|  |  | CCND1 | GO_CELL_CYCLE; HALLMARK_APOPTOSIS |
|  |  | IGF2 | GO_CELL_CYCLE |
|  |  | DCLK1 | GO_CELL_GROWTH |
|  |  | KCNA5 | GO_CELL_CYCLE |
|  |  | CHMP4C | GO_CELL_CYCLE |
|  |  | NIN^f^ | GO_CELL_CYCLE; GO_CELL_GROWTH |
|  |  | IGF1R | CELL_PROLIFERATION_GO_0008520 |
|  |  | CCND2^d^ | GO_CELL_CYCLE; HALLMARK_APOPTOSIS |
|  |  | SORBS2 | GO_CELL_GROWTH |
|  |  | WFDC1 | GO_CELL_GROWTH; GO_NEGATIVE_REGULATION_OF_CELL_GROWTH |
|  |  | TRIM36^b^ | GO_CELL_CYCLE |
|  |  | PTPN3 | GO_CELL_CYCLE |
|  |  | IRF6 | GO_CELL_CYCLE |
|  |  | LGMN | GO_CELL_CYCLE |

Supplementary Table 6. Computational validation of the DEG results in an independent data set.

| **Signalling pathway** | **Discovery DEGs** | **Name of the gene** |
| --- | --- | --- |
| BUYTAERT PHOTODYNAMIC THERAPY STRESS UP | *THBD* | Thrombomodulin |
| CREIGHTON ENDOCRINE THERAPY RESISTANCE 5 | *BCAM* | Basal Cell Adhesion Molecule |
| CTCAGGG MIR125B MIR125A | *THBD* | Thrombomodulin |
| GO STEROID BIOSYNTHETIC PROCESS | *APBA2* | Amyloid Beta Precursor Protein Binding Family A Member 2 |
| GSE7852 TREG VS TCONV FAT UP | *APBA2* | Amyloid Beta Precursor Protein Binding Family A Member 2 |
| GSE9988 LPS VS LOW LPS MONOCYTE UP | *THBD* | Thrombomodulin |
| LET 7B 3P | *THBD* | Thrombomodulin |
| REACTOME NONSENSE MEDIATED DECAY NMD | *APBA2* | Amyloid Beta Precursor Protein Binding Family A Member 2 |

Supplementary Table 7. Levels of KMI67 expression in analysed sample group.

| **Sample** | **Therapy information** | **Counts** |
| --- | --- | --- |
| PA01 | SSA, DA/2 years | 26 |
| PA02 | SSA/1 year | 39 |
| PA03 | SSA/3 month | 11 |
| PA04 | SSA, DA/2 years | 2 |
| PA05 | SSA, DA/2 years | 26 |
| PA06 | SSA/3 month | 160 |
| PA07 | - | 44 |
| PA08 | - | 788 |
| PA09 | - | 229 |
| PA10 | - | 34 |
| PA11 | - | 177 |
| PA12 | - | 1257 |
